# Synergistic formal ring contraction for the enantioselective synthesis of spiropyrazolones†
†Electronic supplementary information (ESI) available. CCDC 1575685. For ESI and crystallographic data in CIF or other electronic format see DOI: 10.1039/c8sc00913a


**DOI:** 10.1039/c8sc00913a

**Published:** 2018-06-28

**Authors:** Marta Meazza, Martin Kamlar, Lucie Jašíková, Bedřich Formánek, Andrea Mazzanti, Jana Roithová, Jan Veselý, Ramon Rios

**Affiliations:** a Faculty of Natural & Environmental Sciences , University of Southampton , Highfield Campus , Southampton , SO17 1BJ , UK . Email: rrt1f11@soton.ac.uk; b Department of Organic Chemistry , Faculty of Science , Charles University , Hlavova 2030/8 , 128 43 , Prague 2 , Czech Republic; c Department of Industrial Chemistry “Toso Montanari” , School of Science , University of Bologna , Viale Risorgimento 4 , 40136 , Bologna , Italy

## Abstract

The rapid generation of molecular complexity from simple reactants is a key challenge in organic synthesis. Spiro compounds, underrepresented 3D motifs in chemical libraries, represent a challenge due to the creation of spiro quaternary carbon and the need to control the 3D shape in one step. Herein, we report the first ring contraction/formal [6 + 2] cycloaddition using synergistic Pd(0)/secondary amine catalysis, obtaining [5,5]-spiropyrazolone derivatives in excellent yields and stereoselectivities. We demonstrate that this reaction has a broad scope of early and late stage derivatization that will benefit the creation of highly valuable chemical libraries using spiropyrazolone motifs. We detected the key palladium activated intermediate in its protonated form by mass spectrometry and characterized its structure by infrared spectroscopy and DFT calculations, allowing us to propose a conceivable mechanistic pathway for this reaction.

## Abstract 2

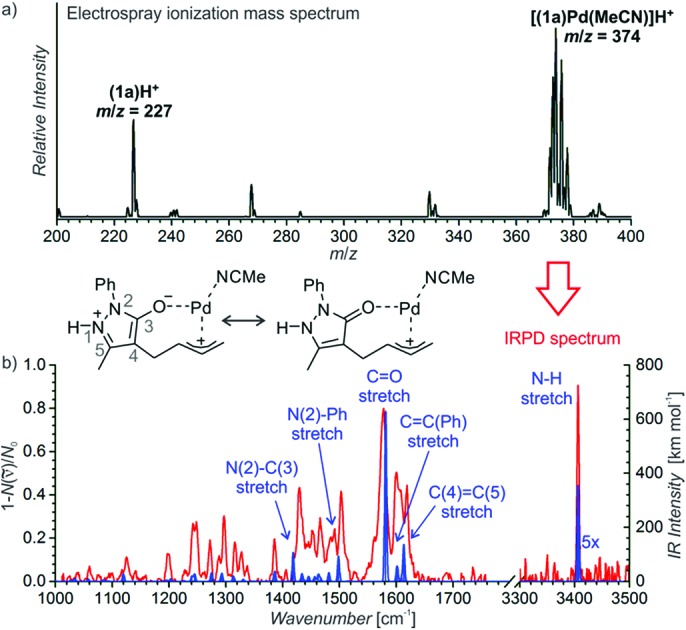
We report the first ring contraction/formal [6 + 2] cycloaddition using synergistic Pd(0)/secondary amine catalysis, obtaining [5,5]-spiropyrazolone derivatives in excellent yields and stereoselectivities. We detected the key palladium activated intermediate in its protonated form by mass spectrometry and characterized its structure by infrared spectroscopy and DFT calculations, allowing us to propose a conceivable mechanistic pathway for this reaction.

## Introduction

The enantioselective synthesis of carbon stereocenters with four different carbon substituents (called quaternary stereocenters) is a challenging aim. When the quaternary stereocenter is in between two rings, a spirocenter, it further complicates the synthesis. Among the various methodologies that have been developed to address this challenge, the organocatalytic synthesis of spiro-heterocycles with high stereoselectivities has been reported by several groups including ours.^1^,^2^ However, when the spiro center is contiguous with multiple tertiary stereocenters, the process usually requires several steps.

**Scheme 1:**
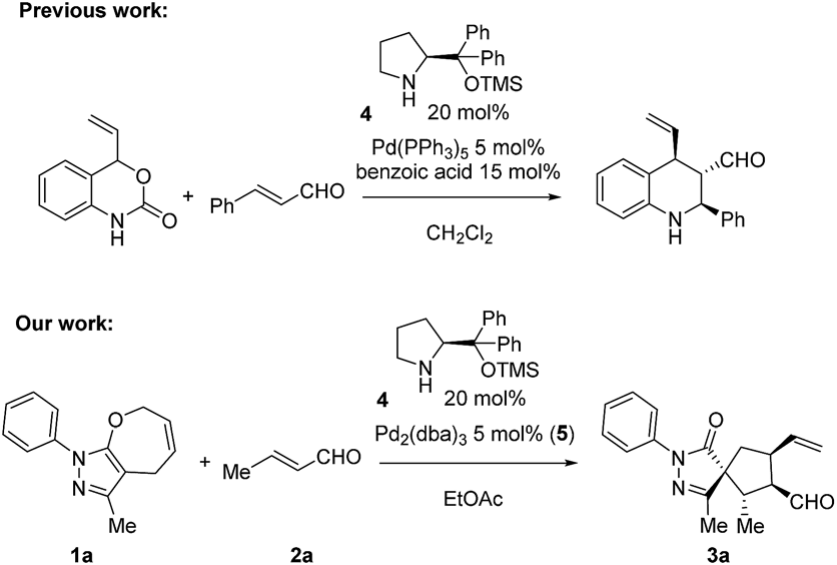
Top: Previous work by Jørgensen and coworkers;^10^ bottom: our work.

Synergistic catalysis^3^,^4^ has recently become one of the most promising strategies for the development of new reactions due to its versatility and the possibility to activate both starting materials at the same time, allowing for a wider scope and diminishing the necessity to use highly active starting materials. The advantage of synergistic catalysis is that both starting materials are activated at the same time by different catalysts. Thus, the activation energy of the reaction is smaller and, despite kinetic issues due to the need for two catalytic cycles, (the concentration of the active species is dependent on the concentration of catalysts), it allows reactions that are not possible otherwise. Moreover, synergistic catalysis presents several other advantages, such as the independent optimization of each catalyst and diminishing the time and costs of the optimization process, compared with bifunctional catalysts, making this approach highly attractive for synthetic chemists. Lately several groups have reported several synergistic cycloadditions for the formation of bicyclic and spiro products. Michelet,^5^ Jørgensen,^6^ Wei Wang^7^ and Rios^8^ reported a formal ring expansion of vinyl cyclopropanes with enals for the formation of spiro compounds under Pd(0) and secondary amine catalysis. Later on, Jørgensen reported vinyl aziridine opening^9^ and [4 + 2] decarboxylation^10^ (Scheme 1 top) using a synergistic approach with excellent results. Rios' group has been one of the pioneers in the use of synergistic catalysis merging transition metal catalysts/metal Lewis acid and amine catalysis. We developed a Michael addition of benzoxazoles^11^ and cyclopropanation of benzoxazoles^12^ with excellent results, these studies being the proof of concept that metal Lewis acid catalysis can coexist with secondary amine catalysis, opening a new gate to develop new reactions.

Interested in the synthesis of pyrazolones,^13^ we envisioned the development of a new synthesis of spiropyrazolones *via* synergistic catalysis. Pyrazolone scaffolds have widespread applications as pharmaceutical and agrochemical products, synthetic scaffolds in combinatorial and medicinal chemistry, dyes or chelating agents. Pyrazole moieties can be found in natural products like withasomnine and formycin and can also be found in a plethora of biologically active compounds such as HIV-1 integrase inhibitors, antibacterial agents, neuroprotective agents (edavarone X), pain killers and anti-inflammatory drugs (mavacoxib, a COX-2 inhibitor) or in fungicides.^14^–^17^ We and others have recently developed several methodologies for the synthesis and functionalization of these scaffolds.^18^–^22^

We focused our attention on the synthesis of spiropyrazolones bearing a carbocyclopentane ring. In contrast to cyclohexanes which are easily accessed by Diels–Alder reactions, the cyclopentane moiety represents a superior challenge, as few methodologies are available for its synthesis.^23^–^25^ With the advent of organocatalysis, several Michael initiated ring closure reactions have been developed.^26^–^30^ Other approaches for the synthesis of cyclopentanes are based on ring opening/expansion of vinyl cyclopropanes.^31^

Spiro compounds are still underrepresented in screening libraries and suffer from a relatively low diversity.^32^ The low occurrence of spiro motifs in modern drug discovery is not the result of their intrinsic adverse physico-chemical properties, but rather reflects the need for new strategies for their efficient synthesis and derivatization. Moreover, 3D/sp^3^-rich scaffolds provide more vectors for functionalization compared to common flat/aromatic scaffolds, overrepresented in many fragment libraries. We therefore envisioned a simple procedure for the synthesis of spiro-pyrazolones that allows for an early stage diversity. Moreover, the products can be further derivatized using reactive handles, thus achieving a late stage diversity. Here, we describe our efforts towards a successful synthesis of spiropyrazolones bearing multiple stereocenters in a controlled stereoselective fashion. This will allow for the fast development of chemical libraries with broad functionalization in a stereoselective form, which will help identify new hits for pharmaceutical targets.

## Results and discussion

A key to our approach is the development of formal ring contraction based on the synergistic activation of pyrazolone derivatives and enals. We envisioned that 7-membered ring **1a** is activated by a transition metal that would favour the opening of the ring, while the enal is activated by a secondary amine catalyst to furnish a [5,5]spiropyrazolone. Based on these premises, we tested the reaction between the pyrazolone derivative **1a** and the α,β-unsaturated aldehyde **2a** (R = Me), where the 7-membered ring **1a** undergoes formal [6 + 2] cycloaddition, forming a cyclopentanone by ring contraction in which the original [5,7]-bicyclic ring transforms into a [5,5]-spiro bicyclic ring (Scheme 1, bottom).

**Scheme 2:**
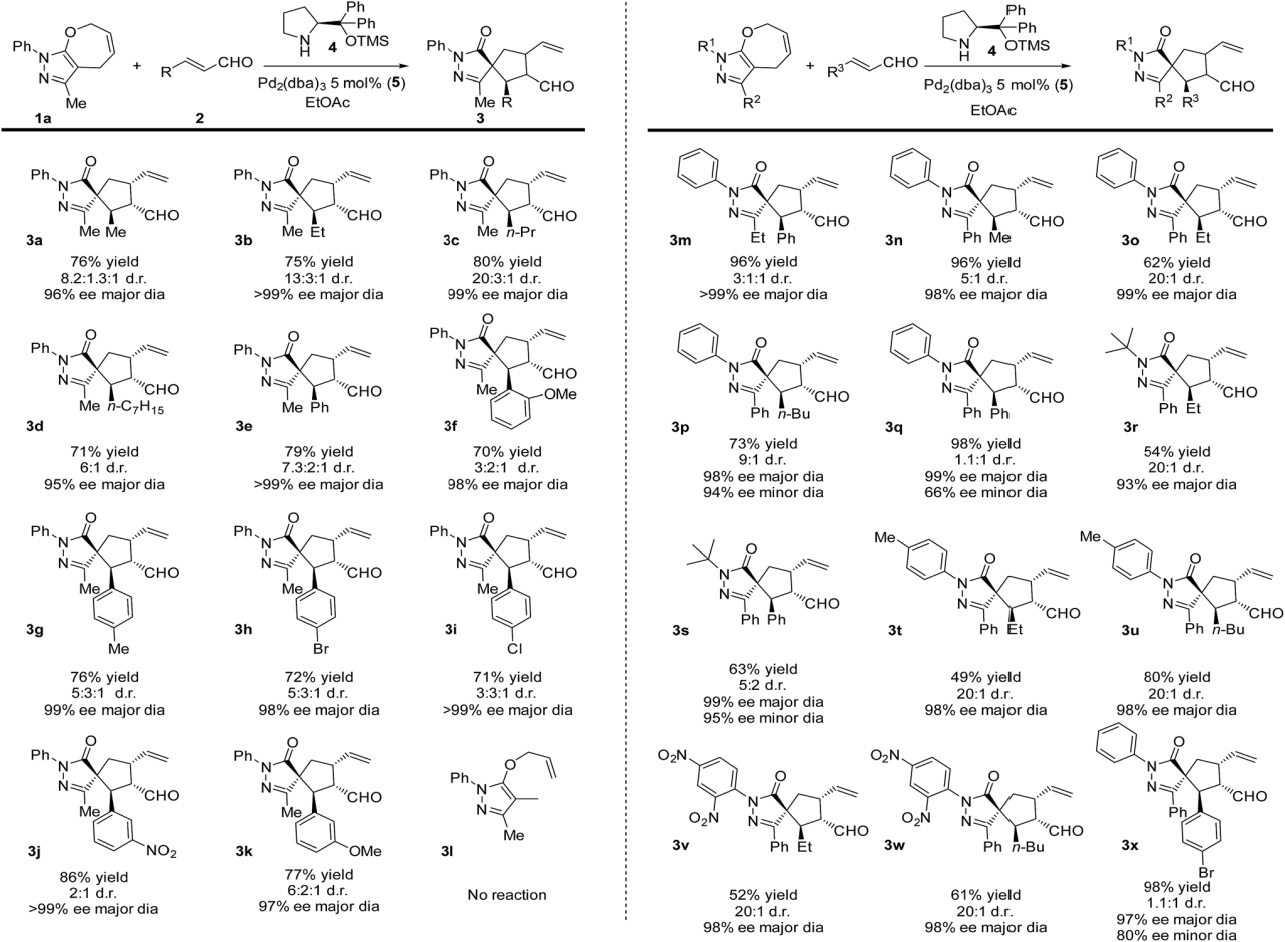
Reaction scope of pyrazolones **1** and α,β-unsaturated aldehyde **2**.

Employing the optimized reaction conditions (see the ESI†), with 5 mol% of Pd_2_(dba)_3_ and 20 mol% secondary amine catalyst **4**, the final spiro compound **3a** was obtained in 76% yield, 8 : 1.3 : 1 d.r. and 96% ee. With the optimized conditions in hand, we studied the scope of the reaction using pyrazolone **1a** (R^2^ = Me) and different enals. As shown in Scheme 2, when aliphatic aldehydes were used, the products **3a–d** were obtained in high yields (71–80%) and excellent diastereo- and enantioselectivities, up to 99% ee. The reaction also proceeds well with aromatic enals substituted with halogens, EWG and EDG in *ortho*, *meta* and *para* positions, and the products **3e–k** were obtained in high yields and enantioselectivities with moderate diastereoselectivities.

Next, we focused our attention on the pyrazolone moiety. Ethyl functionalized, product **3m** was obtained in 96% yield, moderate d.r. and >99% ee. When R^2^ was a phenyl ring and reacted with aliphatic aldehydes, the products **3n–p** were obtained in high yields and stereoselectivities, while lower diastereoselectivities were observed with aromatic aldehydes (products **3q,x**). Then, the scope of the reaction regarding the R^1^ substituent was examined. When R^1^ was a *tert*-butyl group, the reaction gave lower yields compared to the aromatic counterparts but still maintained excellent enantioselectivities. As before, lower diastereoselectivities were recorded using an aromatic aldehyde (**3s**), compared to the aliphatic one (**3r**). Finally, aryls substituted with EWG or EDG were tested, giving the products **3t–w** in 49 to 80% yield, 20 : 1 diastereoselectivities and 98% ee. In order to expand the scope of the reaction, we tested the noncyclic compound **3l**. No reaction was observed, probably due to some conformational issues that could prevent the cyclization.

**Fig. 1:**
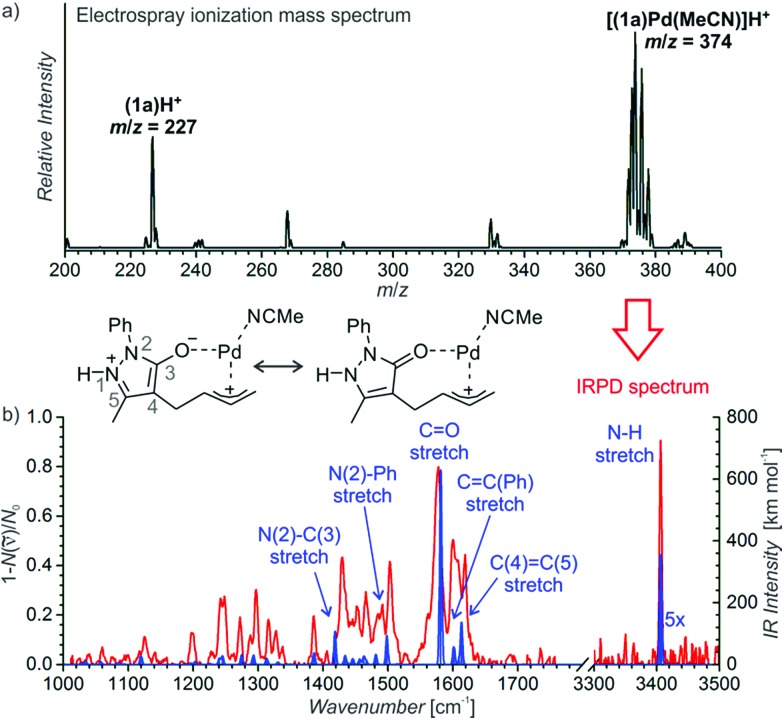
(a) ESI-MS spectrum of 9 mM solution of **1a** in CH_3_CN/CH_2_Cl_2_ (4 : 1 v/v) with 2.5 mol% Pd_2_(dba)_3_. (b) Helium tagged infrared photodissociation spectrum of [(**1a**)Pd(CH_3_CN)]H^+^ (*m*/*z* 374; red line) and theoretically predicted spectrum (blue line) of the depicted complex.

We have probed possible reaction intermediates by electrospray ionization mass spectrometry (ESI-MS).^33^–^35^ ESI-MS of the whole reaction mixture reveals only signals of the protonated Jørgensen catalyst and the iminium ions formed from the catalyst and the aldehydes (reactants or products) (Fig. S20† in the ESI). Next to these strong signals, it was impossible to detect palladium complexes that are probably neutral in solution and have to be protonated for ESI-MS detection. In order to find out about the role of palladium, we have investigated a solution of the palladium catalyst and reactant **1a** alone (Fig. 1a). The dominant signal in the ESI-MS spectrum is that of the protonated palladium complex of **1a** bearing one molecule of acetonitrile ([(**1a**)Pd(CH_3_CN)]H^+^, *m*/*z* 374). We did not detect any other significant signals of ions containing palladium atom(s) (Fig. S21† in the ESI).

The structure of the palladium complex [(**1a**)Pd(CH_3_CN)]H^+^ can be derived from its infrared photodissociation (IRPD) spectrum.^36^–^38^ The IRPD spectrum shows an intense band at about 3400 cm^–1^ that corresponds to the N–H stretching vibration and implies that the complex is protonated at the nitrogen atom of the five-membered ring. The finger-print range of the spectrum corresponds well with that of the theoretically predicted spectrum of the ion 6H^+^ (Fig. 1b). This ion can be formed by protonation of a molecule resulting from the insertion of palladium into the carbon–oxygen bond of the seven-membered ring. Conversely, all the forms of the palladium complexes of reactant **1a** with a preserved seven-membered ring have distinctly different IR spectra and can be excluded (Fig. S22–S25† in the ESI). We have also investigated other possible structures, but they all are higher in energy than 6H^+^, and their IR spectra are not in agreement with the experimental results (Fig. S22–S25† in the ESI).

**Fig. 2:**
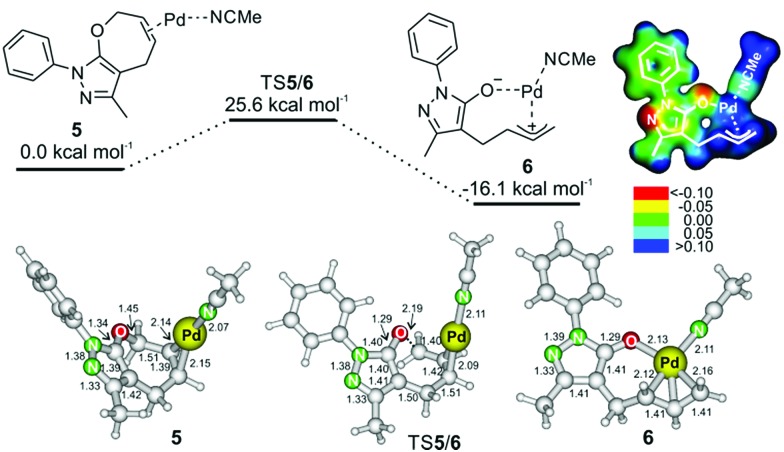
Potential energy surface for the insertion of palladium into the C–O bond (method: B3LYP/6-311++G**(SDD for Pd) and implicit solvation in acetonitrile with the SMD model). The ball and stick models show structures of intermediates **5** and **6**; the distances are in Å. The electrostatic potential map of **6** is color-coded on the isodensity surface of *ρ* = 0.02 e Å^–3^.

A possible reaction path towards intermediate **6** starts by coordination of palladium to reactant **1a** (Fig. 2). The most energetically favored coordination of palladium occurs at the double bond of the seven-membered ring of **1a**. Palladium interacts not only with the C–C double bond, but also with one molecule of acetonitrile (complex **5** in Fig. 2, top; we note that coordination of a second acetonitrile molecule to palladium is endothermic – Fig. S26† in the ESI). Complex **5** rearranges *via* transition structure **TS5/6** to form intermediate **6**. The process of the palladium insertion is exothermic by about 16 kcal mol^–1^.

The electrostatic potential of intermediate **6** shows that the negative charge is mostly localized on the oxygen atom and on the N(1) and C(4) atoms of the heterocycle (see Fig. 2). Accordingly, the protonation proceeds at the N(1) atom, and the reaction with the iminium ion will occur at the C(4) atom. Analogous intermediates were previously suggested in other palladium catalyzed reactions, but this is the first time such an intermediate was trapped, and the structure of its protonated form was experimentally confirmed.

**Scheme 3:**
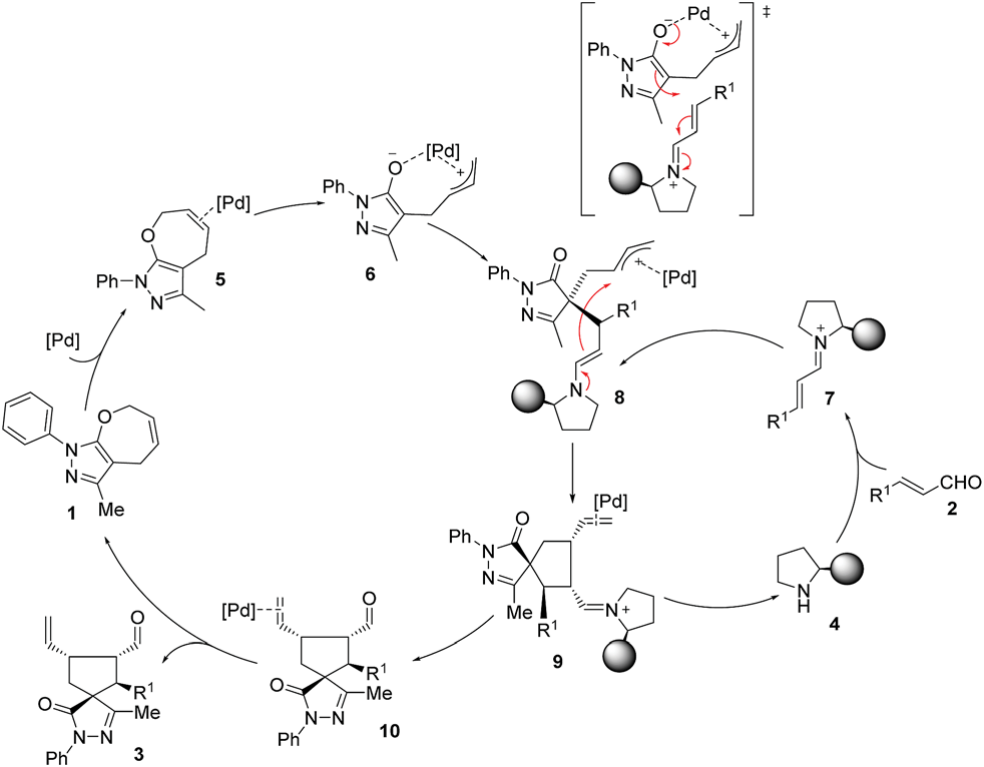
Proposed reaction mechanism.

Based on computational predictions and experimental evidence, we propose a reaction pathway (Scheme 3) which can be outlined as: (i) activation of both reactants to yield highly reactive palladium complex **6** and iminium ion **7** where the Jørgensen–Hayashi catalyst efficiently blocks the upper face of the ion; (ii) coupling between the activated intermediates **6** and **7**. The palladium intermediate **6** reacts as a nucleophile with **7** to form the enamine intermediate **8** that contains the palladium coordinated allyl cation. The ring closure step involves the reaction between the enamine moiety and the allyl cation. Finally, the hydrolysis of the iminium complex **9** regenerates the secondary amine catalyst **4** and provides the palladium complex of product **10**. Decoordination of palladium yields the final compound **3** with the observed stereochemistry and regenerates the catalysts for another synergistic cycle.

The relative and absolute configuration of compounds **3** was derived by means of NMR and chiro-optical spectra, using compounds ent-**3n** and ent-**3g** synthesised using (*R*)-**4** as examples. NOE-NMR spectra and coupling constant analysis allowed us to determine that the major diastereoisomer (in both compounds) corresponds to the 1R*,2S*,3R*,4S* relative configuration, C1 being the spirocenter and C5 being CH_2_. In the same way, the 1R*,2S*,3R*,4R* relative configuration was assigned to the minor diastereoisomer. Being viscous liquids, the absolute configuration was assigned using the theoretical simulations of the electronic circular dichroism (ECD) spectra, based on TD-DFT calculations.^39^,^40^

**Fig. 3:**
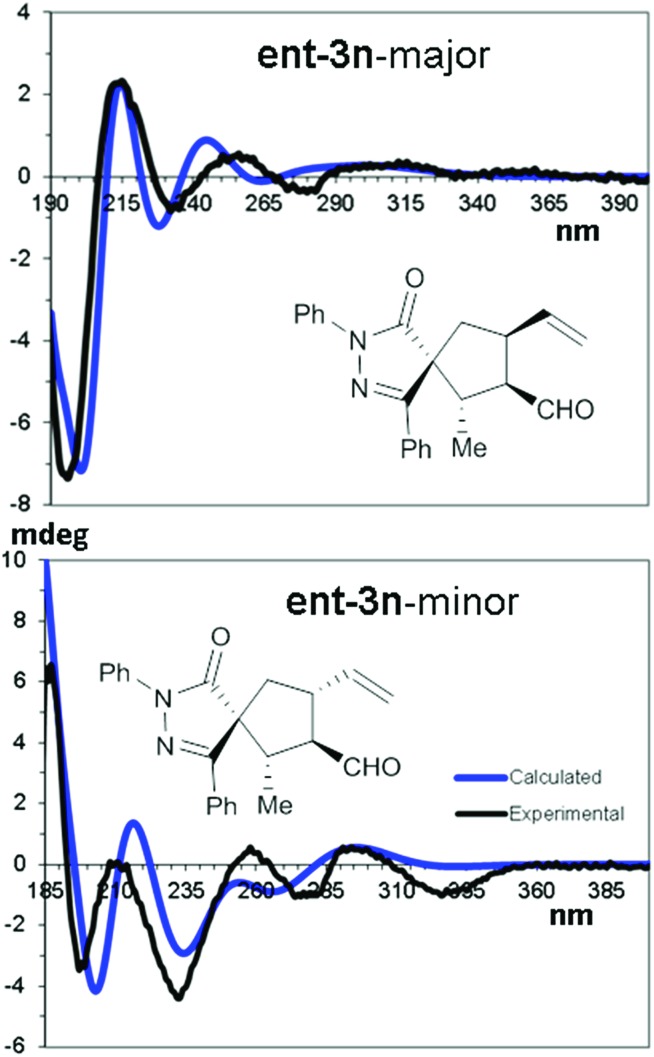
TD-DFT simulations of the major and minor diastereoisomers of ent-**3n**. Calculated TD-DFT spectra were obtained at the CAM-B3LYP/(6-311++G(2d,p) level.

Conformational analysis using the molecular mechanics scan of the potential energy surface (PES) allowed us to find the best ground state geometries, that were then optimized by DFT at the B3LYP/6-31G(d) level of theory and including the solvent with the PCM method.^41^ TD-DFT calculations were then run on four optimized geometries using different functionals (CAM-B3LYP, M06-2X, BH&HLYP, and ΩB97XD) and the 6-311++G(2d,p) basis set to simulate the ECD spectrum of each conformation. The resulting averaged ECD spectrum was obtained by mixing the four ECD spectra using the ratio suggested by DFT optimization (Fig. 3).

The comparison of the simulated spectra with the experimental ones allowed us to assign the 1*R*,2*S*,3*R*,4*S* absolute configuration to the major diastereoisomer of ent-**3n** and the 1*R*,2*S*,3*R*,4*R* configuration to the minor one (full details can be found in the ESI†).

**Scheme 4:**
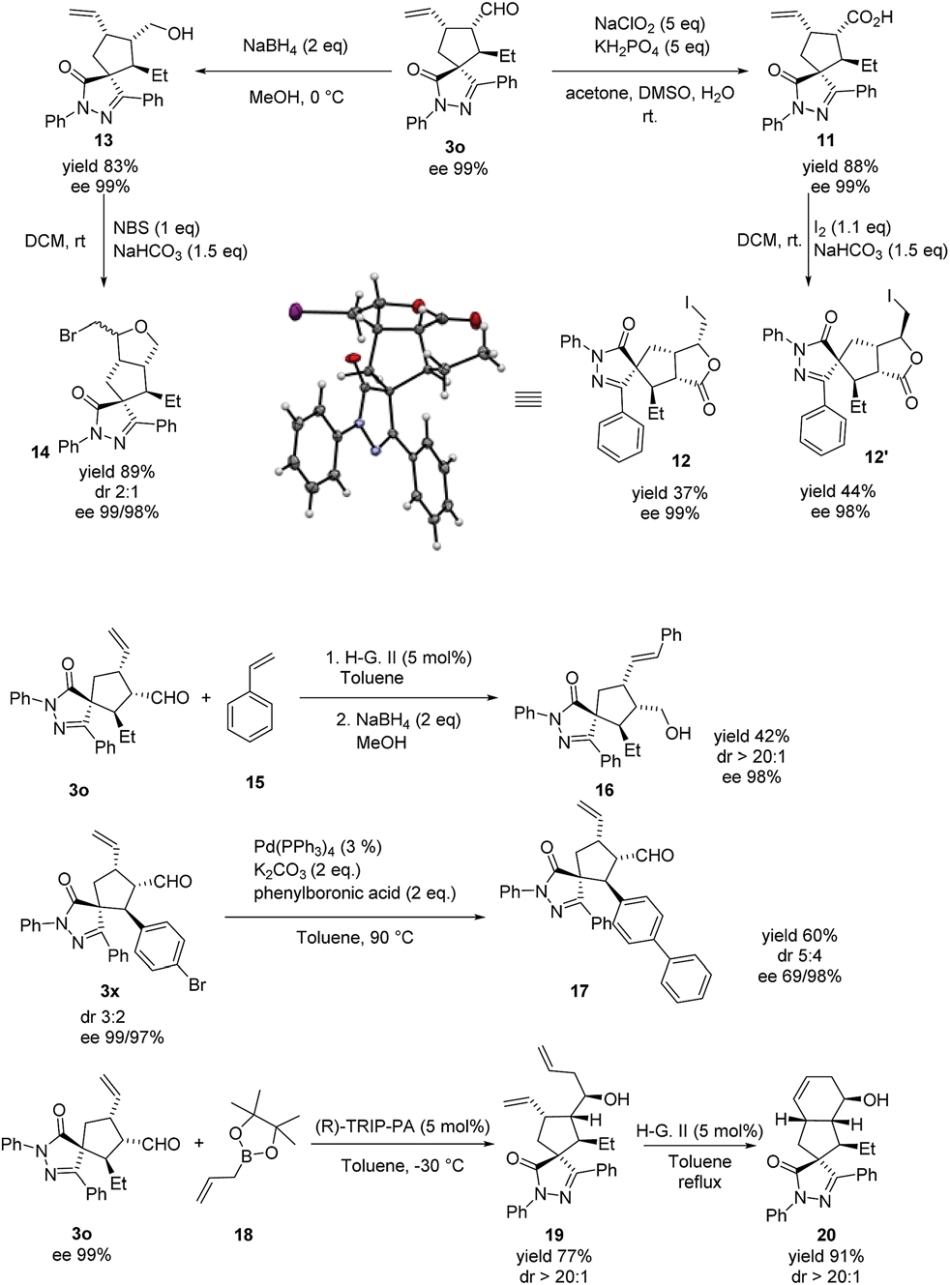
Late stage derivatizations.

The absolute configuration of the major diastereoisomer was confirmed by the X-ray diffraction analysis^42^ of compound **12**, derived from compound **3o** (see Scheme 4).

As stated in the introduction, our objective is to provide an effective and useful platform for the synthesis of spiropyrazolone scaffolds that can be used in screening libraries. These compounds can be easily derivatized in the late stage by using the reactive handles provided by our methodology (alkene and aldehyde, Scheme 4). An example of such derivatization is the reduction of **3o** to compound **13**. Cyclization of **13** by treatment with NBS yielded the polycyclic compound **14** in good yield and a 2 : 1 diastereoselective ratio, retaining the optical purity. In a similar manner, oxidation and cyclization of **3o** give compounds **11** and **12**, respectively, in good yields without loss of optical purity, (the cyclization step gives a 1.1 : 1 mixture of diastereomers). Another example of late stage transformation is a metathesis reaction of **3o** with styrene to afford compound **16** in moderate yield and excellent stereoselectivity. Then, we tested a Suzuki reaction involving the bromine substituted starting enal **3x**. The coupling product **17** was obtained in excellent yield. Finally, we allylated compound **3o** at the aldehyde function to give **19** in good yield and excellent diastereoselectivity. The compound **19** was transformed, by intramolecular ring closing metathesis, to the polycyclic compound **20** with complete retention of the optical purity.^43^

## Conclusions

In conclusion, we reported a new formal ring contraction based on the ring-opening–ring-closing reaction of pyrazolone derivatives. We show that it is possible to merge two catalyzed reaction pathways, leading to spiropyrazolones through synergistic catalysis. The synergistic reaction is efficiently catalyzed by a combination of Pd(0) and secondary amine catalysts, providing spiropyrazolones in high yields and stereoselectivities. Moreover, we proved that this methodology can be used to implement screening libraries with new spiro compounds due to their easy early and late stage diversity introduction.

## Conflicts of interest

There are no conflicts to declare.

## Supplementary Material

Supplementary information

Crystal structure data

## References

[cit1] Krapcho A. P. (1978). Synthesis.

[cit2] Rios R. (2012). Chem. Soc. Rev..

[cit3] Allen A., MacMillan D. W. C. (2012). Chem. Sci..

[cit4] Meazza M. Rios R., Synthesis, 2016, 48 , 960 –973 , ; and references therein .

[cit5] Laugeous M., Ponra S., Ratovelomanana-Vidal V., Michelet V., Vitale M. R. (2016). Chem. Commun..

[cit6] Halskov K. S., Naesborg L., Tur F., Jørgensen K. A. (2016). Org. Lett..

[cit7] Zhu H., Du P., Li J., Liao Z., Liu G., Li H., Wang W. (2016). Beilstein J. Org. Chem..

[cit8] Meazza M., Rios R. (2016). Chem.–Eur. J..

[cit9] Naesborg L., Tur F., Meazza M., Blom J., Halskov K. S., Jørgensen K. A. (2017). Chem.–Eur. J..

[cit10] Leth L. A., Glaus F., Meazza M., Fu L., Thorgersen M. K., Bitsch E. A., Jørgensen K. A. (2016). Angew. Chem., Int. Ed..

[cit11] Meazza M., Ceban V., Pitak M. B., Coles S. J., Rios R. (2014). Chem.–Eur. J..

[cit12] Meazza M., Light M. E., Mazzanti A., Rios R. (2016). Chem. Sci..

[cit13] Meazza M., Rios R. (2016). Chem.–Eur. J..

[cit14] Varvounis G., Fiamegos Y. and Pilidis G., Advances in Heterocyclic Chemistry, ed. A. R. Katritzky, Academic Press Inc, 2001, vol. 80, pp. 73–156.

[cit15] Janin Y. L. (2007). Bioorg. Med. Chem..

[cit16] Gutierrez-Lugo M.-T., Bewley C. A. (2008). J. Med. Chem..

[cit17] Matthews I. R., PCT Int. Appl. WO 46679, 2005.

[cit18] Kimata A., Nakagawa H., Ohyama R., Fukuuchi T., Ohta S., Suzuki T., Miyata N. (2007). J. Med. Chem..

[cit19] Zea A., Alba A.-N. R., Mazzanti A., Moyano A., Rios R. (2011). Org. Biomol. Chem..

[cit20] Alba A.-N. R., Zea A., Valero G., Calbet T., Font-Bardia M., Mazzanti A., Moyano A., Rios R. (2011). Eur. J. Org. Chem..

[cit21] Šimek M., Remeš M., Veselý J., Rios R. (2013). Asian J. Org. Chem..

[cit22] Kamlar M., Císařová I., Veselý J. (2015). Org. Biomol. Chem..

[cit23] Chauhan P. Mahajan S. Enders D., Chem. Commun., 2015, 51 , 12890 –12907 , ; and references therein . 10.1039/c5cc04930j 26178319

[cit24] The Pauson-Khand reaction: Scope, variations and applications, ed. R. Rios, John Wiley & Sons Ltd, 2012.

[cit25] Wenz D. R., Read de Alaniz J. (2015). Eur. J. Org.Eur. J. Org.
Chem.Chem..

[cit26] Tokimizu Y., Wieteck M., Rudolph M., Oishi S., Fujii N., Hashmi A. S. K., Ohno H. (2015). Org. Lett..

[cit27] Ibrahem I., Zhao G.-L., Rios R., Vesely J., Sunden H., Dziedzic P., Cordova A. (2008). Chem.–Eur. J..

[cit28] Rios R., Vesely J., Sunden H., Ibrahem I., Zhao G.-L., Cordova A. (2007). Tetrahedron Lett..

[cit29] Wang J., Li H., Xie H., Zu L., Shen X., Wang W. (2007). Angew. Chem., Int. Ed..

[cit30] Enders D., Wang C., Bats J. W. (2008). Angew. Chem., Int. Ed..

[cit31] Remeš M., Veselý J. (2012). Eur. J. Org. Chem..

[cit32] Meazza M., Guo H., Rios R. (2017). Org. Biomol. Chem..

[cit33] Zheng Y., Tice C. M., Singh S. B. (2014). Bioorg. Med. Chem. Lett..

[cit34] Iacobucci C., Reale S., De Angelis F. (2016). Angew. Chem., Int. Ed..

[cit35] Jašíková L., Anania M., Hybelbauerová S., Roithová J. (2012). J. Am. Chem. Soc..

[cit36] Roithová J. (2012). Chem. Soc. Rev..

[cit37] Škríba A., Schulz J., Roithová J. (2014). Organometallics.

[cit38] Roithová J., Gray A., Andris E., Jašík J., Gerlich D. (2016). Acc. Chem. Res..

[cit39] Pescitelli G., Di Bari L., Berova N. (2011). Chem. Soc. Rev..

[cit40] Mazzanti A., Casarini D. (2012). WIREs Comput. Mol. Sci..

[cit41] Tomasi J., Mennucci B., Cammi R. (2005). Chem. Rev..

[cit42] CCDC 1575685 contain the crystallographic data for this paper. These data are provided free of charge by The Cambridge Crystallographic Data Centre.

[cit43] The stereochemistry of the new formed alcohol is in accordance with: Jain P. Antilla J. C., J. Am. Chem. Soc., 2010, 132 , 11884 –11886 . 20690662 10.1021/ja104956s PMC2928988

