# Supplementary material for: Synergistic formal ring contraction for the enantioselective synthesis of spiropyrazolones
Source: Chem Sci. 2018 Jun 28;9(30):6368–73. doi: 10.1039/c8sc00913a (PMC6115638; doi:10.1039/c8sc00913a)
Supplement: Supplementary file 1 [file SC-009-C8SC00913A-s001.pdf]

Electronic Supplementary Material (ESI) for Chemical Science.
This journal is © The Royal Society of Chemistry 2018

Synergistic formal ring contraction for the enantioselective synthesis of spiropyrazolones

Marta Meazza, Martin Kamlar, Lucie Jašíková, Bedřich Formánek, Andrea Mazzanti, Jana Roithová,*
Jan Veselý,* Ramon Rios *

Supporting information

Table of Contents

1. General information 2
2. Reaction Optimization 3
3. Synthesis of starting materials 4
3.1. General procedure for the synthesis of pyrazolones **1** 4
3.2. General procedure for the synthesis of 7 membered ring derivatives **1** 4
3.3. Characterization data for new compounds 4
4. Synthesis of the final spiro compounds 6
4.1. General procedure for the ring contraction for products **3** 6
4.2. Modified procedure for products **3o-p**, **3r**, **3t-w** 6
4.3. Characterization of compounds **3** 6
5. Derivatizations 16
5.1. Synthesis of **11** 16
5.2. Synthesis of **12** 16
5.3. Synthesis of **13** 17
5.4. Synthesis of **14** 18
5.5. Synthesis of **16** 19
5.6. Synthesis of **17** 20
5.7. Synthesis of **19** and **20** 21
7. NMR spectra starting material 41
8. NMR spectra spiro compounds 47
9. HPLC traces 84
9. Mass spectrometry experiments 114
10. Infrared photodissociation (IRPD) spectroscopy and theoretical calculations 116

1. General information

Chemicals and solvents were either purchased (puriss p.A.) from commercial suppliers or purified by standard techniques. For thin-layer chromatography (TLC), silica gel plates Merck 60 F254 were used, and compounds were visualized by irradiation with UV light and/or by treatment with a solution of phosphomolybdenic acid (25 g), $Ce(SO_4)_2{\cdot}H_2O$ (10 g), conc. H_2SO_4 (60 mL), and H_2O (940 mL) followed by heating. Thin layer chromatography (TLC) was performed on Merck TLC Silicagel 60 F254. The products' spots were visualized by UV-light at 254 nm. Column chromatography was performed using silica gel (Geduran Si60, 40-63 µm). ^{1}H-NMR, ^{13}C-NMR, ^{19}F-NMR, 2D-NMR were recorded with a Bruker DPX400 NMR or with a FT-NMR spectrometer Bruker AVANCE III 600 MHz. Chemical shifts (δ) are reported in ppm relative to residual solvent signals ($CHCl_3$, 7.26 ppm for ^{1}H NMR; $CDCl_3$, 77.00 ppm for ^{13}C NMR). ^{19}F NMR spectra were acquired in proton-decoupled mode. HRMS were recorded using a MaXis (Bruker Daltonics, Bremen, Germany) mass spectrometer equipped with a Time of Flight (TOF) analyzer. Optical rotations were performed on an Optical Activity PolAAr 2001 machine. The HPLC analysis were performed on a Perkin Elmer Flexar HPLC and on an Agilent 1220 Infinity LC system HPLC or on a LC20AD Shimadzu liquid chromatograph with SPD-M20A diode array detector with columns Daicel Chiralpak.

2. Reaction Optimization

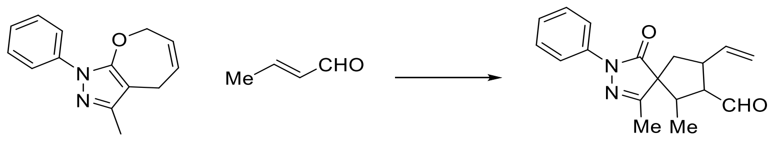

Entry	Solvent	T	Conversion 24 h[c]	d.r.	ee major
1	Toluene	r.t.	31%	8.2:0.6:1	97%
2	CH_3CN	r.t.	61%	22.5:1.7:1	88%
3	CH_2Cl_2	r.t.	62%	11.9:0.9:1	90%
4	DMSO	r.t.	traces	---	---
5	THF	r.t.	38%	4.8:0.9:1	96%
6	EtOAc	r.t.	91%	8:1.3:1	96%
7	DMF	r.t.	27%	4.4:1	38%
8	$CHCl_3$	r.t.	85%	3.3:0.4:1	94%

Table S1: Screening of the solvents

3. Synthesis of starting materials

3.1. General procedure for the synthesis of pyrazolones **1**

Pyrazolone 2-methyl-5-phenyl-2,4-dihydro-3H-pyrazol-3-one was purchased from commercial source. Pyrazolone 2,5-diphenyl-2,4-dihydro-3H-pyrazol-3-one was prepared according published procedure.[1] Spectroscopic data correspond with data published in literature.[2] Pyrazolone 2-(*tert*-butyl)-5-phenyl-2,4-dihydro-3H-pyrazol-3-one was prepared and characterized according published procedure.[1] Pyrazolone 5-phenyl-2-(*p*-tolyl)-2,4-dihydro-3H-pyrazol-3-one was prepared according published procedure[1] and characterized according published data.[3] Pyrazolone 2-(2,4-dinitrophenyl)-5-phenyl-2,4-dihydro-3H-pyrazol-3-one was prepared and characterized according to published procedure.[4] Pyrazolone 2-ethyl-5-phenyl-2,4-dihydro-3H-pyrazol-3-one was prepared[1] and characterized according to published procedure.[4]

3.2. General procedure for the synthesis of 7 membered ring derivatives **1**

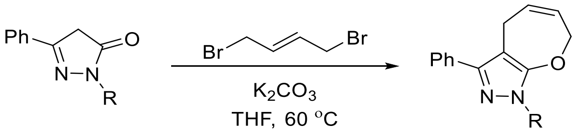

Scheme S1: Synthesis of the starting materials

A mixture of 1,3-indanedione (1 eq), 1,4-dibromobut-2-ene (1 eq), and K_2CO_3 (2.5 eq) in THF was stirred at 60 °C under nitrogen overnight. After completion of the reaction (monitored by ^{1}H-NMR), the mixture was cooled to room temperature and filtered with $CHCl_3$ over celite. The organic phase was washed with water and brine. The organic layers were separated, dried with $MgSO_4$ and concentrated by *vacuum*. The crude product was purified by column chromatography over silica gel (eluent: hexane/EtOAc) to afford the product.

3.3. Characterization data for new compounds

1-methyl-3-phenyl-4,7-dihydro-1H-oxepino[2,3-c]pyrazole,1a

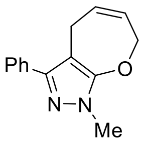

Brown oil, yield 50%. **^{1}H NMR (400 MHz, CDCl$_3$)** δ 7.68 – 7.60 (m, 2H), 7.42 – 7.35 (m, *J* = 8.3, 7.6 Hz, 2H), 7.24 – 7.18 (m, 1H), 6.20 (dt, *J* = 10.7, 5.3 Hz, 1H), 6.02 – 5.92 (m, 1H), 4.66 (dd, *J* = 6.3, 0.6 Hz, 2H), 3.39 – 3.24 (m, 2H), 2.19 (s, 3H). **^{13}C NMR (101 MHz, CDCl$_3$)** δ 152.2, 147.2, 138.7, 134.2, 128.7 (2C), 125.8, 125.7, 121.9 (2C), 97.8, 67.3, 23.1, 12.6. **HRMS** (ESI) *m/z* calcd for $C_{14}H_{15}N_2O$ $[M+H]^+$ = 227.1179; found = 227.1178.

[1] R. Ramajayam, K.-P. Tan, H.-G. Liu, P.-H. Liang, *Bioorg. Med. Chem.* **2010**, *18*, 7849–7854

[2] H. Clavier, L. Giordano, A. Tenaglia, *Angew. Chem. Int. Ed.* **2012**, *51*, 8648 –8651.

[3] D. Castagnolo, A. De Logu, M. Radi, B. Bechi, F. Manetti, M. Magnani, S. Supino, R. Meleddu, L. Chisu, M. Botta, *Bioorg. Med. Chem.* **2008**, *16*, 8587–8591.

[4] D. N. Kuznetsov, A. G. Ruchkina, K. I. Kobrakov, *Chem. Heterocycl. Compd.* **2011**, *47*, 441-447.

1-ethyl-3-phenyl-4,7-dihydro-1H-oxepino[2,3-c]pyrazole, 1b

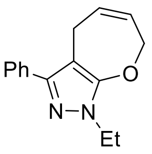

Orange oil, yield 49%. **^{1}H NMR (400 MHz, CDCl$_3$)** δ 7.69 – 7.64 (m, 2H), 7.42 – 7.37 (m, 2H), 7.25 – 7.19 (m, 1H), 6.29 – 6.14 (m, 1H), 6.06 – 5.91 (m, 1H), 4.68 (dd, *J* = 6.3, 0.7 Hz, 2H), 3.44 – 3.28 (m, 2H), 2.58 (q, *J* = 7.6 Hz, 2H), 1.27 (t, *J* = 7.6 Hz, 3H). **^{13}C NMR (101 MHz, CDCl$_3$)** δ 152.4, 134.2, 128.7, 126.3, 125.8, 122.1, 118.6, 97.0, 67.2, 22.9, 20.7, 13.2. **HRMS** (ESI) *m/z* calcd for $C_{15}H_{17}N_2O$ [M+H]$^+$ = 241.1335; found = 241.1337.

1,3-diphenyl-4,7-dihydro-1H-oxepino[2,3-c]pyrazole, 1c

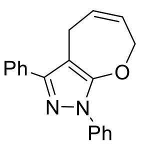

Yellowish oil, yield 40%; **^{1}H NMR (600 MHz, CDCl$_3$)** δ 7.77 – 7.75 (m, 2H), 7.66 – 7.63 (m, 2H), 7.47 – 7.40 (m, 4H), 7.39 – 7.35 (m, 1H), 7.30 – 7.25 (m, 1H), 6.19 (dt, J_1 = 10.8, J_2 = 5.4 Hz, 1H), 6.01 (dddd, J_1 = 10.8, J_2 = 8.1, J_3 = 5.5, J_4 = 3.2 Hz, 1H), 4.76 (d, *J* = 6.4 Hz, 2H), 3.56 (dd, J_1 = 5.4, J_2 = 1.6 Hz, 1H). **^{13}C NMR (151 MHz, CDCl$_3$)** δ 152.8, 150.1, 138.8, 134.7, 133.9, 128.9 (2C), 128.6 (2C), 128.1 (2C), 128.0, 126.4, 125.9, 122.5 (2C), 97.4, 67.2, 24.0. **HRMS** (ESI) *m/z* calcd for $C_{19}H_{17}N_2O$ [M+H]$^+$ = 289.133540; found = 289.133426.

1-(tert-butyl)-3-phenyl-4,7-dihydro-1H-oxepino[2,3-c]pyrazole, 1d

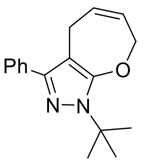

Orange oil, yield 22%; **^{1}H NMR (600 MHz, CDCl$_3$)** δ 7.59 – 7.53 (m, 2H), 7.38 (dd, J_1 = 8.4, J_2 = 7.0 Hz, 1H), 7.31 – 7.27 (m, 1H), 6.07 – 6.03 (m, 1H), 5.89 (dtt, J_1 = 11.3, J_2 = 5.8, 1.8 Hz, 1H), 4.68 – 4.60 (m, 2H), 3.54 – 3.47 (m, 2H), 1.62 (s, 9H). **^{13}C NMR (151 MHz, CDCl$_3$)** δ 153.3, 146.00, 134.8, 132.9, 128.6, 128.5 (2C), 127.9 (2C), 127.2, 125.8, 98.5, 67.3, 58.9, 29.3 (3C), 24.6. **HRMS** (ESI) *m/z* calcd for $C_{17}H_{21}N_2O$ [M+H]$^+$ = 269.164840; found = 269.164796.

3-phenyl-1-(p-tolyl)-4,7-dihydro-1H-oxepino[2,3-c]pyrazole, 1e

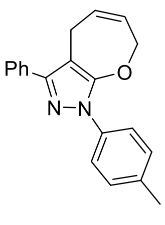

Yellowish oil, yield 65%; **^{1}H NMR (600 MHz, CDCl$_3$)** δ 7.66 – 7.59 (m, 4H), 7.43 (dd, J_1 = 8.4, J_2 = 6.9 Hz, 2H), 7.38 – 7.33 (m, 1H), 7.22 (d, *J* = 8.2 Hz, 1H), 6.18 (dt, J_1 = 10.8, J_2 = 5.4 Hz, 2H), 6.03 – 5.94 (m, 1H), 4.74 (d, *J* = 6.4 Hz, 2H), 3.57 – 3.52 (m, 2H), 2.38 (s, 3H). **^{13}C NMR (151 MHz, CDCl$_3$)** δ 152.6, 149.8, 136.3, 136.3, 134.6, 134.0, 129.5 (2C), 128.6 (2C), 128.1 (2C), 127.9, 125.9, 122.6 (2C), 97.3, 67.2, 24.0, 21.1. **HRMS** (ESI) *m/z* calcd for $C_{20}H_{19}N_2O$ [M+H]$^+$ = 303.149190; found = 303.148985.

1-(2,4-dinitrophenyl)-3-phenyl-4,7-dihydro-1H-oxepino[2,3-c]pyrazole, 1f

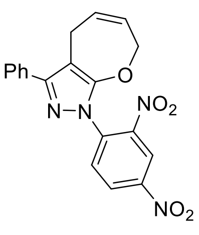

Yellow oil, yield 35%; **^{1}H NMR (600 MHz, CDCl$_3$)** δ 8.78 (d, *J* = 2.5 Hz, 1H), 8.50 (dd, J_1 = 8.9, J_2 = 2.5 Hz, 1H), 8.04 (d, *J* = 8.9 Hz, 1H), 7.65 – 7.61 (m, 2H), 7.50 – 7.40 (m, 4H), 6.17 (dt, J_1 = 10.8, J_2 = 5.4 Hz, 1H), 5.99 (dddt, J_1 = 10.0, J_2 = 8.1, J_3 = 6.5, J_4 = 1.9 Hz, 1H), 4.66 (d, *J* = 6.5 Hz, 2H), 3.54 (dd, J_1 = 5.4, J_2 = 1.7 Hz, 2H). **^{13}C NMR (151 MHz, CDCl$_3$)** δ 154.0, 153.1, 145.3, 135.9, 134.6, 132.7, 129.5, 129.3, 128.9, 128.8 (2C), 128.4, 128.1 (2C), 127.6, 125.5, 121.1, 98.6, 67.3, 24.0. **HRMS** (ESI) *m/z* calcd for $C_{19}H_{15}N_4O_5$ [M+H]$^+$ = 379.103696; found = 379.103394.

4. Synthesis of the final spiro compounds

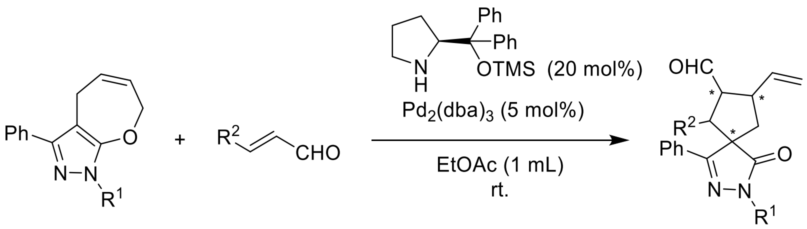

Scheme S2: Scheme of the reaction developed

4.1. General procedure for the ring contraction for products **3**

In a closed vial were added the organic catalyst 2-(diphenyl((trimethylsilyl)oxy)methyl)pyrrolidine **4** (20 mol% equiv), α,β-unsaturated aldehyde **2** (1 equiv), pyrazolone **1** (2 equiv, 0.2 mmol), $Pd_2(dba)_3$ **5** (5 mol% equiv) and EtOAc (1 mL). The reaction mixture was stirred at room temperature and checked by ^{1}H-NMR. The crude mixture was purified by flash column chromatography (*n*-hexane/EtOAc) to obtain the desired product **3**.

4.2. Modified procedure for products **3o-p**, **3r**, **3t-w**

In a closed vial were added the organic catalyst 2-(diphenyl((trimethylsilyl)oxy)methyl)pyrrolidine **4** (20 mol% equiv), α,β-unsaturated aldehyde **2** (5 equiv), pyrazolone **1** (1 equiv, 0.2 mmol), $Pd_2(dba)_3$ **5** (5 mol% equiv) and EtOAc (1 mL). The reaction mixture was stirred at room temperature and checked by ^{1}H-NMR. The crude mixture was purified by flash column chromatography (*n*-hexane/EtOAc) to obtain the desired product **3**.

4.3. Characterization of compounds **3**

1,6-dimethyl-4-oxo-3-phenyl-8-vinyl-2,3-diazaspiro[4.4]non-1-ene-7-carbaldehyde, 3a

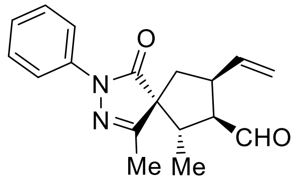

Yellow oil, yield 76%, dr 8.2:1.3:1, ee = 96% major dia. **^{1}H NMR (400 MHz, CDCl$_3$)** δ 9.78 (d, *J* = 1.3 Hz, 1H), 7.91 – 7.86 (m, 2H), 7.42 – 7.37 (m, 2H), 7.21 – 7.14 (m, 1H), 5.69 (ddd, *J* = 16.9, 9.8 Hz, 1H), 5.26 – 5.19 (m, 1H), 5.12 (dd, *J* = 10.0, 0.8 Hz, 1H), 3.97 – 3.83 (m, 1H), 3.46 (td, *J* = 10.5, 1.2 Hz, 1H), 2.83 (dq, *J* = 10.3, 6.8 Hz, 1H), 2.22 (dd, *J* = 13.1, 7.4 Hz, 1H), 2.14 (s, 3H), 1.82 (dd, *J* = 13.5, 8.5 Hz, 1H), 0.90 (d, *J* = 6.8 Hz, 3H). **^{13}C NMR (101 MHz, CDCl$_3$)** δ 202.7, 175.0, 161.1, 137.9, 137.6, 128.8 (2C), 125.0, 118.7 (2C), 117.3, 63.4, 58.6, 42.9, 40.2, 38.4, 13.2, 12.9. **HRMS** (ESI) *m/z* calcd for $C_{18}H_{21}N_2O_2$ $[M+H]^+$ = 297.1598, found = 297.1601. The ee was determined by **HPLC** analysis using Chiralpak AY-H column (hexane/*i*PrOH = 80:20, flow rate 1.0 mL/min, λ = 210 nm): t_S = 8.5, t_R = 13.2. **[α]$^{21}_D$** = -36.8° (c = 0.9, CHCl$_3$. R catalyst).

6-ethyl-1-methyl-4-oxo-3-phenyl-8-vinyl-2,3-diazaspiro[4.4]non-1-ene-7-carbaldehyde, 3b

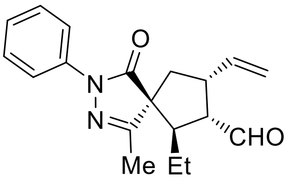

Yellow oil, yield 75%, dr 13.4:3:1, ee = >99% major diastereomer. Major dia: **^{1}H NMR (400 MHz, CDCl$_3$)** δ 9.82 (d, *J* = 1.9 Hz, 1H), 7.90 (dd, *J* = 8.7, 1.1 Hz, 2H), 7.44 – 7.37 (m, 2H), 7.19 (tt, *J* = 7.1, 1.0 Hz, 1H), 5.81 – 5.65 (m, 1H), 5.24 (dt, *J* = 17.0, 1.1 Hz, 1H), 5.14 (dd, *J* = 10.2, 0.4 Hz, 1H), 4.01 (ddd, *J* = 20.0, 10.5, 6.9 Hz, 1H), 3.36 (ddd, *J* = 10.7, 8.6, 1.8 Hz, 1H), 2.86

(dd, *J* = 15.7, 7.7 Hz, 1H), 2.20 (s, 3H), 2.07 (dd, *J* = 13.0, 6.8 Hz, 1H), 1.88 (dd, *J* = 13.0, 10.6 Hz, 1H), 1.48 – 1.31 (m, 2H), 0.76 (t, *J* = 7.5 Hz, 3H). **^{13}C NMR (101 MHz, $CDCl_3$)** δ 203.2, 174.9, 162.1, 138.0, 136.7, 128.8 (2C), 125.0, 118.8 (2C), 117.5, 62.7, 58.4, 46.3, 43.3, 39.4, 23.5, 13.1, 12.5. **HRMS** (ESI) *m/z* calcd for $C_{19}H_{23}N_2O_2$ $[M+H]^+$ = 311.1754, found = 311.1752. The ee was determined by **HPLC** analysis using Chiralpak AY-H column (hexane/*i*PrOH = 95:5, flow rate 1.0 mL/min, λ = 210 nm): t_S = 21.1, t_R = 14.5. **$[\alpha]^{21}_D$** = +23.6° (c = 0.3, $CHCl_3$).

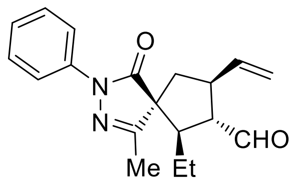

Minor dia: **^{1}H NMR (400 MHz, $CDCl_3$)** δ 9.69 (d, *J* = 3.5 Hz, 1H), 7.95 – 7.91 (m, 2H), 7.44 – 7.39 (m, 2H), 7.23 – 7.17 (m, 1H), 5.94 (ddd, *J* = 17.0, 10.1, 8.8 Hz, 1H), 5.20 (t, *J* = 13.7 Hz, 2H), 3.51 – 3.39 (m, 1H), 3.03 – 2.88 (m, 2H), 2.25 (s, 3H), 2.24 – 2.15 (m, 2H), 1.46 – 1.34 (m, 1H), 1.34 – 1.22 (m, 1H), 0.76 (t, *J* = 7.5 Hz, 3H). **^{13}C NMR (101 MHz, $CDCl_3$)** δ 202.3, 175.0, 161.4, 138.0, 136.3, 128.9 (2C), 125.1, 118.7 (2C), 117.6, 63.5, 60.3, 50.9, 45.3, 40.5, 23.7, 16.9, 12.6.

1-methyl-4-oxo-3-phenyl-6-propyl-8-vinyl-2,3-diazaspiro[4.4]non-1-ene-7-carbaldehyde, 3c

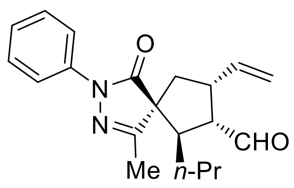

Orange oil, yield 80%, dr 20:3:1, ee = 99%. Major dia: **^{1}H NMR (400 MHz, $CDCl_3$)** δ 9.80 (d, *J* = 1.9 Hz, 1H), 7.89 (dd, *J* = 8.7, 1.1 Hz, 2H), 7.43 – 7.38 (m, 2H), 7.22 – 7.15 (m, 1H), 5.72 (ddd, *J* = 17.0, 9.9, 9.4 Hz, 1H), 5.27 – 5.19 (m, 1H), 5.13 (ddd, *J* = 10.1, 1.3, 0.7 Hz, 1H), 4.01 (ddd, *J* = 19.8, 10.6, 6.9 Hz, 1H), 3.35 (ddd, *J* = 10.7, 8.5, 1.9 Hz, 1H), 2.93 (td, *J* = 8.4, 6.3 Hz, 1H), 2.18 (s, 3H), 2.06 (dd, *J* = 13.0, 6.8 Hz, 1H), 1.87 (dd, *J* = 13.0, 10.8 Hz, 1H), 1.41 – 1.19 (m, 2H), 1.18 – 1.01 (m, 2H), 0.79 (t, *J* = 7.2 Hz, 3H). **^{13}C NMR (101 MHz, $CDCl_3$)** δ 203.2, 174.9, 162.0, 138.0, 136.6, 128.9 (2C), 125.0, 118.8 (2C), 117.5, 62.9, 58.5, 44.5, 43.4, 39.3, 32.7, 21.3, 14.1, 13.1. **HRMS** (ESI) *m/z* calcd for $C_{20}H_{25}N_2O_2$ $[M+H]^+$ = 325.1911, found = 325.1911. The ee was determined by **HPLC** analysis using Chiralpak AY-H column (hexane/*i*PrOH = 95:5, flow rate 1.0 mL/min, λ = 210 nm): t_S = 10.3, t_R = 13.7. **$[\alpha]^{21}_D$** = +34.3° (c = 0.2, $CHCl_3$).

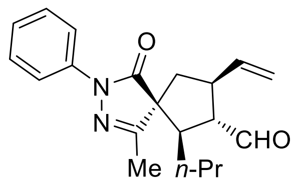

Minor dia: **^{1}H NMR (400 MHz, $CDCl_3$)** δ 9.67 (d, *J* = 3.8 Hz, 1H), 7.95 – 7.89 (m, 2H), 7.46 – 7.36 (m, 2H), 7.23 – 7.15 (m, 1H), 5.93 (ddd, *J* = 17.0, 10.1, 8.8 Hz, 1H), 5.18 (tt, *J* = 10.0, 1.1 Hz, 2H), 3.52 – 3.36 (m, 1H), 3.05 (dt, *J* = 10.7, 7.1 Hz, 1H), 2.92 (td, *J* = 10.6, 3.8 Hz, 1H), 2.24 (s, 3H), 2.21 – 2.15 (m, 2H), 1.33 – 1.00 (m, 4H), 0.78 (t, *J* = 7.1 Hz, 3H). **^{13}C NMR (101 MHz, $CDCl_3$)** δ 202.3, 174.9, 161.4, 138.0, 136.3, 128.9 (2C), 125.1, 118.7 (2C), 117.6, 63.6, 60.5, 45.4, 40.5, 32.8, 30.9, 21.2, 16.9, 14.0.

6-heptyl-1-methyl-4-oxo-3-phenyl-8-vinyl-2,3-diazaspiro[4.4]non-1-ene-7-carbaldehyde, 3d

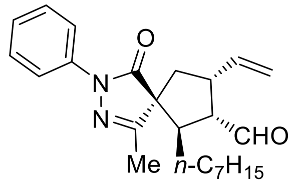

Orange oil, yield 71%, dr 6:1, ee = 95%. **^{1}H NMR (400 MHz, $CDCl_3$)** δ 9.80 (d, *J* = 1.9 Hz, 1H), 7.92 – 7.84 (m, 2H), 7.43 – 7.34 (m, 2H), 7.22 – 7.13 (m, 1H), 5.81 – 5.65 (m, 1H), 5.22 (d, *J* = 16.9 Hz, 1H), 5.13 (dd, *J* = 10.2, 0.5 Hz, 1H), 4.00 (qd, *J* = 10.4, 7.2 Hz, 1H), 3.34 (ddd, *J* = 10.6, 8.5, 1.8 Hz, 1H), 2.91 (dd, *J* = 15.0, 8.1 Hz, 1H), 2.18 (s, 3H), 2.05 (dd, *J* = 13.0, 6.8 Hz, 1H), 1.87 (dd, *J* = 13.0, 10.8 Hz, 1H), 1.41 – 0.98 (m, 12H), 0.81 (t, *J* = 7.0 Hz, 3H). **^{13}C NMR (101 MHz, $CDCl_3$)** δ 203.2, 174.9, 162.1, 138.0, 136.6, 128.8 (2C), 125.0, 118.8 (2C), 117.5, 62.8, 58.6, 44.7, 43.4, 39.4, 31.6, 30.5, 29.5, 28.9, 27.9, 22.5, 14.0, 13.1. **HRMS** (ESI) *m/z* calcd for $C_{24}H_{33}N_2O_2$ $[M+H]^+$ = 381.2537, found = 381.2535. The ee was determined by **HPLC** analysis using

Chiralpak AY-H column (hexane/*i*PrOH = 95:5, flow rate 1.0 mL/min, λ = 210 nm): t_S = 7.2, t_R = 10.0. **[α]$^{21}_{D}$** major = -16.2° (c = 0.8, $CHCl_3$) (*R* catalyst). **[α]$^{21}_{D}$** minor = +13.1° (c = 0.4, $CHCl_3$).

1-methyl-4-oxo-3,6-diphenyl-8-vinyl-2,3-diazaspiro[4.4]non-1-ene-7-carbaldehyde, 3e

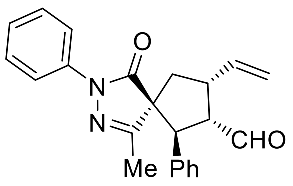

Orange oil, yield 79%, dr 7.3:2:1, ee = >99%. **^{1}H NMR (400 MHz, $CDCl_3$)** δ 9.77 (d, *J* = 1.5 Hz, 1H), 7.55 – 7.49 (m, 2H), 7.32 – 7.26 (m, 2H), 7.22 – 7.08 (m, 6H), 5.81 (dt, *J* = 16.9, 9.7 Hz, 1H), 5.34 – 5.26 (m, 1H), 5.18 (dd, *J* = 10.0, 0.7 Hz, 1H), 4.27 (td, *J* = 10.6, 1.5 Hz, 1H), 4.12 (dd, *J* = 17.8, 8.8 Hz, 1H), 4.04 (d, *J* = 10.7 Hz, 1H), 2.33 (dd, *J* = 13.4, 7.5 Hz, 1H), 2.28 (s, 3H), 1.98 (dd, *J* = 13.4, 8.5 Hz, 1H). **^{13}C NMR (101 MHz, $CDCl_3$)** δ 201.9, 174.6, 160.2, 137.4, 137.2, 135.1, 128.6 (2C), 128.5 (2C), 127.9, 127.6 (2C), 125.2, 119.3 (2C), 117.7, 64.5, 56.6, 50.7, 43.0, 38.6, 13.3. **HRMS** (ESI) *m/z* calcd for $C_{23}H_{23}N_2O_2$ $[M+H]^+$ = 359.1754, found = 359.1755. The ee was determined by **HPLC** analysis using Chiralpak AY-H column (hexane/*i*PrOH = 95:5, flow rate 1.0 mL/min, λ = 210 nm): t_S = 17.2, t_R = 28.5. **[α]$^{21}_{D}$** = +8.9° (c = 0.8, $CHCl_3$).

6-(2-methoxyphenyl)-1-methyl-4-oxo-3-phenyl-8-vinyl-2,3-diazaspiro[4.4]non-1-ene-7-carbaldehyde, 3f

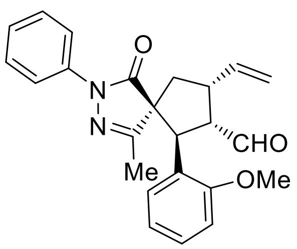

Orange oil, yield 70%, dr 3:2:1, ee = 98%. Major dia: **^{1}H NMR (400 MHz, $CDCl_3$)** δ 9.77 (d, J = 1.5 Hz, 1H), 7.61 – 7.57 (m, 2H), 7.34 – 7.27 (m, 2H), 7.15 – 7.05 (m, 2H), 6.80 (d, J = 7.8 Hz, 1H), 6.77 (d, J = 2.4 Hz, 1H), 6.67 (ddd, J = 8.3, 2.5, 0.8 Hz, 1H), 5.79 (dt, J = 16.9, 9.8 Hz, 1H), 5.29 (dt, J = 16.9, 1.1 Hz, 1H), 5.17 (dd, J = 10.0, 0.8 Hz, 1H), 4.25 (td, J = 10.7, 1.5 Hz, 1H), 4.16 – 4.05 (m, 1H), 4.00 (d, J = 10.8 Hz, 1H), 3.67 (s, 3H), 2.37 – 2.28 (m, 1H), 2.26 (s, 3H), 1.97 (dd, J = 13.4, 8.6 Hz, 1H). **^{13}C NMR (101 MHz, $CDCl_3$)** δ 201.9, 174.6, 160.2, 159.6, 137.5, 137.3, 136.8, 129.5, 128.7 (2C), 125.1, 120.0, 119.1 (2C), 117.7, 113.4, 113.3, 64.4, 56.7, 55.1, 50.7, 42.9, 38.7, 13.3. **HRMS** (ESI) *m/z* calcd for $C_{24}H_{25}N_2O_3$ $[M+H]^+$ = 389.1860, found = 389.1863. The ee was determined by **HPLC** analysis using Chiralpak AY-H column (hexane/*i*PrOH = 90:10, flow rate 1.0 mL/min, λ = 210 nm): t_S = 17.9, t_R = 8.9. **[α]$^{21}_{D}$** = +12.1° (c = 0.7, $CHCl_3$).

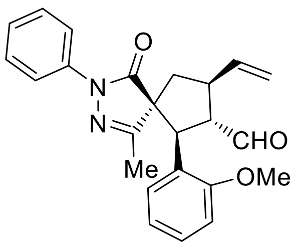

Minor dia: **^{1}H NMR (400 MHz, $CDCl_3$)** δ 9.69 (d, *J* = 2.4 Hz, 1H), 7.53 – 7.46 (m, 2H), 7.28 (dd, *J* = 5.7, 3.6 Hz, 2H), 7.13 – 7.09 (m, 2H), 6.78 (d, *J* = 7.7 Hz, 1H), 6.76 – 6.73 (m, 1H), 6.69 (ddd, *J* = 8.3, 2.5, 0.8 Hz, 1H), 6.09 (ddd, *J* = 17.1, 10.1, 8.4 Hz, 1H), 5.19 (dt, *J* = 17.0, 1.1 Hz, 1H), 5.14 (dd, *J* = 10.1, 0.7 Hz, 1H), 4.04 (ddd, *J* = 12.5, 10.4, 2.3 Hz, 1H), 3.69 (s, 3H), 3.64 (d, *J* = 12.4 Hz, 1H), 3.23 – 3.04 (m, 1H), 2.42 – 2.36 (m, 1H), 2.30 (s, 3H), 2.13 (dd, *J* = 14.1, 8.1 Hz, 1H). **^{13}C NMR (101 MHz, $CDCl_3$)** δ 201.6, 175.0, 160.3, 159.6, 138.6, 137.4, 135.2, 129.6, 128.6 (2C), 125.1, 120.0, 119.2 (2C), 116.7, 113.7, 113.4, 63.9, 58.3, 55.2, 54.1, 45.1, 37.0, 13.4.

1-methyl-4-oxo-3-phenyl-6-(*p*-tolyl)-8-vinyl-2,3-diazaspiro[4.4]non-1-ene-7-carbaldehyde, 3g

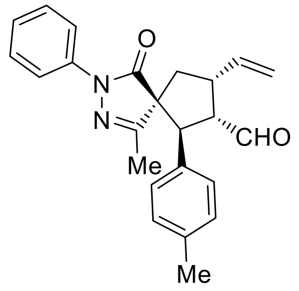

Yellow oil, yield 76%, dr 5:3:1, ee = 99%. Major dia: **^{1}H NMR (400 MHz, CDCl$_3$)** δ 9.69 (d, *J* = 1.6 Hz, 1H), 7.48 (dd, *J* = 8.7, 1.1 Hz, 2H), 7.25 – 7.20 (m, 2H), 7.07 – 6.99 (m, 3H), 6.91 (d, *J* = 8.0 Hz, 2H), 5.72 (dt, *J* = 16.9, 9.7 Hz, 1H), 5.21 (dt, 1H), 5.10 (dd, *J* = 10.1, 0.8 Hz, 1H), 4.17 (td, *J* = 10.7, 1.5 Hz, 1H), 4.08 – 3.97 (m, 1H), 3.93 (d, *J* = 10.8 Hz, 1H), 2.24 (dd, *J* = 13.4, 7.5 Hz, 1H), 2.19 (s, 3H), 2.13 (s, 3H), 1.89 (dd, *J* = 13.4, 8.5 Hz, 1H). **^{13}C NMR (101 MHz, CDCl$_3$)** δ 202.1, 174.7, 160.3, 137.6, 137.5, 137.3, 132.1, 129.3 (2C), 128.6 (2C), 127.5 (2C), 125.1, 119.3 (2C), 117.6, 64.5, 56.7, 50.5, 43.0, 38.7, 21.0, 13.3. **HRMS** (ESI) *m/z* calcd for $C_{24}H_{25}N_2O_2$ $[M+H]^+$ = 373.1911, found = 373.1915. The ee was determined by **HPLC** analysis using Chiralpak OZ-H column (hexane/*i*PrOH = 98:2, flow rate 1.0 mL/min, λ = 210 nm): t_S = 22.3, t_R = 24.2. **$[\alpha]^{21}_D$** = +19.1° (c = 0.17, CHCl$_3$)

6-(4-bromophenyl)-1-methyl-4-oxo-3-phenyl-8-vinyl-2,3-diazaspiro[4.4]non-1-ene-7-carbaldehyde, 3h

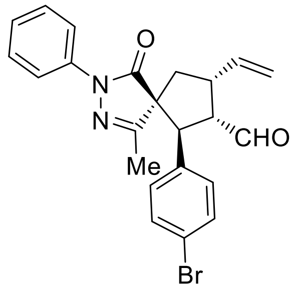

Orange oil, yield 72%, dr 5:3:1, ee = 98%. Major dia: **^{1}H NMR (400 MHz, CDCl$_3$)** δ 9.76 (d, *J* = 1.3 Hz, 1H), 7.57 – 7.53 (m, 2H), 7.34 – 7.29 (m, 4H), 7.17 – 7.11 (m,1H), 7.11 – 7.08 (m, 2H), 5.77 (dt, *J* = 16.8, 9.7 Hz, 1H), 5.30 (dt, *J* = 16.9, 1.1 Hz, 1H), 5.19 (dd, *J* = 10.1, 0.8 Hz, 1H), 4.22 (td, *J* = 10.7, 1.3 Hz, 1H), 4.16 – 4.06 (m, 1H), 3.98 (d, *J* = 10.6 Hz, 1H), 2.35 – 2.29 (m, 1H), 2.25 (s, 3H), 2.01 – 1.90 (m, 1H). **^{13}C NMR (101 MHz, CDCl$_3$)** δ 201.6, 174.4, 160.0, 137.3, 137.0, 134.4, 131.7 (2C), 129.4 (2C), 128.7 (2C), 125.4, 122.0, 119.2 (2C), 118.0, 64.3, 56.8, 49.9, 42.8, 38.7, 13.3. **HRMS** (ESI) *m/z* calcd for $C_{23}H_{22}BrN_2O_2$ $[M+H]^+$ = 437.0859, found = 437.0849. The ee was determined by **HPLC** analysis using Chiralpak OZ-H column (hexane/*i*PrOH = 93:7, flow rate 1.0 mL/min, λ = 210 nm): t_S = 10.0, t_R = 13.4. **$[\alpha]^{21}_D$** = +41.3° (c = 0.2, CHCl$_3$).

6-(4-chlorophenyl)-1-methyl-4-oxo-3-phenyl-8-vinyl-2,3-diazaspiro[4.4]non-1-ene-7-carbaldehyde, 3i

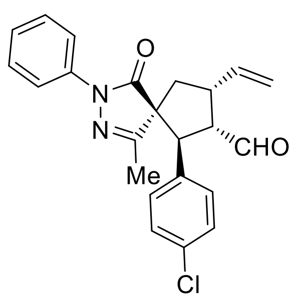

Orange oil, yield 71%, dr 3:3:1, ee = >99%. Major dia: **^{1}H NMR (400 MHz, CDCl$_3$)** δ 9.76 (d, *J* = 1.3 Hz, 1H), 7.58 – 7.53 (m, 2H), 7.34 – 7.29 (m, 2H), 7.18 – 7.10 (m, 5H), 5.77 (dt, *J* = 16.9, 9.7 Hz, 1H), 5.30 (dt, *J* = 16.9, 1.1 Hz, 1H), 5.19 (dd, *J* = 10.0, 0.7 Hz, 1H), 4.22 (td, *J* = 10.6, 1.3 Hz, 1H), 4.17 – 4.06 (m, 1H), 3.99 (d, *J* = 10.6 Hz, 1H), 2.36 – 2.29 (m, 1H), 2.26 (s, 3H), 1.96 (dd, *J* = 13.5, 8.4 Hz, 1H). **^{13}C NMR (101 MHz, CDCl$_3$)** δ 201.6, 174.4, 160.0, 137.3, 137.0, 133.9, 133.8, 129.0 (2C), 128.8 (2C), 128.7 (2C), 125.3, 119.2 (2C), 117.9, 64.3, 56.8, 49.9, 42.8, 38.7, 13.2. **HRMS** (ESI) *m/z* calcd for $C_{23}H_{22}ClN_2O_2$ $[M+H]^+$ = 393.1364, found = 393.1363. The ee was determined by **HPLC** analysis using Chiralpak AY-H column (hexane/*i*PrOH = 93:7, flow rate 1.0 mL/min, λ = 210 nm): t_S = 12.1, t_R = 22.6. **$[\alpha]^{21}_D$** = +37.5° (c = 0.2, CHCl$_3$).

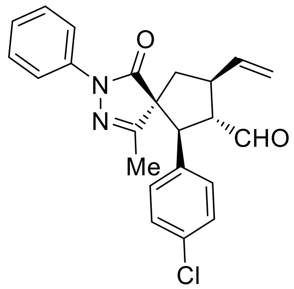

Minor dia, ee = 90%. **^{1}H NMR (400 MHz, $CDCl_3$)** δ 9.69 (d, *J* = 2.3 Hz, 1H), 7.47 – 7.43 (m, 2H), 7.31 – 7.26 (m, 2H), 7.20 – 7.11 (m, 5H), 6.09 (ddd, *J* = 17.1, 10.1, 8.4 Hz, 1H), 5.21 (dt, *J* = 17.1, 1.1 Hz, 1H), 5.16 (dd, *J* = 10.1, 0.6 Hz, 1H), 4.00 (ddd, *J* = 12.6, 10.5, 2.3 Hz, 1H), 3.65 (d, *J* = 12.3 Hz, 1H), 3.17 – 3.04 (m, 1H), 2.39 (dd, *J* = 14.2, 9.8 Hz, 1H), 2.30 (s, 3H), 2.20 – 2.10 (m, 1H). **^{13}C NMR (101 MHz, $CDCl_3$)** δ 201.3, 174.8, 160.2, 138.3, 137.1, 134.1, 132.3, 129.0 (2C), 128.7 (2C), 128.7 (2C), 125.3, 119.2 (2C), 117.0, 63.7, 58.2, 53.0, 45.4, 37.0, 13.4.

1-methyl-6-(3-nitrophenyl)-4-oxo-3-phenyl-8-vinyl-2,3-diazaspiro[4.4]non-1-ene-7-carbaldehyde, 3j

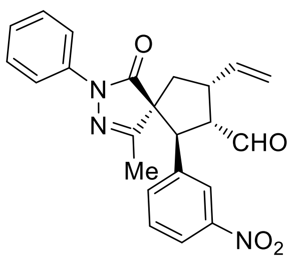

Orange oil, yield 86%, dr 2:1, ee = >99%. Major dia: **^{1}H NMR (400 MHz, $CDCl_3$)** δ 9.79 (d, *J* = 0.6 Hz, 1H), 8.11 (t, *J* = 1.7 Hz, 1H), 8.01 (dd, *J* = 8.2, 1.3 Hz, 1H), 7.58 – 7.51 (m, 3H), 7.37 (t, *J* = 8.0 Hz, 1H), 7.29 (t, *J* = 8.0 Hz, 2H), 7.12 (t, *J* = 7.4 Hz, 1H), 5.77 (dt, *J* = 16.9, 9.7 Hz, 1H), 5.34 (d, *J* = 16.8 Hz, 1H), 5.22 (d, *J* = 10.2 Hz, 1H), 4.32 (t, *J* = 10.6 Hz, 1H), 4.17 (dt, *J* = 15.6, 7.7 Hz, 1H), 4.11 (t, *J* = 7.3 Hz, 1H), 2.38 (dd, *J* = 13.5, 7.6 Hz, 1H), 2.30 (s, 3H), 2.06 – 1.95 (m, 1H). **^{13}C NMR (101 MHz, $CDCl_3$)** δ 201.1, 174.0, 159.7, 148.2, 137.7, 137.1, 136.7, 133.8, 129.6, 128.8 (2C), 125.4, 123.0, 122.8, 118.9 (2C), 118.3, 64.2, 56.8, 49.7, 42.7, 38.6, 13.3. **HRMS** (ESI) *m/z* calcd for $C_{23}H_{22}N_3O_4$ $[M+H]^+$ = 404.1605, found = 404.1604. The ee was determined by **HPLC** analysis using Chiralpak AY-H column (hexane/*i*PrOH = 80:20, flow rate 1.0 mL/min, λ = 210 nm): t_S = 16.5, t_R = 38.2. **$[\alpha]^{21}_D$ major dia** = -349.4° (c = 0.2, $CHCl_3$) (*R* catalyst). **$[\alpha]^{21}_D$ major dia** = +37.0° (c = 0.2, $CHCl_3$).

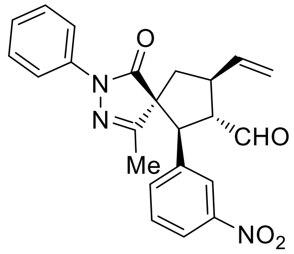

Minor dia: **^{1}H NMR (400 MHz, $CDCl_3$)** δ = 9.76 (d, *J* = 1.8 Hz, 1H), 8.06 – 7.98 (m, 2H), 7.54 (d, *J* = 7.7 Hz, 1H), 7.45 (d, *J* = 7.6 Hz, 1H), 7.39 (t, *J* = 7.9 Hz, 1H), 7.29 – 7.21 (m, 3H), 7.09 (t, *J* = 7.5 Hz, 1H), 6.15 (ddd, *J* = 17.1, 10.0, 8.5 Hz, 1H), 5.23 (dd, *J* = 16.3, 13.7 Hz, 2H), 4.11 – 4.05 (m, 1H), 3.78 (d, *J* = 12.1 Hz, 1H), 3.18 – 3.05 (m, 1H), 2.43 (dd, *J* = 14.3, 9.6 Hz, 1H), 2.34 (s, 3H), 2.25 – 2.14 (m, 1H). **^{13}C NMR (101 MHz, $CDCl_3$)** δ = 200.8, 171.5, 160.0, 148.1, 138.1, 137.0, 136.4, 133.7, 129.6, 128.7 (2C), 125.4, 123.2, 122.7, 118.8 (2C), 117.5, 63.6, 58.1, 52.5, 45.8, 37.1, 14.1.

6-(3-methoxyphenyl)-1-methyl-4-oxo-3-phenyl-8-vinyl-2,3-diazaspiro[4.4]non-1-ene-7-carbaldehyde, 3k

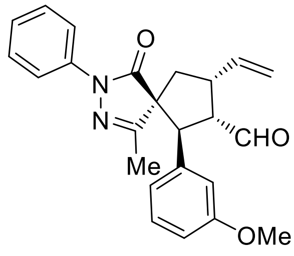

Yellow oil, yield 77%, dr 6:2:1, ee = 97%. Major dia: **^{1}H NMR (400 MHz, $CDCl_3$)** δ 9.77 (d, *J* = 1.5 Hz, 1H), 7.60 – 7.56 (m, 2H), 7.33 – 7.27 (m, 2H), 7.15 – 7.06 (m, 2H), 6.83 – 6.78 (m, 1H), 6.78 – 6.75 (m, 1H), 6.67 (ddd, *J* = 8.3, 2.6, 0.9 Hz, 1H), 5.79 (dt, *J* = 16.9, 9.8 Hz, 1H), 5.33 – 5.25 (m, 1H), 5.17 (ddd, *J* = 10.1, 1.3, 0.5 Hz, 1H), 4.25 (td, *J* = 10.7, 1.5 Hz, 1H), 4.12 (dd, *J* = 12.7, 5.5 Hz, 1H), 4.00 (d, *J* = 10.8 Hz, 1H), 3.67 (s, 3H), 2.37 – 2.28 (m, 1H), 2.26 (s, 3H), 2.01 – 1.92 (m, 1H). **^{13}C NMR (101 MHz, $CDCl_3$)** δ 201.9, 174.6, 160.2, 159.6, 137.5, 137.2, 136.7, 129.5, 128.7 (2C), 125.1, 120.0, 119.1 (2C), 117.7, 113.4, 113.3, 64.4, 56.7, 55.1, 50.7, 42.9, 38.7, 22.6. **HRMS** (ESI) *m/z* calcd for $C_{24}H_{25}N_2O_3$ $[M+H]^+$ = 389.1860, found = 389.1860. The ee was determined by **HPLC** analysis using Chiralpak AY-H column (hexane/*i*PrOH = 95:5, flow rate 1.0 mL/min, λ = 210 nm): t_S = 36.6, t_R = 28.7. **$[\alpha]^{21}_D$** = +158.1° (c = 0.1, $CHCl_3$).

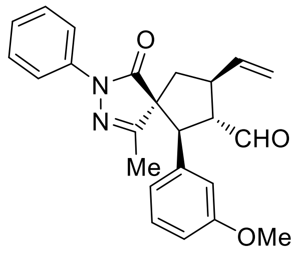

Minor dia: **^{1}H NMR (400 MHz, $CDCl_3$)** δ 9.69 (d, *J* = 2.4 Hz, 1H), 7.51 – 7.46 (m, 2H), 7.31 – 7.27 (m, 1H), 7.26 – 7.23 (m, 1H), 7.14 – 7.08 (m, 2H), 6.78 (dd, *J* = 7.7, 0.6 Hz, 1H), 6.76 – 6.73 (m, 1H), 6.69 (ddd, *J* = 8.3, 2.5, 0.8 Hz, 1H), 6.09 (ddd, *J* = 17.1, 10.1, 8.4 Hz, 1H), 5.23 – 5.16 (m, 1H), 5.14 (dd, *J* = 10.1, 0.7 Hz, 1H), 4.04 (ddd, *J* = 12.5, 10.4, 2.3 Hz, 1H), 3.69 (s, 3H), 3.64 (d, *J* = 12.4 Hz, 1H), 3.22 – 3.08 (m, 1H), 2.43 – 2.36 (m, 1H), 2.30 (s, 3H), 2.13 (dd, *J* = 14.1, 8.1 Hz, 1H). **^{13}C NMR (101 MHz, $CDCl_3$)** δ 201.6, 175.0, 160.3, 159.6, 138.6, 137.3, 135.2, 129.6, 128.6 (2C), 125.1, 119.9, 119.2 (2C), 116.7, 113.7, 113.4, 63.8, 58.3, 55.1, 54.0, 45.1, 31.6, 22.6.

1-ethyl-4-oxo-3,6-diphenyl-8-vinyl-2,3-diazaspiro[4.4]non-1-ene-7-carbaldehyde, 3m

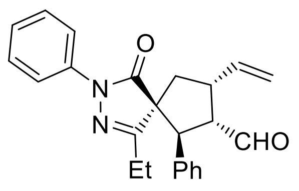

Orange oil, yield 96%, dr 3:1:1, ee = >99%. **^{1}H NMR (400 MHz, $CDCl_3$)** δ 9.77 (d, *J* = 1.5 Hz, 1H), 7.58 – 7.52 (m, 2H), 7.34 – 7.26 (m, 2H), 7.21 – 7.07 (m, 6H), 5.79 (ddd, *J* = 16.9, 9.7 Hz, 1H), 5.33 – 5.25 (m, 1H), 5.17 (dd, *J* = 10.0, 0.8 Hz, 1H), 4.30 – 4.03 (m, 3H), 2.70 (dq, *J* = 17.5, 7.4 Hz, 1H), 2.54 (dq, *J* = 17.5, 7.3 Hz, 1H), 2.32 (dd, *J* = 13.4, 7.5 Hz, 1H), 1.98 (dd, *J* = 13.4, 8.5 Hz, 1H), 1.35 (t, *J* = 7.3 Hz, 3H). **^{13}C NMR (101 MHz, $CDCl_3$)** δ 202.2, 175.0, 164.0, 137.7, 137.4, 135.4, 128.8, 128.7, 128.0, 127.8, 125.3, 119.4, 117.8, 64.8, 56.8, 51.0, 43.2, 38.8, 20.6, 9.6. **HRMS** (ESI) *m/z* calcd for $C_{24}H_{24}N_2O_2$ $[M+H]^+$ = 373.1838, found = 373.1911. The ee was determined by **HPLC** analysis using Chiralpak AY-H column (hexane/*i*PrOH = 90:10, flow rate 1.0 mL/min, λ = 230 nm): t_S = 9.6, t_R = 16.0. **$[\alpha]^{21}_D$** = +128.1° (c = 0.2, $CHCl_3$).

6-methyl-4-oxo-1,3-diphenyl-8-vinyl-2,3-diazaspiro[4.4]non-1-ene-7-carbaldehyde, 3n

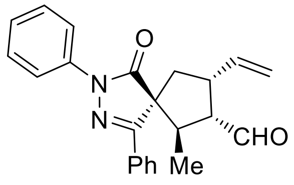

Transparent oil, yield 96%, dr 5:1, ee = 98%. **^{1}H NMR (400 MHz, $CDCl_3$)** δ 9.82 (d, *J* = 1.3 Hz, 1H), 8.03 – 7.98 (m, 2H), 7.93 – 7.87 (m, 2H), 7.50 – 7.48 (m, 2H), 7.47 – 7.42 (m, *J* = 11.4, 4.7 Hz, 3H), 7.23 (t, *J* = 7.4 Hz, 1H), 5.77 (dt, *J* = 16.9, 9.8 Hz, 1H), 5.28 – 5.22 (m, 1H), 5.14 (dd, *J* = 10.0, 0.8 Hz, 1H), 4.09 – 3.95 (m, 1H), 3.60 (td, *J* = 10.6, 1.2 Hz, 1H), 3.34 – 3.27 (m, 1H), 2.41 (dd, *J* = 13.8, 8.0 Hz, 1H), 2.31 (dd, *J* = 13.9, 8.3 Hz, 1H), 0.95 (d, *J* = 6.7 Hz, 3H). **^{13}C NMR (101 MHz, $CDCl_3$)** δ 202.7, 175.6, 158.3, 137.8, 130.4, 130.3, 129.0 (2C), 128.9 (2C), 126.8 (2C), 126.4, 125.4, 119.0 (2C), 117.3, 63.7, 58.4, 43.1, 41.3, 39.4, 13.5. **HRMS** (ESI) *m/z* calcd for $C_{23}H_{23}N_2O_2$ $[M+H]^+$ = 359.1754, found = 359.1748. The ee was determined by **HPLC** analysis using Chiralpak AY-H column (hexane/*i*PrOH = 97:3, flow rate 1.0 mL/min, λ = 210 nm): t_S = 12.3, t_R = 19.1. **$[\alpha]^{21}_D$** = +28.6° (c = 1.0, $CHCl_3$).

6-ethyl-4-oxo-1,3-diphenyl-8-vinyl-2,3-diazaspiro[4.4]non-1-ene-7-carbaldehyde, 3o

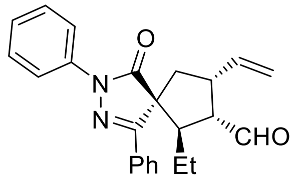

Transparent oil, yield 62%, 20:1, ee = 99%. **^{1}H NMR (600 MHz, $CDCl_3$)** δ 9.88 (d, *J* = 1.8 Hz, 1H), 8.02 – 7.98 (m, 4H), 7.51 (q, *J* = 6.4 Hz, 3H), 7.45 (t, *J* = 7.7 Hz, 2H), 7.23 (t, *J* = 7.4 Hz, 1H), 5.78 (dt, J_1 = 16.8, J_2 = 9.6 Hz, 1H), 5.25 (d, *J* = 17.0 Hz, 1H), 5.14 (d, *J* = 10.1 Hz, 1H), 4.17 (qd, J_1 = 10.5, J_2 =7.3 Hz, 1H), 3.47 (ddd, J_1 = 10.8, J_2 = 8.2, J_3 = 1.8 Hz, 1H), 3.45 – 3.37 (m, 1H), 2.38 (dd, J_1 = 13.4, J_2 = 10.8 Hz, 1H), 2.23 (dd, J_1 = 13.4, J_2 = 7.0 Hz, 1H), 1.50 – 1.36 (m, 2H), 0.70 (t, *J* = 7.5 Hz, 3H). **^{13}C NMR (151 MHz, $CDCl_3$)** δ = 203.7, 175.5, 159.1, 138.1, 137.0, 130.5, 130.4, 129.1 (2C), 129.1 (2C), 127.0 (2C), 125.5, 119.2 (2C), 117. 7, 63.2, 58.6, 47.5, 43.6, 40.7, 23.9, 12.8. **HRMS** (ESI)

m/z calcd for $C_{24}H_{25}N_2O_2$ $[M+H]^+$ = 373.1911; found = 373.1910. The ee was determined by **HPLC** analysis using Chiralpak ODH column (98/2 heptane/*i*-PrOH, flow rate 0.5 ml/min; λ = 322 nm, t_R = 13.6 min., t_S = 16.4 min). $[\alpha]^{25}_D$ = +16.3° (c = 1.4, $CHCl_3$).

6-butyl-4-oxo-1,3-diphenyl-8-vinyl-2,3-diazaspiro[4.4]non-1-ene-7-carbaldehyde, 3p

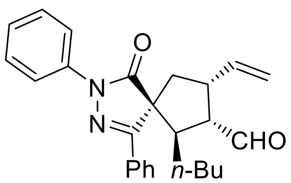

Transparent oil, yield 73%, dr 9:1, ee = 98% major dia/94% minor dia. **^{1}H NMR (600 MHz, $CDCl_3$)** δ 9.88 (s, 1H), 8.03-7.97 (m, 4H), 7.51 (d, *J* = 7.7 Hz, 3H), 7.47 – 7.43 (m, 2H), 7.24 (t, *J* = 7.4 Hz, 1H), 5.83 – 5.73 (m, 1H), 5.25 (d, *J* = 16.8 Hz, 1H), 5.14 (d, *J* = 10.1 Hz, 1H), 4.18 (qd, J_1 = 10.3, J_2 = 7.1 Hz, 1H), 3.48 (dd, J_1 = 9.0, J_2 = 5.6 Hz, 2H), 2.38 (dd, J_1 = 13.4, J_2 = 10.9 Hz, 1H), 2.22 (dd, J_1 = 13.4, J_2 = 7.0 Hz, 1H), 1.45 – 1.31 (m, 2H), 1.17 – 0.97 (m, 4H), 0.70 (t, *J* = 7.3 Hz, 3H). **^{13}C NMR (151 MHz, $CDCl_3$)** δ 203.7, 175.6, 159.0, 138.1, 137.0, 130.5, 130.4, 129.1 (2C), 129.1 (2C), 127.0 (2C), 125.6, 119.3 (2C), 117.7, 63.3, 58.9, 45.7, 43.7, 40.7, 30.6, 30.3, 22.7, 13.9. **HRMS** (ESI) *m/z* calcd for $C_{26}H_{27}N_2O$ $[M+H]^+$ = 383.2118; found = 383.2117. The ee was determined by **HPLC** analysis using Chiralpak IA column (95/5 heptane/*i*-PrOH, flow rate 1.0 ml/min; λ = 190 nm, major dia: t_R = 7.2 min., t_S = 14.9 min, minor dia: t_S = 9.2 min., t_R = 10.2 min). $[\alpha]^{25}_D$ = +37.2° (c = 0.7, $CHCl_3$).

4-oxo-1,3,6-triphenyl-8-vinyl-2,3-diazaspiro[4.4]non-1-ene-7-carbaldehyde, 3q

Orange oil, yield 98%, dr 1:1, ee = 99% dia 1, ee = 66% dia 2.

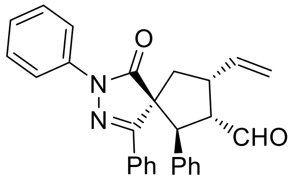

Dia 1: **^{1}H NMR (400 MHz, $CDCl_3$)** δ 9.82 (d, *J* = 1.3 Hz, 1H), 7.99 (dd, *J* = 6.6, 3.1 Hz, 2H), 7.61 – 7.57 (m, 2H), 7.55 – 7.52 (m, 3H), 7.35 – 7.29 (m, 3H), 7.17 – 7.07 (m, 7H), 5.92 (dt, *J* = 16.8, 9.8 Hz, 1H), 5.33 (d, *J* = 16.6 Hz, 1H), 5.21 (d, *J* = 10.1 Hz, 1H), 4.55 (d, *J* = 10.7 Hz, 1H), 4.45 (t, *J* = 10.5 Hz, 1H), 4.28 – 4.17 (m, 1H), 2.54 (dd, *J* = 8.0, 2.1 Hz, 2H). **^{13}C NMR (101 MHz, $CDCl_3$)** δ 202.0, 175.2, 157.3, 137.6, 137.3, 134.9, 130.6, 130.5, 129.1 (2C), 128.7 (2C), 128.3 (2C), 127.9, 127.7 (2C), 126.8 (2C), 125.5, 119.6 (2C), 117.7, 64.7, 56.0, 51.7, 43.2, 39.5. **HRMS** (ESI) *m/z* calcd for $C_{28}H_{25}N_2O_2$ $[M+H]^+$ = 421.1911, found = 421.1912. The ee was determined by **HPLC** analysis using Chiralpak AY-H column (heptane/*i*PrOH = 98:2, flow rate 0.5 mL/min, λ = 254 nm): t_S = 39.6, t_R = 17.9. $[\alpha]^{21}_D$ = +8.0° (c = 0.6, $CHCl_3$).

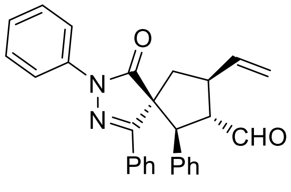

Dia 2: **^{1}H NMR (400 MHz, $CDCl_3$)** δ 9.72 (d, *J* = 2.5 Hz, 1H), 8.01 (ddd, *J* = 7.8, 5.2, 2.0 Hz, 2H), 7.62 – 7.54 (m, 3H), 7.47 (dd, *J* = 8.7, 1.1 Hz, 2H), 7.35 – 7.28 (m, 2H), 7.18 – 7.01 (m, 5H), 6.16 – 6.04 (m, 1H), 5.31 (d, *J* = 17.1 Hz, 1H), 5.21 (d, *J* = 10.1 Hz, 1H), 4.14 (dt, *J* = 22.2, 7.4 Hz, 1H), 3.52 – 3.40 (m, 1H), 2.88 (dd, *J* = 14.5, 9.6 Hz, 1H), 2.54 (dd, *J* = 8.0, 1.9 Hz, 1H), 2.41 (dd, *J* = 14.5, 9.5 Hz, 1H). **^{13}C NMR (101 MHz, $CDCl_3$)** δ 201.6, 175.7, 157.7, 138.1, 137.1, 133.3, 130.6, 130.5, 130.0, 129.3 (2C), 128.6 (2C), 128.3 (2C), 128.1, 127.6 (2C), 126.3 (2C), 119.6 (2C), 117.1, 63.1, 57.5, 55.6, 45.7, 38.2. The ee was determined by **HPLC** analysis using Chiralpak AY-H column (heptane/*i*PrOH = 98:2, flow rate 0.5 mL/min, λ = 254 nm): t_S = 34.7, t_R = 25.2. $[\alpha]^{21}_D$ = +8.0° (c = 0.6, $CHCl_3$).

3-(*tert*-butyl)-6-ethyl-4-oxo-1-phenyl-8-vinyl-2,3-diazaspiro[4.4]non-1-ene-7-carbaldehyde, 3r

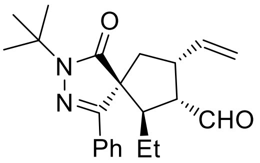

Transparent oil, yield 54%, dr 20:1, ee = 93%. **^{1}H NMR (600 MHz, $CDCl_3$)** δ 9.84 (d, *J* = 2.0 Hz, 1H), 7.91 – 7.82 (m, 2H), 7.45-7.41 (m, 3H), 5.75 (dt, J_1 = 16.9, J_2 = 9.4 Hz, 1H), 5.21 (dt, *J* = 16.9, J_2 = 1.2 Hz, 2H), 5.14 – 5.06 (m, 1H), 4.08 (qd, J_1 = 10.5, J_2 = 7.4 Hz, 1H), 3.39 (ddd, J_1 = 10.8, J_2 = 8.3, J_3 = 2.0 Hz, 1H), 3.26 (td, J_1 = 8.2, J_2 = 6.4 Hz, 1H), 2.26 (dd, J_1 = 13.4, J_2 = 10.7 Hz, 1H), 2.09 (dd, J_1 = 13.3, J_2 = 7.1 Hz, 1H), 1.57 (s, 9H), 1.46 – 1.31 (m, 2H), 0.68 (t, *J* = 7.5 Hz, 3H). **^{13}C NMR (151 MHz, $CDCl_3$)** δ 204.0, 177.3, 156.3, 137.4, 131.0, 129.7, 129.0 (2C), 126.5 (2C), 117.3, 62.7, 58.6, 58.0, 47.2, 43.4, 40.3, 28.4 (3C), 23.7, 12.7. **HRMS** (ESI) *m/z* calcd for $C_{22}H_{29}N_2O_2$ $[M+H]^+$ = 353.2224; found = 353.2228. The ee was determined by **HPLC** analysis using Chiralpak IC column (80/20 heptane/*i*-PrOH, flow rate 1.0 ml/min; λ = 190 nm, t_S = 4.0 min., t_R = 4.8 min). **$[\alpha]^{25}_D$** = +33.3° (c = 0.3, $CHCl_3$).

3-(*tert*-butyl)-4-oxo-1,6-diphenyl-8-vinyl-2,3-diazaspiro[4.4]non-1-ene-7-carbaldehyde, 3s

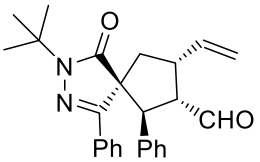

Transparent oil, yield 63%, dr = 5:2, ee = 99% major dia/95% minor dia. Major dia: **^{1}H NMR (600 MHz, $CDCl_3$)** δ 9.80 (d, *J* = 1.5 Hz, 1H), 7.93 – 7.87 (m, 2H), 7.54 – 7.43 (m, 3H), 7.17 – 7.14 (m, 3H), 7.07 – 7.02 (m, 2H), 5.91 (dt, J_1 = 16.8, J_2 = 9.8 Hz, 1H), 5.29 (dd, J_1 = 16.8, J_2 = 1.0 Hz, 1H), 5.17 (d, *J* = 10.2 Hz, 1H), 4.46 – 4.41 (m, 1H), 4.41 – 4.36 (m, 1H), 4.17 – 4.09 (m, 1H), 2.46 – 2.37 (m, 2H), 1.19 (s, 9H). **^{13}C NMR (151 MHz, $CDCl_3$)** δ 202.7, 177.0, 154.7, 138.2, 135.5, 131.6, 130.0, 129.3 (2C), 128.5 (2C), 128.20 (2C), 127.8, 126.6 (2C), 117.7, 64.4, 57.6, 55.7, 51.3, 43.5, 39.2, 28.2 (3C). **HRMS** (ESI) m/z calcd for $C_{26}H_{29}N_2O_2$ $[M+H]^+$ = 401.2224; found = 401.2221. The ee was determined by **HPLC** analysis using Chiralpak IC column (98/2 heptane/*i*-PrOH, flow rate 0.5 ml/min; λ = 190 nm, major dia: t_S = 16.2 min., t_R = 23.9 min. Minor dia: t_S = 15.1 min., t_R = 18.0 min). **$[\alpha]^{25}_D$** = -18.4° (c = 0.49, $CHCl_3$).

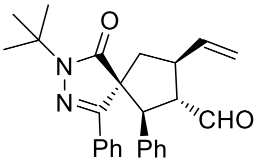

Minor dia: **^{1}H NMR (600 MHz, CDCl3)** δ 9.80 (d, J = 1.5 Hz, 1H), 7.93 – 7.87 (m, 2H), 7.54 – 7.43 (m, 3H), 7.17 – 7.14 (m, 3H), 7.07 – 7.02 (m, 2H), 5.91 (dt, J_1 = 16.8, J_2 = 9.8 Hz, 1H), 5.29 (dd, J_1 = 16.8, J_2 = 1.0 Hz, 1H), 5.17 (d, *J* = 10.2 Hz, 1H), 4.46 – 4.41 (m, 1H), 4.41 – 4.36 (m, 1H), 4.17 – 4.09 (m, 1H), 2.46 – 2.37 (m, 2H), 1.19 (s, 9H). **^{13}C NMR (151 MHz, CDCl3)** δ 202.7, 177.0, 154.7, 138.2, 135.5, 131.6, 130.0, 129.3 (2C), 128.5 (2C), 128.20 (2C), 127.8, 126.6 (2C), 117.7, 64.4, 57.6, 55.7, 51.3, 43.5, 39.2, 28.2 (3C).

6-ethyl-4-oxo-1-phenyl-3-(*p*-tolyl)-8-vinyl-2,3-diazaspiro[4.4]non-1-ene-7-carbaldehyde, 3t

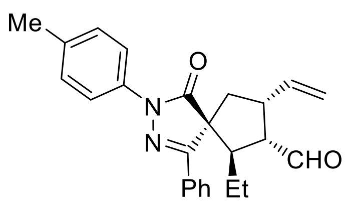

Transparent oil, yield 49%, dr 20:1, ee = 98%. **^{1}H NMR (600 MHz, $CDCl_3$)** δ 9.87 (d, *J* = 1.8 Hz, 1H), 8.00 – 7.97 (m, 2H), 7.87 (d, *J* = 8.5 Hz, 2H), 7.54 – 7.47 (m, 3H), 7.24 (d, *J* = 8.3 Hz, 2H), 5.78 (dt, J_1 = 16.9, J_2 = 9.6 Hz, 1H), 5.25 (d, *J* = 16.8 Hz, 1H), 5.14 (d, *J* = 10.2 Hz, 1H), 4.16 (qd, J_1 = 10.6, J_2 = 7.3 Hz, 1H), 3.46 (ddd, J_1 = 10.8, J_2 = 8.2, J_3 = 1.9 Hz, 1H), 3.43 – 3.37 (m, 1H), 2.37 (d, *J* = 3.5 Hz, 4H), 2.23 (dd, J_1 = 13.4, J_2 = 7.0 Hz, 1H), 1.48 – 1.36 (m, 2H), 0.69 (t, *J* = 7.5 Hz, 3H). **^{13}C NMR (151 MHz, $CDCl_3$)** δ 203.7, 175.4, 158.9, 137.0, 135.7, 135.3, 130.5, 129.6 (2C), 129.1 (2C), 127.0 (2C), 119.3 (2C), 117.6, 63.1, 58.6, 47.5, 43.6, 40.7, 23.9, 21.1, 12.8. **HRMS** (ESI) *m/z* calcd for $C_{25}H_{27}N_2O_2$ $[M+H]^+$ = 387.2067; found =

387.2069. The ee was determined by **HPLC** analysis using Chiralpak IC column (90/10 heptane/*i*-PrOH, flow rate 1.0 ml/min; λ = 190 nm, t_S = 5.1 min., t_R = 5.8 min). **$[\alpha]^{25}_D$** = +36.5° (c = 0.3, $CHCl_3$).

(5*R*,6*R*,7*S*,8*R*)-6-butyl-4-oxo-1-phenyl-3-(*p*-tolyl)-8-vinyl-2,3-diazaspiro[4.4]non-1-ene-7-carbaldehyde, 3u

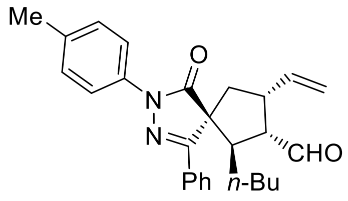

Transparent oil, yield 80%, dr 20:1, ee = 98%. **^{1}H NMR (600 MHz, $CDCl_3$)** δ 9.87 (s, 1H), 8.01 – 7.97 (m, 2H), 7.87 (d, *J* = 8.4 Hz, 2H), 7.50 (q, J_1 = 7.7, J_2 = 7.0 Hz, 3H), 7.25 (d, *J* = 8.4 Hz, 2H), 5.78 (dt, J_1 = 16.9, J_2 = 9.6 Hz, 1H), 5.24 (d, *J* = 16.9 Hz, 1H), 5.14 (d, *J* = 10.1 Hz, 1H), 4.17 (qd, J_1 = 10.1, J_2 = 6.7 Hz, 1H), 3.46 (dt, J_1 = 8.6, J_2 =4.9 Hz, 2H), 2.49 – 2.33 (m, 4H), 2.22 (dd, J_1 = 13.4, J_2 = 7.0 Hz, 1H), 1.36 (dddd, J_1 = 29.1, J_2 = 19.4, J_3 = 12.4, J_4 = 5.8 Hz, 2H), 1.13 – 1.06 (m, 2H), 1.01 (dtd, J_1 = 18.4, J_2 = 7.9, J_3 = 6.8, J_4 = 4.4 Hz, 2H), 0.69 (t, *J* = 7.3 Hz, 3H). **^{13}C NMR (151 MHz, $CDCl_3$)** δ 203.7, 175.4, 158.8, 137.0, 135.6, 135.3, 130.4, 130.0 (2C), 129.1 (2C), 126.9 (2C), 119.3 (2C), 117.6, 63.2, 58.9, 45.7, 43.6, 40.6, 30.6, 30.3, 22.7, 21.1, 13.9. **HRMS** (ESI) *m/z* calcd for $C_{27}H_{31}N_2O_2$ $[M+H]^+$ = 415.2380; found = 415.2373. The ee was determined by **HPLC** analysis using Chiralpak ODH column (99/1 heptane/*i*-PrOH, flow rate 0.5 ml/min; λ = 323 nm, t_R = 14.7 min., t_S = 16.6 min). **$[\alpha]^{25}_D$** = +38.8° (c = 1.0, $CHCl_3$).

3-(2,4-dinitrophenyl)-6-ethyl-4-oxo-1-phenyl-8-vinyl-2,3-diazaspiro[4.4]non-1-ene-7-carbaldehyde, 3v

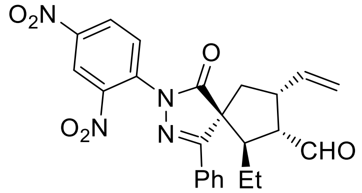

Transparent oil, yield 52%, dr 20:1, ee = 98%. **^{1}H NMR (600 MHz, $CDCl_3$)** δ 9.86 (s, 1H), 8.79 (d, *J* = 2.6 Hz, 1H), 8.52 (dd, J_1 = 9.0, J_2 = 2.6 Hz, 1H), 8.11 (d, *J* = 9.0 Hz, 1H), 7.96 (dd, J_1 = 6.8, J_2 = 3.0 Hz, 2H), 7.53 (dd, J_1 = 5.1, J_2 = 2.0 Hz, 3H), 5.75 (dt, J_1 = 16.9, J_2 = 9.6 Hz, 1H), 5.25 (d, *J* = 16.9 Hz, 1H), 5.16 (d, *J* = 10.1 Hz, 1H), 4.13 – 3.97 (m, 1H), 3.49 (q, *J* = 7.6 Hz, 1H), 3.45 – 3.37 (m, 1H), 2.40 (dd, J_1 = 13.6, J_2 = 11.0 Hz, 1H), 2.23 (dd, J_1 = 13.5, J_2 = 7.0 Hz, 1H), 1.44 (p, *J* = 7.4 Hz, 2H), 0.72 (t, *J* = 7.4 Hz, 3H). **^{13}C NMR (151 MHz, $CDCl_3$)** δ 203.2, 175.1, 162.1, 144.8, 142.1, 136.2, 134.3, 131.5, 129.4 (2C), 129.4, 127.7, 127.2 (2C), 125.1, 121.4, 118.2, 62.5, 58.5, 47.6, 43.4, 40.9, 23.9, 12.8. **HRMS** (ESI) *m/z* calcd for $C_{24}H_{23}N_4O_6$ $[M+H]^+$ = 463.1612; found = 463.1614. The ee was determined by **HPLC** analysis using Chiralpak IA column (95/5 heptane/*i*-PrOH, flow rate 1.0 ml/min; λ = 190 nm, t_S = 28.1 min., t_R = 30.9 min). **$[\alpha]^{25}_D$** = +30.0° (c = 0.1, $CHCl_3$).

6-butyl-3-(2,4-dinitrophenyl)-4-oxo-1-phenyl-8-vinyl-2,3-diazaspiro[4.4]non-1-ene-7-carbaldehyde, 3w

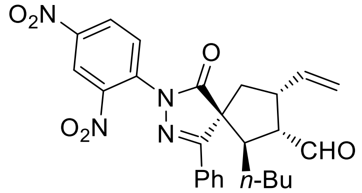

Transparent oil, yield 61%, dr 20:1, ee = 98%. **^{1}H NMR (600 MHz, $CDCl_3$)** δ 9.85 (d, *J* = 1.6 Hz, 1H), 8.80 (d, *J* = 2.5 Hz, 1H), 8.52 (dd, J_1 = 9.0, J_2 = 2.5 Hz, 1H), 8.11 (d, *J* = 9.0 Hz, 1H), 8.02 – 7.92 (m, 2H), 7.54 (dd, J_1 = 5.1, J_2 = 2.0 Hz, 3H), 5.75 (ddd, J_1 = 16.9, J_2 = 10.1, J_3 = 9.0 Hz, 1H), 5.25 (dt, J_1 = 16.9, J_2 = 1.2 Hz, 1H), 5.16 (dt, *J* = 10.3, J_2 = 1.0 Hz, 1H), 4.12 – 4.02 (m, 1H), 3.55 (td, J_1 = 8.0, J_2 = 6.8 Hz, 1H), 3.42 (ddd, J_1 = 11.2, J_2 = 8.0, J_3 = 1.7 Hz, 1H), 2.40 (dd, J_1 = 13.5, J_2 = 11.1 Hz, 1H), 2.23 (dd, J_1 = 13.5, J_2 = 6.9 Hz, 1H), 1.45 – 1.32 (m, 2H), 1.19 – 1.08 (m, 2H), 1.06 – 0.98 (m, 2H), 0.72 (t, *J* = 7.3 Hz, 3H). **^{13}C NMR (151 MHz, $CDCl_3$)** δ 203.2, 175.2, 162.0, 144.8, 142.1, 136.2, 134.3, 131.5, 129.4 (2C), 129.4, 127.2

(2C), 125.1, 121.4, 118.2, 62.7, 58.7, 45.9, 43.5, 40.8, 30.5, 30.4, 22.6, 13.8. **HRMS** (ESI) *m/z* calcd for $C_{26}H_{27}N_2O_2$ $[M+H]^+$ = 491.1925; found = 491.1927. The ee was determined by **HPLC** analysis using Chiralpak IA column (80/20 heptane/*i*-PrOH, flow rate 1.0 ml/min; λ = 190 nm, t_R = 10.9 min., t_S = 12.9 min). **[α]$^{25}_D$** = +47.9° (c = 1.1, $CHCl_3$).

6-(4-bromophenyl)-4-oxo-1,3-diphenyl-8-vinyl-2,3-diazaspiro[4.4]non-1-ene-7-carbaldehyde, 3x

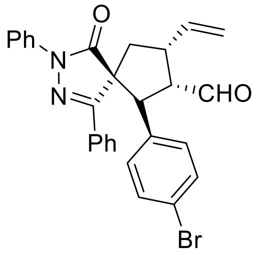

Yellowish solid, yield 85 %, m.p. = 62,5 °C, dr = 1.4:1, ee = 99/77 %; **major dia**:**^{1}H NMR (600 MHz, CDCl$_3$)** δ 9.80 (d, J = 1.3 Hz, 1H), 8.01 – 7.93 (m, 2H), 7.65 – 7.61 (m, 2H), 7.59 – 7.52 (m, 3H), 7.35 (dd, J_1 = 8.7, J_2 = 7.4 Hz, 2H), 7.25 (d, *J* = 8.5 Hz, 2H), 7.20 – 7.17 (m, 1H), 6.94 (d, *J* = 8.5 Hz, 2H), 5.89 (dt, J_1 = 16.8, J_2 = 9.8 Hz, 1H), 5.39–5.16 (m, 2H), 4.48 (d, *J* = 10.7 Hz, 1H), 4.39 (td, J_1 = 10.6, J_2 = 1.3 Hz, 1H), 4.27–4.18 (m, 1H), 2.54 (qd, J_1 = 14.0, J_2 = 8.0 Hz, 2H) ppm; **^{13}C NMR (151 MHz, CDCl$_3$)** δ 201.8, 175.1, 157.3, 137.5, 137.4, 134.4, 131.7 (2C), 130.8, 130.6, 129.6 (2C), 129.4, 128.9 (2C), 126.8 (2C), 125.8, 122.1, 119.7 (2C), 118.1, 64.6, 56.2, 51.1, 43.2, 39.8 ppm. IR (KBr): ν = 3479, 3408, 3067, 2932, 2729, 1697, cm^{-1}; HRMS (ESI) m/z calcd for $[M+Na]^+$ = 521.0835; found = 521.0830. The ee was determined by HPLC analysis using Chiralpak IA column (80/20 heptane/i-PrOH, flow rate 1.0 ml/min; λ = 190 nm, **minor dia**: t_{major} = 5.8 min., t_{minor} = 10.9 min, **major dia**: t_{major} = 7.1 min., t_{minor} = 10.0 min); [α]$^{25}_D$ = +23.3° (c = 0.37, $CHCl_3$).

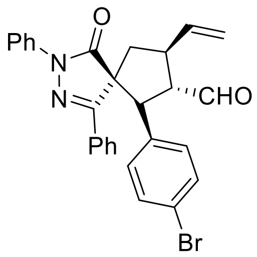

minor dia:**^{1}H NMR (600 MHz, CDCl$_3$)** δ 9.70 (d, *J* = 2.4 Hz, 1H), 8.05 – 7.92 (m, 2H), 7.57 – 7.53 (m, 3H), 7.52 – 7.49 (m, 2H), 7.34 – 7.30 (m, 2H), 7.27 (d, *J* = 8.5 Hz, 2H), 7.17 – 7.14 (m, 1H), 6.92 (d, *J* = 8.5 Hz, 2H), 6.10 (ddd, J_1 = 16.9, J_2 = 10.1, J_3 = 8.1 Hz, 1H), 5.37 – 5.19 (m, 2H), 4.12 (ddd, J_1 = 12.9, J_2 = 10.7, J_3 = 2.4 Hz, 1H), 4.05 (d, J = 12.4 Hz, 1H), 3.46–3.37 (m, 1H), 2.88 (dd, J_1 = 14.6, J_2 = 9.7 Hz, 1H), 2.40 (dd, J_1 = 14.7, J_2 = 9.5 Hz, 1H) ppm, **^{13}C NMR (151 MHz, CDCl3)** δ 201.4, 175.7, 157.7, 138.1, 137.2, 132.7, 130.9, 129.9, 129.6 (2C), 129.5 (2C), 128.9 (2C), 126.4 (2C), 125.9, 122.4, 119.8 (2C), 117.6, 63.03, 57.7, 54.9, 46.0, 38.5 ppm.

5. Derivatizations

5.1. Synthesis of **11**

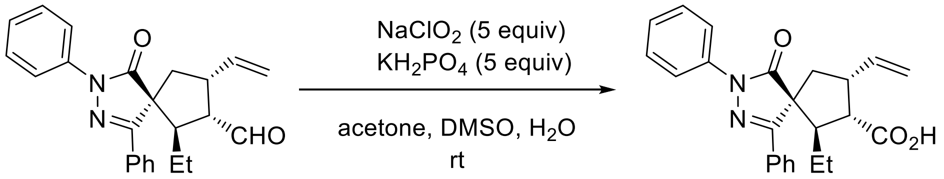

Scheme S3

Spiro compound **3o** (1 equiv, 65 mg, 0.17 mmol) was dissolved in acetone (5 mL) and DMSO (2 mL). To the solution was added dropwise a mixture of $NaClO_2$ (5 equiv, 77 mg, 0.85 mmol) and KH_2PO_4 (5 equiv, 0.85 mmol, 116 mg) in water (5 mL). The reaction was stirred at room temperature for 3 hours. The reaction mixture was then evaporated, 10 ml of water were added and was extracted with Et_2O (3x15 mL). The organic layer was washed with brine and then dried over $MgSO_4$. After filtration, the crude was purified on silica gel, obtaining the corresponding acid derivative **11** as white solid (58 mg, yield 88%, 99% ee).

6-ethyl-4-oxo-1,3-diphenyl-8-vinyl-2,3-diazaspiro[4.4]non-1-ene-7-carboxylic acid, 11

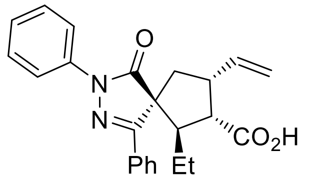

White solid, m.p. = 148.9 °C, yield 88 %, ee 99 %. **^{1}H NMR (600 MHz, CDCl$_3$)** δ 8.11 – 8.08 (m, 2H), 8.03 (dq, J_1 = 7.1, J_2 = 1.2 Hz, 2H), 7.54 – 7.49 (m, 3H), 7.48 – 7.43 (m, 2H), 7.28 – 7.21 (m, 1H), 5.90 (ddd, J_1 = 16.8, J_2 = 10.1, J_3 = 8.6 Hz, 1H), 5.22 (dt, J_1 = 17.0, J_2 = 1.2 Hz, 1H), 5.17 – 5.12 (m, 1H), 4.03 (qd, J_1 = 10.4, J_2 = 7.5 Hz, 1H), 3.41 (dd, J_1 = 10.7, J_2 = 8.4 Hz, 1H), 3.32 (td, J_1 = 8.6, J_2 = 6.0 Hz, 1H), 2.62 (dd, J_1 = 13.4, J_2 = 10.7 Hz, 1H), 2.29 (dd, J_1 = 13.4, J_2 = 7.1 Hz, 1H), 1.61 – 1.50 (m, 1H), 1.49 – 1.40 (m, 2H) 0.80 (t, J = 7.4 Hz, 3H) ppm. **^{13}C NMR (151 MHz, CDCl$_3$)** δ 180.5, 175.7, 158.7, 138.0, 136.8, 130.5, 130.3, 129.1 (2C), 129.0 (2C), 126.9 (2C), 125.5, 119.2 (2C), 117.8, 63.6, 52.7, 51.8, 43.6, 40.6, 24.0, 12.5 ppm. **HRMS** (ESI) *m/z* calcd for $C_{24}H_{25}N_2O_3$ $[M+H]^+$ = 389.1860; found = 389.1857. The ee was determined by **HPLC** analysis using Chiralpak IA (70/30 heptane/*i*-PrOH, flow rate 0.2 ml/min; 35 °C, λ = 254 nm, t_S = 30.5 min., t_R = 41.0 min.). **$[\alpha]^{25}_D$** = +90.9° (c = 0.4, $CHCl_3$).

5.2. Synthesis of **12**

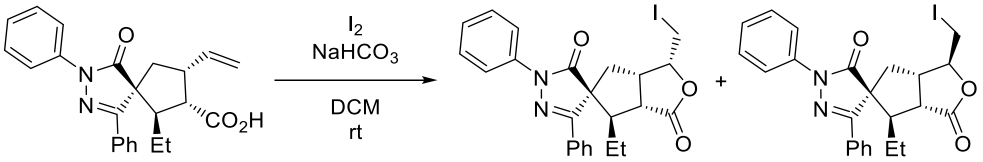

Scheme S4

Following the published procedure,[5] a saturated water solution of $NaHCO_3$ (1 mL) and I_2 (1.1 equiv, 0,09 mmol, 11 mg) was added to the solution of acid **11** (1 equiv, 0.08 mmol, 30 mg) in DCM (1 mL) under vigorous stirring at room temperature. After full conversion (TLC monitoring) was added to the reaction mixture a sat. water solution of Na_2SO_3/$NaHCO_3$ (1:1, 3 mL). The organic layer was

[5] Kurth, M. J., Brown, E. G. *J. Am. Chem. Soc.* **1987**, *109*, 6844-6845.

separated, washed with brine, dried over $MgSO_4$, filtered and concentrated *in vacuo*. The crude was purified on silica gel, leading to the isolation of both diastereomers of the corresponding lactone derivative in an overall yield of 81% (dr = 1:1).

4-ethyl-1-(iodomethyl)-1',3'-diphenyl-3a,4,6,6a-tetrahydro-1H,3H-spiro[cyclopenta[c]furan-5,4'-pyrazole]-3,5'(1'H)-dione, 12

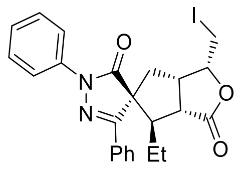

Dia 1: Transparent oil, yield 37%; ee 99%. **^{1}H NMR (600 MHz, $CDCl_3$)** δ 8.01 – 7.95 (m, 2H), 7.83 – 7.77 (m, 2H), 7.52 – 7.40 (m, 5H), 7.27 – 7.22 (m, 1H), 4.86 (dt, J_1 = 9.7, J_2 = 5.8 Hz, 1H), 4.20 (qd, J_1 = 9.3, J_2 = 5.7 Hz, 1H), 3.46 (dd, J_1 = 10.2, J_2 = 5.8 Hz, 1H), 3.24 (dd, J_1 = 9.0, J_2 = 5.0 Hz, 1H), 3.13 – 3.06 (m, 2H), 2.35 (d, J_1 = 9.5 Hz, 2H), 1.69 (ddt, J_1 = 13.3, J_2 = 9.2, J_3 = 7.3 Hz, 1H), 1.59 – 1.47 (m, 1H), 0.91 (t, J_1 = 7.4 Hz, 3H) ppm. **^{13}C NMR (151 MHz, $CDCl_3$)** δ 178.8, 174.7, 157.7, 137.8, 130.8, 129.9, 129.4 (2C), 129.1 (2C), 126.6 (2C), 125.8, 119.3 (2C), 79.0, 64.1, 52.3, 51.4, 42.7, 33.8, 25.0, 12.7, -0.8 ppm. **HRMS** (ESI) m/z calcd for $C_{24}H_{24}IN_2O_3$ $[M+H]^+$ = 515.0826; found = 515.0829. The ee was determined by **HPLC** analysis using Chiralpak IC (80/20 heptane/*i*-PrOH, flow rate 1.0 ml/min; λ = 254 nm, t_{major} = 8.2 min., t_{minor} = 9.2 min.). **$[\alpha]^{25}_D$** = +1.3° (c = 0.2, $CHCl_3$).

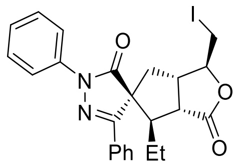

Dia 2: Transparent oil, yield 44%, ee 98%. **^{1}H NMR (600 MHz, $CDCl_3$)** δ 7.98 (d, J = 7.8 Hz, 2H), 7.80 (dd, J1= 7.4, J2= 2.1 Hz, 2H), 7.53 – 7.42 (m, 5H), 7.27 – 7.22 (m, 1H), 4.43 (ddd, J1= 6.6, J2= 3.9, J3= 2.4 Hz, 1H), 3.76 – 3.68 (m, 1H), 3.49 – 3.41 (m, 2H), 3.33 (dd, J_1= 10.5, J_2= 7.3 Hz, 1H), 2.97 (dd, J_1= 14.6, J_2= 7.5 Hz, 1H), 2.54 (dd, J_1= 13.4, J_2= 8.1 Hz, 1H), 2.44 (dd, J_1= 13.4, J_2= 10.3 Hz, 1H), 1.81 – 1.70 (m, 1H), 1.59 – 1.49 (m, 1H), 0.91 (t, J = 7.5 Hz, 3H) ppm. **^{13}C NMR (151 MHz, CDCl3)** δ 178.2, 174.8, 157.6, 137.8, 130.8, 130.1, 129.3 (2C), 129.1 (2C), 126.7 (2C), 125.8, 119.2, 82.7, 65.1, 52.1, 50.0, 45.1, 40.6, 24.7, 12.6, 8.0 ppm. **HRMS** (ESI) *m/z* calcd for $C_{24}H_{24}IN_2O_3$ $[M+H]^+$ = 515.0826; found = 515.0823. The ee was determined by **HPLC** analysis using Chiralpak IA (80/20 heptane/*i*-PrOH, flow rate 1.0 ml/min; λ = 254 nm, t_{minor} = 8.6 min., t_{major} = 15.8 min.). $[\alpha]^{25}_D$ = +20.7° (c = 0.8, $CHCl_3$).

5.3. Synthesis of **13**

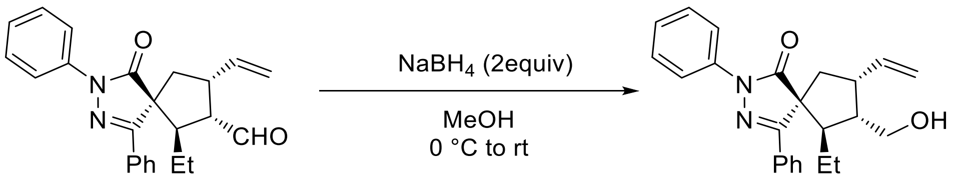

Scheme S5

To the solution of spiro compound **3o** (1 equiv, 0,16 mmol, 58 mg) in MeOH (5 mL) was slowly added $NaBH_4$ (2 equiv, 0.32 mmol, 12 mg) at 0 °C. Reaction mixture was stirred until full conversion (TLC monitoring) and then was to the reaction mixture added cooled mixture of EtOAc/1 M HCl (1:1, 10 mL). Organic layer was separated, washed with brine and dried over $MgSO_4$. After filtration and evaporation to dryness was the crude separated on silica obtaining compound xx as transparent oil (48 mg, 83%).

6-ethyl-7-(hydroxymethyl)-2,4-diphenyl-8-vinyl-2,3-diazaspiro[4.4]non-3-en-1-one, 13

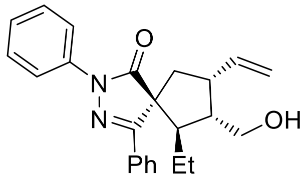

Transparent oil, yield 83%; ee 99%. **^{1}H NMR (600 MHz, $CDCl_3$)** δ 8.10 – 8.07 (m, 2H), 8.05 – 8.02 (m, 2H), 7.50 – 7.41 (m, 5H), 7.25 – 7.19 (m, 1H), 6.15 (ddd, J_1 = 17.2, J_2 = 10.3, J_2 = 8.1 Hz, 1H), 5.24 – 5.15 (m, 2H), 3.89 (dd, J_1 = 11.1, J_2 = 3.2 Hz, 1H), 3.86 – 3.79 (m, 1H), 3.72 (dd, J_1 = 11.1, J_2 = 4.9 Hz, 1H), 2.89 (q, J = 7.6 Hz, 1H), 2.59 – 2.44 (m, 2H), 2.18 (dd, J_1 = 13.1, J_2 = 7.1 Hz, 1H), 1.63 – 1.46 (m, 2H), 0.75 (t, J = 7.5 Hz, 3H) ppm. **^{13}C NMR (151 MHz, $CDCl_3$)** δ 175.9, 160.0, 139.2, 138.1, 130.6, 130.1, 128.8 (2C), 128.7 (2C), 126.9 (2C), 125.1, 119.0 (2C), 116.4, 63.0, 63.0, 49.6, 48.8, 42.6, 40.7, 24.2, 12.5 ppm. **HRMS** (ESI) *m/z* calcd for $C_{24}H_{27}N_2O_2$ $[M+H]^+$ = 375.2067; found = 375.2066. The ee was determined by **HPLC** analysis using Chiralpak AD (95/5 heptane/*i*-PrOH, flow rate 1.0 ml/min; λ = 190 nm, t_{minor} = 13.2 min., t_{major} = 15.5 min.). **$[\alpha]^{25}_D$** = +94.9° (c = 1.2, $CHCl_3$).

5.4. Synthesis of **14**

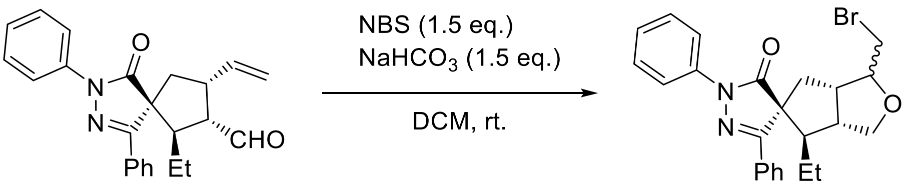

Scheme S6

According published procedure,[6] in a flask charged with homoallylic alcohol (1 eq., 0.08 mmol, 29 mg) and DCM (2 mL) at 0 °C, was added *N*-bromosuccinimide (1.5 eq., 0.12 mmol, 21 mg) and $NaHCO_3$ (1.5 eq., 0.12 mmol, 10 mg). The reaction mixture was allowed to stir at 0 °C and then allowed to reach room temperature. When all the starting material was consumed (TLC monitoring), water was added to the reaction mixture and was then extracted with Et_2O (3 x 10 mL). The organic layer was washed with brine, dried over $MgSO_4$, concentrated and purified on silica. The corresponding product **14** was isolated as a mixture of diastereomers as a transparent oil (32 mg, dr = 2:1, 89 %).

(1*S*,3a*R*,4*R*,5*R*,6a*S*)-1-(bromomethyl)-4-ethyl-1',3'-diphenyl-3a,4,6,6a-tetrahydro-1H,3H-spiro[cyclopenta[c]furan-5,4'-pyrazol]-5'(1'H)-one, 14

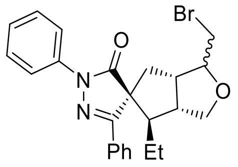

Transparent oil, yield 89%; dr = 2:1, ee 98/99 %. **Major dia**: **^{1}H NMR (600 MHz, $CDCl_3$)** δ 8.03 – 7.97 (m, 2H), 8.03 – 7.97 (m, 2H), 7.50 – 7.46 (m, 3H), 7.47 – 7.40 (m, 2H), 7.25 – 7.19 (m, 1H), 4.16 – 4.09 (m, 2H), 3.80 (dd, J_1 = 9.6, J_2 = 3.0 Hz, 1H), 3.46 (dd, J_1 = 10.3, J_2 = 6.6 Hz, 1H), 3.41 – 3.35 (m, 1H), 3.36 (dd, J_1 = 10.3, J_2 = 6.8 Hz, 1H), 3.17 (tdd, J_1 = 9.8, J_2 = 6.8, J_3 = 3.0 Hz, 1H), 2.58 (dt, J_1 = 9.3, J_2 = 7.3 Hz, 1H), 2.47 (dd, J_1 = 13.6, J_2 = 8.8 Hz, 1H), 2.39 (dd, J_1 = 13.7, J_1 = 8.4 Hz, 1H), 1.42 (p, J = 7.4 Hz, 2H), 0.79 (t, J = 7.6 Hz, 3H) ppm. **^{13}C NMR (151 MHz, $CDCl_3$)** δ 176.1, 158.0, 138.5, 130.6, 130.5, 129.1 (2C), 129.0 (2C), 126.8 (2C), 125,4, 119.1 (2C), 85.9, 73.4, 67.1, 55.2, 50.6, 48.6, 39.9, 33.5, 23.5, 12.8 ppm. **HRMS** (ESI) *m/z* calcd for $C_{24}H_{26}BrN_2O_2$ $[M+H]^+$ = 453.1172; found = 453.1174. The ee was determined by **HPLC** analysis using Chiralpak IC (99/1 heptane/i-PrOH, flow rate 1.0

[6] Lee, A S.-Y; Tsao, K.-W.; Chang, Y-T. ; Chu, S. F. *Tetrahedron Lett*. **2007**, *48*, 6790.

ml/min; λ = 254 nm, **minor dia**: t_{minor} = 8.7 min., t_{major} = 9.1 min, **major dia**: t_{major} = 10.5 min., t_{minor} = 15.1 min). **$[\alpha]^{25}_D$** = +76.8° (c = 0.4, $CHCl_3$).

Minor dia: **^{1}H NMR (600 MHz, $CDCl_3$)** δ 8.03 – 7.97 (m, 2H), 8.03 – 7.97 (m, 2H), 7.50 – 7.46 (m, 3H), 7.47 – 7.40 (m, 2H), 7.25 – 7.19 (m, 1H), 4.00 (q, *J* = 6.9 Hz, 1H), 3.86 (d, *J* = 4.2 Hz, 2H), 3.68 – 3.59 (m, 1H), 3.58 (dd, J_1 = 10.5, J_2 = 7.2 Hz, 1H), 3.41 – 3.35 (m, 1H), 3.04 (tt, J_1 = 8.6, *J2* = 4.0 Hz, 1H), 2.52 – 2.46 (m, 1H), 2.38 – 2.31 (m, 1H), 2.14 (dd, J_1 = 13.0, J_2 = 8.1 Hz, 1H), 1.42 (p, *J* = 7.4 Hz, 2H), 0.84 – 0.78 (m, 3H) ppm. **^{13}C NMR (151 MHz, $CDCl_3$)** δ 175.8, 158.2, 138.1, 130.6, 130.5, 129.2 (2C), 129.0 (2C), 126.8 (2C), 125.4, 119.1 (2C), 81.2, 74.6, 65.8, 56.3, 50.3, 45.9, 33.9, 29.4, 23.8, 12.9 ppm.

5.5. Synthesis of **16**

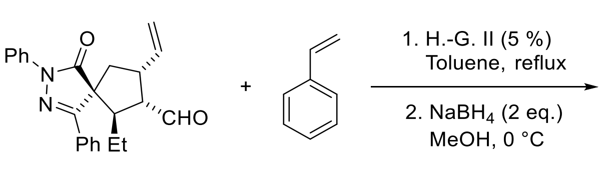

Scheme S7

Spiro compound **3o** (1 eq., 0.054 mmol, 20 mg), styrene (1 eq., 0.054 mmol, 6 mg) and H.-G. II catalyst (5%, 0.003 mmol, 1.8 mg) were refluxed in toluene (1 mL) until reaching full conversion (TLC monitoring). The solvent was then removed under vacuum and methanol (1 mL) was added, followed by addition of $NaBH_4$ (2 eq., 0.11 mmol, 4 mg) at 0 °C. The reaction mixture was then stirred at room temperature until full conversion (TLC monitoring) and then quenched by adding of 1M HCl/EtOAc (2 mL, 1:1) at 0 °C. The organic layer was separated and dried over $NaSO_4$. After filtration and evaporation the crude material was loaded on silica. Product **16** was isolated as a transparent oil (10 mg, 42% yield).

(*E*)-6-ethyl-4-oxo-1,3-diphenyl-8-styryl-2,3-diazaspiro[4.4]non-1-ene-7-carbaldehyde, 16

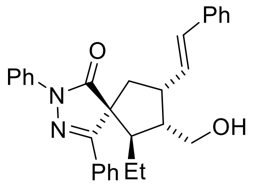

Transparent oil, yield 42%; dr > 20:1; ee 98%. The ee was determined by HPLC analysis using Chiralpak IA (80/20 heptane/i-PrOH, flow rate 1.0 ml/min; λ = 190 nm, t_{major} = 6.0 min., t_{minor} = 7.0 min); **^{1}H NMR (600 MHz, CDCl3)** δ 8.12 – 8.09 (m, 2H), 8.06 – 8.02 (m, 2H), 7.51 – 7.43 (m, 5H), 7.40 – 7.36 (m, 2H), 7.32 (dd, J_1 = 8.4, J_1 = 6.9 Hz, 2H), 7.25 – 7.18 (m, 2H), 6.58 – 6.46 (m, 2H), 3.99 (tt, J_1 = 10.4, J_2 = 7.5 Hz, 1H), 3.93 (dd, J_1 = 10.9, J_2 = 3.1 Hz, 1H), 3.77 (dd, J_1 = 10.9, J_2 = 4.7 Hz, 1H), 2.95 (q, *J* = 7.6 Hz, 1H), 2.60 (dd, J_1 = 13.1, J_2 = 10.8 Hz, 1H), 2.53 (ddd, J_1 = 10.8, J_2 = 4.8, J_3 = 2.9 Hz, 1H), 2.25 (dd, J_1 = 13.1, J_2 = 7.1 Hz, 1H), 1.68 – 1.50 (m, 1H), 0.77 (t, *J* = 7.5 Hz, 3H) ppm; **^{13}C NMR (151 MHz, CDCl3)** δ 176.1, 160.3, 138.3, 137.4, 131.8, 130.9, 130.6, 130.3, 129.0 (2C), 129.0 (2C), 128.8 (2C), 127.5, 127.2 (2C), 126.4 (2C), 125.3, 119.3 (3C), 63.4, 63.3, 49.7, 49.6, 42.7, 41.7, 24.5, 12.7 ppm; $[\alpha]^{25}_D$ = +35,4° (c = 0.24, $CHCl_3$); IR (KBr): ν = 3551, 3485, 3428, 3411, 2962, 2872, 1706, 1497, 1317 cm^{-1}; HRMS (ESI) m/z calcd for $C_{30}H_{30}N_2O_2$ [M+Na]+ = 473.2199; found = 473.2198.

5.6. Synthesis of **17**

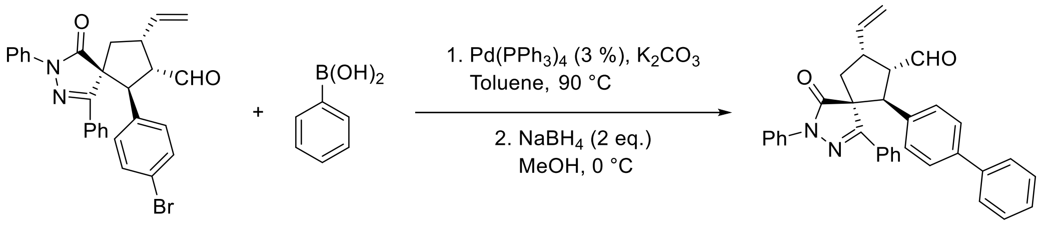

Scheme S8

According to a published procedure,[7] to the suspension of spiro compound **3w** (1 eq., 0.05 mmol, 25 mg), phenylboronic acid (2 eq., 0.1 mmol, 12 mg) and K_2CO_3 (2 eq., 0.1 mmol, 14 mg) in dry toluene (1 mL) was added $Pd(PPh_3)_4$ (3 %, 0.0015 mmol, 1.8 mg) under argon atmosphere. The reaction mixture was then heated at 90 °C until full conversion (TLC monitoring), then the solvent was removed by vacuum and methanol (1 mL) was added followed by addition of $NaBH_4$ (2 eq., 0.1 mmol, 3.8 mg) at 0 °C. The reaction mixture was then stirred at room temperature until full conversion (TLC monitoring) and then quenched by adding of 1M HCl/EtOAc (1 mL, 1:1) at 0°C. The organic layer was separated and dried over $NaSO_4$. After filtration and evaporation, the crude material was loaded on silica and the corresponding product **17** was isolated as a mixture of diastereomers as a transparent oil (15 mg, 60% yield).

6-([1,1'-biphenyl]-4-yl)-7-(hydroxymethyl)-2,4-diphenyl-8-vinyl-2,3-diazaspiro[4.4]non-3-en-1-one, 17

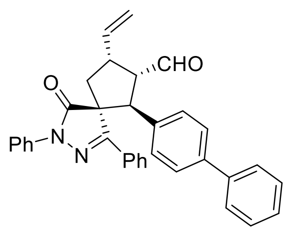

Transparent oil, yield 60%; dr 1.2:1, ee 69/98%.; **major dia**: **^{1}H NMR (600 MHz, $CDCl_3$)** δ 7.94-7.00 (m, 19H), 6.28 (dt, J_1 = 16.9, J_2 = 9.7 Hz, 1H), 5.37 – 5.11 (m, 2H), 4.02 (d, J = 11.4 Hz, 1H), 3.86 – 3.79 (m, 1H), 3.77 (dd, J_1 = 11.5, J_2 = 3.6 Hz, 1H), 3.67–3.63 (m, 1H), 3.60–3.54 (m, 1H), 2.58 (qd, J_1 = 14.0, J_2 = 7.8 Hz, 2H) ppm. **^{13}C NMR (151 MHz, $CDCl_3$)** δ 175.8, 158.1, 139,7, 137.6, 134.7, 130.5-119.9 (22xC), 116.9, 77.2, 65.3, 61.8, 54.4, 46.1, 45.7, 43.5, 39.1 ppm. The ee was determined by HPLC analysis using Chiralpak IA (90/10 heptane/i-PrOH, flow rate 1.0 ml/min; λ = 190 nm, **major dia**: t_{major} = 7.7 min., t_{minor} = 14.9 min, **minor dia**: t_{minor} = 24.6 min., t_{minor} = 40.3 min) $[\alpha]^{25}_D$ = +22,7° (c = 0.44, $CHCl_3$); IR (KBr): ν = 3482, 3425, 2932, 2463, 1963, 1712, 1497 cm^{-1}; HRMS (ESI) m/z calcd for $C_{34}H_{31}N_2O_2$ $[M+H]^+$ = 499.2380; found = 499.2382.

minor dia: **^{1}H NMR (600 MHz, $CDCl_3$)** δ 7.94-6.95 (m, 19 H), 6.08 (ddd, J_1 = 16.7, J_1 = 10.0, J_1 = 8.6 Hz, 1H), 5.44 – 5.18 (m, 2H), 3.92 (d, J = 12.5 Hz, 1H), 3.87 – 3.81 (m, 1H), 3.70 – 3.64 (m, 1H), 3.26 (p, J = 9.6 Hz, 1H), 3.12 (ddt, J_1 = 14.4, J_2 = 11.1, J_3 = 3.4 Hz, 1H), 2.80 (dd, J_1 = 14.6, J_2 = 9.4 Hz, 1H), 2.36 (dd, J_1 = 14.6, J2 = 9.7 Hz, 2H) ppm; **^{13}C NMR (151 MHz, CDCl3)** δ 176.6, 159.0, 140.7, 137.5, 133.8, 130.5-120.0 (22xC),116.8, 63.4, 60.1, 55.5, 47.9, 45.7, 38.3 ppm

[7] Waddell, T. G.; Carter, A. D.; Miller, T. D. *J. Org. Chem*. **1992**, *57*, 381.

5.7. Synthesis of **19** and **20**

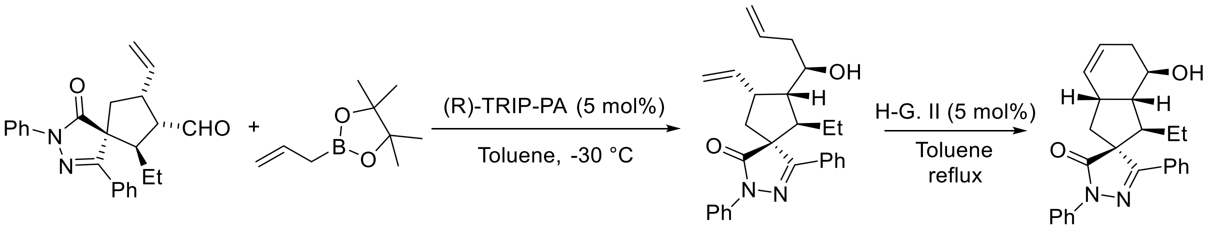

Scheme S9

Allylic alkylation using (*R*)-TRIP-PA catalyst

According to a published procedure,[8] in a schlenk flask which was evacuated, flame dried and filled with argon was added (*R*)-TRIP-PA catalyst (0.005 eq., 0.0054 mmol, 4.1 mg), aldehyde (1eq., 0.108 mmol, 40 mg) and dry toluene (1.5 mL). The reaction mixture was then cooled to -30 °C followed by addition of allylboronic acid pinacol ester (1.2 eq., 0.13 mmol, 22 mg). The reaction mixture was then stirred overnight at the same temperature and then directly loaded on silica. Purification with a flash column chromatography (hexane/EtOAc) led to isolation of corresponding product as a single diastereomer in form of a transparent oil (34 mg, 77%).

6-ethyl-7-(1-hydroxybut-3-en-1-yl)-2,4-diphenyl-8-vinyl-2,3-diazaspiro[4.4]non-3-en-1-one, 19

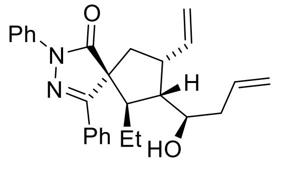

Transparent oil, yield 77%; dr > 20:1; **^{1}H NMR (600 MHz, $CDCl_3$)** δ 8.31 – 8.27 (m, 2H), 8.11 – 7.99 (m, 2H), 7.51 – 7.42 (m, 5H), 7.23 (m, 1H), 6.12 (ddd, J_1 = 17.2, J_2 = 10.2, J_3 = 8.2 Hz, 1H), 5.83 (dddd, J_1 = 17.0, J_2 = 10.2, J_3 = 8.3, J_4 = 5.7 Hz, 1H), 5.26 – 5.06 (m, 4H), 4.08 – 3.89 (m, 2H), 3.19 (ddd, J_1 = 11.0, J_2 = 6.3, J_3 = 4.3 Hz, 1H), 2.70 (t, J = 12.6 Hz, 1H), 2.29-2.17 (m, 3H), 2.03 (dd, J_1 = 12.5, J_2 = 6.4 Hz, 1H), 1.94 (bs, 1H), 1.85 (ddq, J_1 = 14.6, J_2 = 11.3, J_3 = 7.3 Hz, 1H), 1.55 (dqd, J_1 = 15.1, J_2 = 7.6, J_3 = 4.3 Hz, 1H), 0.62 (t, J = 7.5 Hz, 3H) ppm; **^{13}C NMR (151 MHz, $CDCl_3$)** δ 176.2, 161.2, 139.3, 138.5, 135.2, 130.6, 130.1, 129.0 (2C), 128.8 (2C), 127.4 (2C), 125.2, 119.3 (2C), 118.8, 116.6, 70.4, 63.0, 52.5, 46.9, 43.8, 42.6, 40.8, 27.0, 12.3 ppm; [α]25D = +72.3° (c = 0.92, CHCl3); IR (KBr): ν = 3554, 3485, 3073, 2968, 2929, 1703, 1323, 1308 cm-1; HRMS (ESI) m/z calcd for $C_{27}H_{30}N_2O_2$ [M+Na]+ =437.2199; found = 437.2199.

Intramolecular metathesis

To the flask charged with compound **19** (1eq., 0.065 mmol, 27 mg) in dry toluene (2 mL) was added H.-G. II catalyst (5 %, 0.0033 mmol, 2 mg). The reaction mixture was then heated to reflux overnight until full conversion was reached (TLC monitoring). The crude product was then purified with flash column chromatography on silica gel, leading to the isolation of product as a transparent oil as single diastereomer (21 mg, 91%).

[8] Jain, P.; Antilla, J. C. *J. Am. Chem. Soc.* **2010**, *132*, 11884.

3-ethyl-4-hydroxy-1',3'-diphenyl-1,3,3a,4,5,7a-hexahydrospiro[indene-2,4'-pyrazol]-5'(1'H)-one, 20

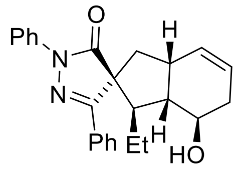

Transparent oil, yield 91%; dr > 20:1; **^{1}H NMR (600 MHz, $CDCl_3$)** δ 8.02 (d, *J* = 8.1 Hz, 2H), 7.96 (dd, J_1 = 6.6, J_2 = 3.0 Hz, 2H), 7.44 (ddd, J_1 = 7.4, J_2 = 5.6, J_3 = 2.6 Hz, 5H), 7.22 (t, *J* = 7.4 Hz, 1H), 5.81 – 5.77 (m, 1H), 5.76 – 5.69 (m, 1H), 3.97 (q, *J* = 5.6 Hz, 1H), 3.55 (dq, J_1 = 8.1, J_2 = 2.7 Hz, 1H), 2.68 (td, J_1 = 8.6, J_2 = 5.9 Hz, 1H), 2.56 (q, *J* = 8.2 Hz, 1H), 2.46 (dd, J_1 = 13.8, J_2 = 8.3 Hz, 1H), 2.37 – 2.31 (m, 1H), 2.25 (dd, J_1 = 13.8, J_2 = 7.7 Hz, 1H), 2.22 – 2.16 (m, 1H), 1.66 – 1.54 (m, 3H), 0.71 (t, *J* = 7.5 Hz, 3H) ppm; **^{13}C NMR (151 MHz, $CDCl_3$)** δ 176.6, 160.4, 138.3, 130.8, 130.6, 130.3, 129.0, 128.9, 128.9, 126.9, 125.3, 123.0, 119.3, 68.9, 62.6, 52.2, 49.3, 40.8, 37.1, 31.4, 24.3, 12.6 ppm; [α]25D = +15.9° (c = 0.32, CHCl3); IR (KBr): ν = 3548, 3482, 3464, 3022, 2962, 2932, 2875, 1703, 1682, 1601, 1314 cm-1; HRMS (ESI) m/z calcd for $C_{25}H_{26}N_2O_2$ [M+Na]+ = 409.1886; found = 409.1885.

6. Configuration studies

Compound **3n**

Full assignment of the hydrogen chemical shifts was obtained by 2D COSY experiments, starting from the methyl signal that is coupled with H-2 at 3.20 ppm (carbon numbering as from Figure S1). H-3 was found at 3.44 ppm by correlation with H-2 and with CHO. H-4 was then assigned to the signal at 3.96 ppm by COSY correlation with H-6 and H-3. The two diastereotopic hydrogens H-5a and H-5b were finally assigned by correlation with H4 and from HSQC spectrum. The relative configuration of the four stereogenic carbons was then determined by NOE experiments (Figures A and B).

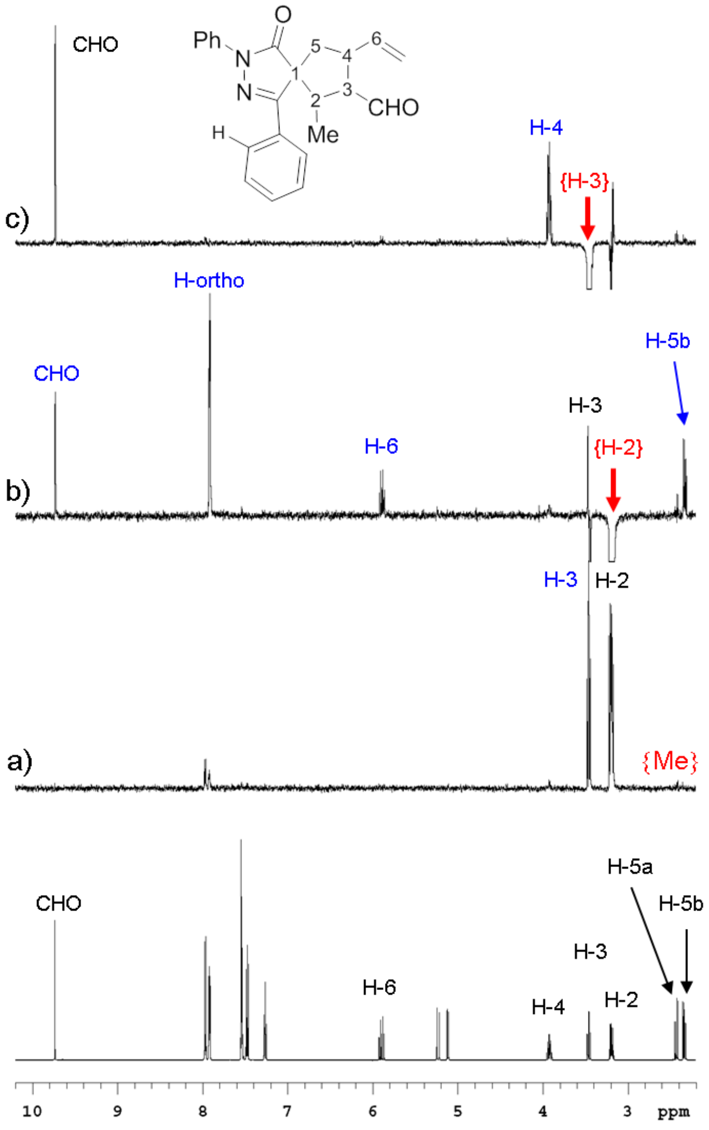

Figure S1. DPFGSE-NOE spectra of compound **3n** (600 MHz in CD_3CN)

On saturation of the methyl signal (trace a in Figure S1) a strong NOE is visible on H-3 (the NOE on H-2 is a "control" signal), while only tiny enhancements are observable on aromatic hydrogens. This

suggests that the methyl group is on the same side of H-3 and far from the aromatic ring in position 5 of the pyrazolone ring. This is confirmed when the signal of H-2 is saturated (trace b). Strong NOE are observed on the *ortho*-hydrogens of the phenyl in position 5 of pyrazolone and on the CHO signal. These two data confirm that H-2 is close to the aromatic ring and to the CHO. Supposing the *R** configuration at the quaternary spiro-carbon, the above data assign the *S** configuration at C-2 and the *R** to C-3. When H-3 is saturated, a noticeable enhancement is also visible on H-6, i.e. the vinyl CH. This suggest that the vinyl group is on the same side of H-3. Saturation of H-3 at 3.44 ppm (trace c) shows NOE enhancement only on H-4 (the NOE on CHO is a control NOE), while no effect is observable on H-6. This confirm that H-3 and H-4 are in a *syn* relationship, implying the *S** configuration of C-4.

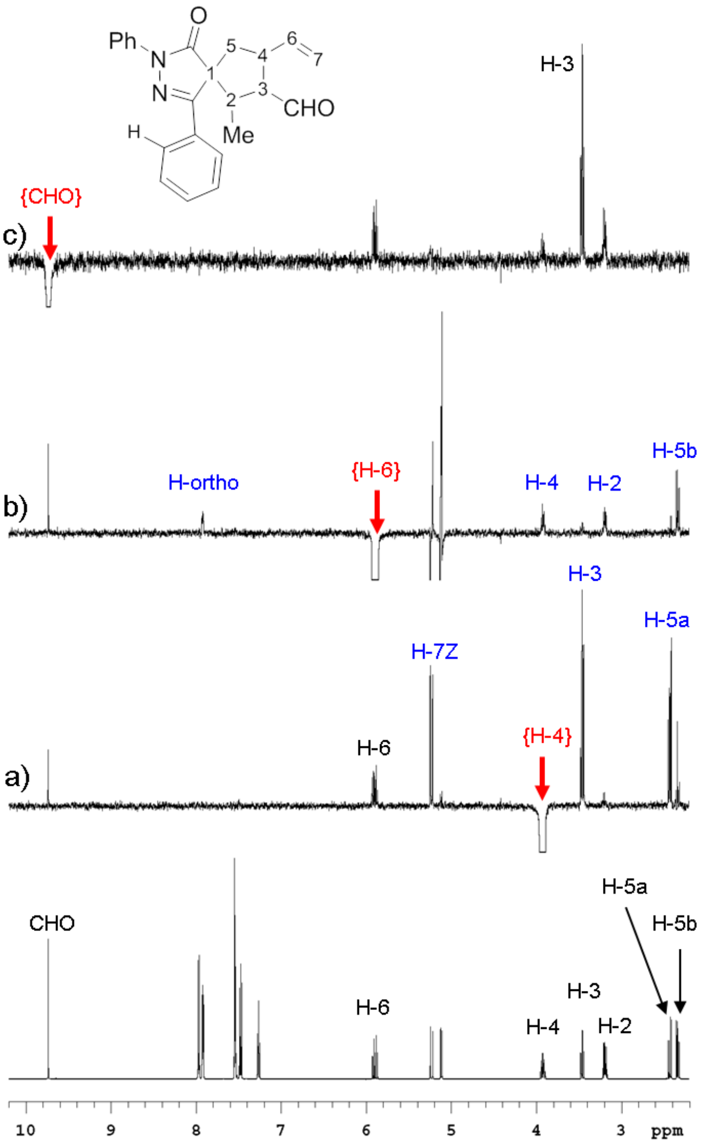

Figure S2. DPFGSE-NOE spectra of compound **3n** (600 MHz in CD_3CN)

Further NOE spectra were acquired to gain more redundancy and more information about the preferred conformation (Figure S2). When H-4 is saturated (trace a of Figure S2) a strong NOE is visible on $H7_z$, stronger that the NOE experienced by H-6. This clearly indicates that the preferred conformation of the vinyl group puts H-6 in an *anti* relationship with H-4 (dihedral angle close to 180°). It is worth to note that saturation of H-6 yields weak but similar NOEs on H-4, H-2 and on the *ortho* hydrogens of the phenyl ring. The NOE data therefore assign the 1*R**,2*S**,3*R**,4*S** relative configuration to **3n.**

Conformational analysis

Starting from the assigned relative configuration of **3n**, a complete conformational analysis was performed in order to find all the low-energy conformations. In the present case the spiro-structure is rather rigid, but it maintains some degree of conformational freedom. The all-carbon five-membered ring can assume different conformations due to its out-of-plane conformation, and the CHO and vinyl moieties can assume different orientations. A full scan of the potential energy surface (PES) was performed using molecular mechanics and the MMFF force field. All the energy minima enclosed in the lowest 10 kcal/mol range were then fully optimized using DFT calculations at the B3LYP/6-31g(d) level of theory. Frequency analysis was performed to check whether they corresponded to energy minima (no imaginary frequencies observed). After DFT optimization, four conformations were found to be enclosed in a ≈ 3 kcal/mol range (Figure S3 and Table 1). They are different because of the different relative dispositions of the CHO and vinyl moieties, with small modifications of the conformation of the aliphatic five-membered ring.

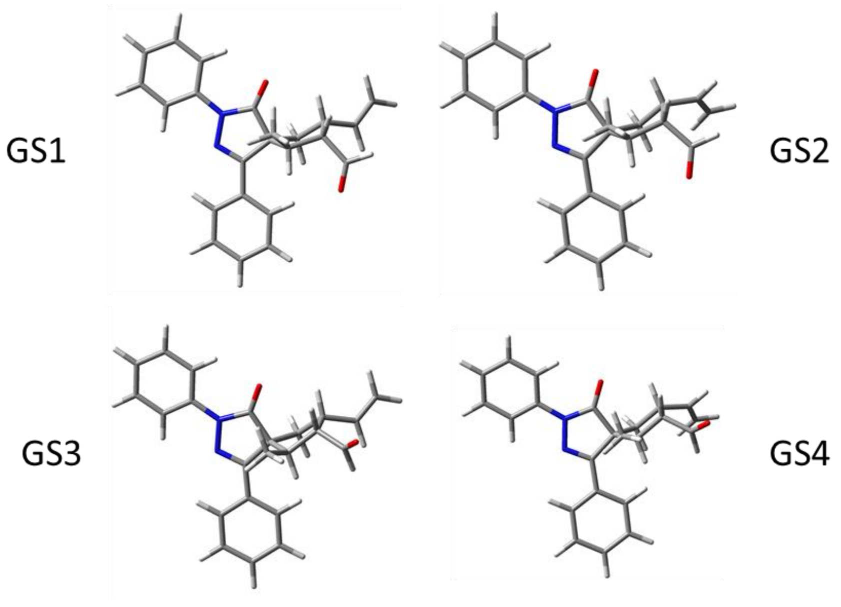

Figure S3. The four best conformations of **3n,** optimized at the B3LYP/6-31G(d) level.

Almost all the experimental NOEs well agree with the lowest energy conformation GS1. However, the NOE observed on H-6 when saturating H-2 (trace b in figure S1) suggests that the population of GS3 is not negligible, while calculations suggest a very small population (less than 1% at +25°C). DFT calculations were then run again at the B3LYP/6-31G(d) level taking into consideration the solvent (acetonitrile) using the PCM formalism.[9] The new results confirm that GS1 is the best conformation, but they also suggest a larger stabilization of GS3 (10% population) and GS4, in better agreement with the experimental NOE data.

Table S2. relative energies of the four conformations of 3m. Optimization at the B3LYP/6-31G(d) level. Values in kcal/mol

Conf.	Gas phase		PCM (acetonitrile)	
	H°	Pop(H°)	H°	Pop(H°)
GS1	**0.00**	83	**0.00**	71
GS2	1.00	15	0.80	19
GS3	2.89	1	1.32	8
GS4	3.12	<1	2.04	2

Absolute configuration

Many compounds are viscous oils and anomalous dispersion X-ray crystallography[10] is unfeasible. For this reason, the absolute configuration of compound **3n** was determined by the theoretical simulations of chiro-optical spectra. The determination of the absolute configuration (AC) of chiral molecules using chiro-optical techniques such as circular dichroism (Electronic CD and Vibrational CD) has become very reliability because of the development of theoretical methods for the prediction of these properties based on DFT (for VCD) and on Time-Dependent DFT (for ECD).[11] In the present case the theoretical calculation of the electronic circular dichroism spectra (ECD) of **3n** was selected for the absolute configuration assignment. The ECD spectrum of **3n** was acquired in HPLC-grade

[9] J. Tomasi, B. Mennucci, R. Cammi, *Chem. Rev.*, **2005**, *105*, 2999-3093.

[10] For a review see: H. D. Flack, G. Bernardinelli, *Chirality,* **2008**, *20*, 681-690

[11] For reviews see: a) G. Bringmann, T. Bruhn, K. Maksimenka, Y. Hemberger, *Eur. J. Org. Chem*. **2009**, 2717-2727. b)T. D. Crawford, M. C. Tam, M. L. Abrams, *J. Chem. Phys. A* **2007**, *111*,12057–12068. c) G. Pescitelli, L. Di Bari, N. Berova, *Chem. Soc. Rev.* **2011**, *40*, 4603-4625. For a review on conformational analysis for the absolute configuration determination see: A. Mazzanti, D. Casarini, D. *WIRES Comput. Mol. Sci.***2012**, *2*, 613-641

acetonitrile solution ($1{\cdot}10^{-4}$ M) with a cell path of 0.2 cm in the 190-400 nm region by the sum of 16 scans at 50 nm/min scan rate (Figure S4). Albeit rather weak, the experimental ECD spectrum exhibits a broad positive Cotton effect centred at 305 and 255 nm and two negative branches at 275 and 233 nm. The most intense region of the spectrum has a positive band at 215 and a negative one at 195 nm.

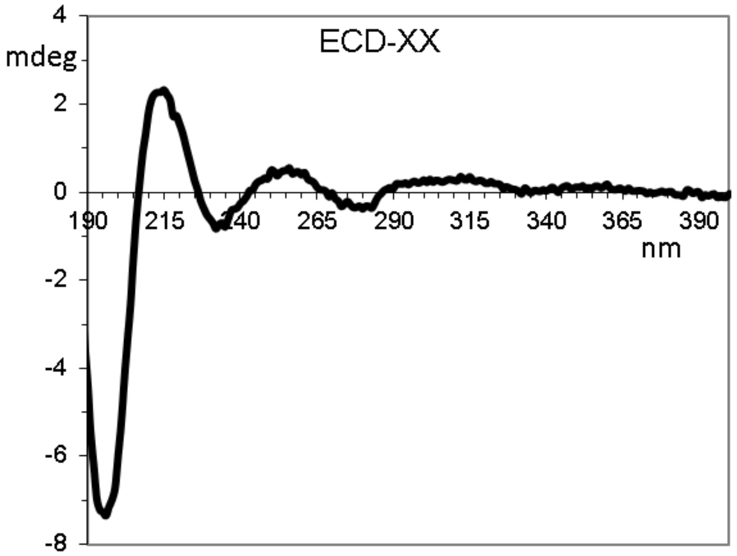

Figure S4. ECD spectrum of **3n** in acetonitrile

The TD-DFT simulations of the ECD spectra were performed using the geometries of the four conformations GS1-GS4 optimized at the PCM-B3LYP/6-31G(d) level. For data redundancy, calculations were performed with the hybrid functionals BH&HLYP[12] and M06-2X,[13] with ωB97XD that includes empirical dispersion,[14] and with CAM-B3LYP[15] that includes long range correction using the Coulomb Attenuating Method. The calculations included the contribution of the solvent using the PCM formalism, and employed the 6-311++G(2d,p), that is known to yield good performances at a reasonable computational cost.[16] The rotational strengths were calculated in both length and velocity

[12] In Gaussian 09 the BH&HLYP functional has the form: $0.5{*}E_X^{HF} + 0.5{*}E_X^{LSDA} + 0.5{*}\Delta E_X^{Becke88} + E_C^{LYP}$

[13] Y. Zhao and D.G. Truhlar, *Theor. Chem. Acc*. 2008, **120**, 215-241.

[14] J-D. Chai and M. Head-Gordon, *Phys. Chem. Chem. Phys.*, 2008, **10**, 6615-6620.

[15] T. Yanai, D. Tewand, and N.Handy, *Chem. Phys. Lett.* 2004, **393**, 51-57.

[16] a) M. Meazza, M. E. Light, A. Mazzanti and R. Rios. *Chem. Sci.* **2016**, *7*, 984; b) P. Gunasekaran, S. Perumal, J. Carlos Menéndez, M. Mancinelli, S. Ranieri, A. Mazzanti, *J. Org. Chem.* **2014**, ***79,*** 11039–11050. c) L. Caruana, M. Fochi, M. Comes Franchini, S. Ranieri, A. Mazzanti, L. Bernardi, *Chem. Commun.* **2014**, *50*, 445-447. d) M.

representation, obtaining similar results (RMS difference < 5%) that ruled out large basis set incompleteness errors (BSSE).[17] The results of the TD-DFT calculations, assuming the 1*S*,2*R*,3*S*,4*R* absolute configuration are shown in Figure S5. All the simulations suggest a broad positive band at about 290 nm, while the band at 235 nm has different sign for different conformation, being positive in GS1 and GS2 and negative for GS4 and GS4. The two pairs are different because of the conformation of the formyl moiety. The higher energy region is mainly negative in conformation GS1, GS2 and GS4, while GS3 is weaker and positive. The whole trend is well replicated by all the four functionals used.

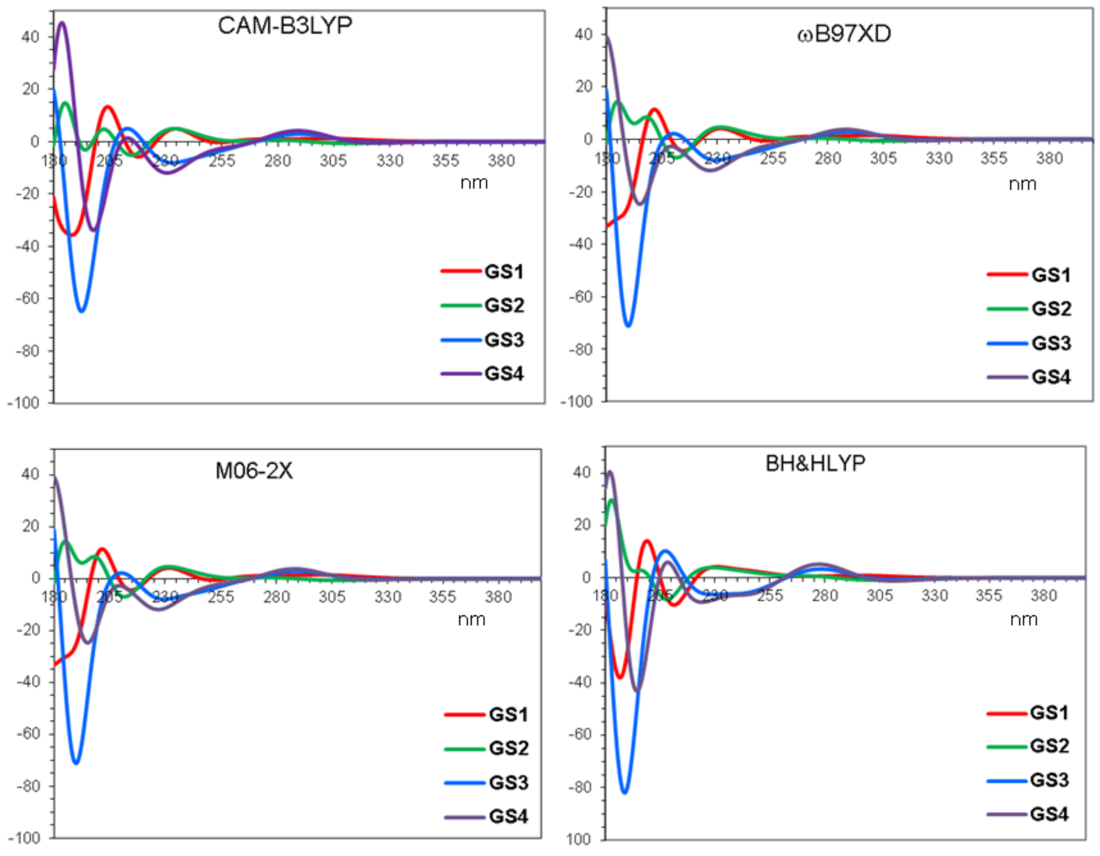

Figure S5. TD-DFT simulated spectra calculated for the four conformations of **3n** using CAM-B3LYP, BH&HLYP, M06-2X, ωB97XD and the 6-311++G(2d,p) basis set. The solvent (acetonitrile) was included with the PCM formalism. For each conformation, the first 70 excited states were calculated, and the spectrum was obtained using a 0.25 eV line width at half height.

Ambrogi, A. Ciogli, M. Mancinelli, S. Ranieri, A. Mazzanti, *J. Org. Chem.* **2013**, *78*, 3709-3719. e) L. Caruana, M. Fochi, S. Ranieri, A. Mazzanti, L. Bernardi, *Chem. Commun.* **2013**, *49*, 880-882.

[17] P.J. Stephens, D.M. McCann, F.J. Devlin, J.R. Cheeseman and M.J. Frisch, *J. Am. Chem. Soc.* 2004, **126**, 7514-7521

The simulation of the weighted spectrum was obtained by using the populations obtained from Boltzmann distribution and the relative enthalpies obtained with the PCM-optimization (Table S2, 71:19:8:2 ratio). The simulated spectra were vertically scaled and red-shifted to get the best match with the experimental spectrum (Figure S6 scaling factors: 0.25, 0.3, 0.3, 0.25; red shift: 10, 12, 12, 13 nm for CAM-B3LYP, ωB97XD, M06-2X and BH&HLYP, respectively). The red-shift was calibrated to match the most intense band at 195 nm. Regarding the shape, the four simulations are in a good agreement with the experimental spectrum. All of them correctly match the sign and sequence of the Cotton effects, but the low energy bands are slightly overestimated in energy (i.e. are simulated at shorter wavelengths). In any case, the simulations reliably assign the 1*R*, 2*S*, 3*R*, 4*S* absolute configuration to **3m**, when R-catalyst was used.

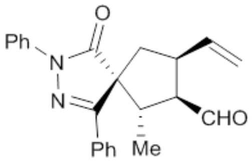

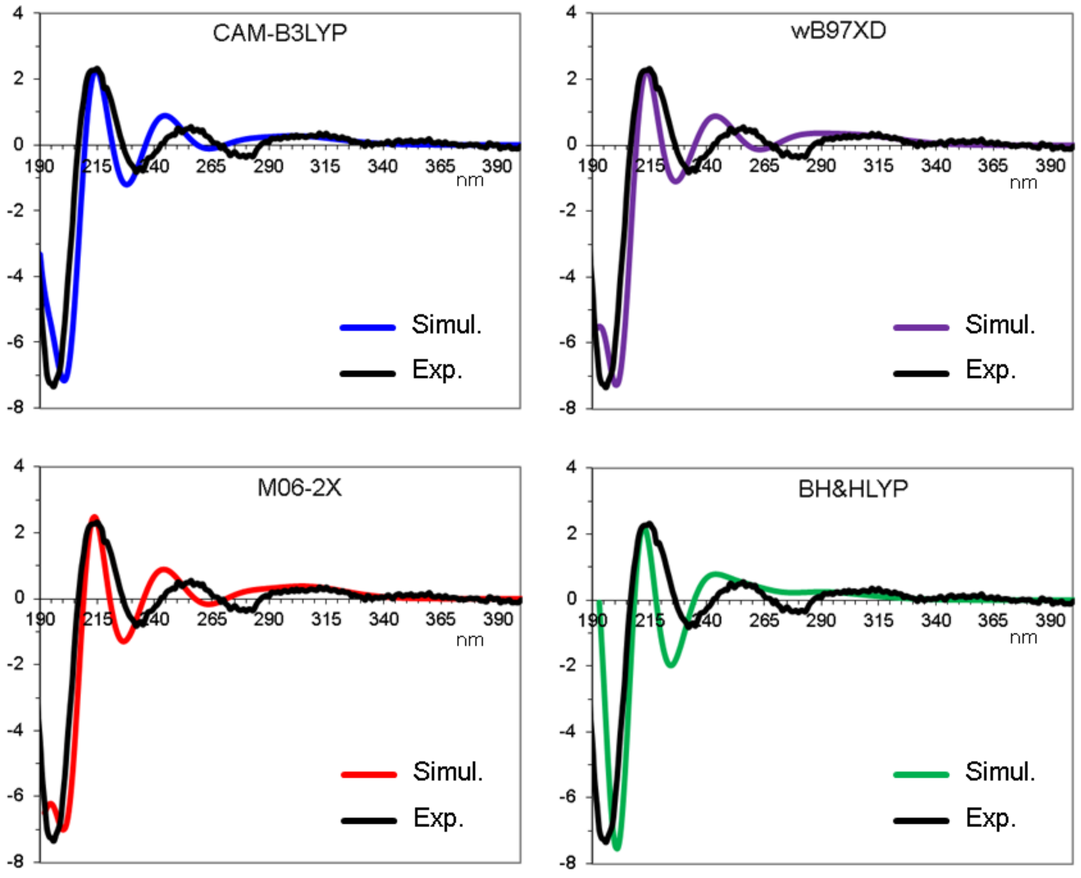

Figure S6. Simulations of the experimental ECD spectrum of **3n**. For each quadrant, the black line corresponds to the experimental spectrum. The colored lines correspond to the simulations obtained using the populations derived from PCM-B3LYP/6-31G(d) optimization.

Minor diastereomer of 3n

The relative configuration of the minor diastereomer of **3n** (hereafter **3n-minor**) was determined by NOE-NMR experiments (Figures S7 and S8).

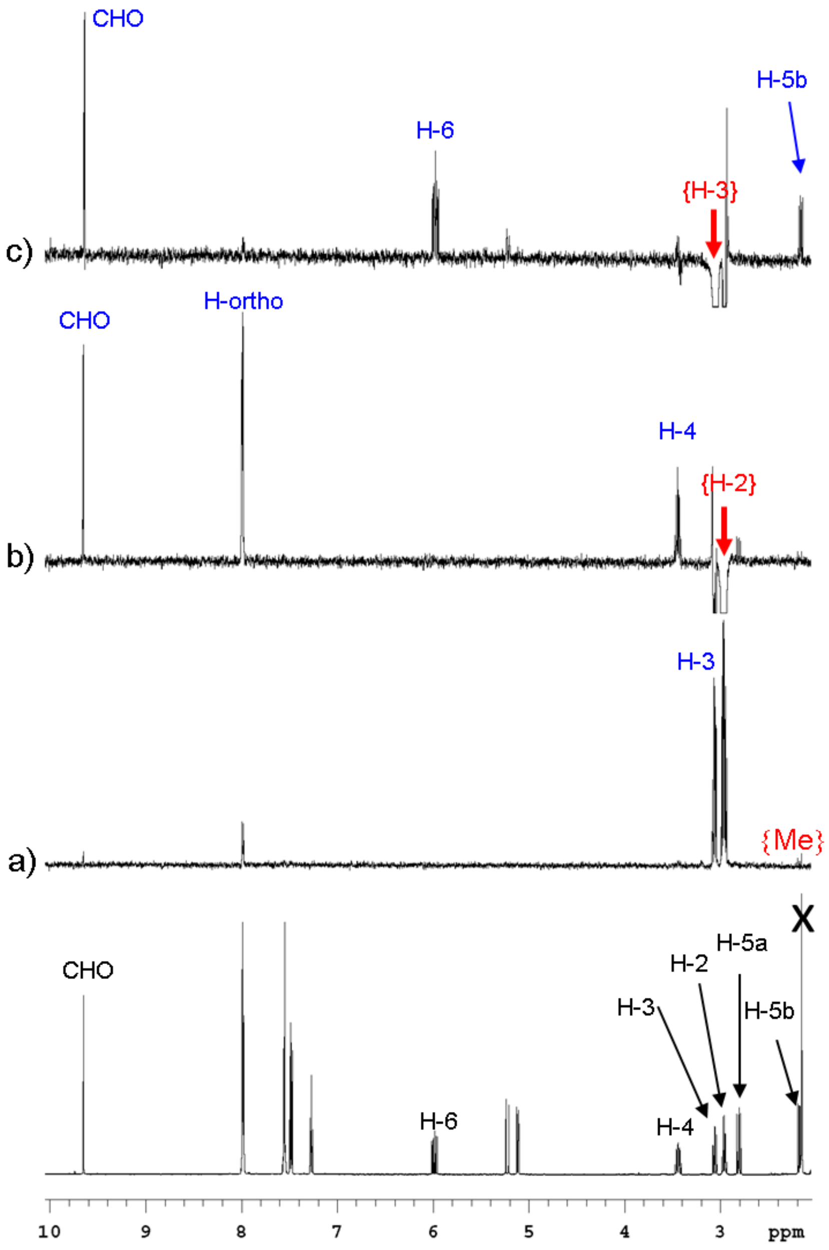

Figure S7. DPFGSE-NOE spectra of compound **3n-minor** (600 MHz in CD_3CN).

While the spectrum obtained by saturation of the 2-Me signal, is almost identical to that of the major diastereomer, the spectra obtained by saturation of H-2, H-4 and H-6 are largely different. A large NOE on H-4 was observed when H-2 was irradiated, and saturation of the H-4 signal at 3.42 ppm (trace a of Figure S7) yields a large NOE on the *ortho*-hydrogens of the phenyl ring, as well as a large NOE on H-2. These NOEs suggest that the configuration at C-4 is changed. This is confirmed when H-6

is saturated. A large NOE is visible on H-3 (trace b of Figure S8). It is therefore confirmed that the minor diastereomer of **3m** is epimer at C-4, thus implying the 1*R**,2*S**,3*R**,4*R** relative configuration.

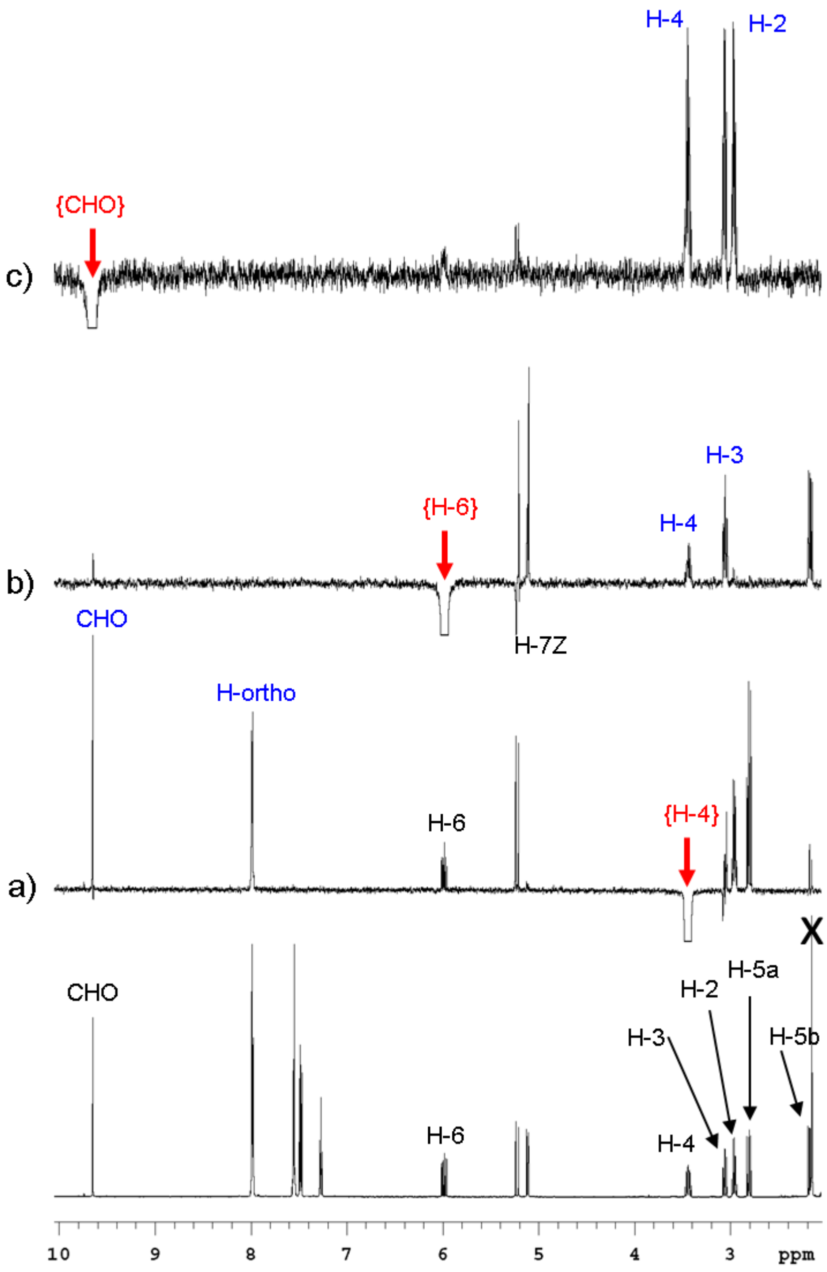

Figure S8. DPFGSE-NOE spectra of compound **3n-minor** (600 MHz in CD_3CN)

As in the case of the major diastereomer, the absolute configuration of **3n-minor** was derived from the simulation of its ECD spectrum. The ECD spectrum of **3n-minor** shows the same trend of the major diastereomer, but with different intensity of the Cotton effects (Figure S9).

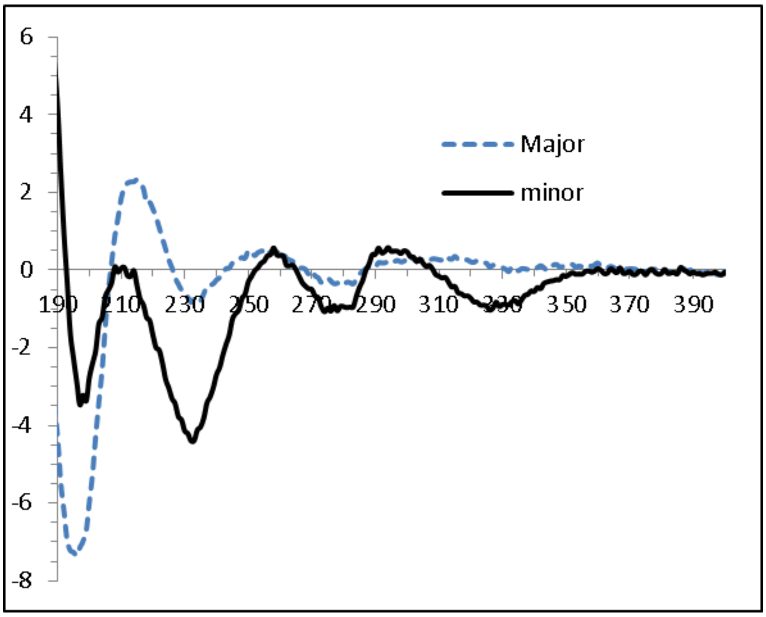

Figure S9. ECD spectrum of **3n-minor** (black trace) compared with the ECD spectrum of **3n** (dotted blue trace).

The exploration of the PES and subsequent optimization by DFT at the PCM-B3LYP/6-31G(d) level of theory suggested the existence of five conformations, comprised into a 2 kcal/mol energy window (Figure S10 and Table S3).

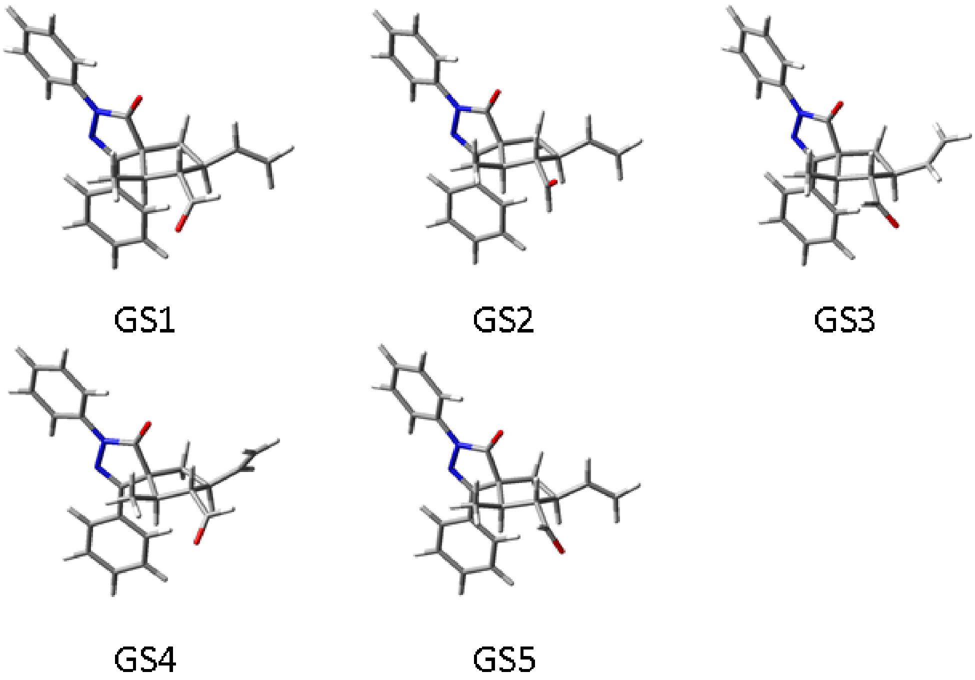

Figure S10. Optimized structures of the 5 best conformations of **3n-minor**

Table S3. Relative energies of the four conformations of 3m-minor. Optimization at the B3LYP/6-31G(d) level. Values in kcal/mol

Conf	PCM (acetonitrile)		
	E	H°	Pop(H°)
GS1	**0.00**	**0.00**	53
GS2	0.31	0.26	34
GS3	2.01	2.15	1
GS4	1.95	2.01	2
GS5	0.89	0.94	10

The simulations of the ECD spectrum were obtained assuming the 1*R*, 2*S*, 3*R*, 4*R* absolute configuration, using the same functionals and basis sets already employed for the major diastereomer.

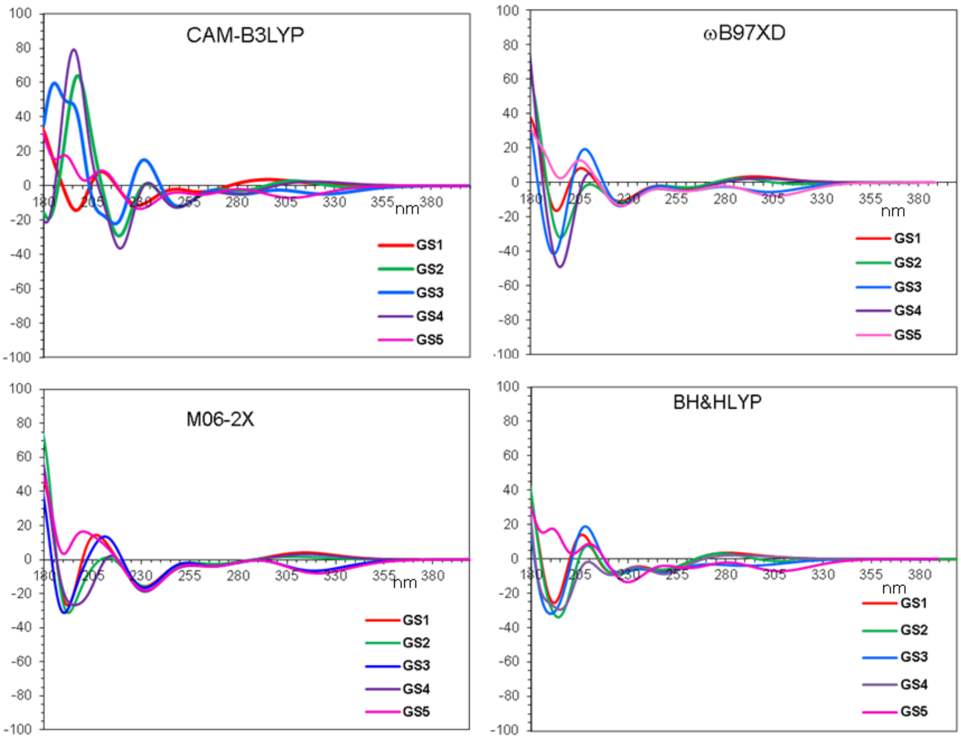

Figure S11. TD-DFT simulated spectra calculated for the four conformations of **3n-minor** using CAM-B3LYP, BH&HLYP, M06-2X, ωB97XD and the 6-311++G(2d,p) basis set. Solvent acetonitrile was included with the PCM formalism. For each conformation the first 70 excited states were calculated, and the spectrum was obtained using a 0.25 eV line width at half height.

The relative population to be used in the simulation of the experimental ECD was derived from the relative enthalpies of optimized geometries (53:31:2:2:12 for GS1-GS5, respectively). The simulations for 1*R*, 2*S*, 3*R*, 4*R* absolute configuration (figure S11 and S12) show a good agreement with the experimental spectrum, particularly when using the CAM-B3LYP and ωB97XD functionals.

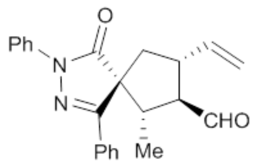

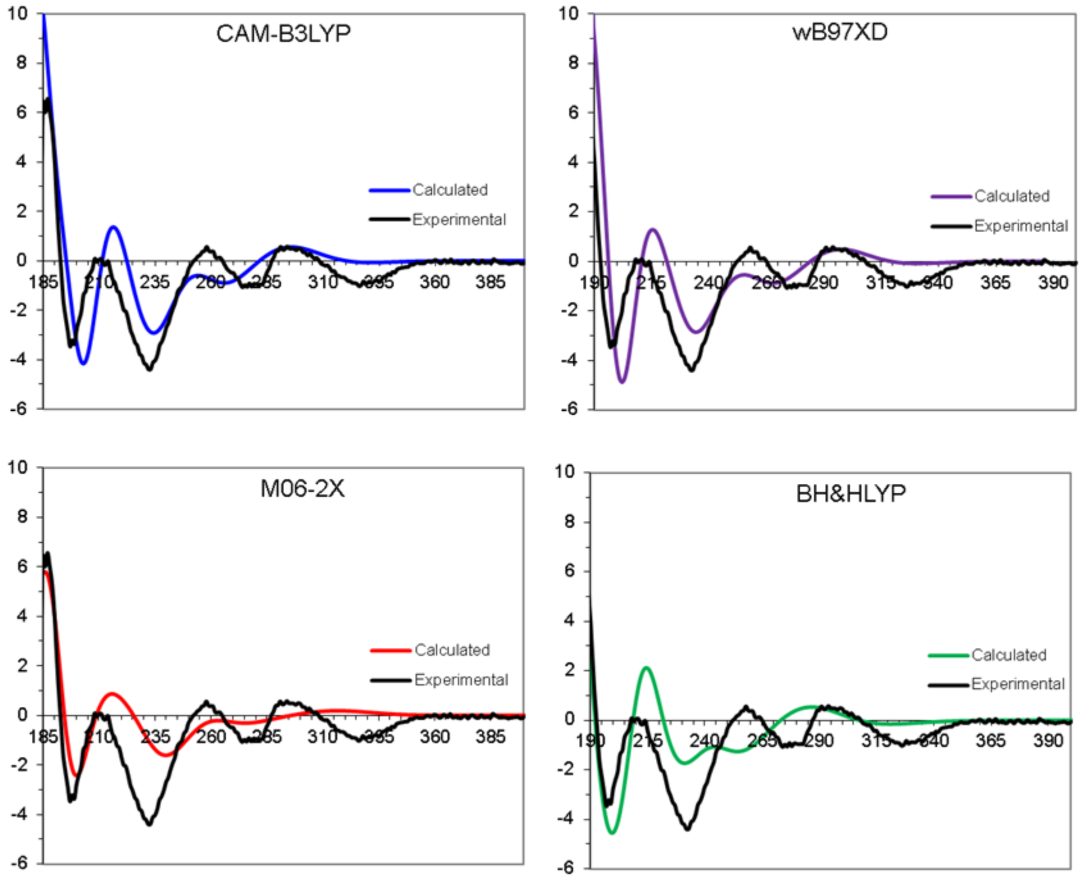

Figure S12. Simulations of the experimental ECD spectrum of **3n-minor**. For each quadrant, the black line corresponds to the experimental spectrum. The colored lines correspond to the simulations obtained using the populations derived from PCM-B3LYP/6-31G(d) optimization.

2-Phenyl compound (3g)

The relative configuration of compound **3g** was derived from NOE-NMR as in the case of **3n** and **3n-minor**. The major diastereomer was purified by reverse-phase HPLC on C18 column. All the ^{1}H and ^{13}C chemical shifts were assigned by 2D-NMR spectroscopy (COSY, HSQC and HMBC). Apart from the different chemicals shifts, the ^{1}H region of the spectrum including H-2,H-3 and H-4 (Figure S13) is very similar to that of the major diastereomer of **3n**, and the coupling constant are very similar too.

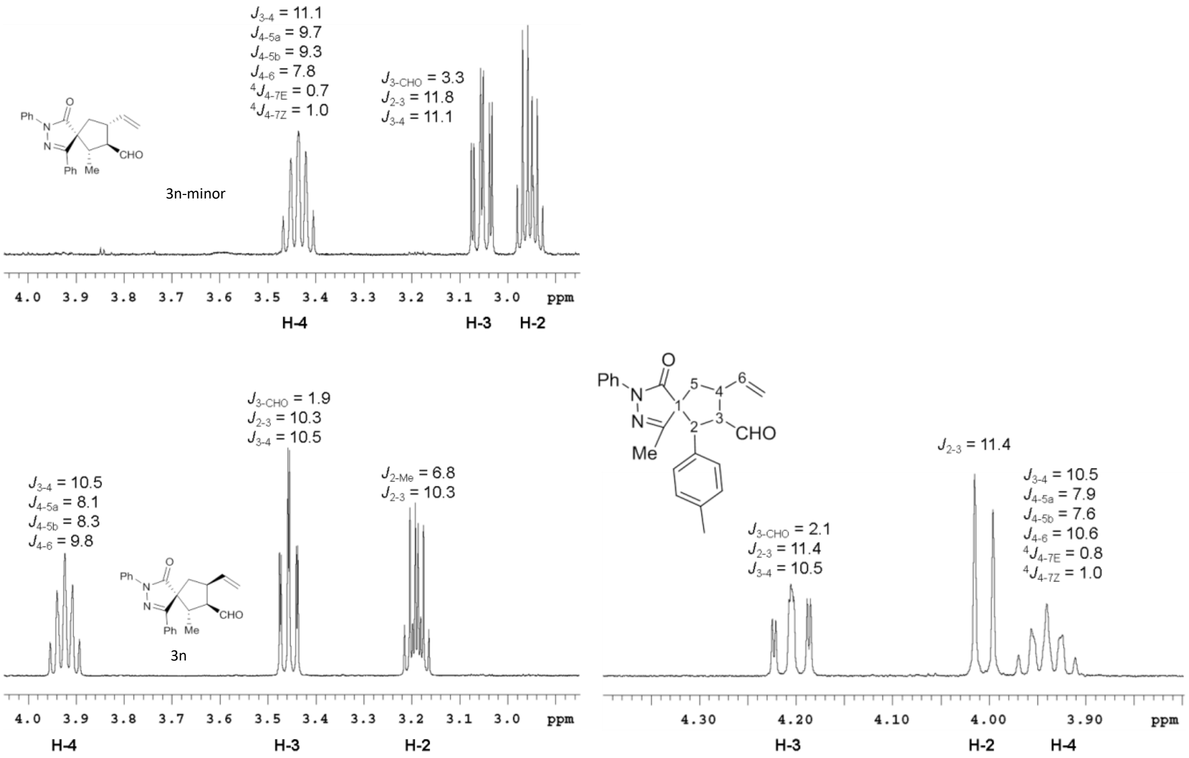

Figure S13: Left: portion of the ^{1}H spectrum of **3n and 3n-minor.** Right: portion of the ^{1}H spectrum of **3g** (600 MHz in CD_3CN).

On saturation of the methyl signal of pyrazolone (hereafter methyl) a strong NOE was observed on H-2 (trace a of Figure S14), but a noticeable NOE was observed also on the *ortho* hydrogens of the phenyl in position 2. The spatial proximity between the Methyl and H-2 was confirmed by the saturation of H-2 (trace c of Figure S13), where the *ortho* hydrogens of the phenyl act as a control signal. In the same spectrum an intense NOE is visible also on H-6. This implies that the vinyl group is on the same side of H-2 with respect to the cycle. Saturation of H-3 (trace a in Figure S15) yields strong NOEs on the CHO (control signal), on the *ortho* hydrogens and on H-4. This means that H-3 and H-4 are on the same side of the cycle and on the opposite side of H-2. The above consideration suggests the 1*R**,2*R**,3*R**,4*S**. It should be noted that the actual spatial disposition of the substituents is the same observed for the major diastereomer of **3n**, and that the different

stereochemical descriptor at C-2 is due to the higher priority of the phenyl group of **3g** with respect to the methyl in compound **3m**.

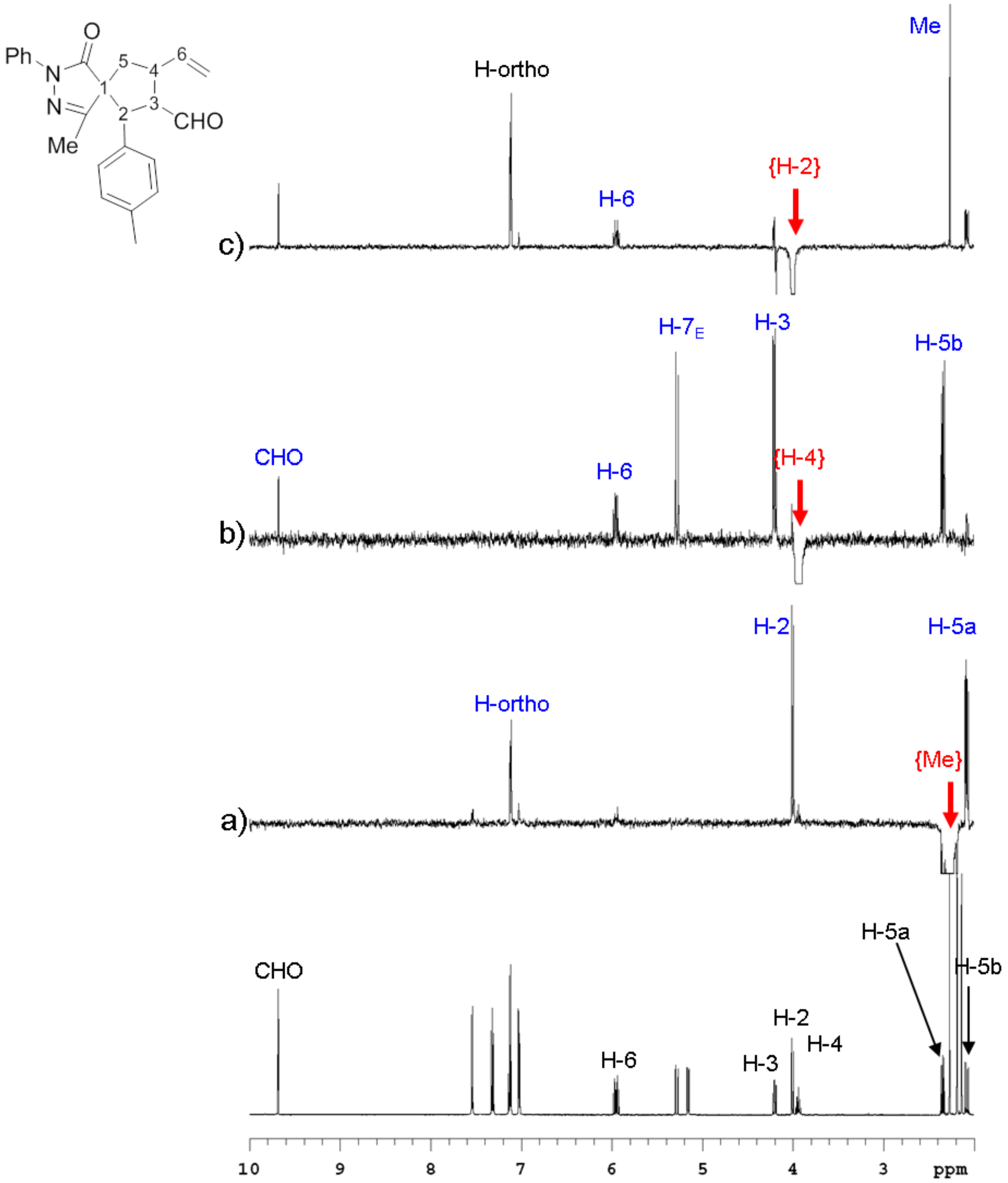

Figure S14. DPFGSE NOE spectra of **3g** (600 MHz in CD_3CN). Bottom: control spectrum. Traces a-c: NOE spectra obtained on saturation of Methyl, H-4 and H-2.

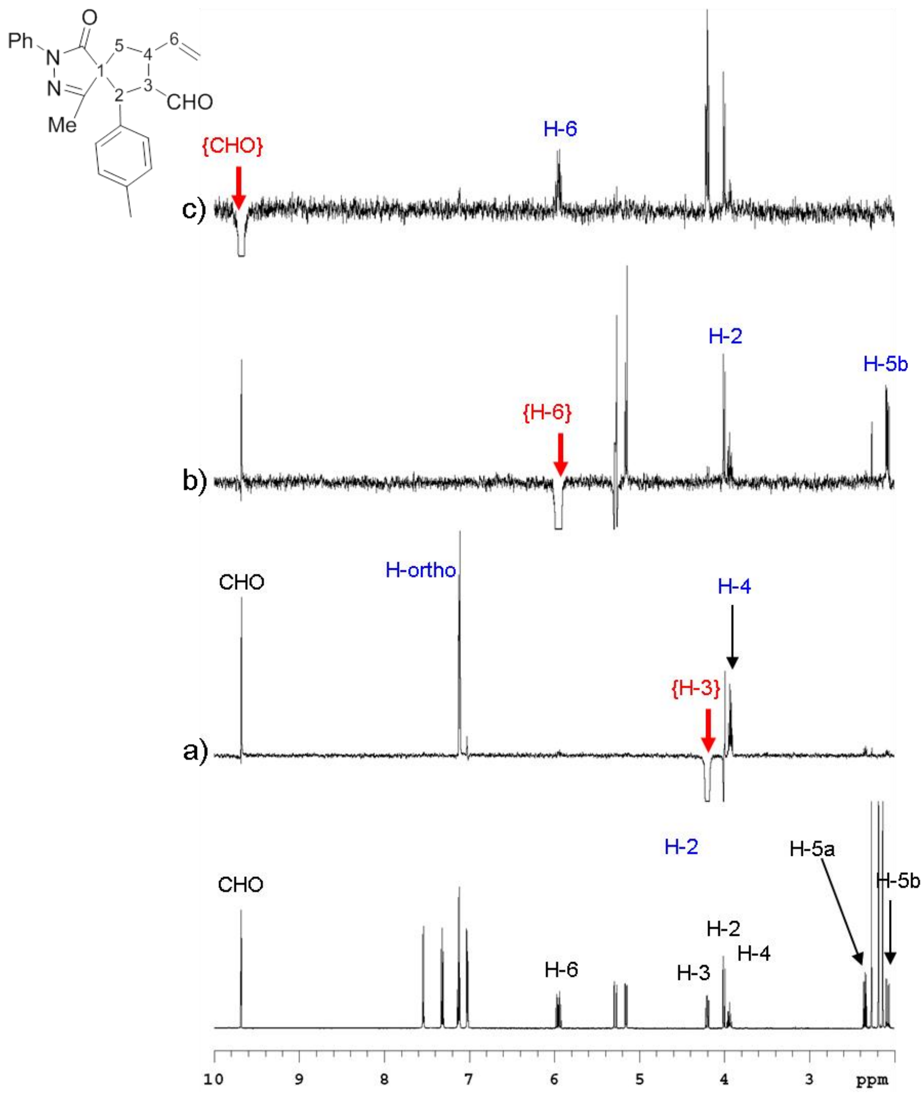

Figure S15. DPFGSE NOE spectra of **3g** (600 MHz in CD_3CN). Bottom: control spectrum. Traces a-c: NOE spectra obtained on saturation of H-3, H-6 and CHO.

Conformational analysis of **3g** yielded again four conformations comprised into a 2 kcal/mol energy window (Table S4 and Figure S16). As for **3n**, calculations were run at the B3LYP/6-31G(d) level of theory and including the solvent acetonitrile using the PCM formalism.

Table S4. Relative energies of the four conformations of 3g. Optimization at the B3LYP/6-31G(d) level. Values in kcal/mol.

Conf	PCM (acetonitrile)		
	E	H°	Pop(H°)
GS1	**78**	**0.00**	69
GS2	10	0.65	23
GS3	9	1.44	6
GS4	3	2.02	2

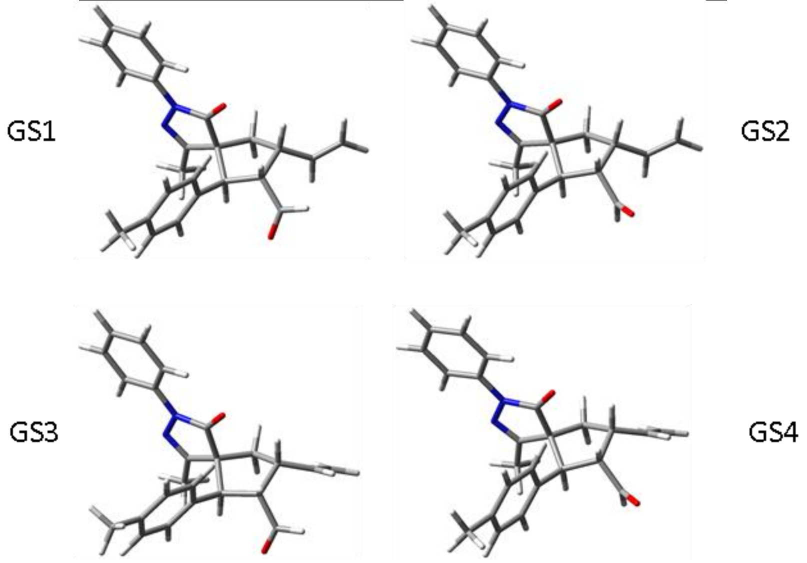

Figure S16. 3D optimized geometries of the four conformations of **3g**

The ECD spectrum of **3g** was acquired in acetonitrile as in the previous cases. Due to the presence of the phenyl in position 2, that is tilted out of the plane of pyrazolone, the ECD spectrum of **3g** is more intense, showing two strong bands at 240 and 202 nm (Figure S17).

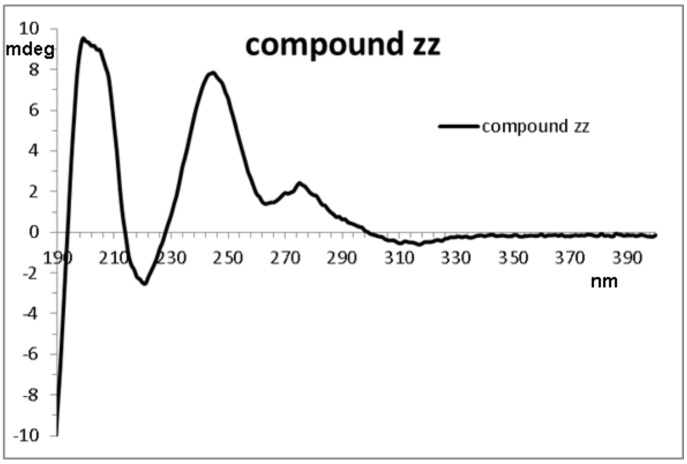

Figure S17. Experimental ECD spectrum of **3g** in acetonitrile

TD-DFT calculation of the ECD spectra of the four conformations were obtained using CAM-B3LYP, BH&HLYP, M06-2X, ωB97XD and the 6-311++G(2d,p) basis set, and the solvent was included using PCM (Figure S17). Compound **3g** was obtained with the pseudoenantiomeric *S*-catalyst, thus the simulations were run supposing the 1*S*, 2*S*, 3*S*, 4*R* absolute configuration. The simulation of the experimental ECD spectrum was obtained using the population ratio derived from Boltzmann distribution and the relative energies from Table 3 (69:23:6:2 for GS1-GS4, respectively). The weighted spectra reproduce very well the experimental ones, in particular when M06-2X was used (Figure S18 and S19).

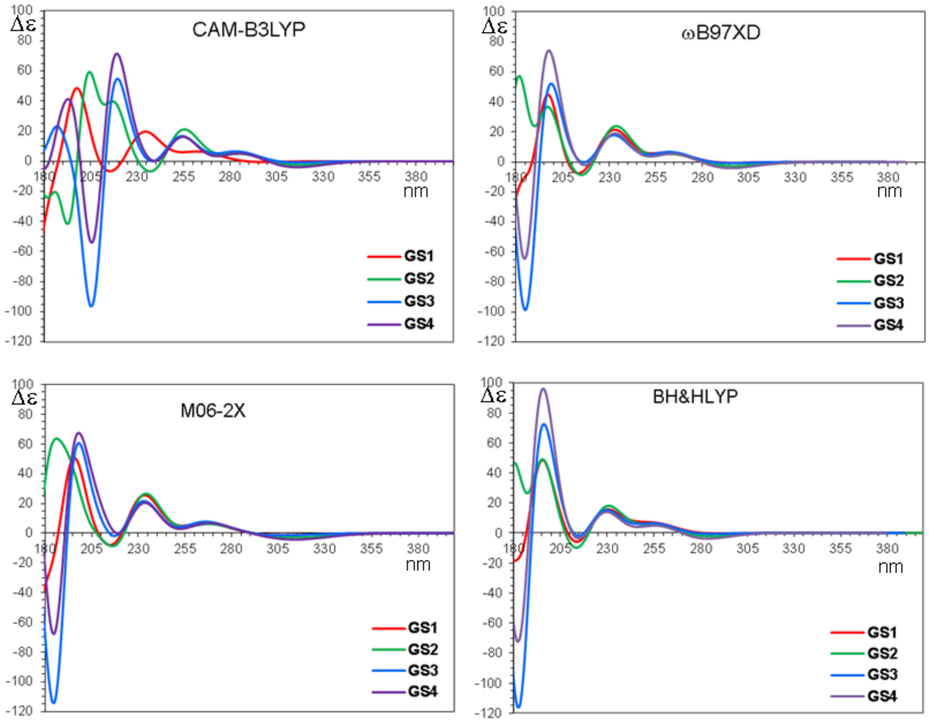

Figure S18. Top: TD-DFT simulated spectra calculated for the four conformations of **3g** using CAM-B3LYP, BH&HLYP, M06-2X, ωB97XD and the 6-311++G(2d,p) basis set. Solvent acetonitrile was included with the PCM formalism. For each conformation the first 70 excited states were calculated, and the spectrum was obtained using a 0.25 eV line width at half height.

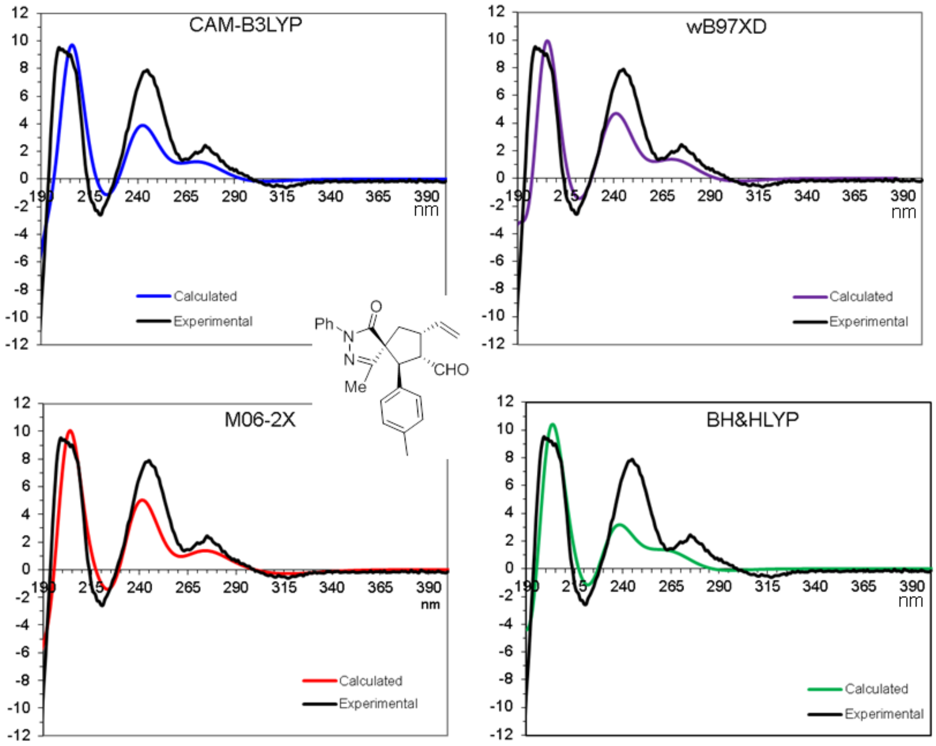

Figure S19. Simulations of the experimental ECD spectrum of **3g-major**. For each quadrant, the black line corresponds to the experimental spectrum. The colored lines correspond to the simulations obtained using the populations derived from PCM-B3LYP/6-31G(d) optimization.

7. NMR spectra starting material

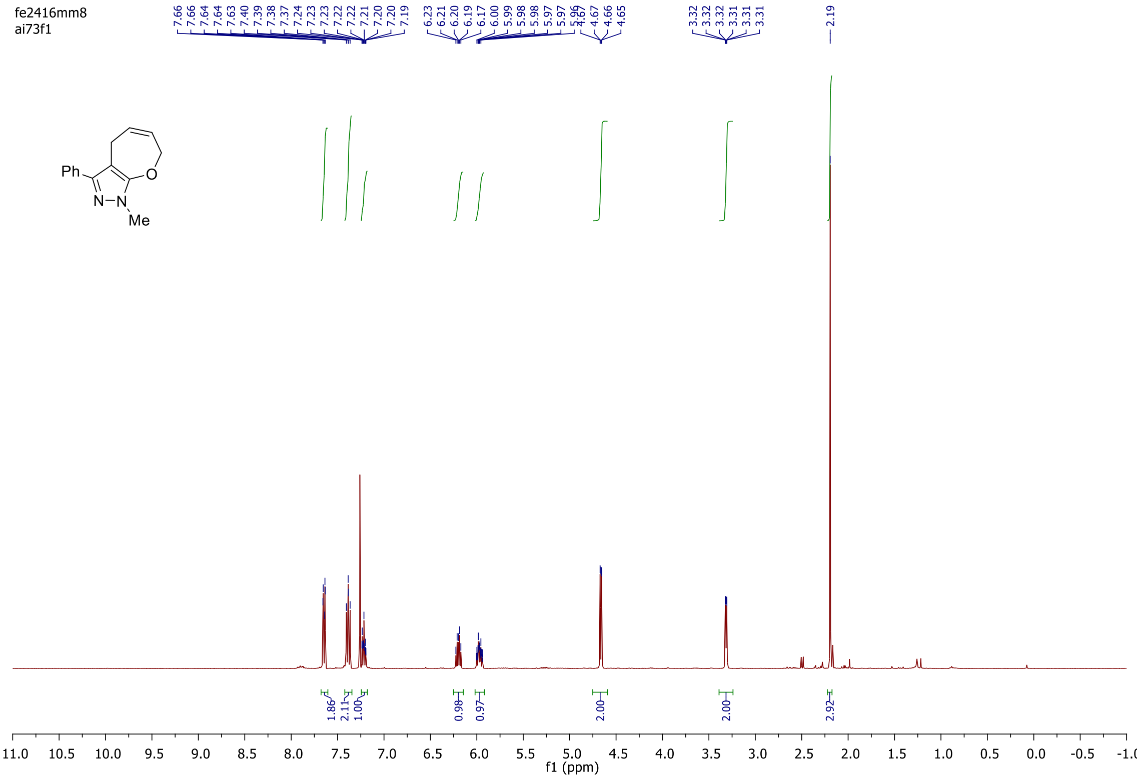

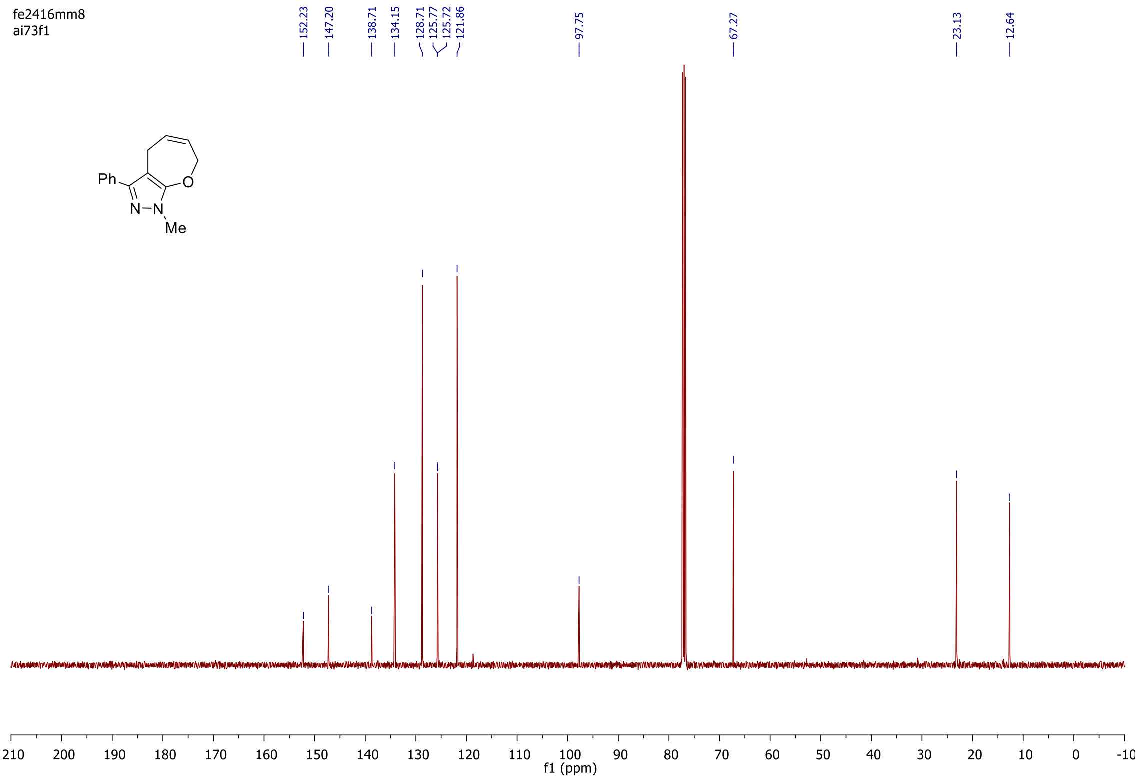

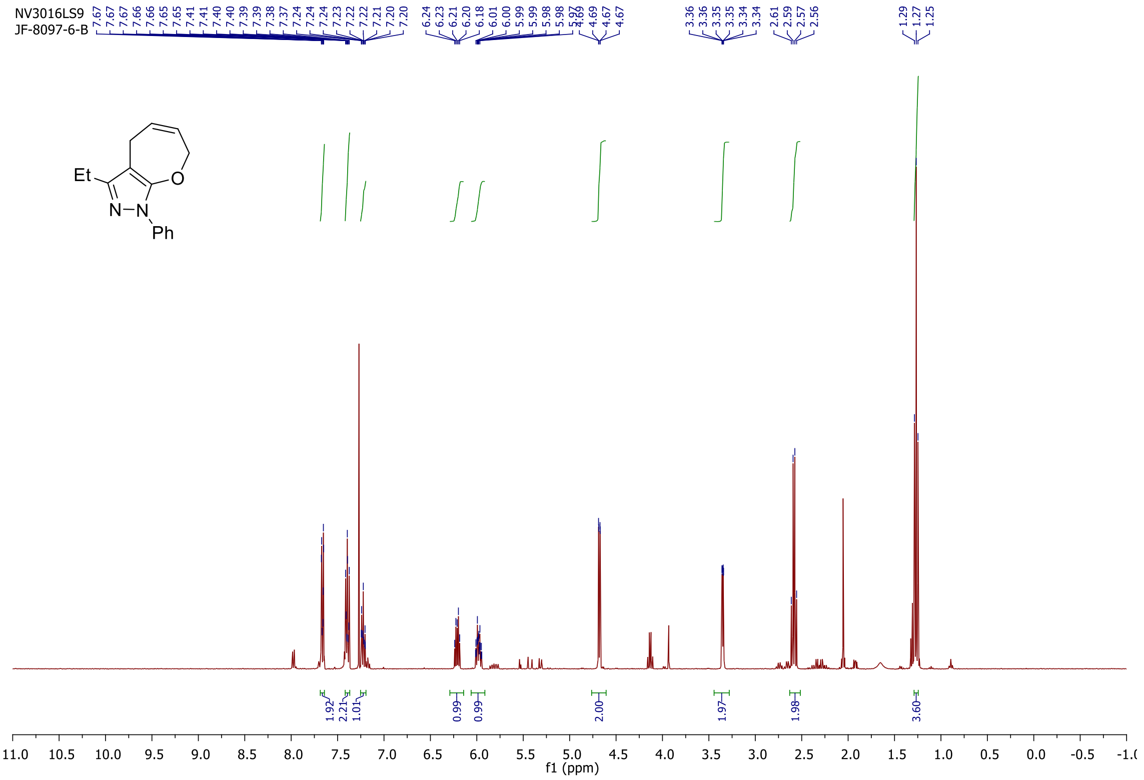
NV3016LS9
JF-8097-6-B
7.67 7.67 7.67 7.66 7.66 7.65 7.65 7.41 7.41 7.40 7.40 7.39 7.39 7.38 7.37 7.24 7.24 7.24 7.23 7.22 7.22 7.21 7.20 7.20
6.24 6.23 6.21 6.20 6.18 6.01 6.00 5.99 5.99 5.98 5.98 5.97 4.69 4.67 4.67
3.36 3.36 3.35 3.35 3.34 3.34 2.61 2.59 2.57 2.56
1.29 1.27 1.25
Et
N
N
O
Ph
1.92 2.21 1.01 0.99 0.99 2.00 1.97 1.98 3.60
11.0 10.5 10.0 9.5 9.0 8.5 8.0 7.5 7.0 6.5 6.0 5.5 5.0 4.5 4.0 3.5 3.0 2.5 2.0 1.5 1.0 0.5 0.0 -0.5
f1 (ppm)

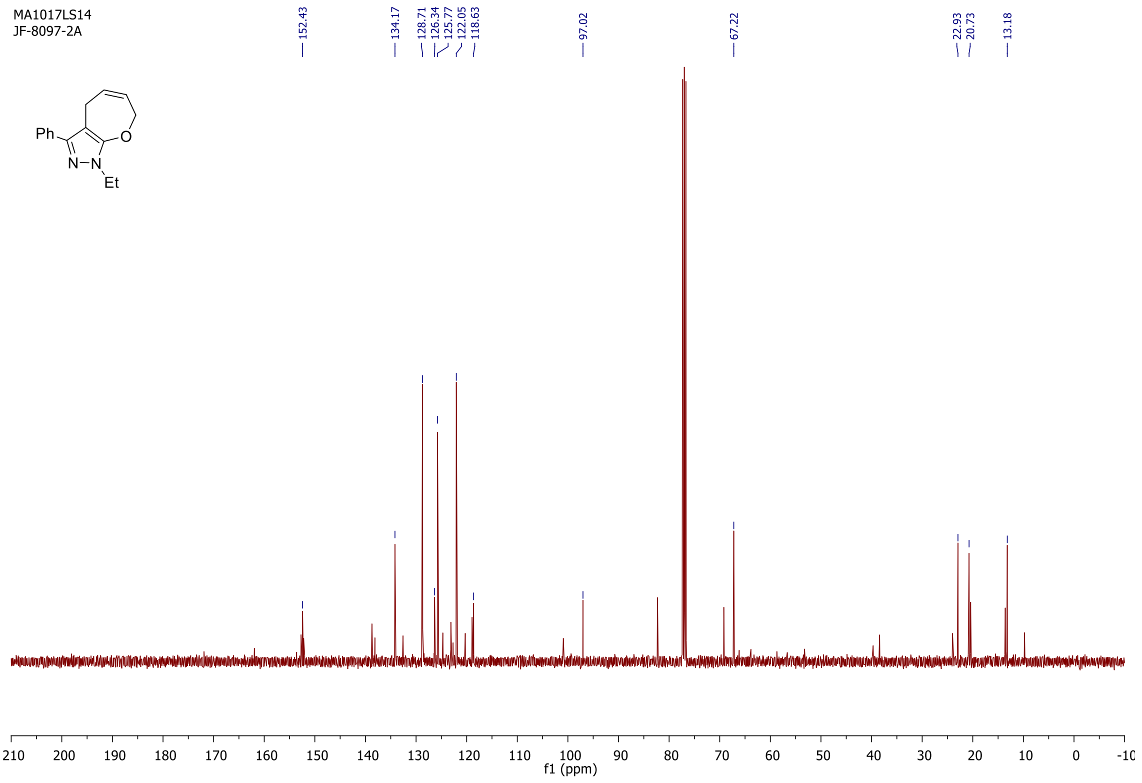
MA1017LS14
JF-8097-2A
152.43
134.17 128.71 126.34 125.77 122.05 118.63
97.02
67.22
22.93 20.73
13.18
Ph
N
N
O
Et
210 200 190 180 170 160 150 140 130 120 110 100 90 80 70 60 50 40 30 20 10 0
f1 (ppm)

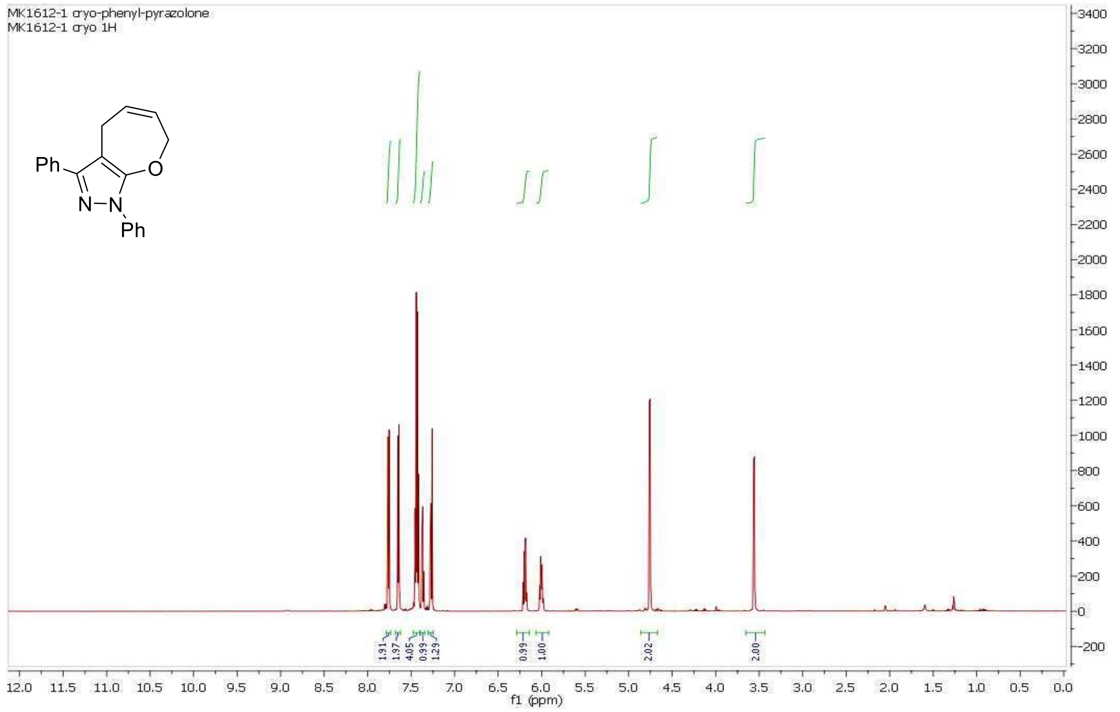
MK1612-1 cryo-phenyl-pyrazolone
MK1612-1 cryo 1H
Ph
N
N
O
Ph
1.91
1.97
4.05
0.99
1.29
0.99
1.00
2.02
2.00
f1 (ppm)

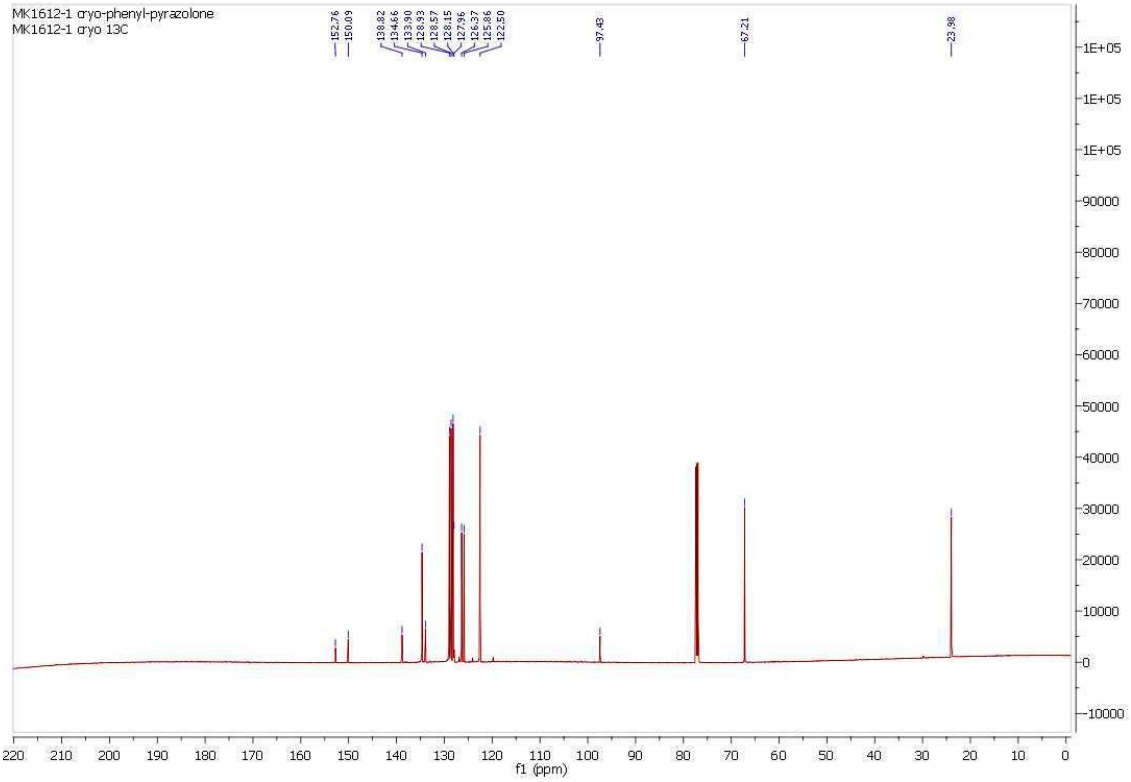
MK1612-1 cryo-phenyl-pyrazolone
MK1612-1 cryo 13C
152.76
150.09
138.82
134.66
133.90
128.93
128.57
128.15
127.96
126.37
125.86
122.50
97.43
67.21
23.98
f1 (ppm)

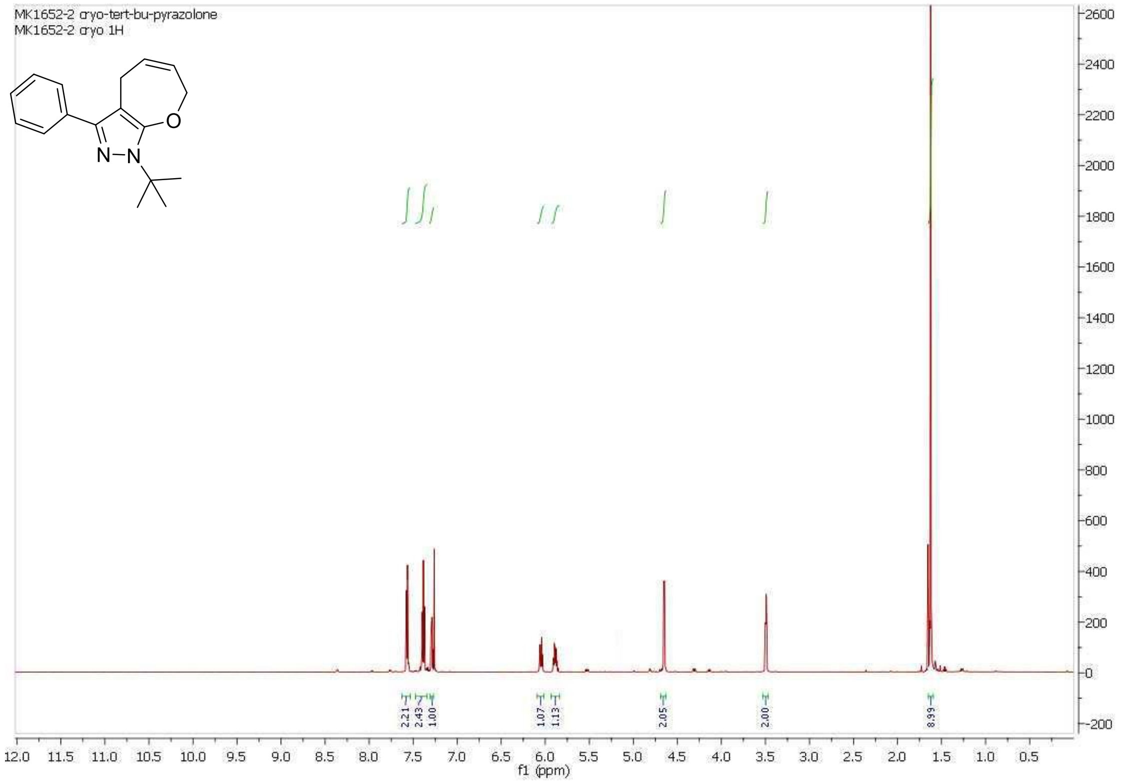
MK1652-2 cryo-tert-bu-pyrazolone
MK1652-2 cryo 1H
N
N
O
2.21
2.43
1.00
1.07
1.13
2.05
2.00
8.99
f1 (ppm)

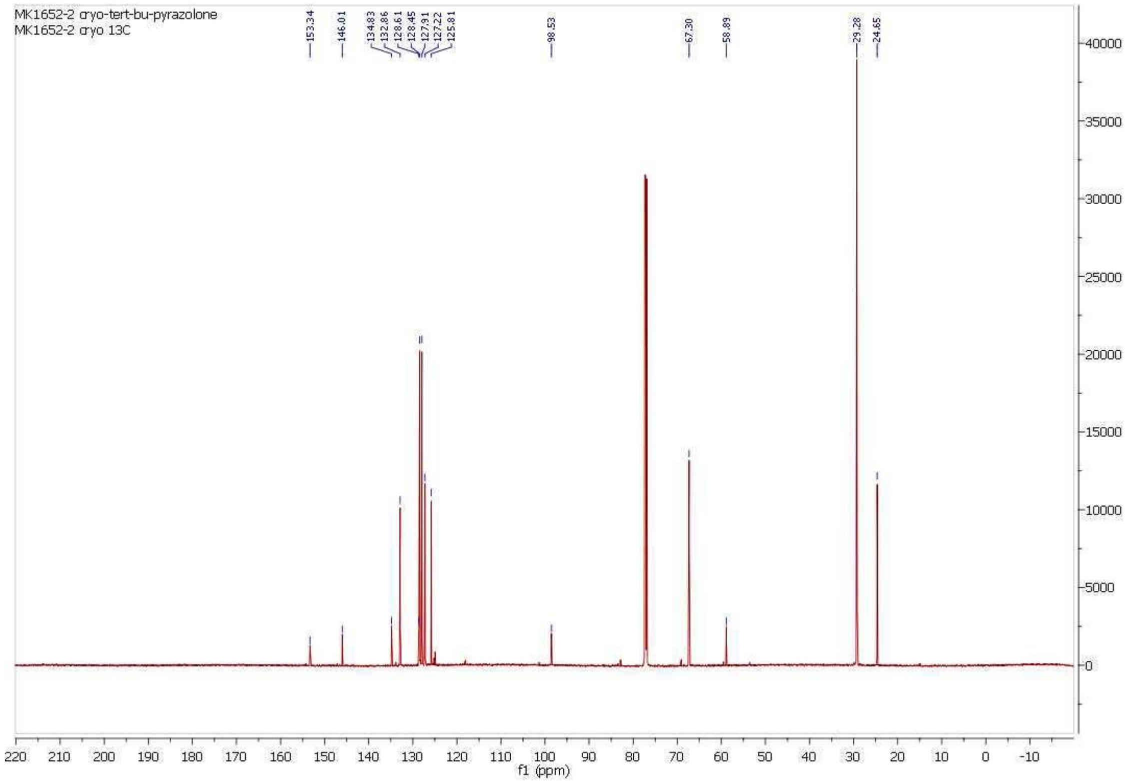
MK1652-2 cryo-tert-bu-pyrazolone
MK1652-2 cryo 13C
153.34
146.01
134.83
132.86
128.61
128.45
127.91
127.22
125.81
98.53
67.30
58.89
29.28
24.65
f1 (ppm)

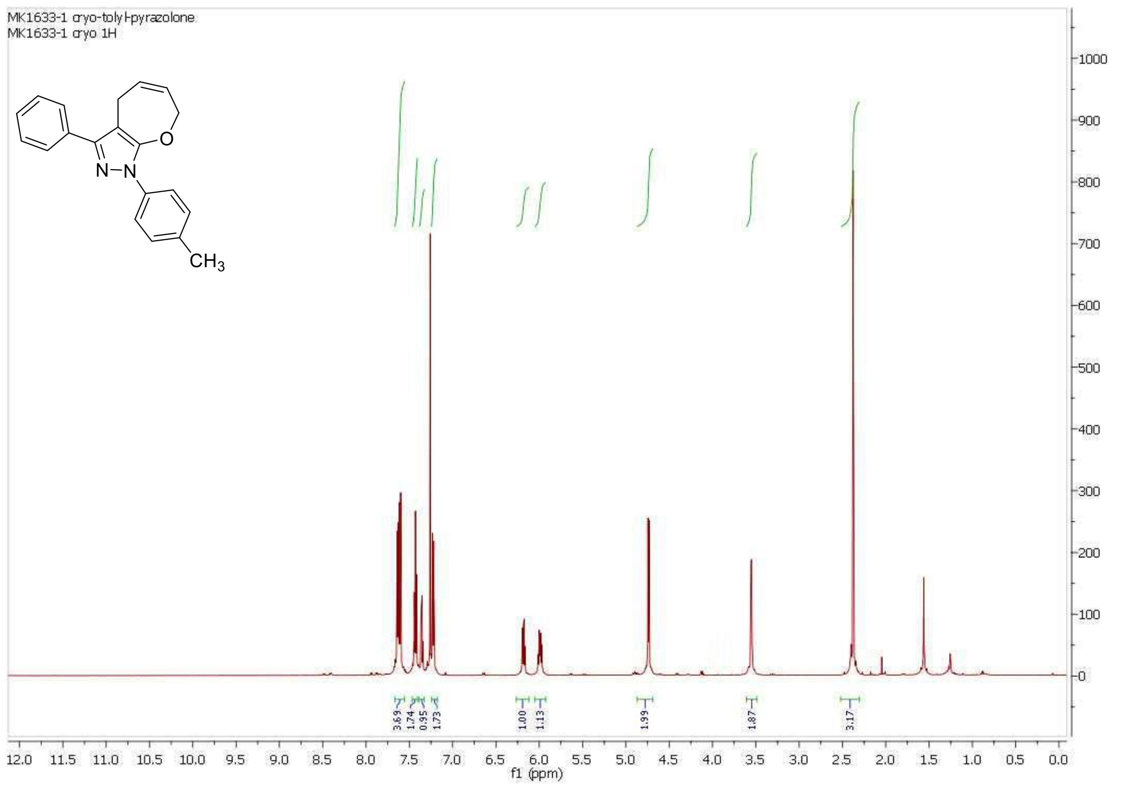
MK1633-1 cryo-tolyl-pyrazolone
MK1633-1 cryo 1H
N
N
O
CH3
3.69
1.74
0.95
1.73
1.00
1.13
1.99
1.87
3.17
f1 (ppm)

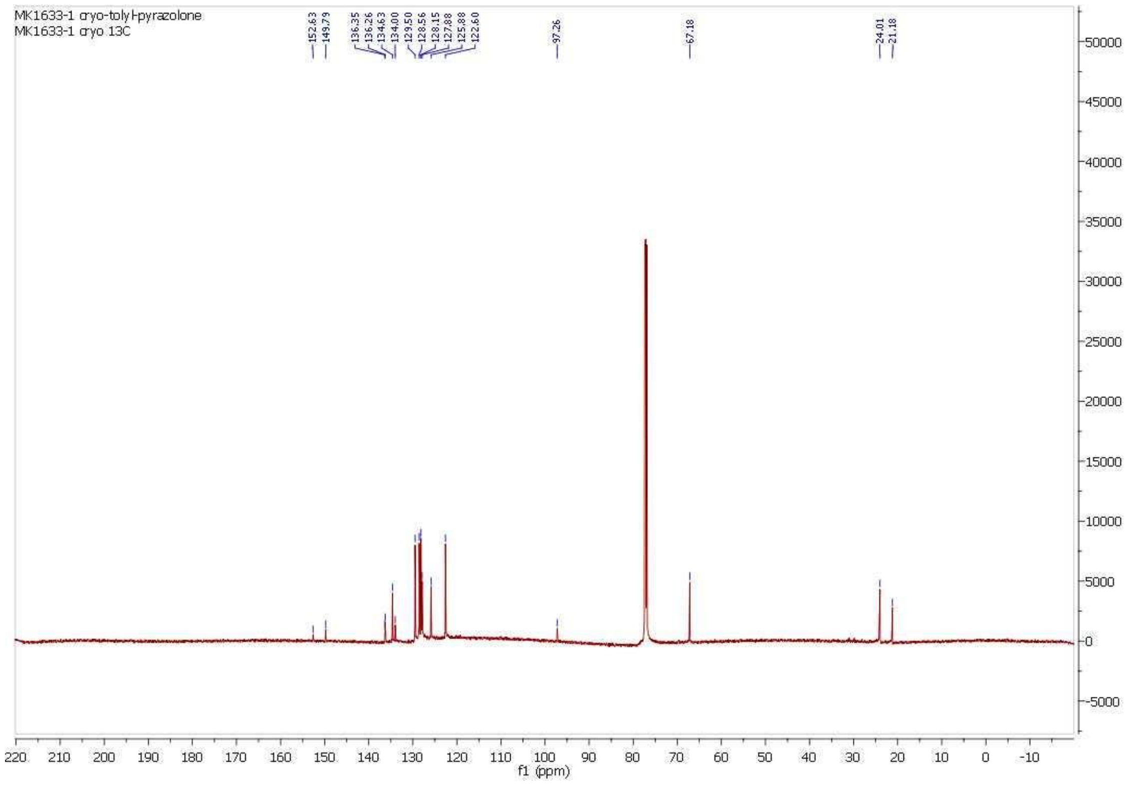
MK1633-1 cryo-tolyl-pyrazolone
MK1633-1 cryo 13C
152.63
149.79
136.35
136.26
134.63
134.00
129.50
128.56
128.15
127.88
125.88
122.60
97.26
67.18
24.01
21.18
f1 (ppm)

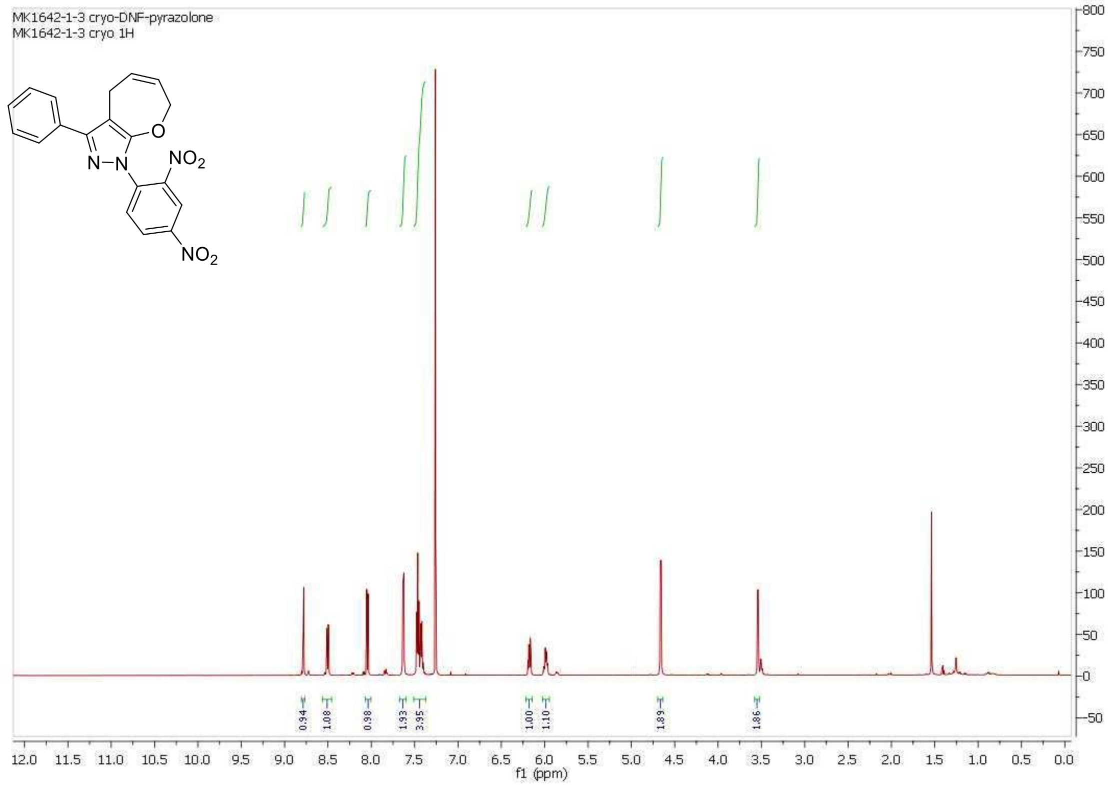
MK1642-1-3 cryo-DNF-pyrazolone
MK1642-1-3 cryo 1H
NO2
NO2
O
N
N
0.94
1.08
0.98
1.93
3.95
1.00
1.10
1.89
1.86
f1 (ppm)

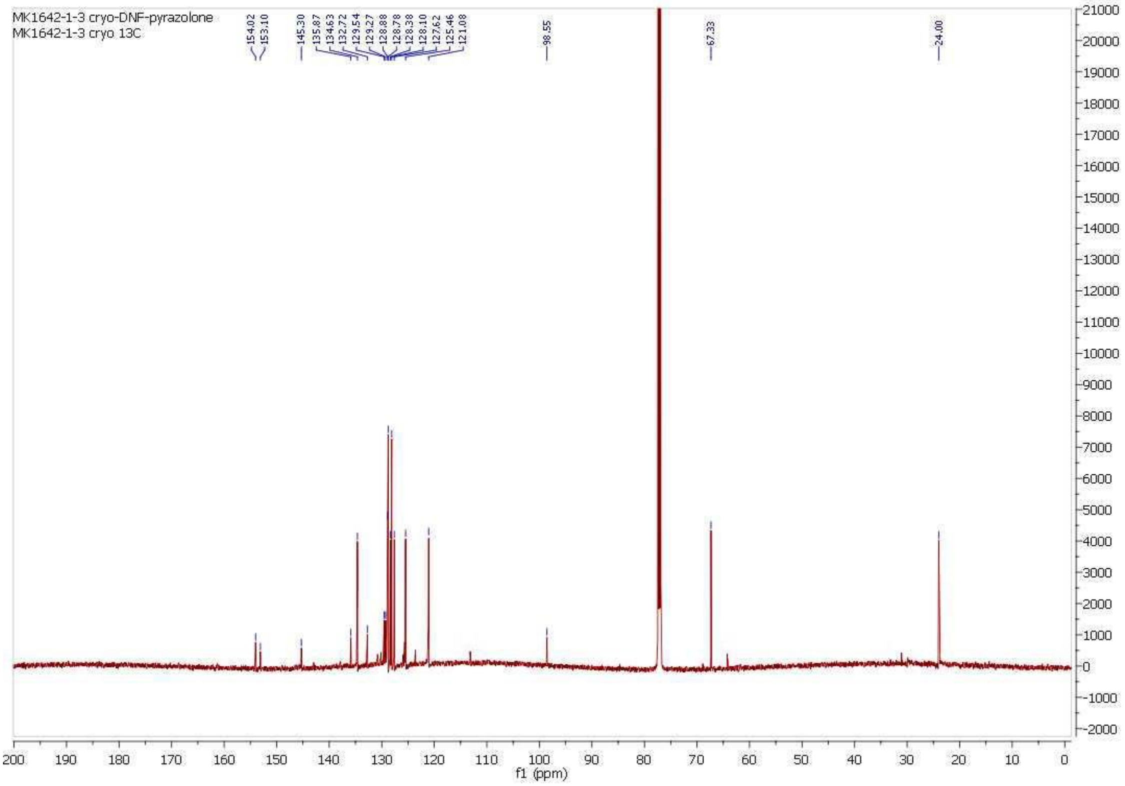
MK1642-1-3 cryo-DNF-pyrazolone
MK1642-1-3 cryo 13C
154.02
153.10
145.30
135.87
134.63
132.72
129.54
129.27
128.88
128.78
128.38
128.10
127.62
125.46
121.08
98.55
67.33
24.00
f1 (ppm)

8. NMR spectra spiro compounds

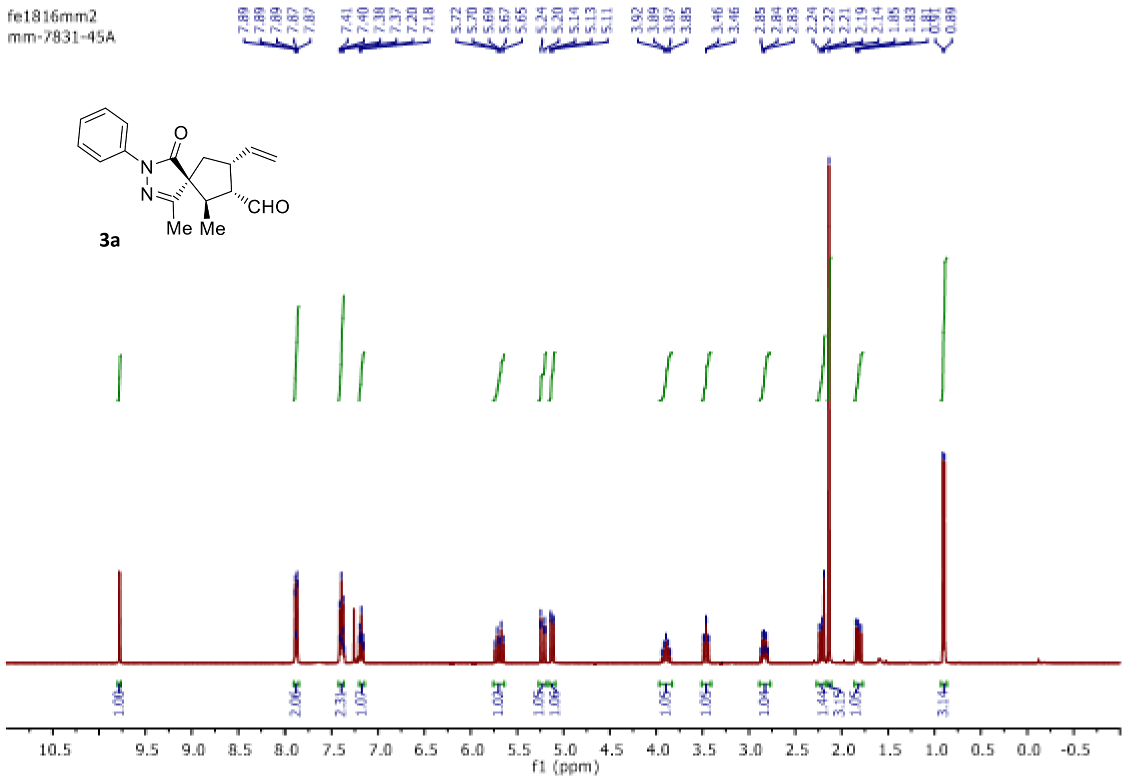

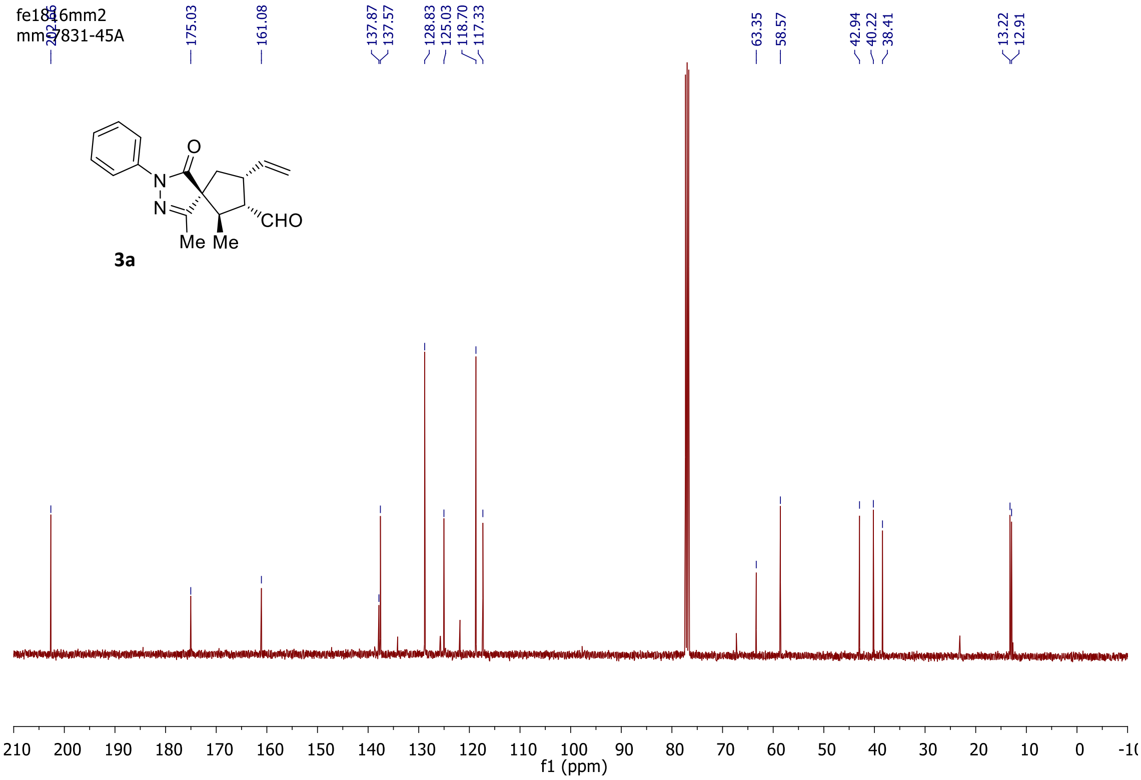

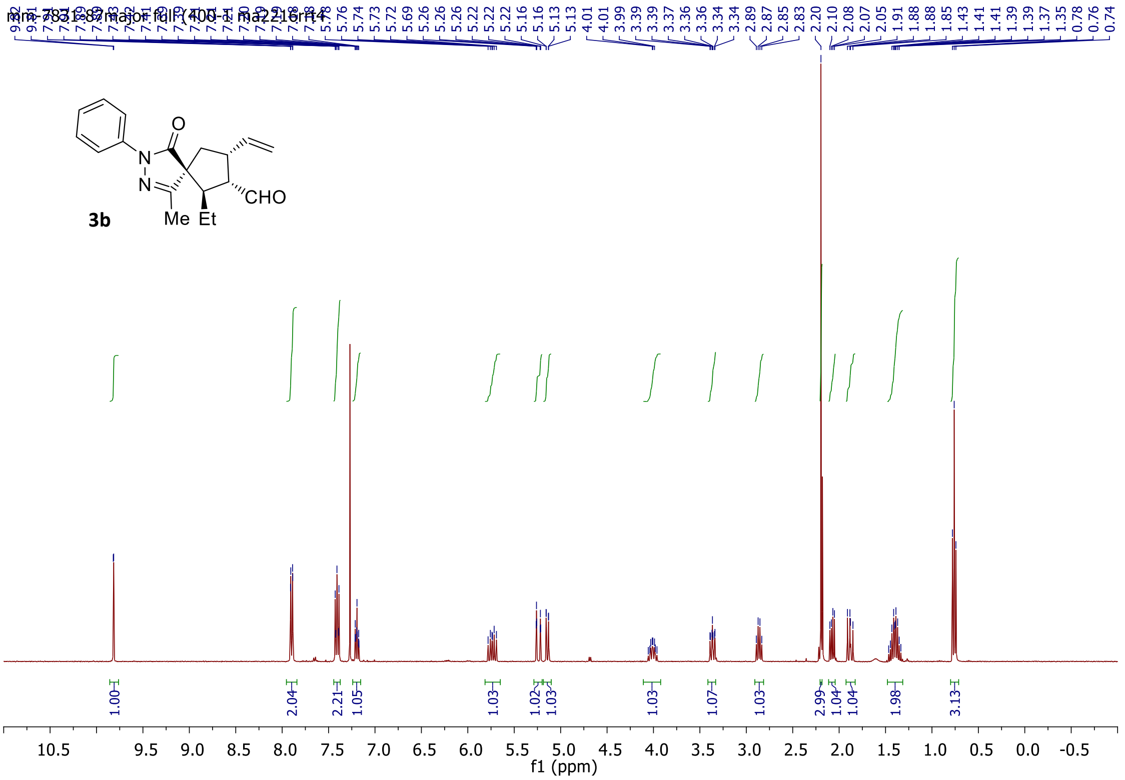
mm-7831-87major full 400-1 ma2216rrt4
3b
Me Et
CHO
f1 (ppm)

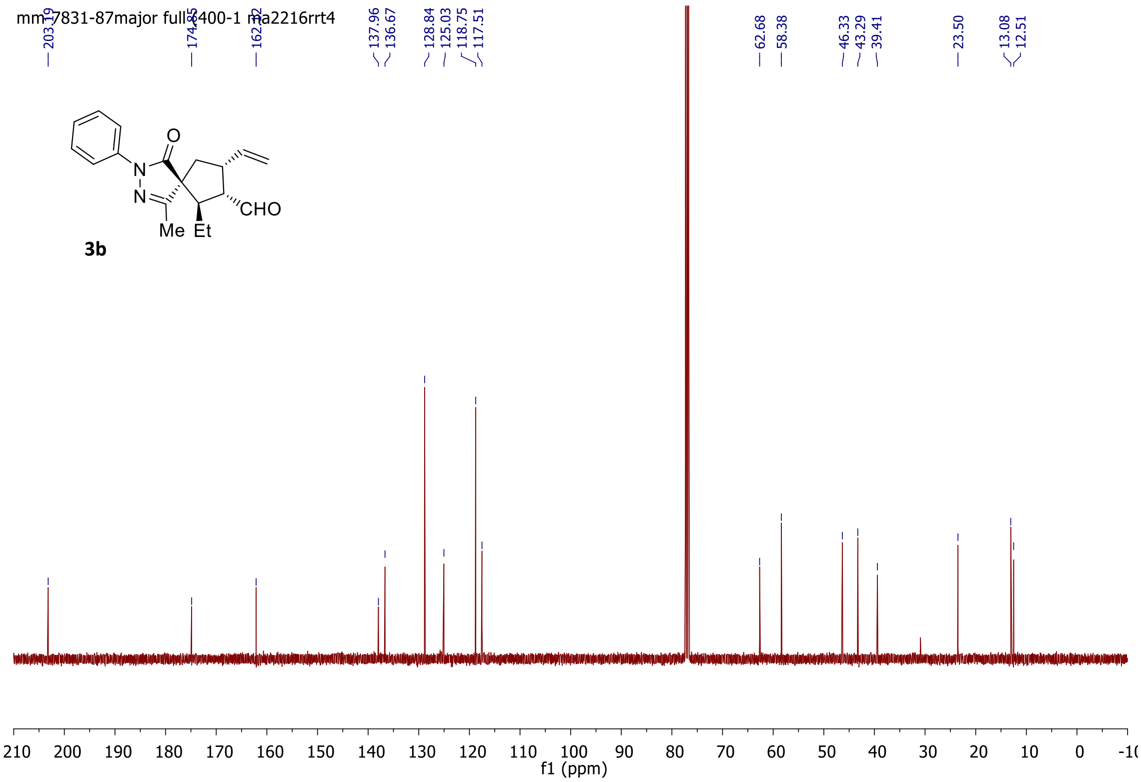
mm-7831-87major full 400-1 ma2216rrt4
203.19
162.22
137.96
136.67
128.84
125.03
118.75
117.51
62.68
58.38
46.33
43.29
39.41
23.50
13.08
12.51
3b
Me Et
CHO
f1 (ppm)

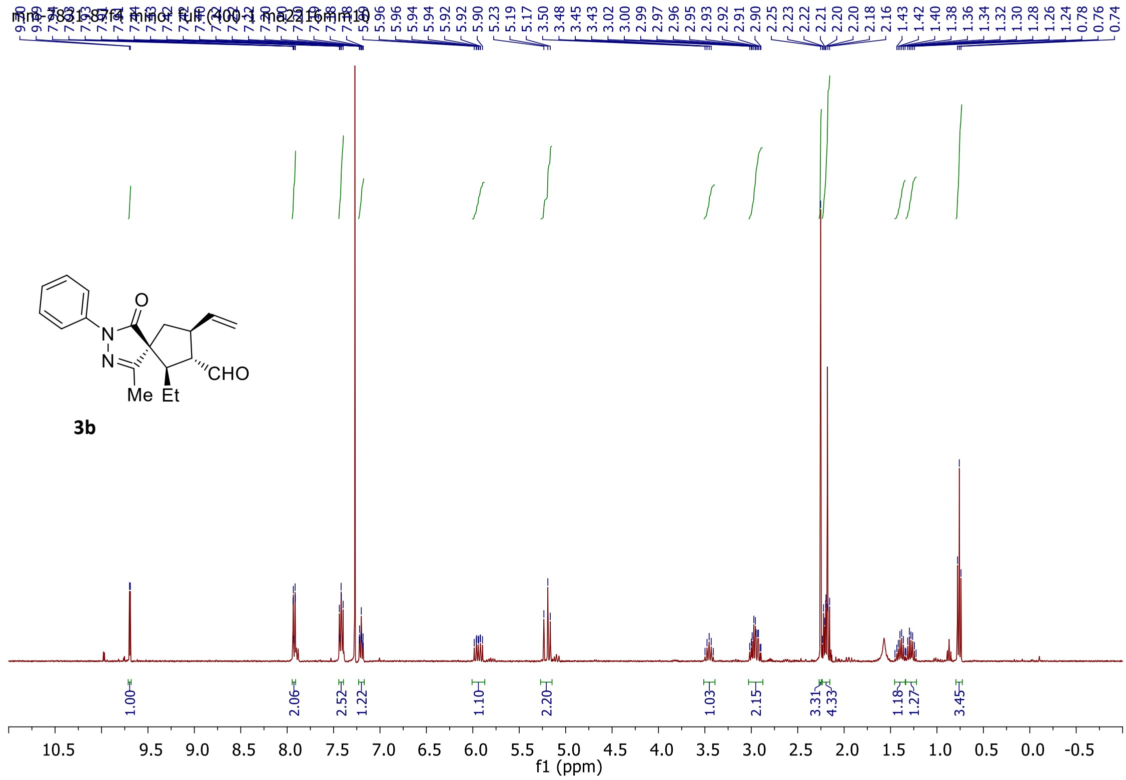
5.96
5.96
5.94
5.94
5.92
5.92
5.90
5.23
5.19
5.17
3.50
3.48
3.45
3.43
3.02
3.00
2.99
2.97
2.96
2.95
2.93
2.92
2.91
2.90
2.25
2.23
2.22
2.21
2.20
2.20
2.18
2.16
1.43
1.42
1.40
1.38
1.36
1.34
1.32
1.30
1.28
1.26
1.24
0.78
0.76
0.74
CHO
Me Et
3b
1.00
2.06
2.52
1.22
1.10
2.20
1.03
2.15
3.31
4.33
1.18
1.27
3.45
f1 (ppm)

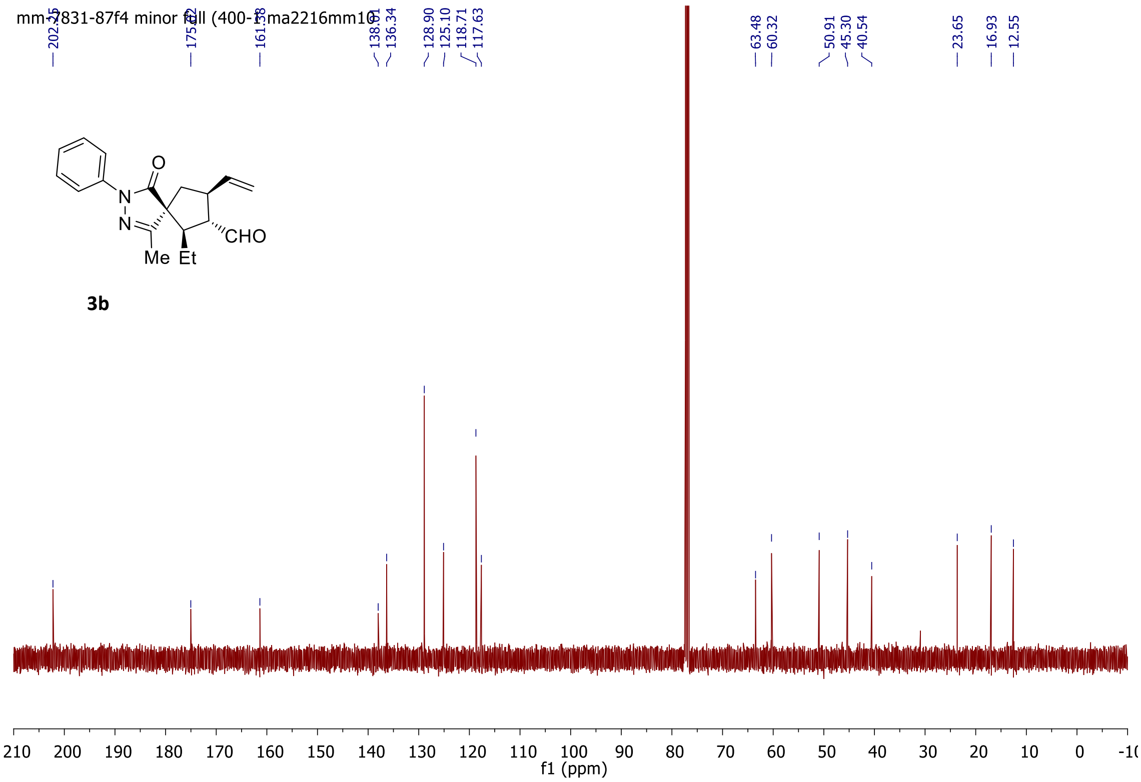
136.34
128.90
125.10
118.71
117.63
63.48
60.32
50.91
45.30
40.54
23.65
16.93
12.55
CHO
Me Et
3b
f1 (ppm)

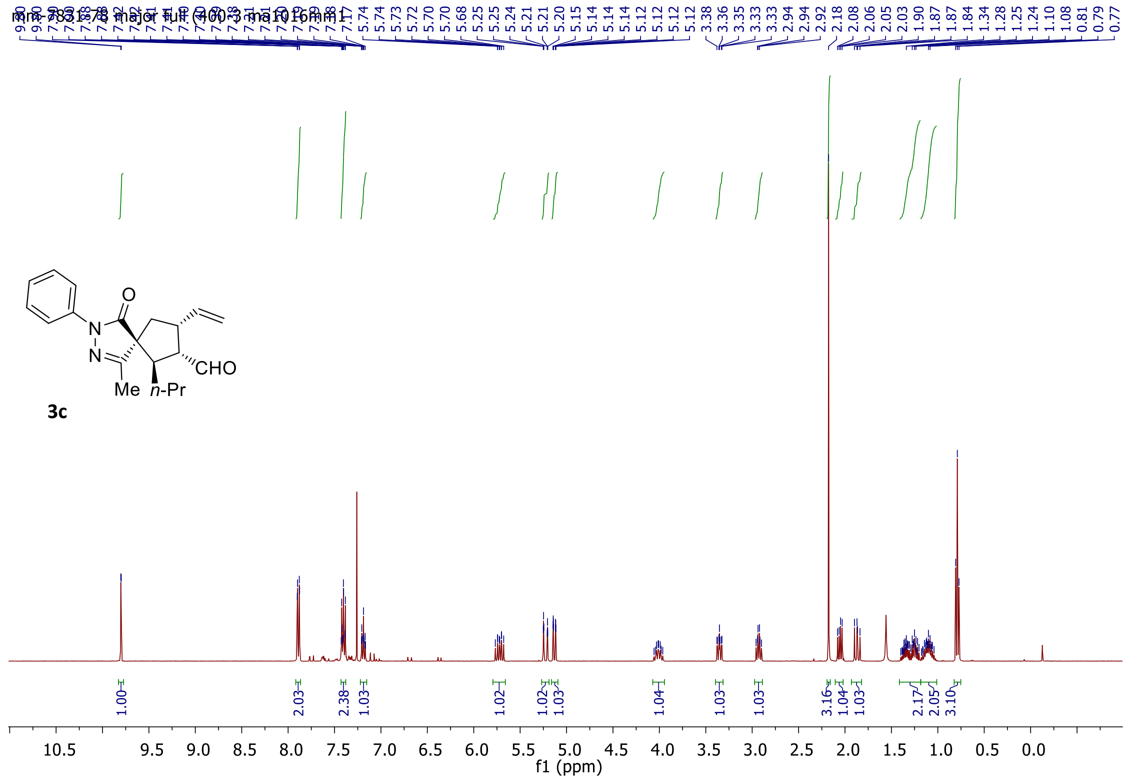
mm-7831-78 major full (400-3 ma1016mm1
5.74 5.73 5.72 5.70 5.68 5.25 5.24 5.21 5.21 5.20 5.15 5.14 5.14 5.12 5.12 5.12 5.12 3.38 3.36 3.35 3.33 3.33 2.94 2.94 2.92 2.18 2.08 2.06 2.05 2.03 1.90 1.87 1.87 1.84 1.34 1.28 1.25 1.24 1.10 1.08 0.81 0.79 0.77
N
N
O
CHO
Me
n-Pr
3c
1.00 2.03 2.38 1.03 1.02 1.02 1.03 1.04 1.03 1.03 3.16 1.04 1.03 2.17 2.05 3.10
10.5 9.5 9.0 8.5 8.0 7.5 7.0 6.5 6.0 5.5 5.0 4.5 4.0 3.5 3.0 2.5 2.0 1.5 1.0 0.5 0.0
f1 (ppm)

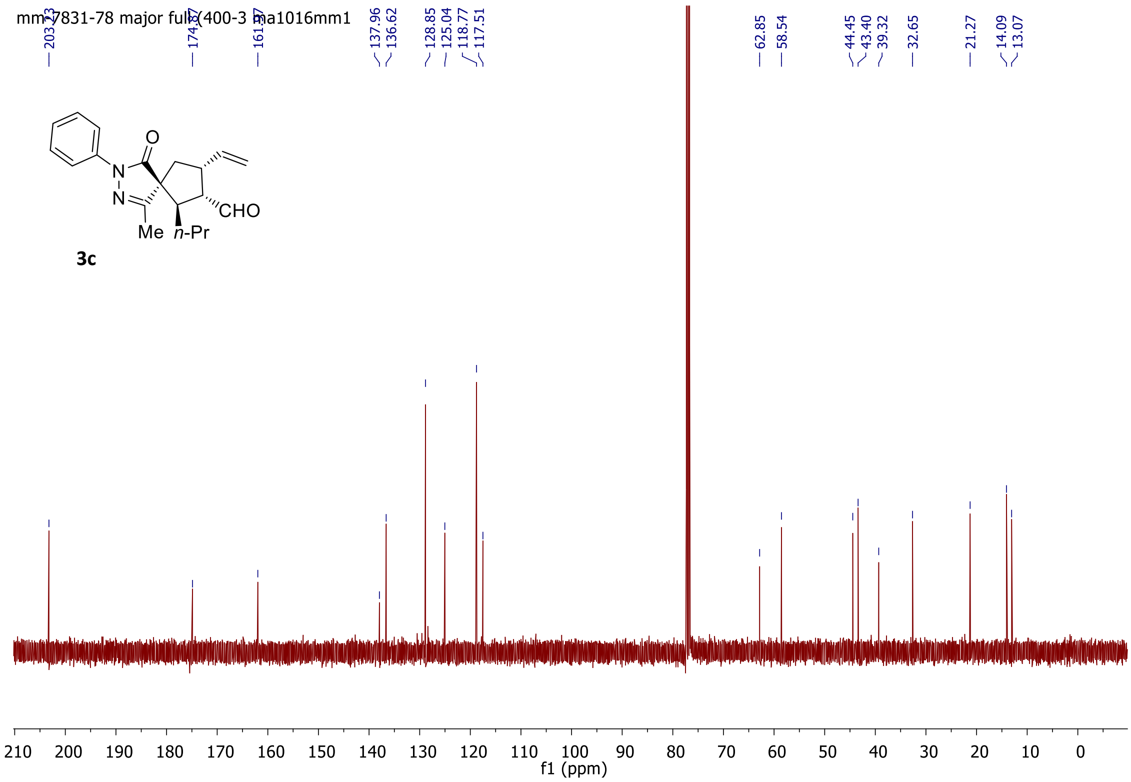
mm-7831-78 major full (400-3 ma1016mm1
203.13 174.37 161.97 137.96 136.62 128.85 125.04 118.77 117.51 62.85 58.54 44.45 43.40 39.32 32.65 21.27 14.09 13.07
N
N
O
CHO
Me
n-Pr
3c
210 200 190 180 170 160 150 140 130 120 110 100 90 80 70 60 50 40 30 20 10 0
f1 (ppm)

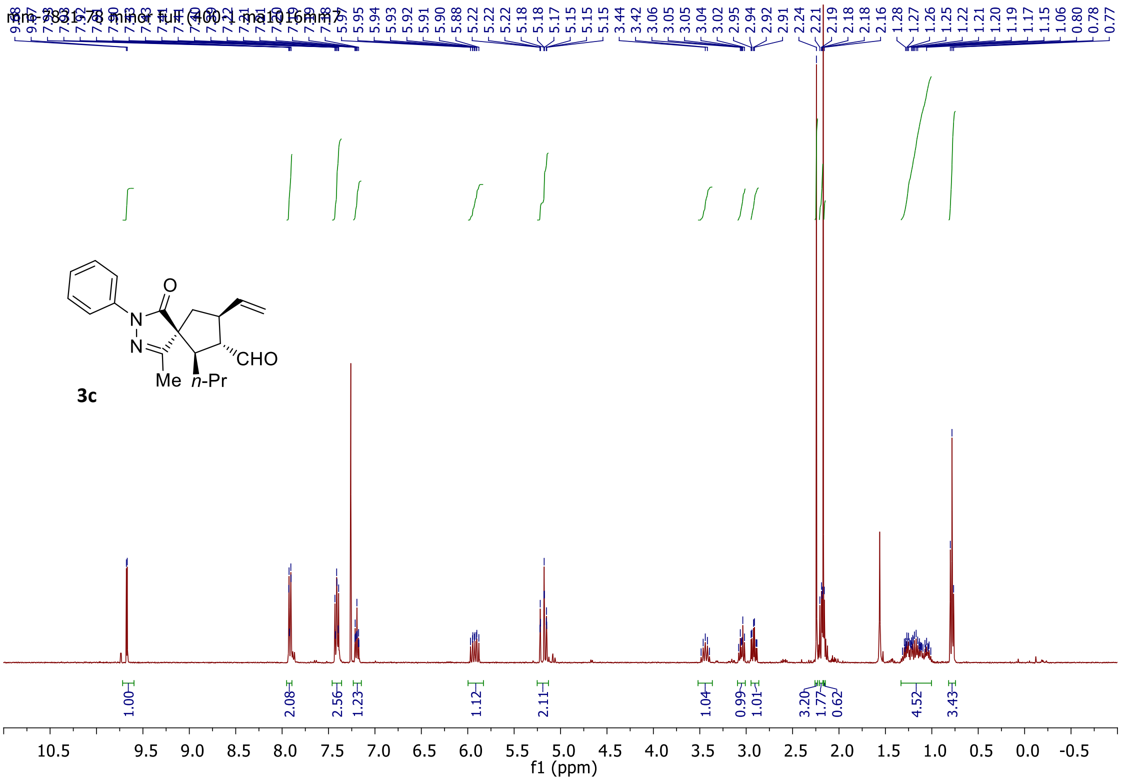
CHO
Me
n-Pr
3c
f1 (ppm)

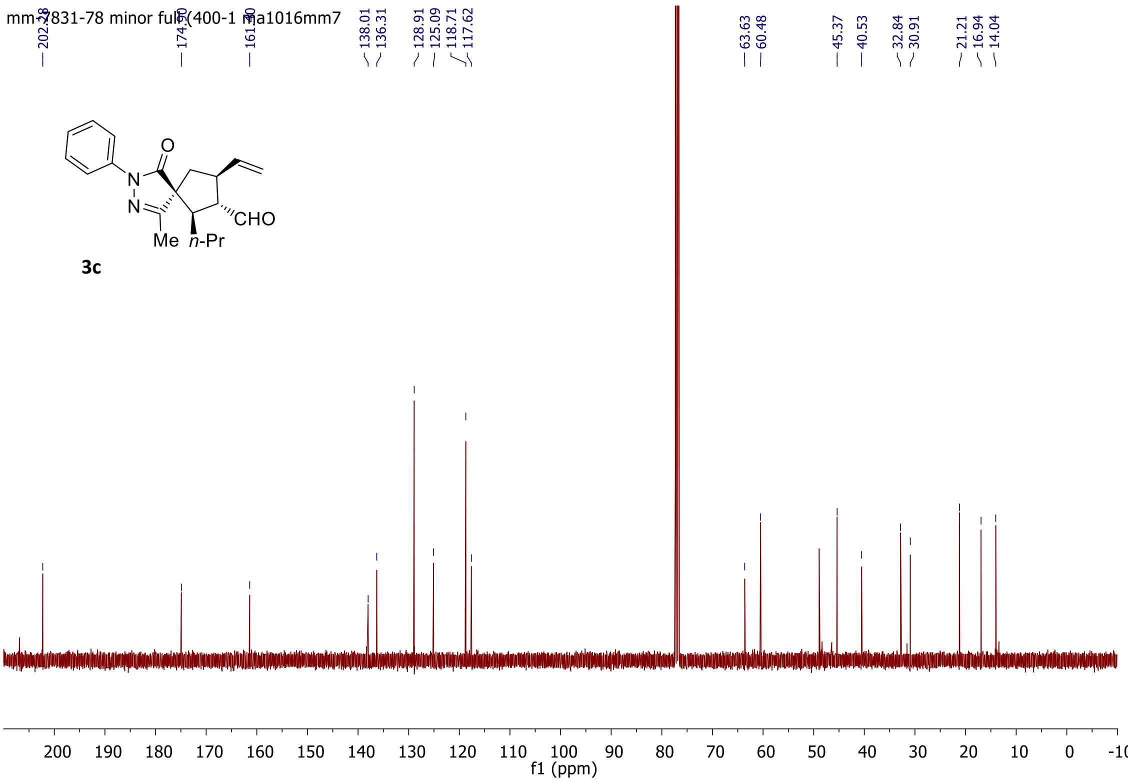
mm-7831-78 minor full (400-1
ma1016mm7
138.01
136.31
128.91
125.09
118.71
117.62
63.63
60.48
45.37
40.53
32.84
30.91
21.21
16.94
14.04
CHO
Me
n-Pr
3c
f1 (ppm)

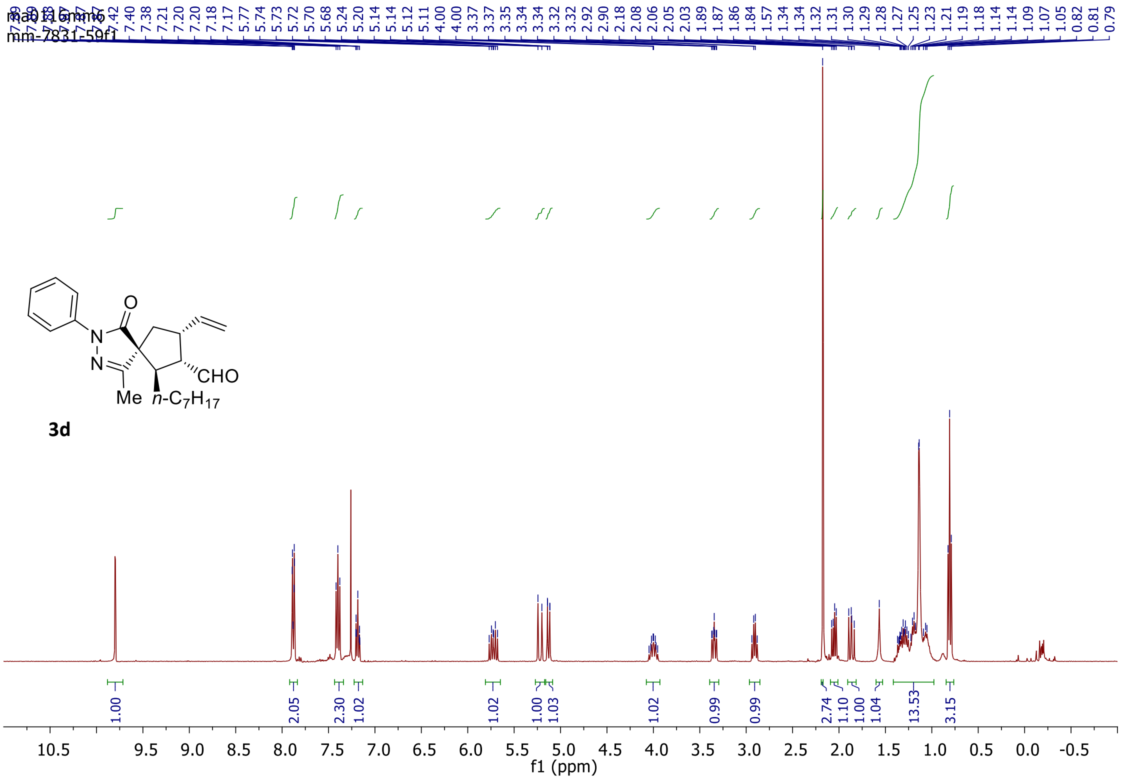
mm-7831-59f1
7.42 7.40 7.38 7.21 7.20 7.20 7.18 7.17 5.77 5.74 5.73 5.72 5.70 5.68 5.24 5.20 5.14 5.14 5.12 5.11 4.00 3.37 3.35 3.34 3.34 3.32 3.32 2.92 2.90 2.18 2.08 2.06 2.05 2.03 1.89 1.87 1.86 1.84 1.57 1.34 1.34 1.32 1.31 1.30 1.29 1.28 1.27 1.25 1.23 1.21 1.19 1.18 1.14 1.14 1.09 1.07 1.05 0.82 0.81 0.79
O
N
N
CHO
Me
n-C7H17
3d
1.00 2.05 2.30 1.02 1.02 1.00 1.03 1.02 0.99 0.99 2.74 1.10 1.00 1.04 13.53 3.15
10.5 9.5 9.0 8.5 8.0 7.5 7.0 6.5 6.0 5.5 5.0 4.5 4.0 3.5 3.0 2.5 2.0 1.5 1.0 0.5 0.0 -0.5
f1 (ppm)

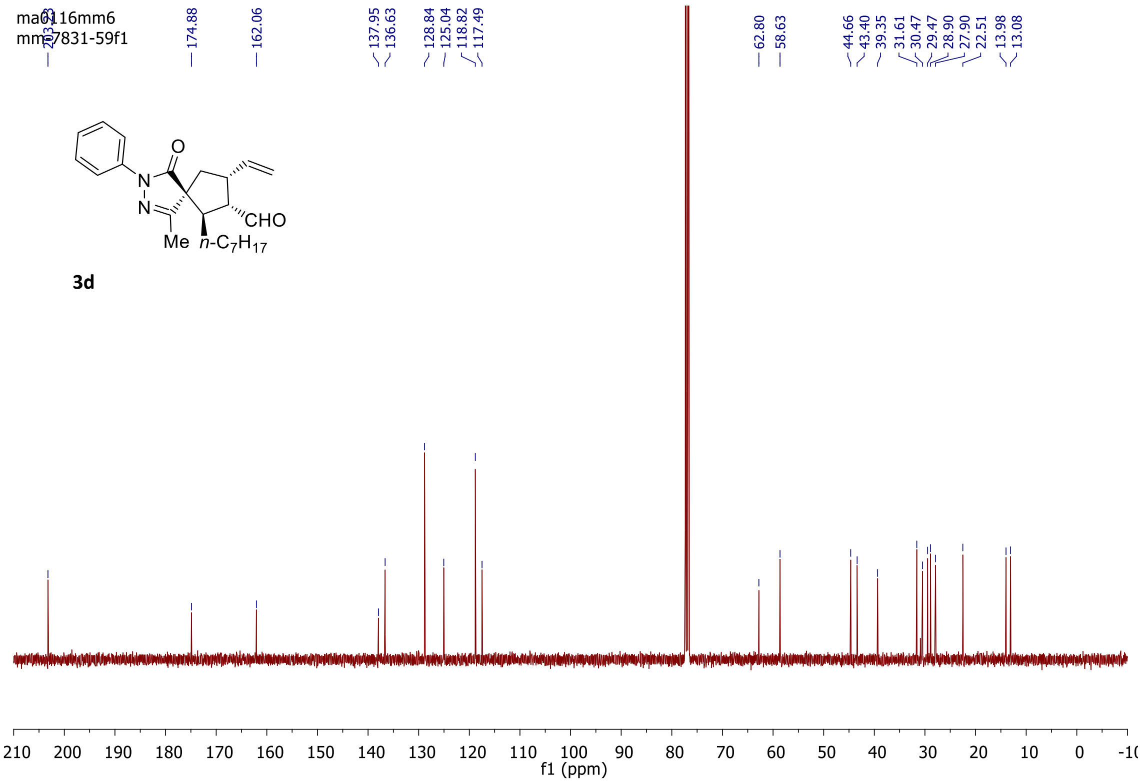
ma0116mm6
mm-7831-59f1
174.88
162.06
137.95
136.63
128.84
125.04
118.82
117.49
62.80
58.63
44.66
43.40
39.35
31.61
30.47
29.47
28.90
27.90
22.51
13.98
13.08
O
N
N
CHO
Me
n-C7H17
3d
210 200 190 180 170 160 150 140 130 120 110 100 90 80 70 60 50 40 30 20 10 0
f1 (ppm)

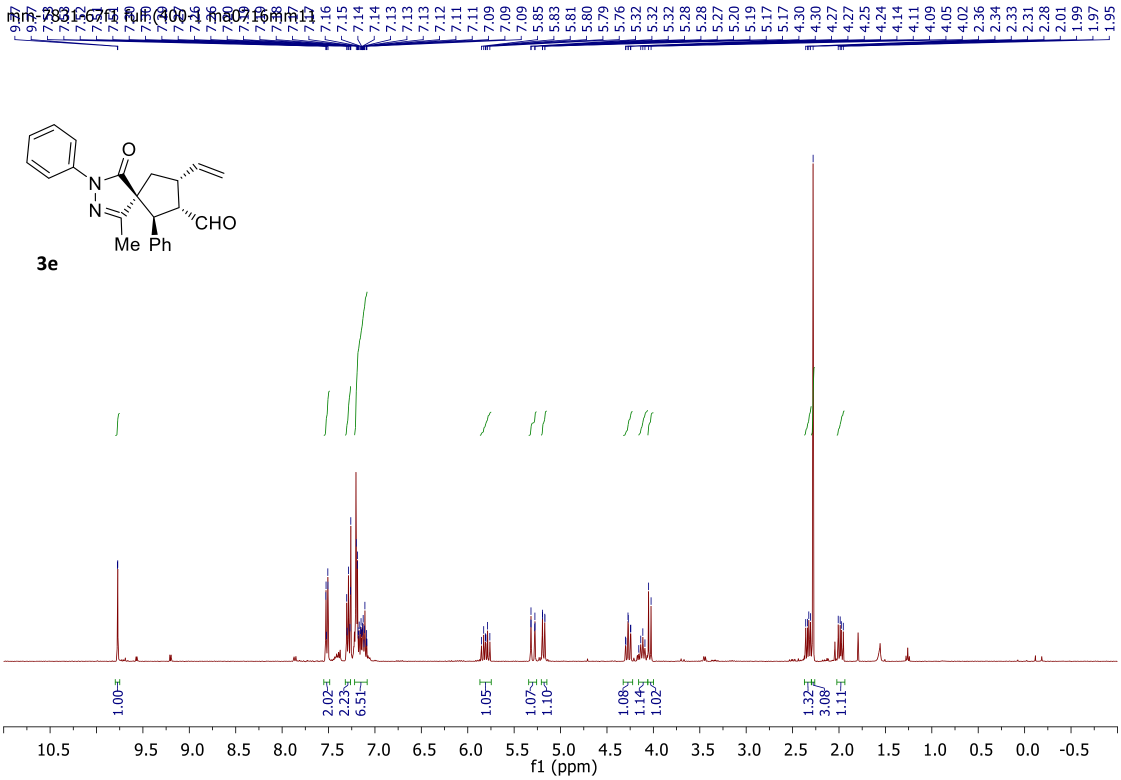
mm-7831-67f1 full (400-1 ma0716mm11
9.77
7.16
7.15
7.14
7.14
7.13
7.13
7.13
7.12
7.11
7.11
7.09
7.09
7.09
5.85
5.83
5.81
5.80
5.79
5.76
5.32
5.32
5.32
5.28
5.28
5.27
5.20
5.19
5.17
5.17
4.30
4.30
4.27
4.27
4.25
4.24
4.14
4.11
4.09
4.05
4.02
2.36
2.34
2.33
2.31
2.28
2.01
1.99
1.97
1.95
O
N
N
CHO
Me
Ph
3e
1.00
2.02
2.23
6.51
1.05
1.07
1.10
1.08
1.14
1.02
1.32
3.08
1.11
10.5
9.5
9.0
8.5
8.0
7.5
7.0
6.5
6.0
5.5
5.0
4.5
4.0
3.5
3.0
2.5
2.0
1.5
1.0
0.5
0.0
-0.5
f1 (ppm)

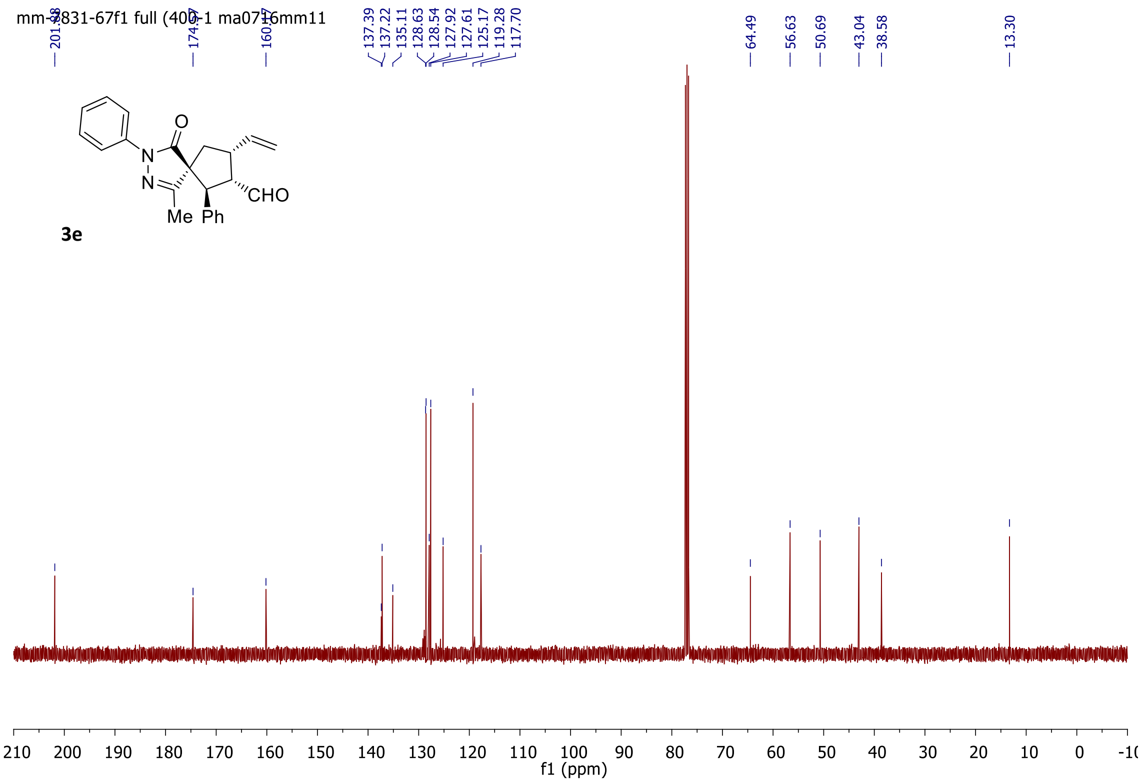
mm-7831-67f1 full (400-1 ma0716mm11
137.39
137.22
135.11
128.63
128.54
127.92
127.61
125.17
119.28
117.70
64.49
56.63
50.69
43.04
38.58
13.30
O
N
N
CHO
Me
Ph
3e
210
200
190
180
170
160
150
140
130
120
110
100
90
80
70
60
50
40
30
20
10
0
f1 (ppm)

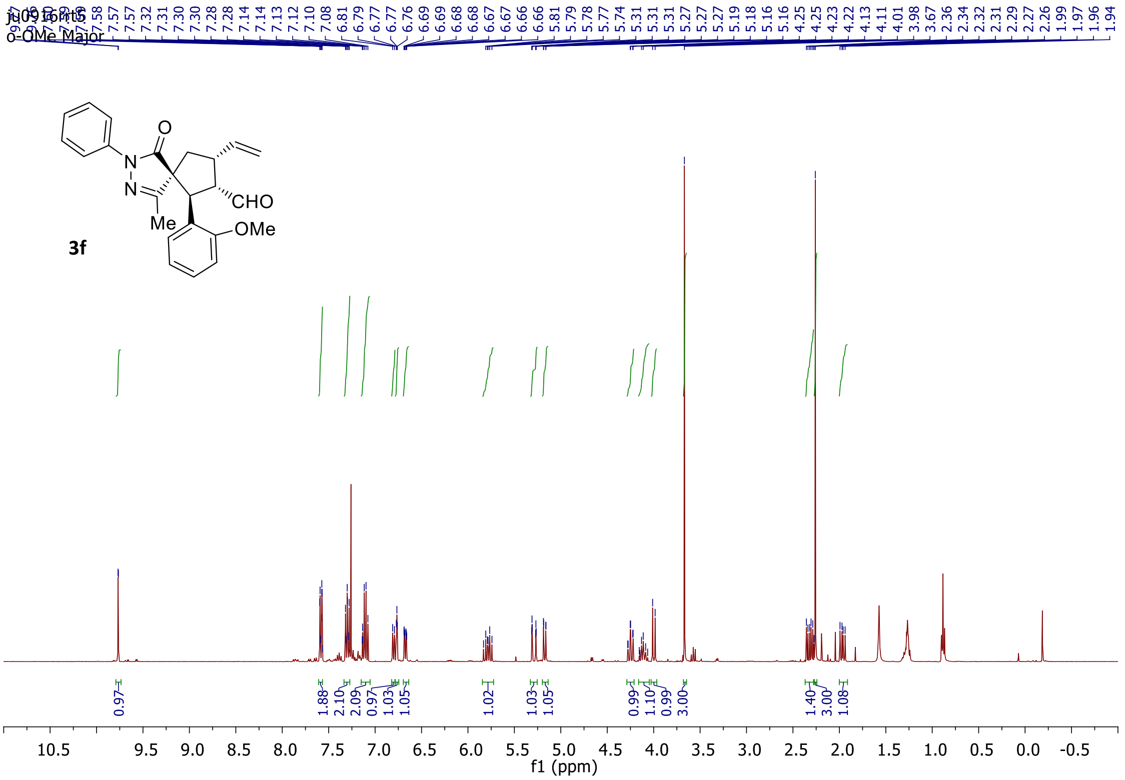
ju0916rrt5
o-OMe Major
7.58 7.57 7.57 7.32 7.31 7.30 7.30 7.28 7.28 7.14 7.14 7.13 7.12 7.10 7.08 6.81 6.79 6.77 6.77 6.76 6.69 6.69 6.68 6.67 6.67 6.66 6.66 5.81 5.79 5.78 5.77 5.74 5.31 5.31 5.31 5.27 5.27 5.27 5.19 5.18 5.16 5.16 4.25 4.25 4.23 4.22 4.13 4.11 4.01 3.98 3.67 2.36 2.34 2.32 2.31 2.29 2.27 2.26 1.99 1.97 1.96 1.94
O
N
N
CHO
Me
OMe
3f
0.97 1.88 2.10 2.09 0.97 1.03 1.05 1.02 1.03 1.05 0.99 1.10 0.99 3.00 1.40 3.00 1.08
f1 (ppm)

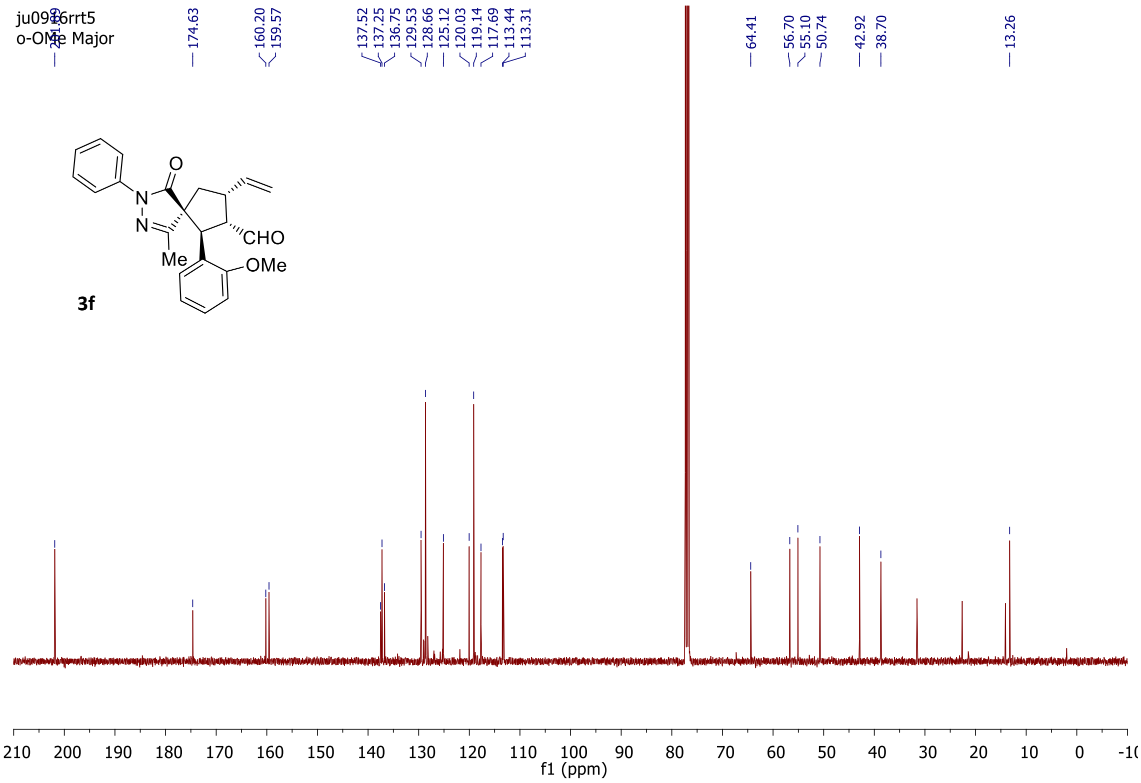
ju0916rrt5
o-OMe Major
174.63
160.20 159.57
137.52 137.25 136.75 129.53 128.66 125.12 120.03 119.14 117.69 113.44 113.31
64.41
56.70 55.10 50.74
42.92 38.70
13.26
O
N
N
CHO
Me
OMe
3f
f1 (ppm)

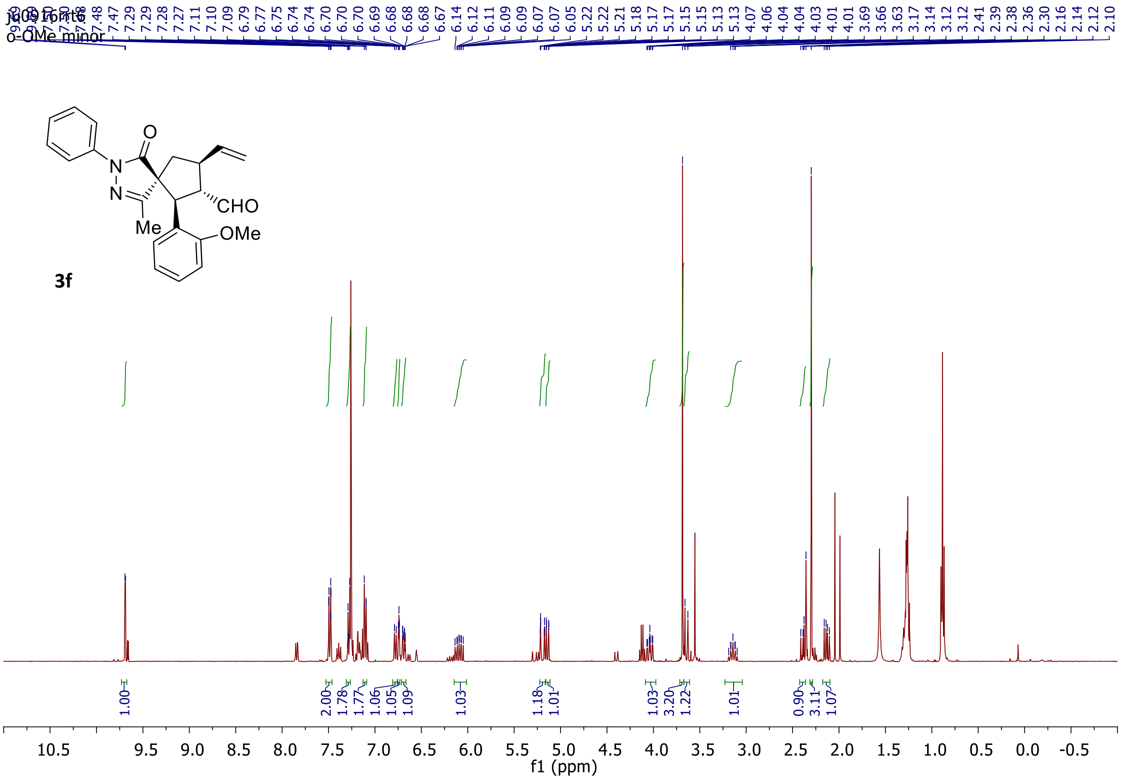
ju0916rrt6
o-OMe minor
7.48 7.47 7.29 7.29 7.28 7.27 7.11 7.10 7.09 6.79 6.77 6.75 6.74 6.74 6.70 6.70 6.70 6.69 6.68 6.68 6.68 6.67 6.14 6.12 6.11 6.09 6.09 6.07 6.07 6.05 5.22 5.22 5.21 5.18 5.17 5.17 5.15 5.15 5.13 5.13 4.07 4.06 4.04 4.04 4.03 4.01 4.01 3.69 3.66 3.63 3.17 3.14 3.12 3.12 2.41 2.39 2.38 2.36 2.30 2.16 2.14 2.12 2.10
O
N
N
CHO
Me
OMe
3f
1.00 2.00 1.78 1.77 1.06 1.05 1.09 1.03 1.18 1.01 1.03 3.20 1.22 1.01 0.90 3.11 1.07
10.5 9.5 9.0 8.5 8.0 7.5 7.0 6.5 6.0 5.5 5.0 4.5 4.0 3.5 3.0 2.5 2.0 1.5 1.0 0.5 0.0 -0.5
f1 (ppm)

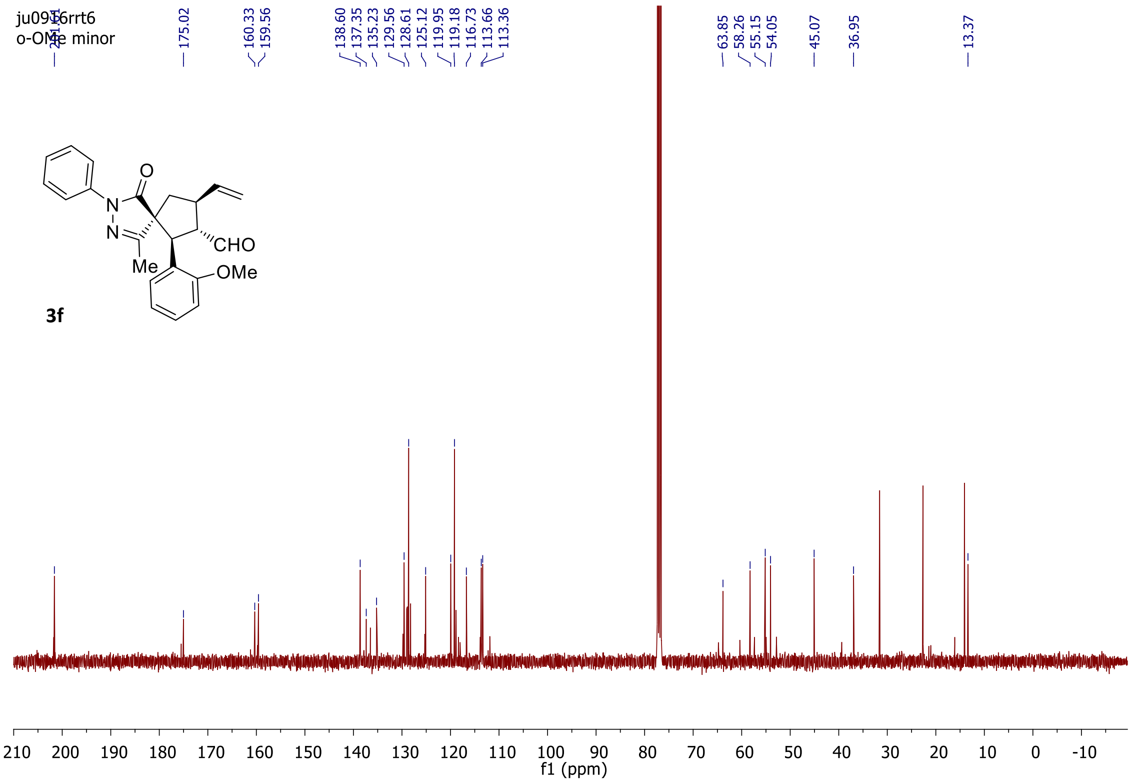
ju0916rrt6
o-OMe minor
175.02
160.33 159.56
138.60 137.35 135.23 129.56 128.61 125.12 119.95 119.18 116.73 113.66 113.36
63.85 58.26 55.15 54.05
45.07
36.95
13.37
O
N
N
CHO
Me
OMe
3f
210 200 190 180 170 160 150 140 130 120 110 100 90 80 70 60 50 40 30 20 10 0 -10
f1 (ppm)

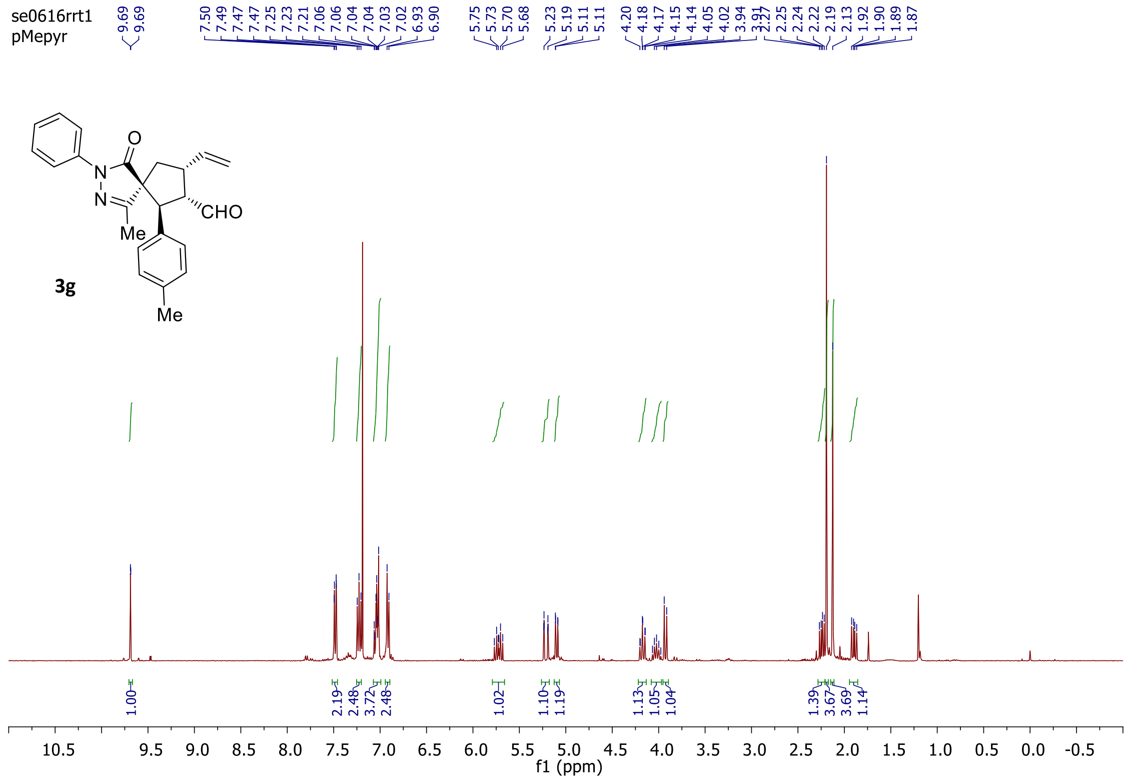
se0616rrt1
pMepyr
9.69
9.69
7.50
7.49
7.47
7.47
7.25
7.23
7.21
7.06
7.04
7.04
7.03
7.02
6.93
6.90
5.75
5.73
5.70
5.68
5.23
5.19
5.11
5.11
4.20
4.18
4.17
4.15
4.14
4.05
4.02
3.94
3.91
2.27
2.25
2.24
2.22
2.19
2.13
1.92
1.90
1.89
1.87
CHO
Me
3g
Me
1.00
2.19
2.48
3.72
2.48
1.02
1.10
1.19
1.13
1.05
1.04
1.39
3.67
3.69
1.14
f1 (ppm)

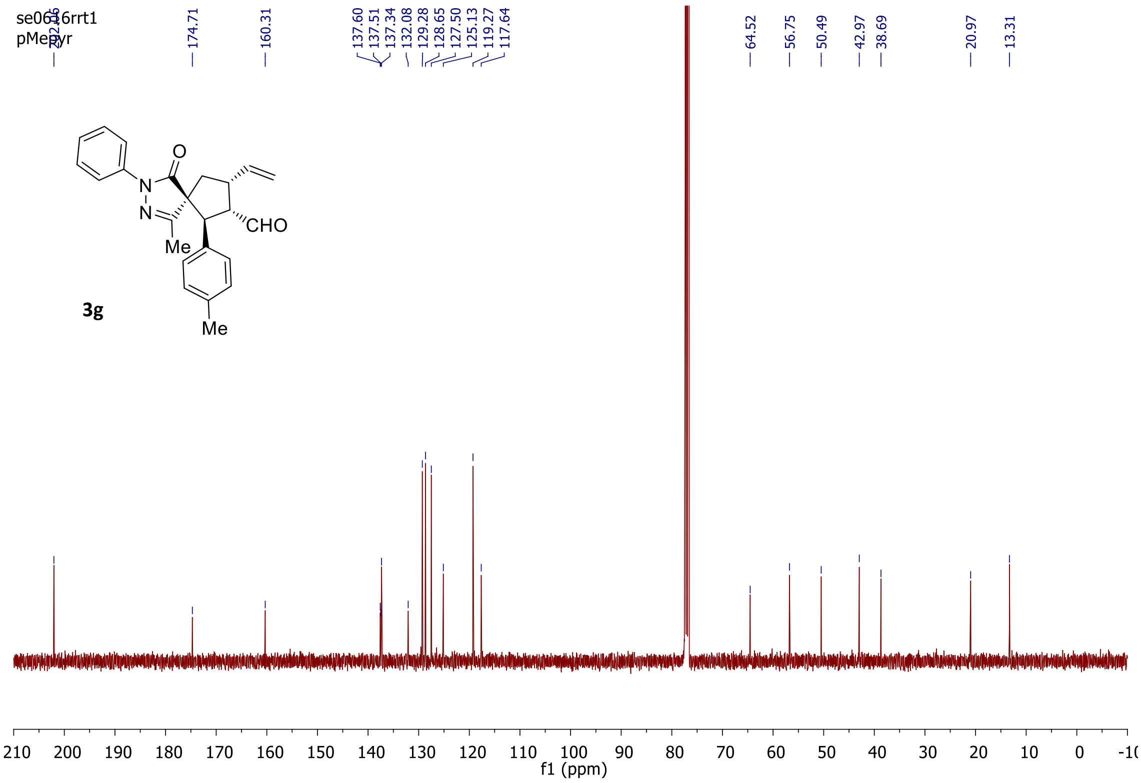
se0616rrt1
pMepyr
202.16
174.71
160.31
137.60
137.51
137.34
132.08
129.28
128.65
127.50
125.13
119.27
117.64
64.52
56.75
50.49
42.97
38.69
20.97
13.31
CHO
Me
3g
Me
f1 (ppm)

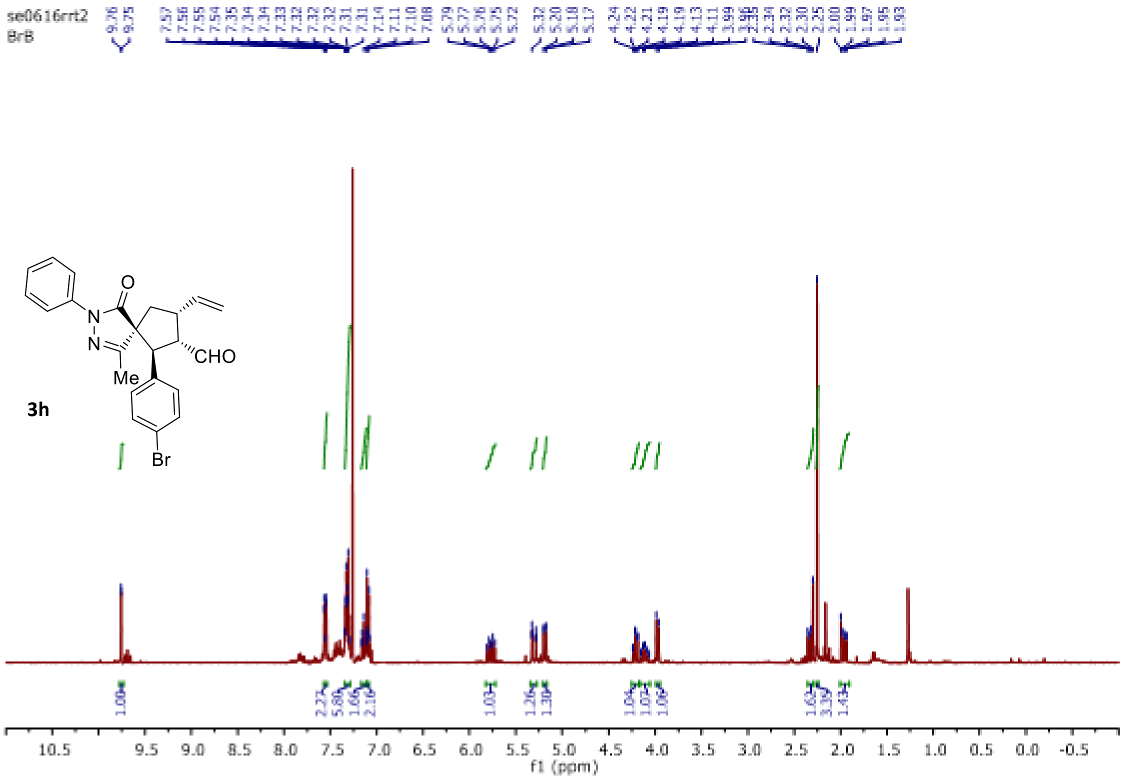

se0616rrt2
BrB
3h
O
N
N
Me
CHO
Br
f1 (ppm)

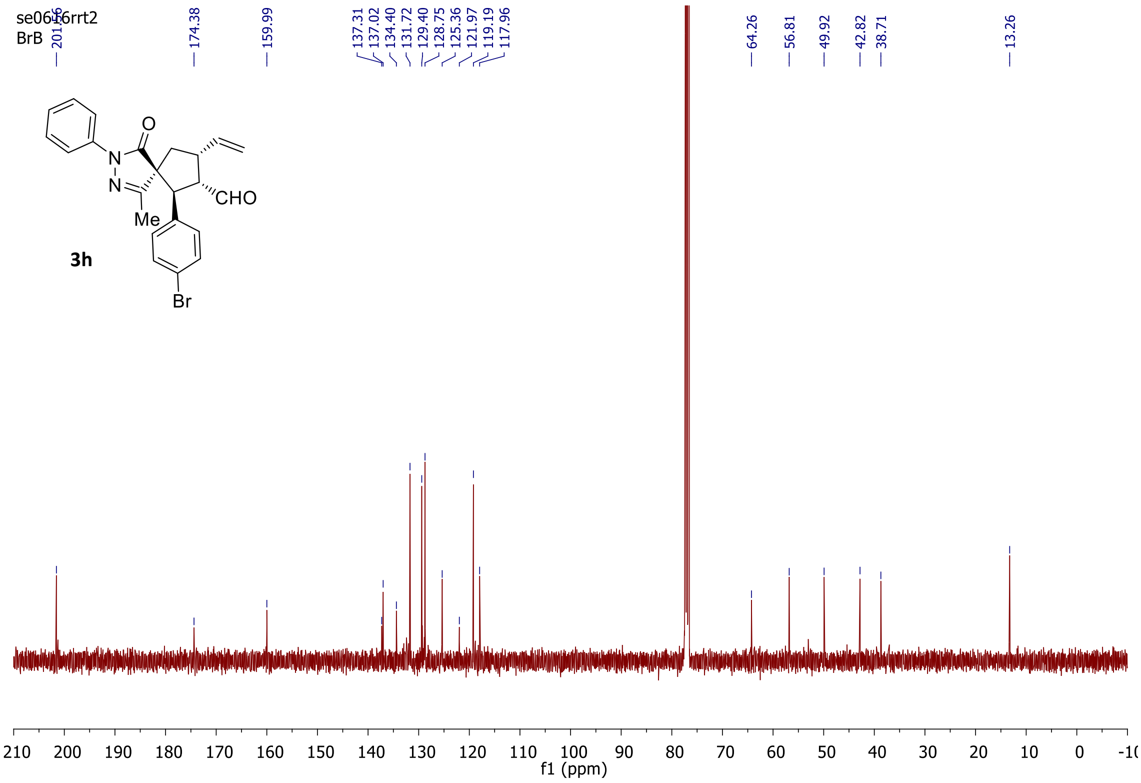

se0616rrt2
BrB
174.38
159.99
137.31
137.02
134.40
131.72
129.40
128.75
125.36
121.97
119.19
117.96
64.26
56.81
49.92
42.82
38.71
13.26
3h
O
N
N
Me
CHO
Br
f1 (ppm)

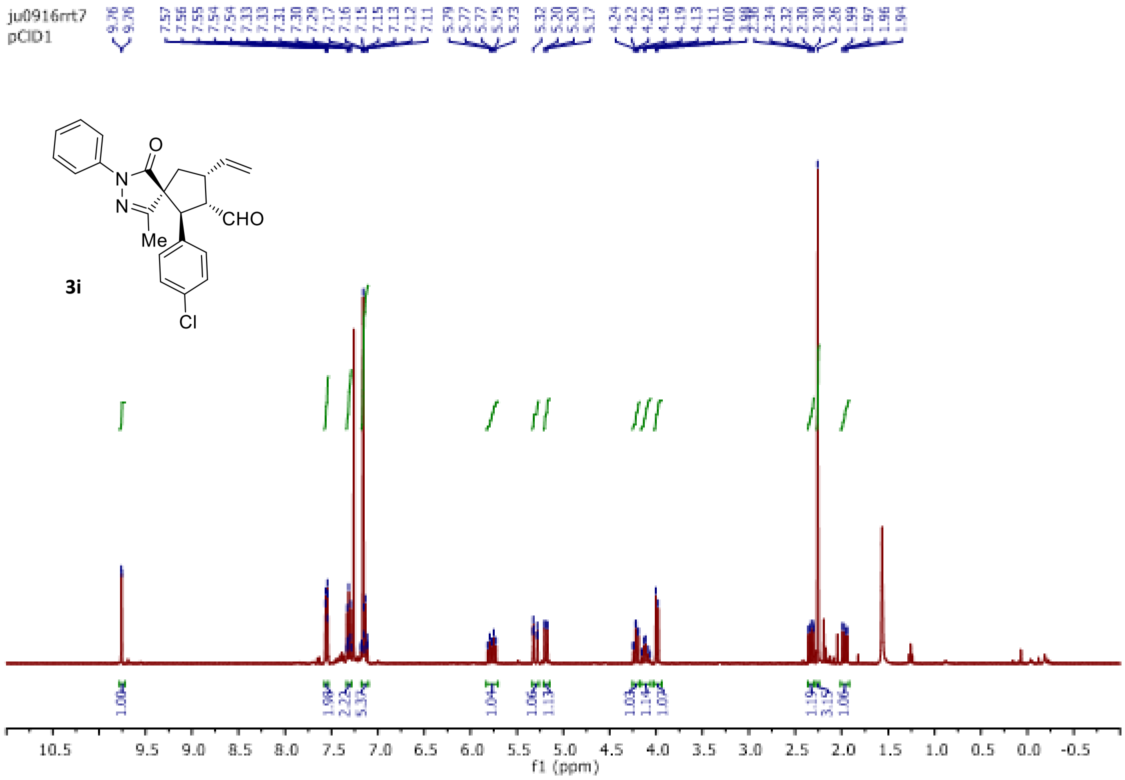
ju0916rrt7
pCID1
9.76 9.76
7.57 7.56 7.55 7.54 7.54 7.33 7.33 7.31 7.30 7.29 7.17 7.16 7.15 7.15 7.13 7.12 7.11
5.79 5.77 5.77 5.75 5.73
5.32 5.20 5.20 5.17
4.24 4.22 4.22 4.19 4.19 4.13 4.11 4.00 3.98 3.98
2.34 2.32 2.30 2.30 2.26 1.99 1.97 1.96 1.94
O
N
N
Me
CHO
Cl
3i
1.00
1.98 2.21 5.37
1.04
1.06 1.13
1.03 1.14 1.07
1.19 3.15 1.06
10.5 9.5 9.0 8.5 8.0 7.5 7.0 6.5 6.0 5.5 5.0 4.5 4.0 3.5 3.0 2.5 2.0 1.5 1.0 0.5 0.0 -0.5
f1 (ppm)

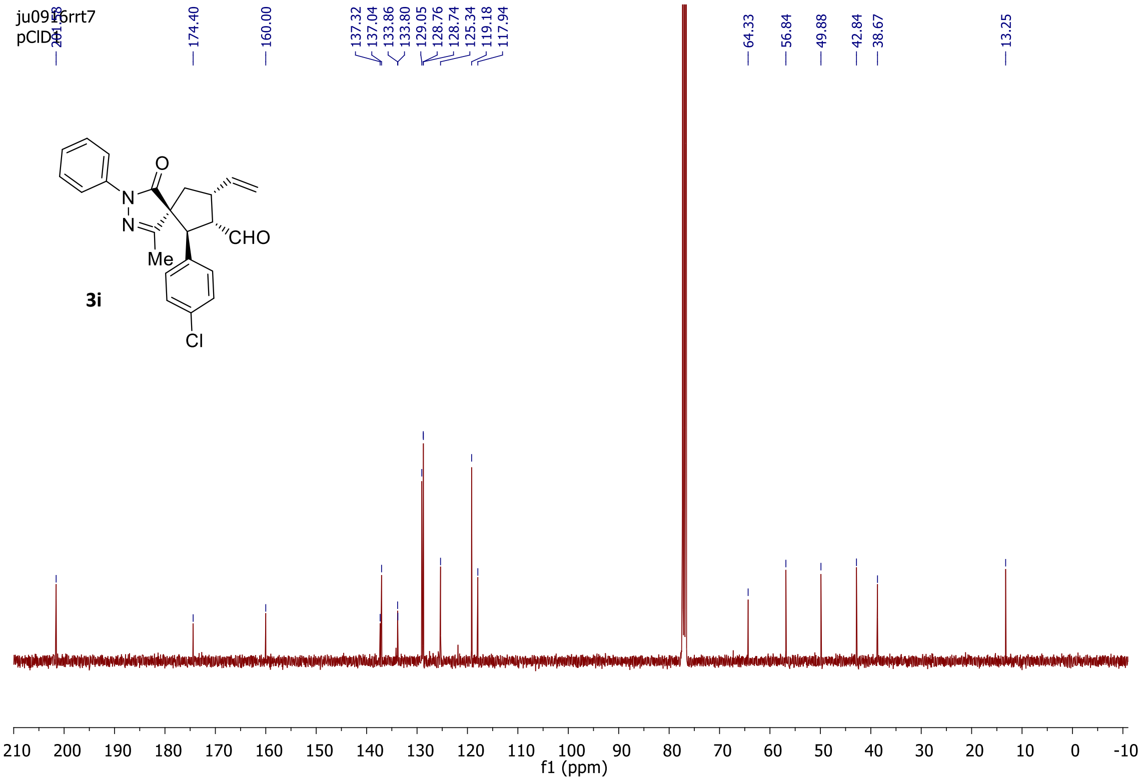
ju0916rrt7
pCID1
201.18
174.40
160.00
137.32 137.04 133.86 133.80 129.05 128.76 128.74 125.34 119.18 117.94
64.33
56.84
49.88
42.84
38.67
13.25
O
N
N
Me
CHO
Cl
3i
210 200 190 180 170 160 150 140 130 120 110 100 90 80 70 60 50 40 30 20 10 0 -10
f1 (ppm)

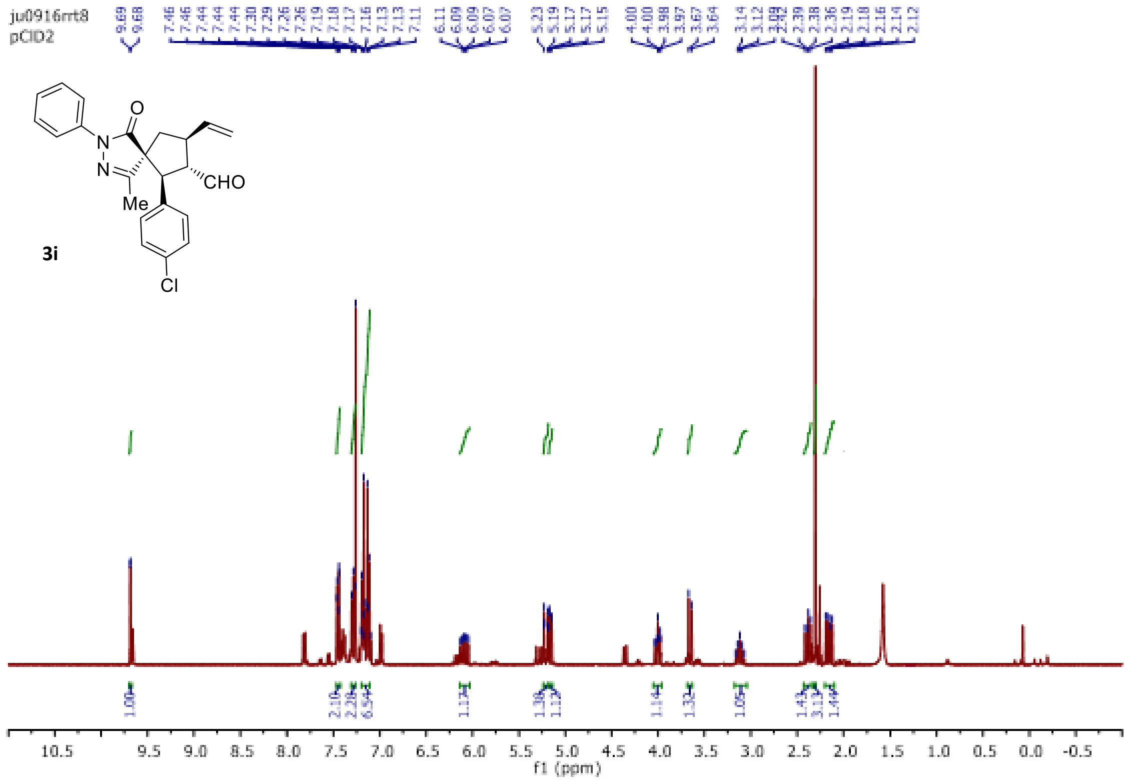
ju0916rrt8
pClD2
3i
CHO
Me
Cl
f1 (ppm)

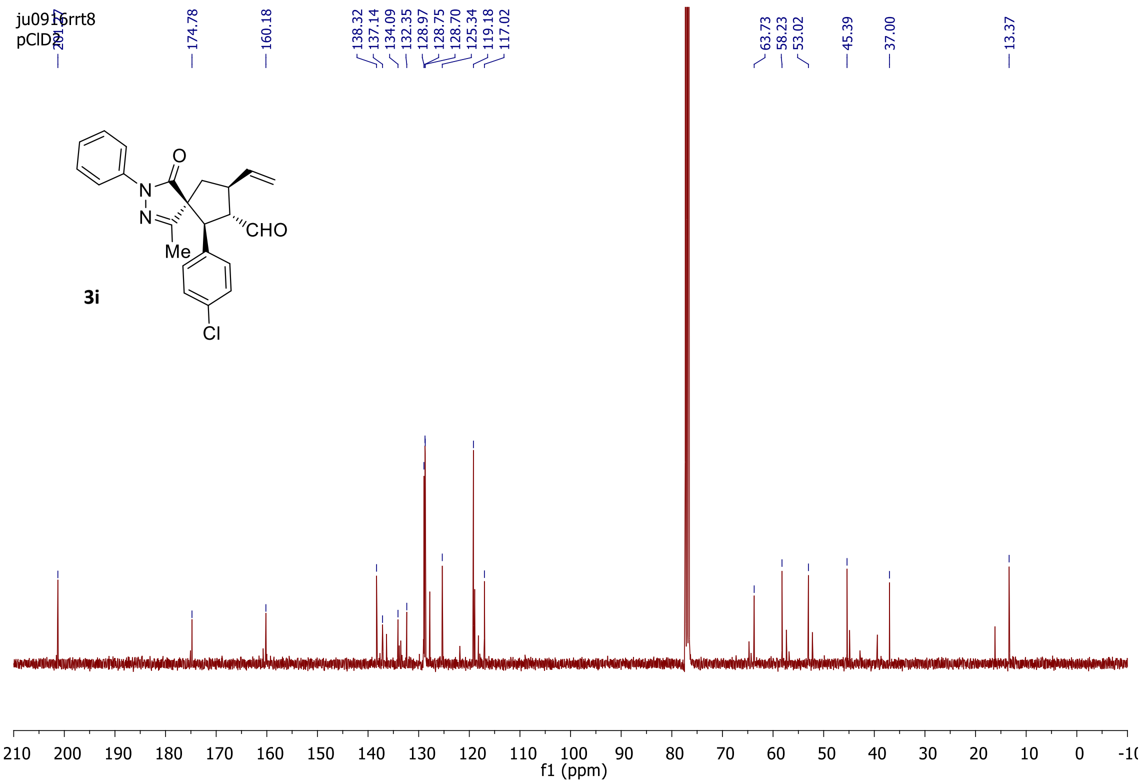
ju0916rrt8
pClD2
174.78
160.18
63.73
58.23
53.02
45.39
37.00
13.37
3i
CHO
Me
Cl
f1 (ppm)

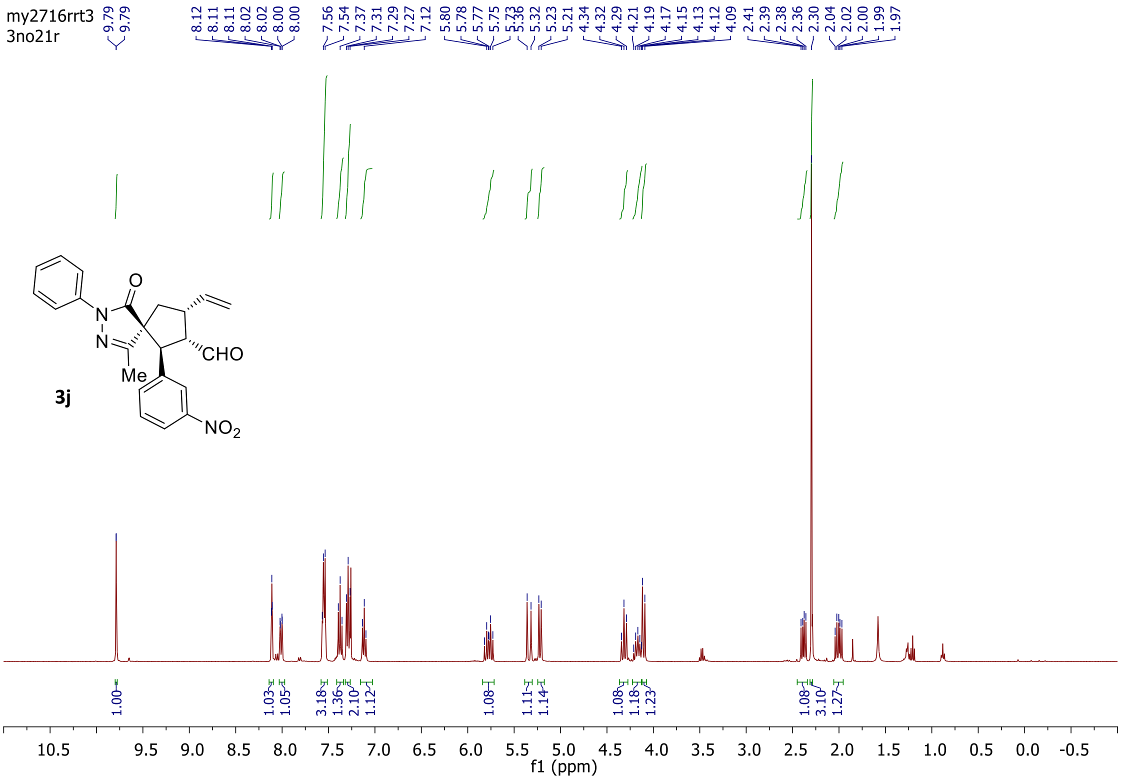
my2716rrt3
3no21r
9.79 9.79 8.12 8.11 8.11 8.02 8.02 8.00 8.00 7.56 7.54 7.37 7.31 7.29 7.27 7.12 5.80 5.78 5.77 5.75 5.73 5.36 5.32 5.23 5.21 4.34 4.32 4.29 4.21 4.19 4.17 4.15 4.13 4.12 4.09 2.41 2.39 2.38 2.36 2.30 2.04 2.02 2.00 1.99 1.97
O
N
N
N
CHO
Me
3j
NO2
1.00 1.03 1.05 3.18 1.36 2.10 1.12 1.08 1.11 1.14 1.08 1.18 1.23 1.08 3.10 1.27
10.5 9.5 9.0 8.5 8.0 7.5 7.0 6.5 6.0 5.5 5.0 4.5 4.0 3.5 3.0 2.5 2.0 1.5 1.0 0.5 0.0 -0.5
f1 (ppm)

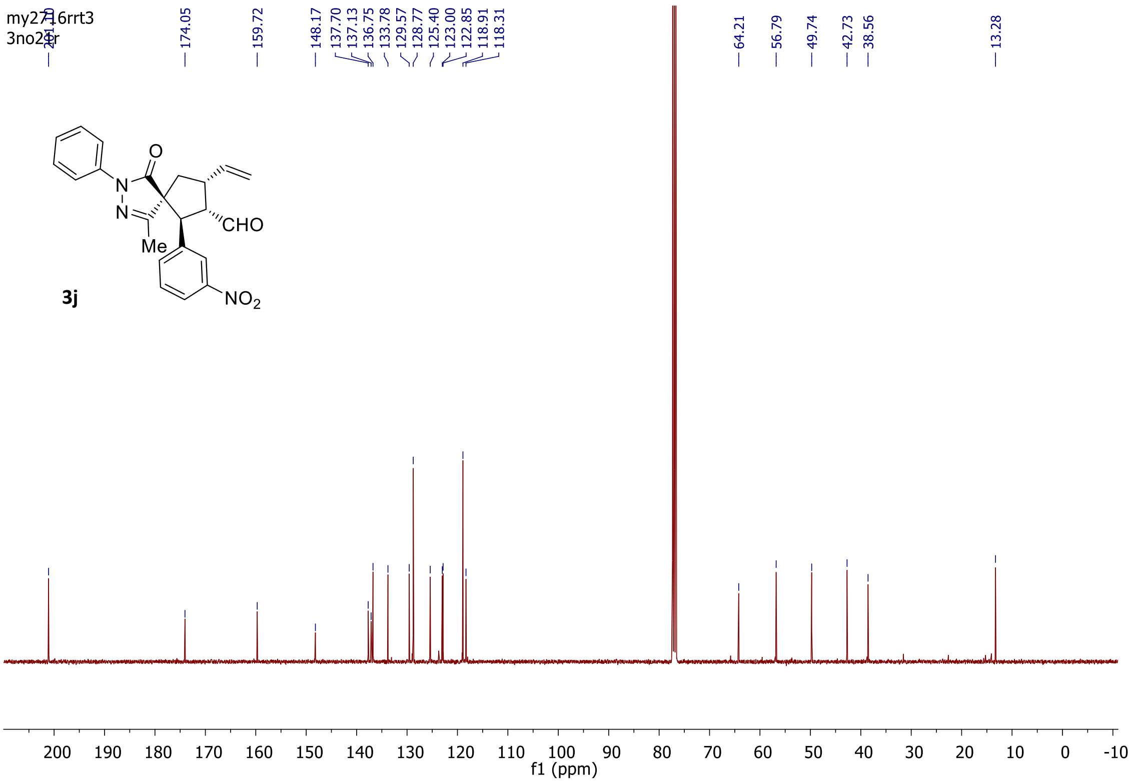
my2716rrt3
3no21r
174.05 159.72 148.17 137.70 137.13 136.75 133.78 129.57 128.77 125.40 123.00 122.85 118.91 118.31 64.21 56.79 49.74 42.73 38.56 13.28
O
N
N
N
CHO
Me
3j
NO2
200 190 180 170 160 150 140 130 120 110 100 90 80 70 60 50 40 30 20 10 0 -10
f1 (ppm)

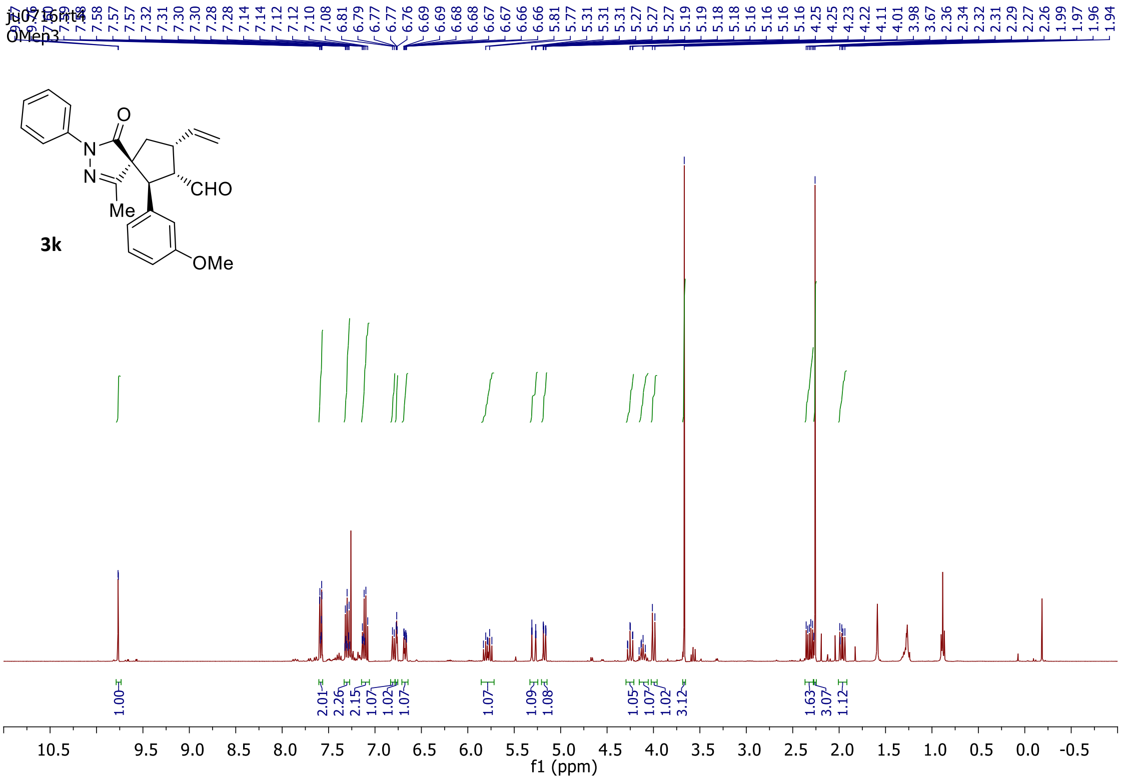

7.58
7.57
7.57
7.32
7.31
7.30
7.30
7.28
7.28
7.14
7.12
7.12
7.10
7.08
6.81
6.79
6.77
6.77
6.76
6.69
6.69
6.68
6.67
6.67
6.66
6.66
5.81
5.77
5.31
5.31
5.27
5.27
5.27
5.19
5.19
5.18
5.16
5.16
5.16
4.25
4.25
4.23
4.22
4.11
4.01
3.98
3.67
2.36
2.34
2.32
2.31
2.29
2.27
2.26
1.99
1.97
1.96
1.94
O
N
N
Me
CHO
OMe
3k
1.00
2.01
2.26
2.15
1.07
1.02
1.07
1.07
1.09
1.08
1.05
1.07
1.02
3.12
1.63
3.07
1.12
10.5
9.5
9.0
8.5
8.0
7.5
7.0
6.5
6.0
5.5
5.0
4.5
4.0
3.5
3.0
2.5
2.0
1.5
1.0
0.5
0.0
-0.5
f1 (ppm)

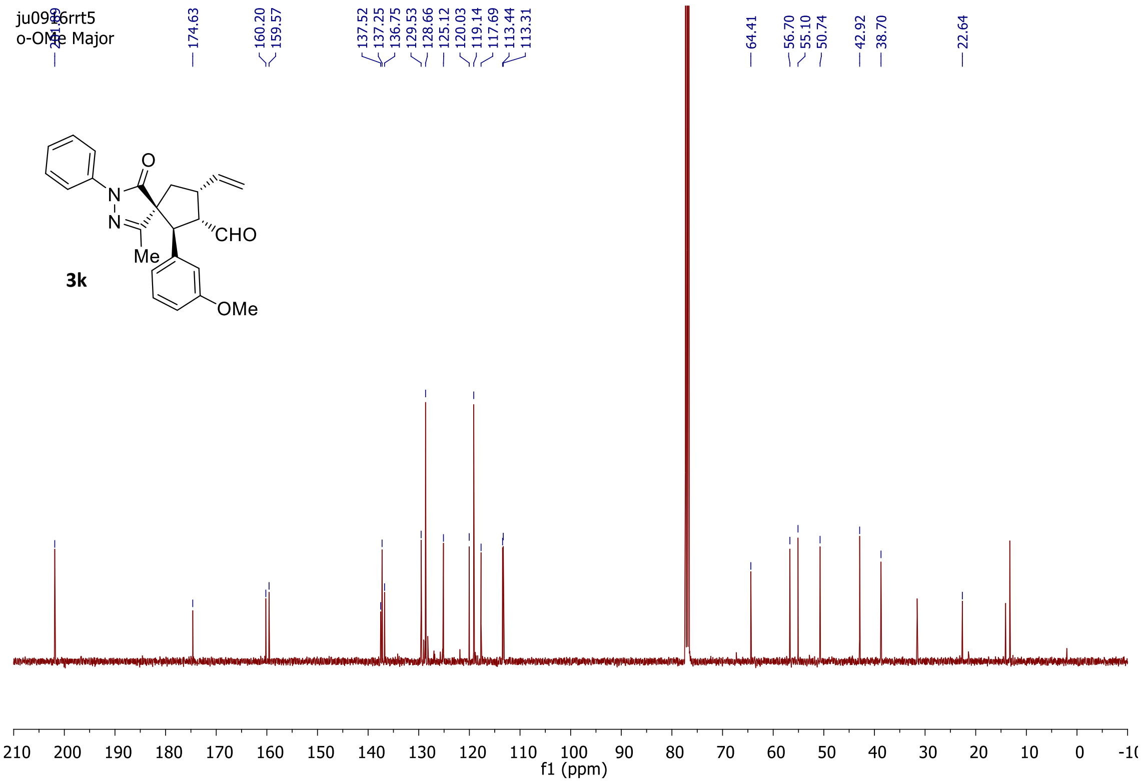

o-OMe Major
174.63
160.20
159.57
137.52
137.25
136.75
129.53
128.66
125.12
120.03
119.14
117.69
113.44
113.31
64.41
56.70
55.10
50.74
42.92
38.70
22.64
O
N
N
Me
CHO
OMe
3k
210
200
190
180
170
160
150
140
130
120
110
100
90
80
70
60
50
40
30
20
10
0
f1 (ppm)

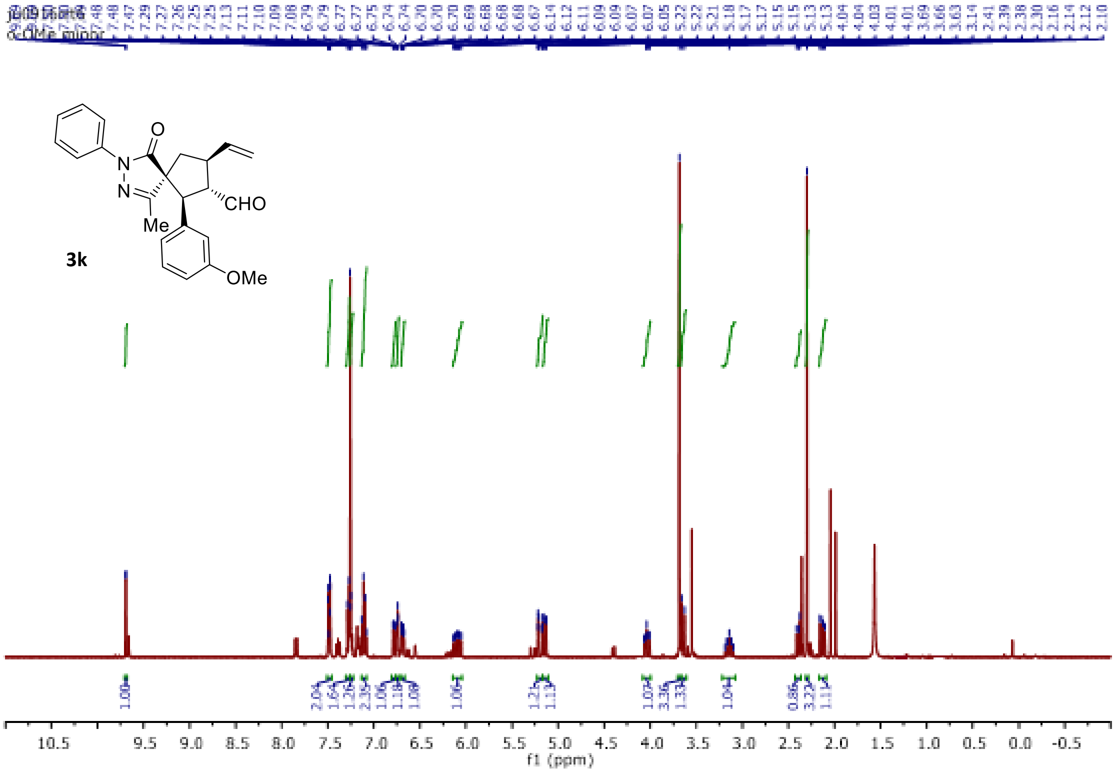

O
N
N
CHO
Me
3k
OMe
10.5
9.5
9.0
8.5
8.0
7.5
7.0
6.5
6.0
5.5
5.0
4.5
4.0
3.5
3.0
2.5
2.0
1.5
1.0
0.5
0.0
-0.5
f1 (ppm)

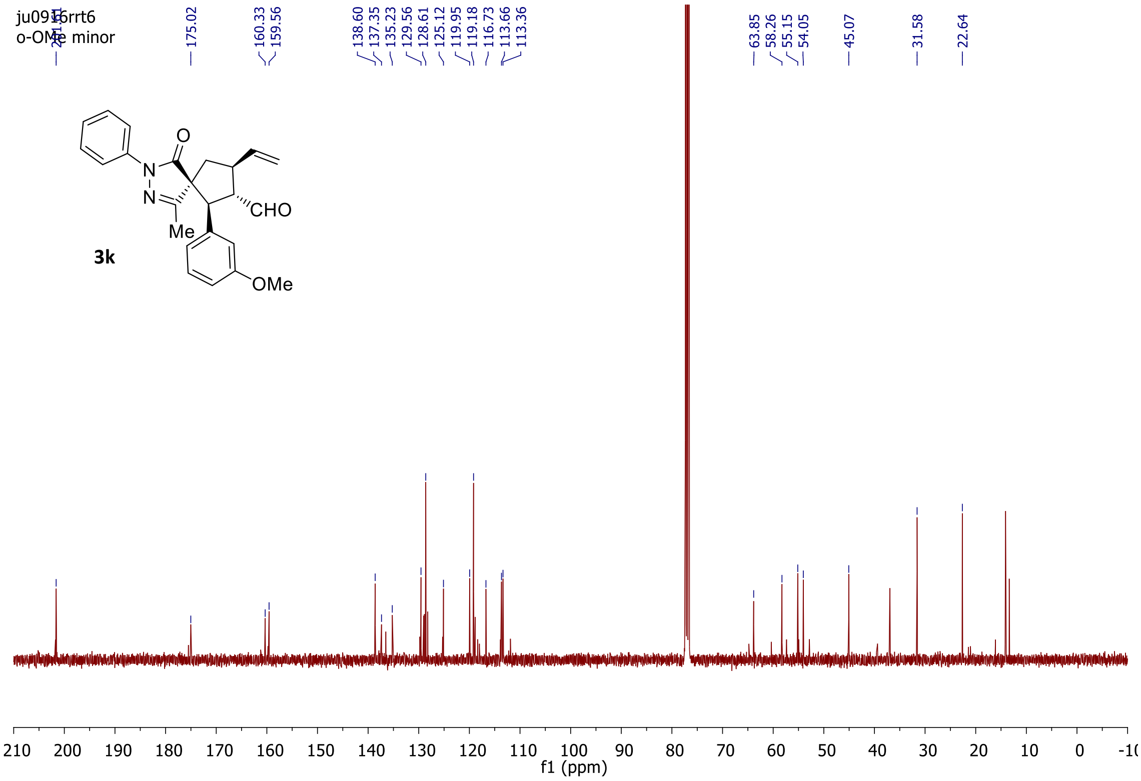

o-OMe minor
175.02
160.33
159.56
138.60
137.35
135.23
129.56
128.61
125.12
119.95
119.18
116.73
113.66
113.36
63.85
58.26
55.15
54.05
45.07
31.58
22.64
O
N
N
CHO
Me
3k
OMe
210
200
190
180
170
160
150
140
130
120
110
100
90
80
70
60
50
40
30
20
10
0
f1 (ppm)

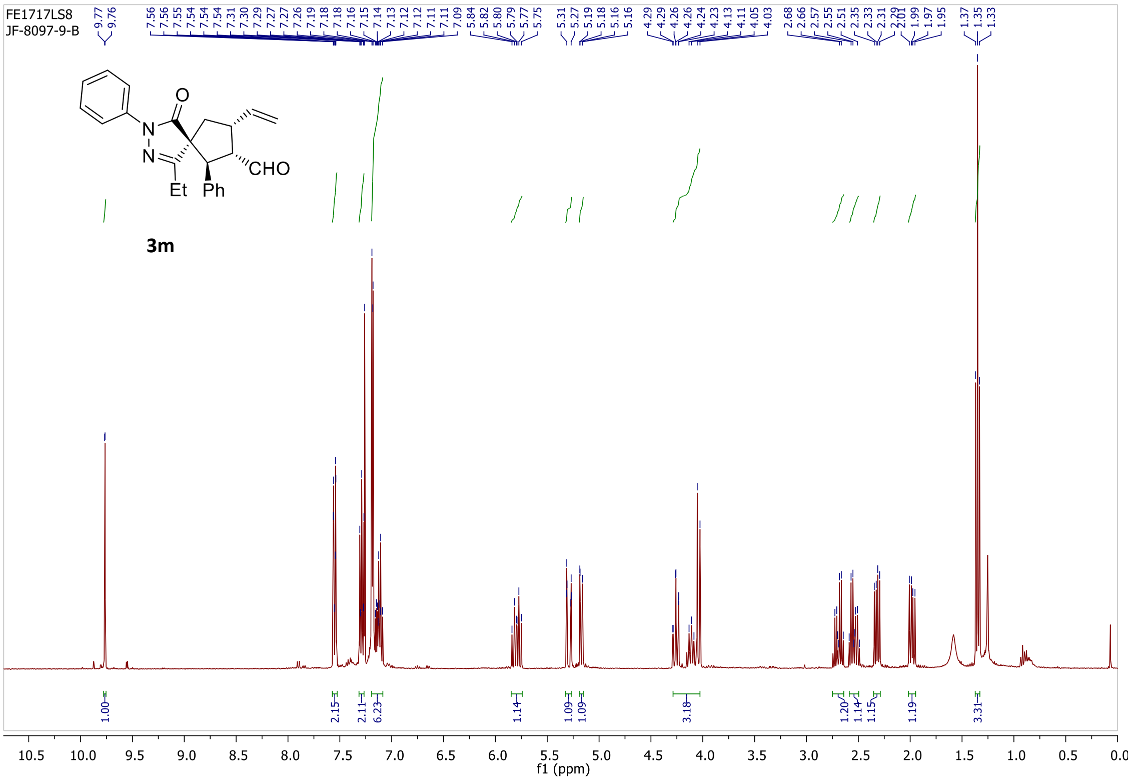
FE1717LS8
JF-8097-9-B
O
N
N
N
CHO
Et
Ph
3m
f1 (ppm)

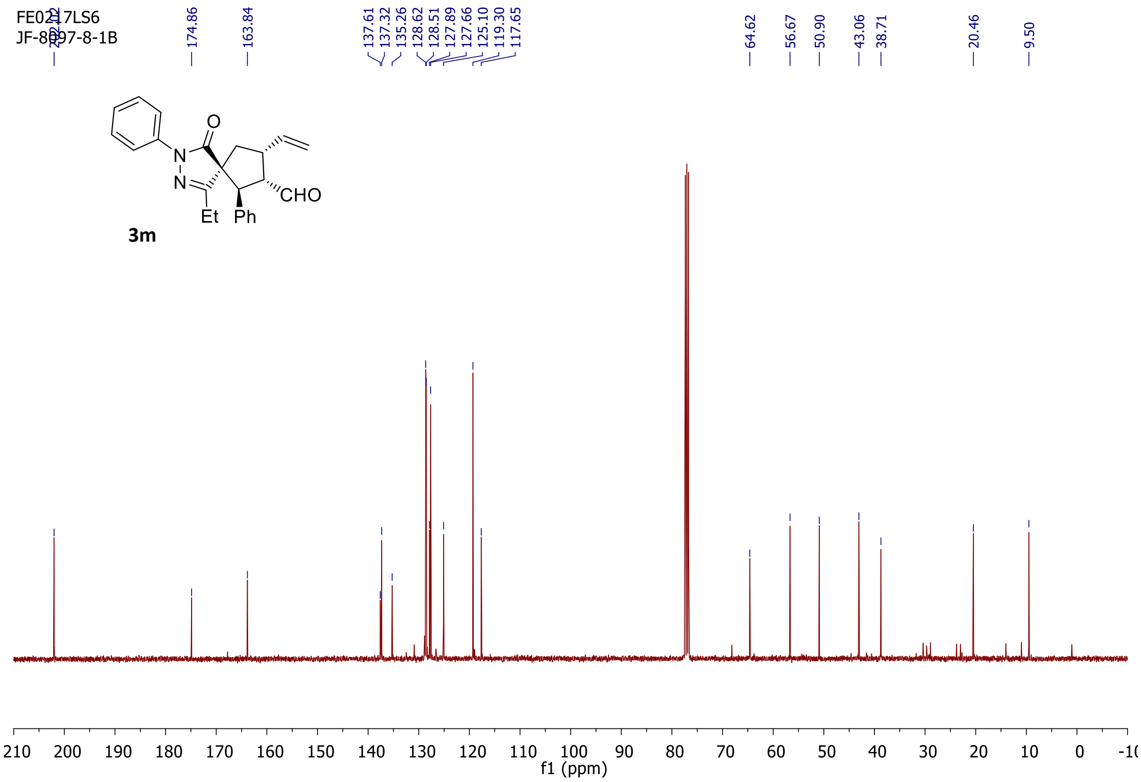
FE0217LS6
JF-8097-8-1B
174.86
163.84
137.61
137.32
135.26
128.62
128.51
127.89
127.66
125.10
119.30
117.65
64.62
56.67
50.90
43.06
38.71
20.46
9.50
O
N
N
N
CHO
Et
Ph
3m
f1 (ppm)

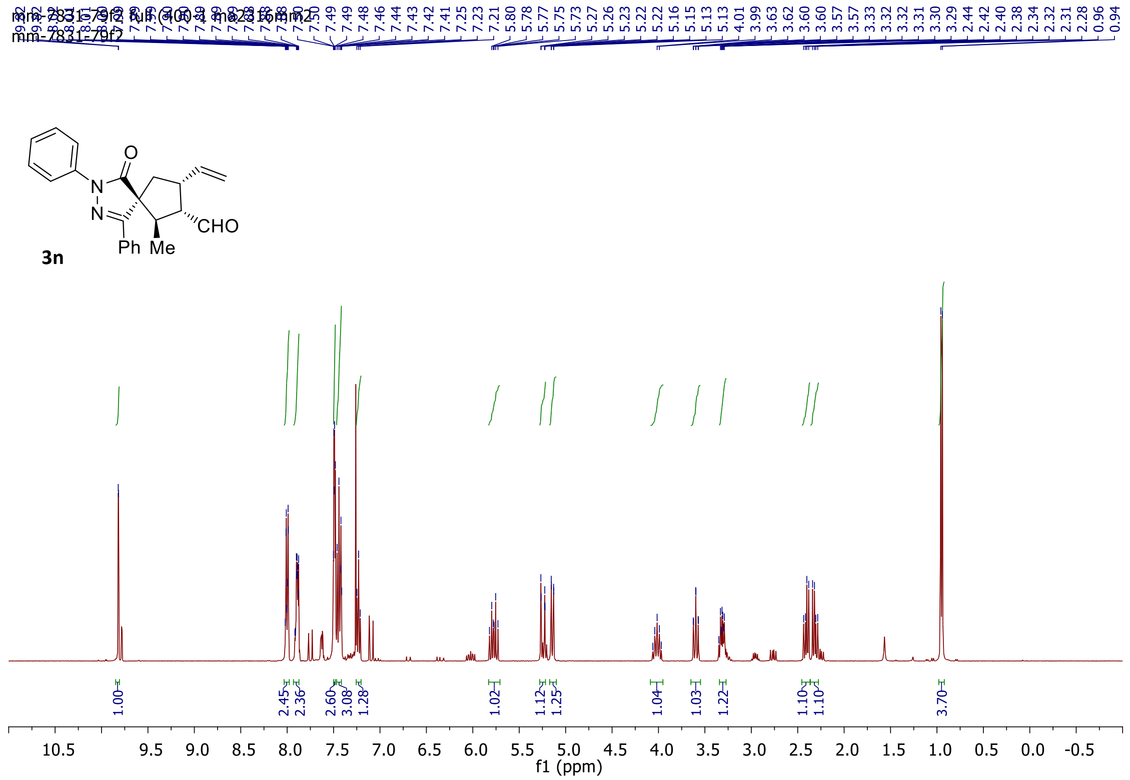
mm-7831-79f2 full (400-1 ma2316mm2
mm-7831-79f2
7.50 7.49 7.49 7.48 7.46 7.44 7.43 7.42 7.41 7.25 7.23 7.21 5.80 5.78 5.77 5.75 5.73 5.27 5.26 5.23 5.22 5.22 5.16 5.15 5.13 5.13 4.01 3.99 3.63 3.62 3.60 3.60 3.57 3.57 3.33 3.32 3.32 3.31 3.30 3.29 2.44 2.42 2.40 2.38 2.34 2.32 2.31 2.28 0.96 0.94
O
N
N
CHO
Ph
Me
3n
1.00 2.45 2.36 2.60 3.08 1.28 1.02 1.12 1.25 1.04 1.03 1.22 1.10 1.10 3.70
10.5 9.5 9.0 8.5 8.0 7.5 7.0 6.5 6.0 5.5 5.0 4.5 4.0 3.5 3.0 2.5 2.0 1.5 1.0 0.5 0.0 -0.5
f1 (ppm)

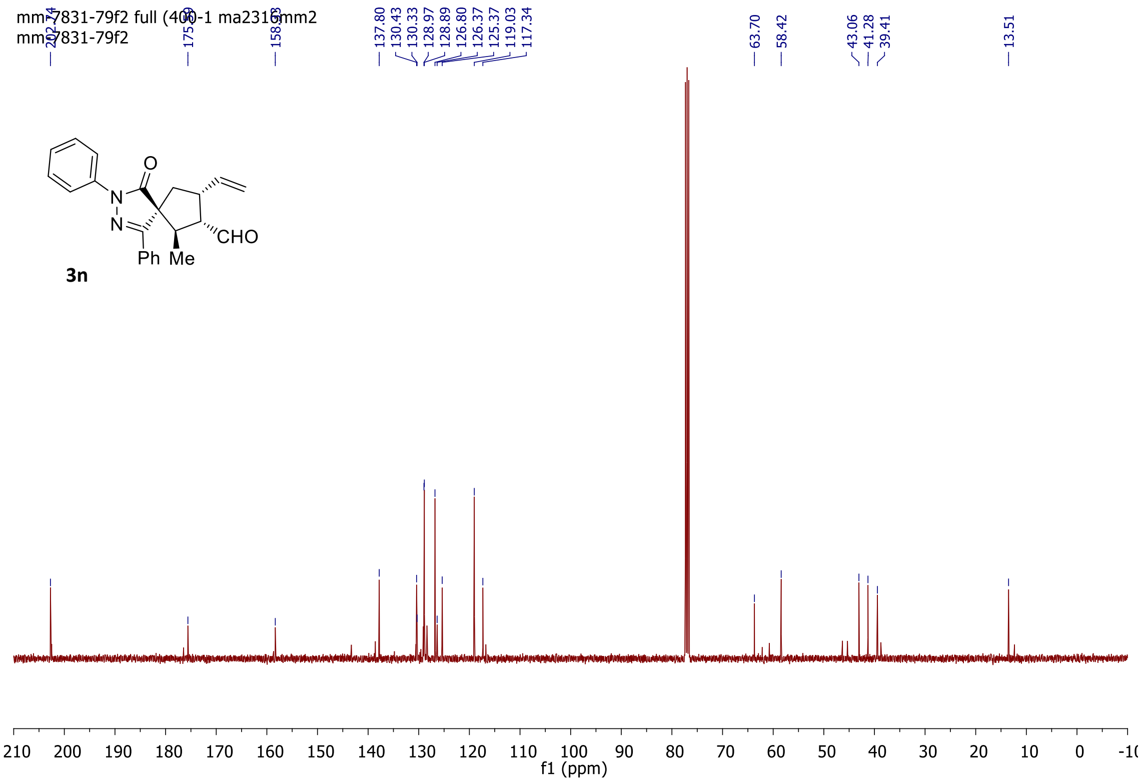
mm-7831-79f2 full (400-1 ma2316mm2
mm-7831-79f2
202.74
137.80 130.43 130.33 128.97 128.89 126.80 126.37 125.37 119.03 117.34
63.70 58.42
43.06 41.28 39.41
13.51
O
N
N
CHO
Ph
Me
3n
210 200 190 180 170 160 150 140 130 120 110 100 90 80 70 60 50 40 30 20 10 0
f1 (ppm)

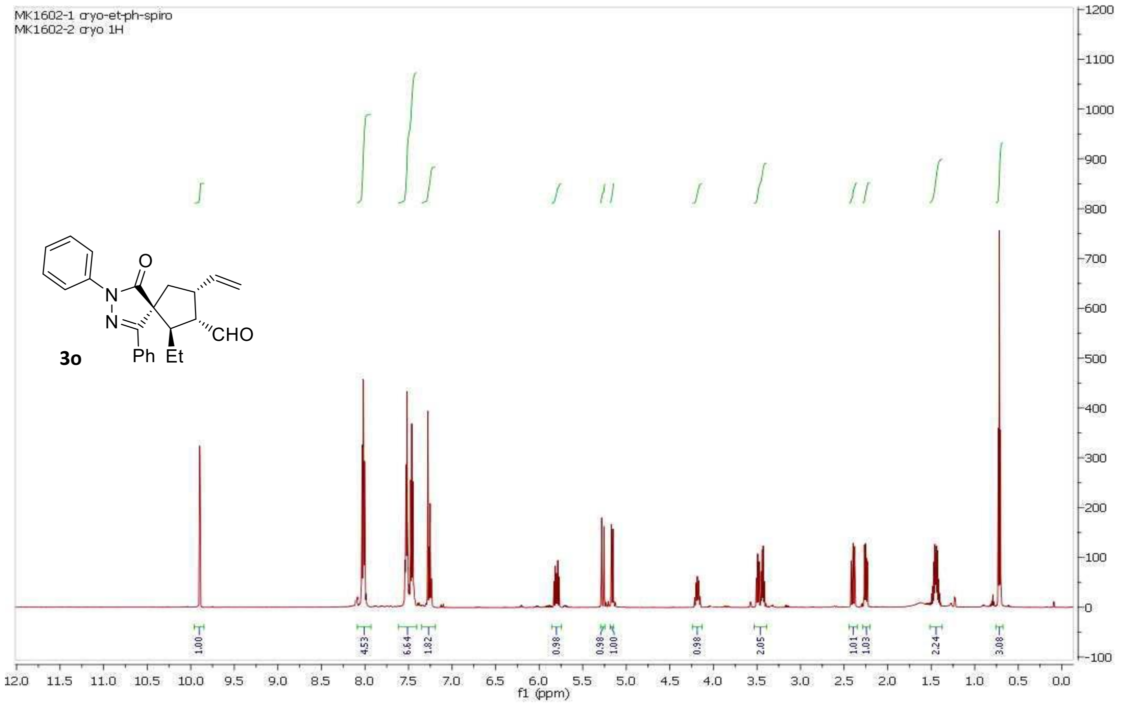
MK1602-1 cryo-et-ph-spiro
MK1602-2 cryo 1H
3o
Ph Et
CHO
1.00
4.53
6.64
1.82
0.98
0.98
1.00
0.98
2.05
1.01
1.03
2.24
3.08
f1 (ppm)

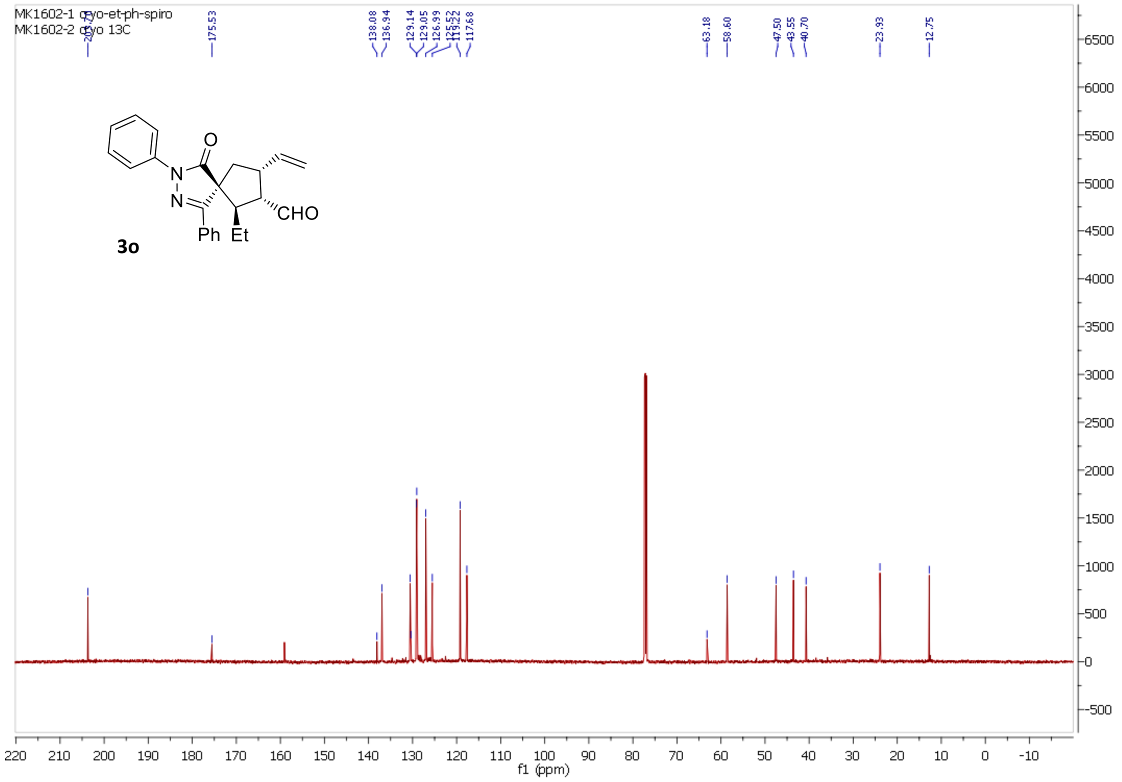
MK1602-1 cryo-et-ph-spiro
MK1602-2 cryo 13C
175.53
138.08
136.94
129.14
129.05
126.99
117.68
63.18
58.60
47.50
43.55
40.70
23.93
12.75
3o
Ph Et
CHO
f1 (ppm)

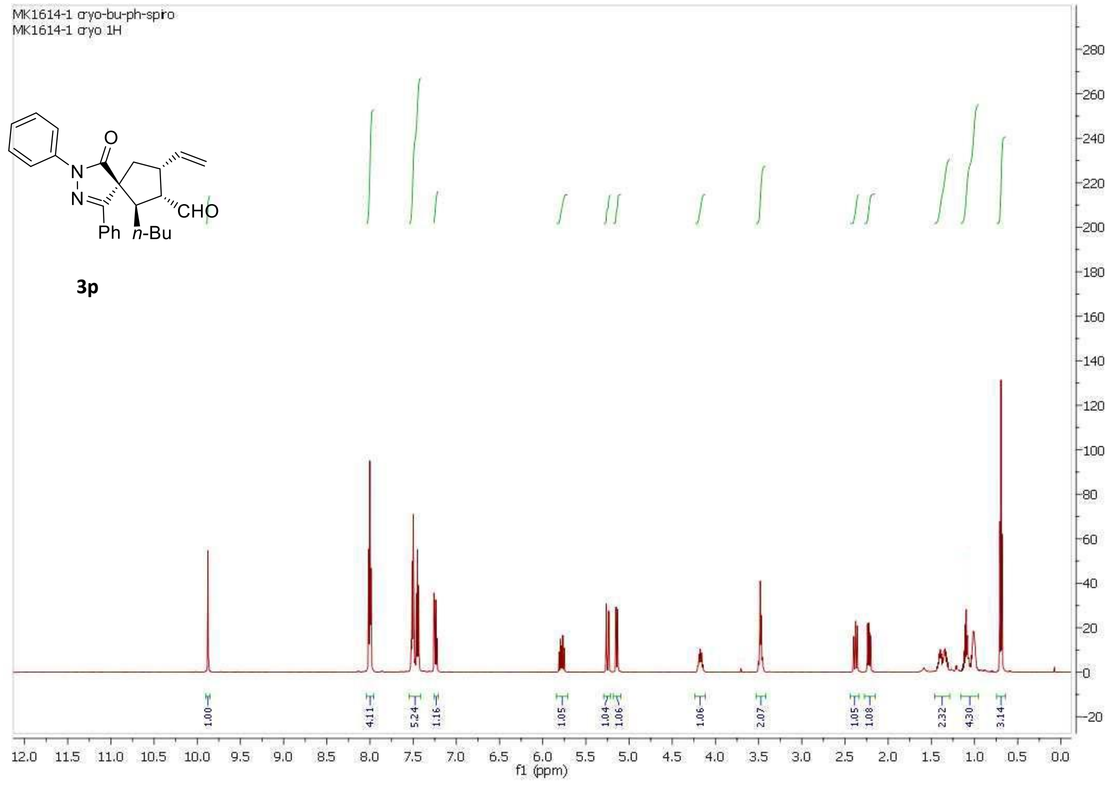

MK1614-1 cryo-bu-ph-spiro
MK1614-1 cryo 1H
3p
Ph
n-Bu
CHO
1.00
4.11
5.24
1.16
1.05
1.04
1.06
1.06
2.07
1.05
1.08
2.32
4.30
3.14
f1 (ppm)

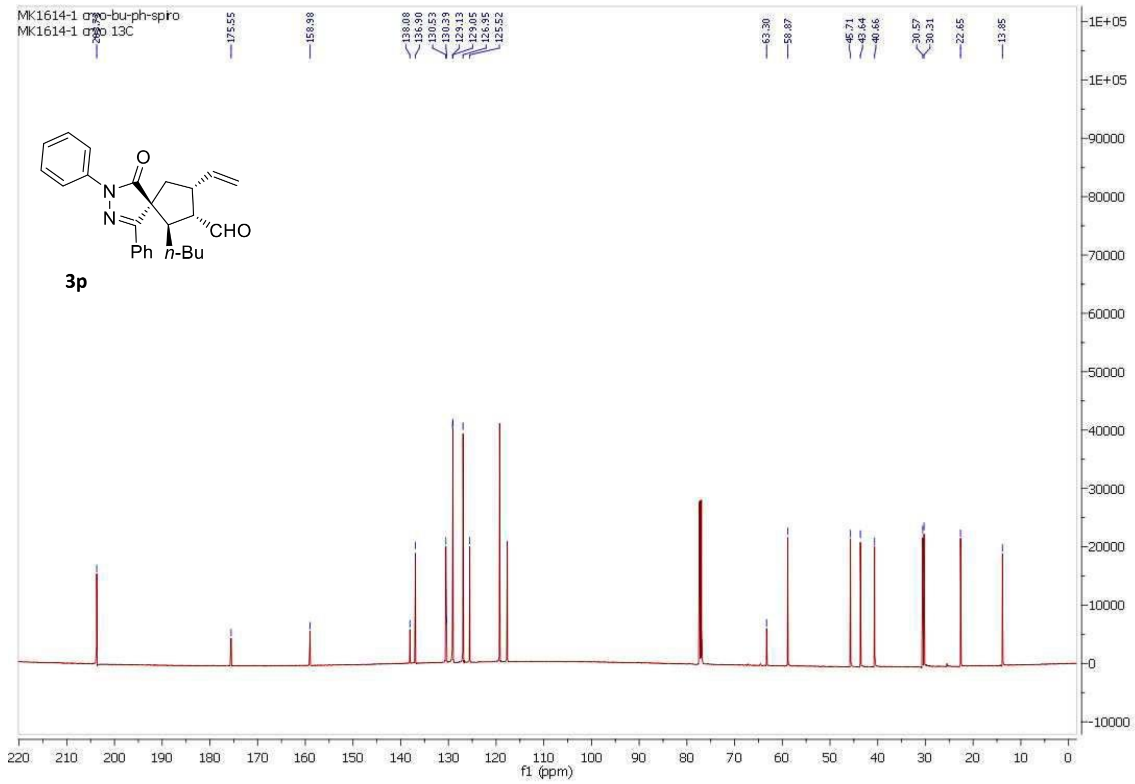

MK1614-1 cryo-bu-ph-spiro
MK1614-1 cryo 13C
175.55
158.98
138.08
136.90
130.53
130.39
129.13
129.05
126.95
125.52
63.30
58.87
45.71
43.64
40.66
30.57
30.31
22.65
13.85
3p
Ph
n-Bu
CHO
f1 (ppm)

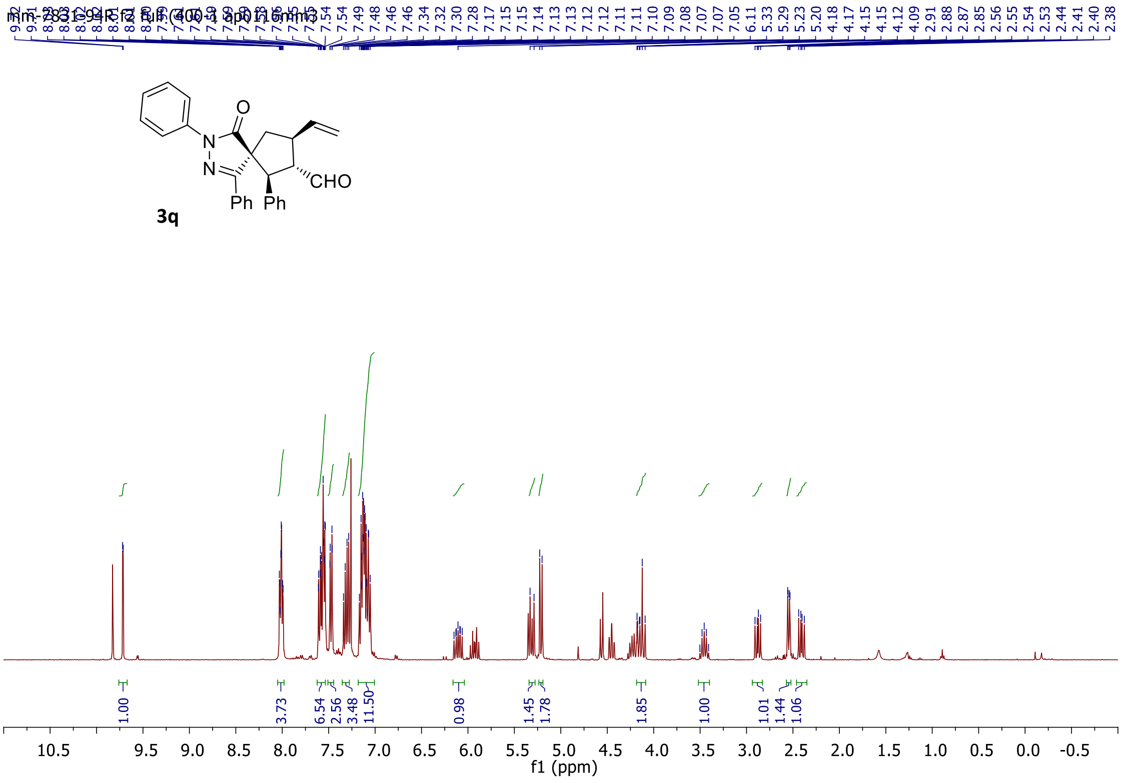
mm-7831-94R f2 full (400-1 ap0116mm3
7.54
7.49
7.48
7.46
7.46
7.34
7.32
7.30
7.28
7.17
7.15
7.15
7.14
7.13
7.13
7.12
7.12
7.11
7.11
7.10
7.09
7.08
7.07
7.07
7.05
6.11
5.33
5.29
5.23
5.20
4.18
4.17
4.15
4.15
4.12
4.09
2.91
2.88
2.87
2.85
2.56
2.55
2.54
2.53
2.44
2.41
2.40
2.38
O
N
N
CHO
Ph
Ph
3q
1.00
3.73
6.54
2.56
3.48
11.50
0.98
1.45
1.78
1.85
1.00
1.01
1.44
1.06
f1 (ppm)

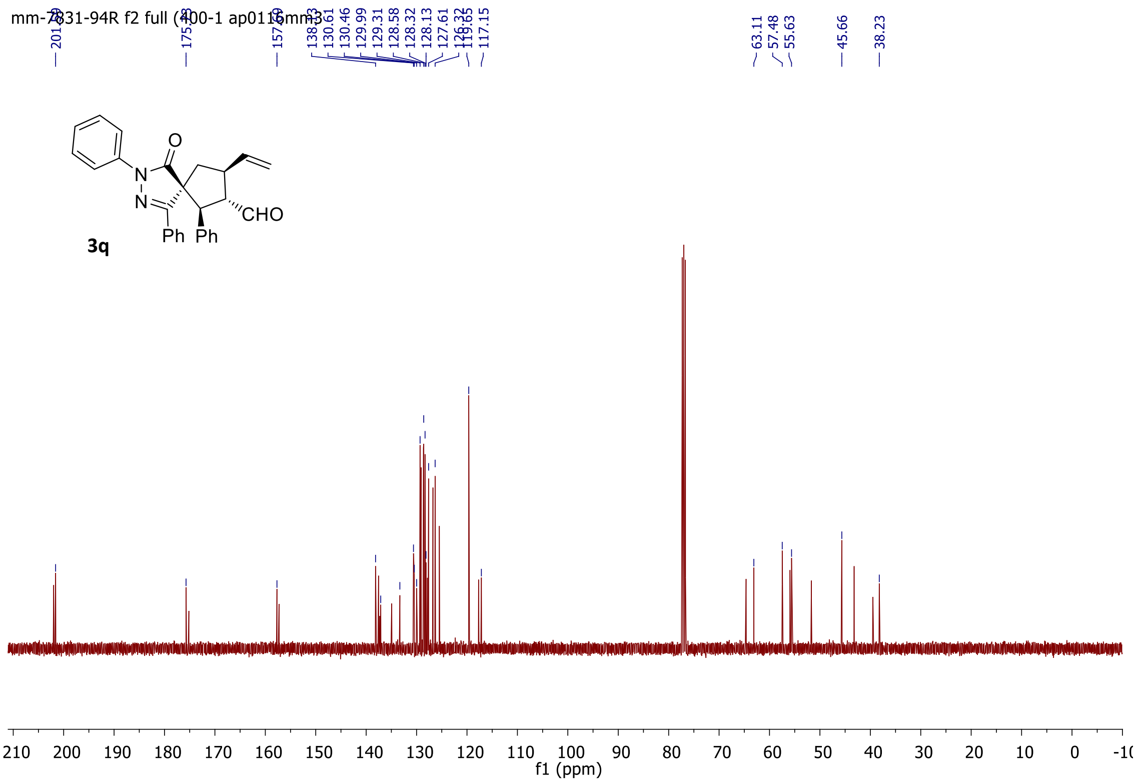
mm-7831-94R f2 full (400-1 ap0116mm3
130.61
130.46
129.99
129.31
128.58
128.32
128.13
127.61
126.32
119.65
117.15
63.11
57.48
55.63
45.66
38.23
O
N
N
CHO
Ph
Ph
3q
f1 (ppm)

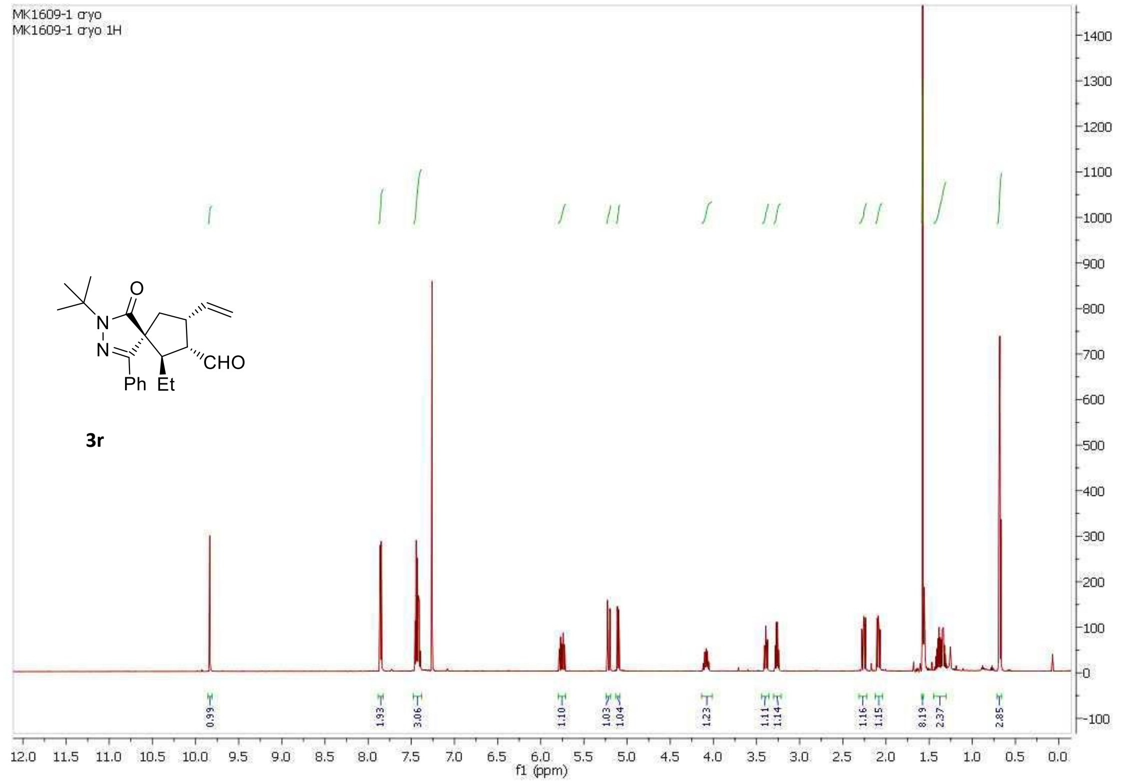
MK1609-1 cryo
MK1609-1 cryo 1H
3r
CHO
Ph
Et
f1 (ppm)
0.99
1.93
3.06
1.10
1.03
1.04
1.23
1.11
1.14
1.16
1.15
8.19
2.37
2.85

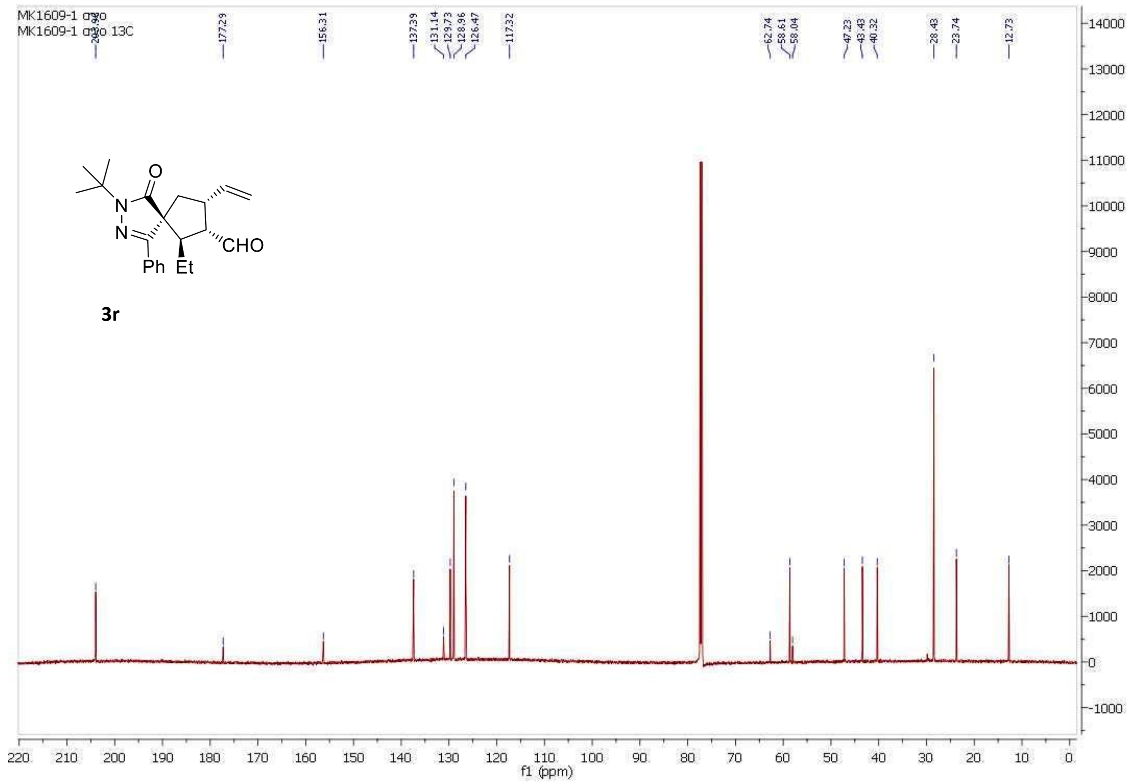
MK1609-1 cryo
MK1609-1 cryo 13C
177.29
156.31
137.39
131.14
129.73
128.96
126.47
117.32
62.74
58.61
58.04
47.23
43.43
40.32
28.43
23.74
12.73
3r
CHO
Ph
Et
f1 (ppm)

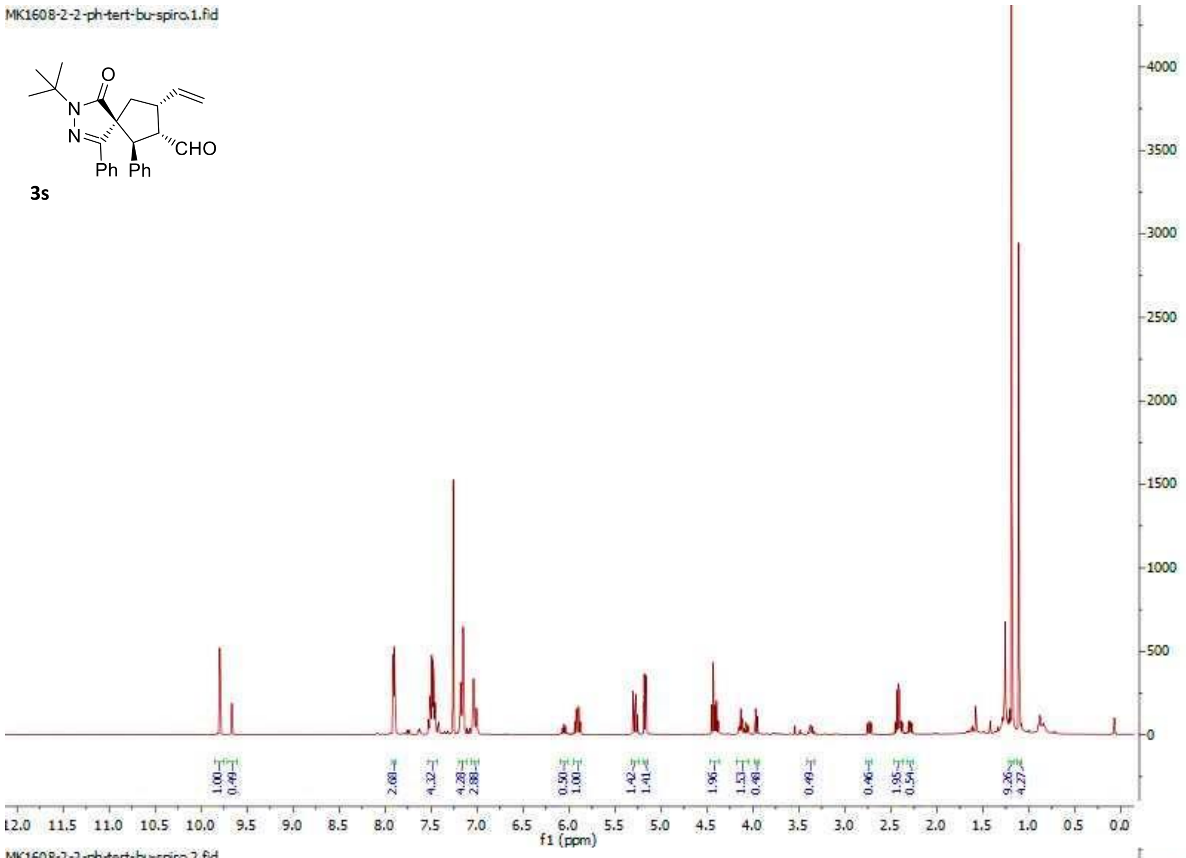
MK1608-2-2-ph-tert-bu-spiro.1.fid
3s
f1 (ppm)

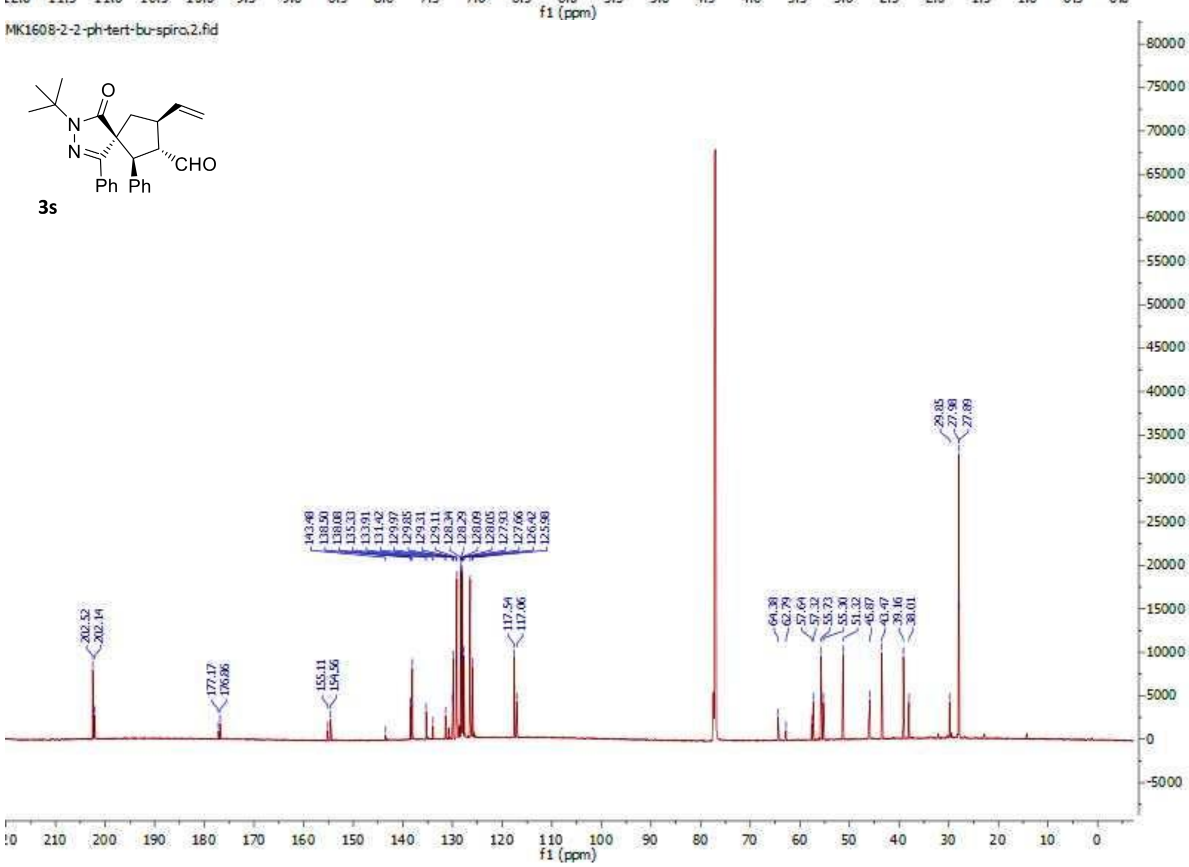
MK1608-2-2-ph-tert-bu-spiro.2.fid
3s
f1 (ppm)

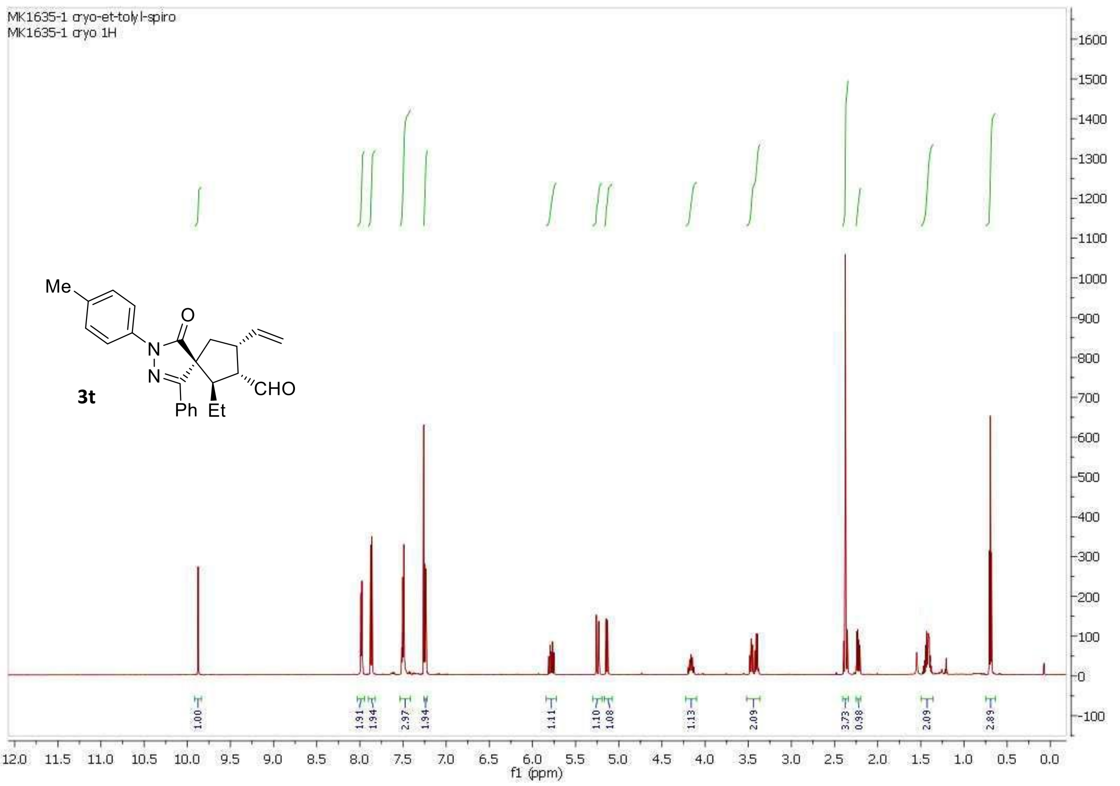
MK1635-1 cryo-et-tolyl-spiro
MK1635-1 cryo 1H
Me
O
N
N
CHO
3t
Ph
Et
1.00
1.91
1.94
2.97
1.94
1.11
1.10
1.08
1.13
2.09
3.73
0.98
2.09
2.89
f1 (ppm)

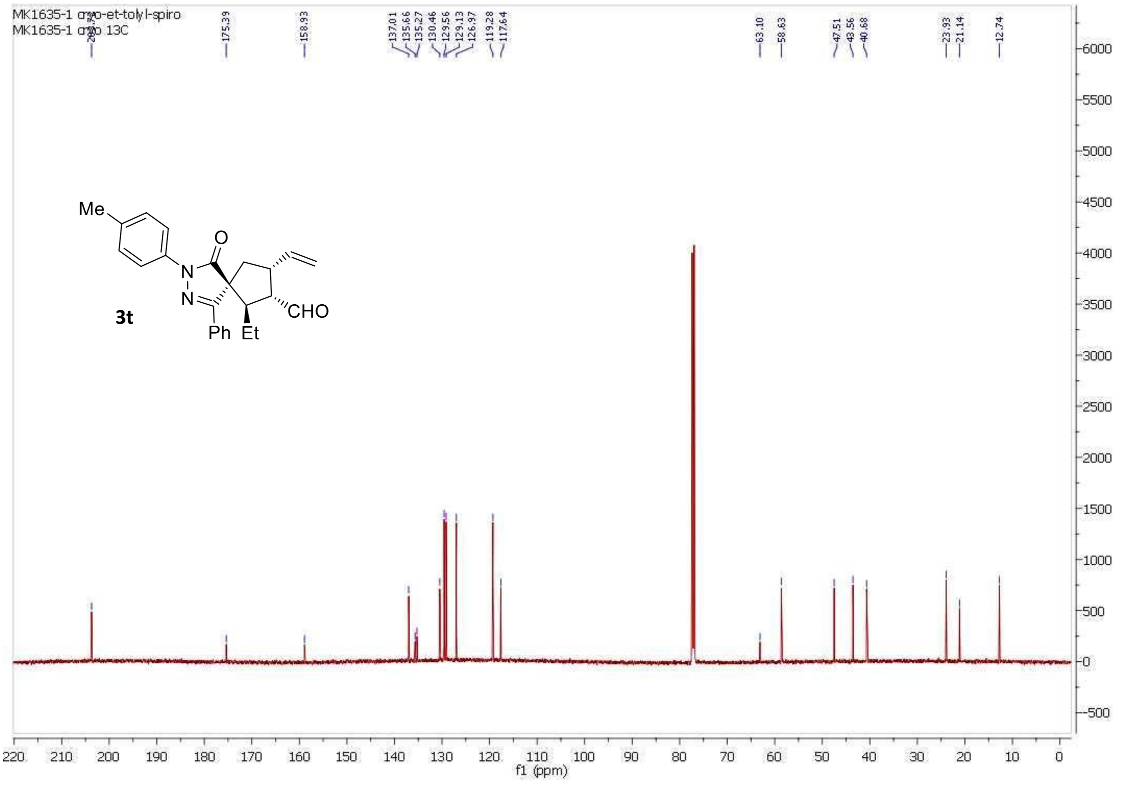
MK1635-1 cryo-et-tolyl-spiro
MK1635-1 cryo 13C
175.39
158.93
137.01
135.66
135.27
130.46
129.56
129.13
126.97
119.28
117.64
63.10
58.63
47.51
43.56
40.68
23.93
21.14
12.74
Me
O
N
N
CHO
3t
Ph
Et
f1 (ppm)

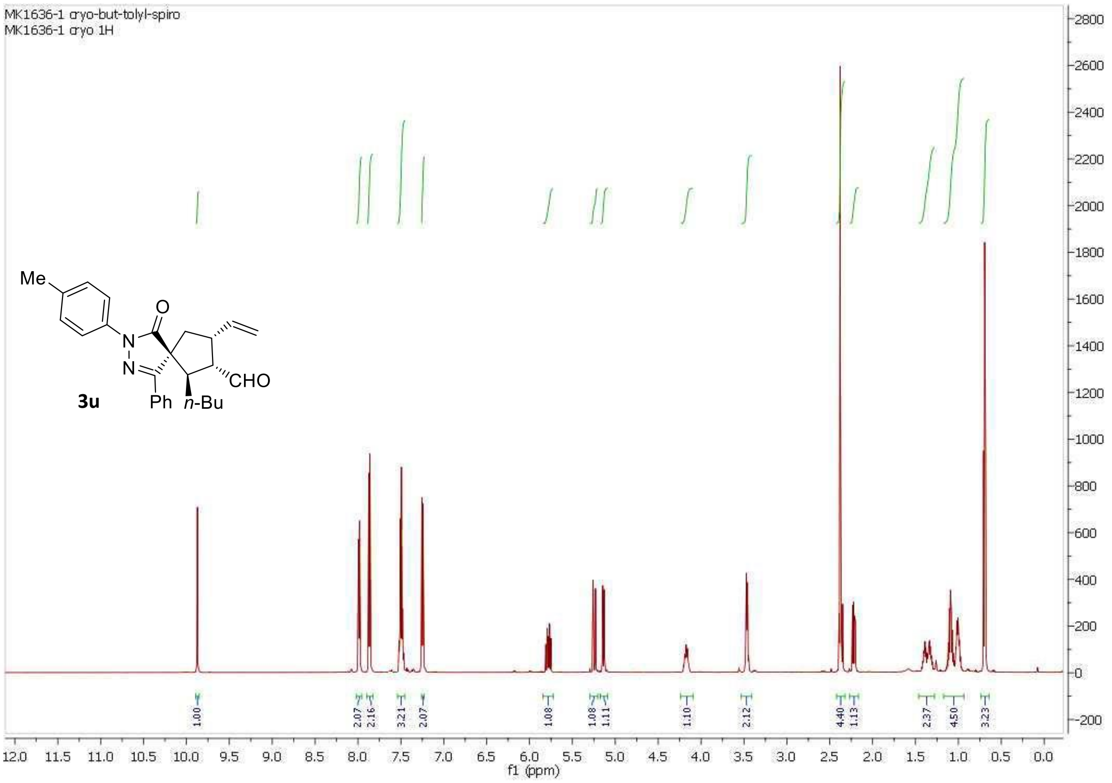
MK1636-1 cryo-but-tolyl-spiro
MK1636-1 cryo 1H
Me
O
N
N
CHO
3u
Ph
n-Bu
1.00
2.07
2.16
3.21
2.07
1.08
1.08
1.11
1.10
2.12
4.40
1.13
2.37
4.50
3.23
f1 (ppm)

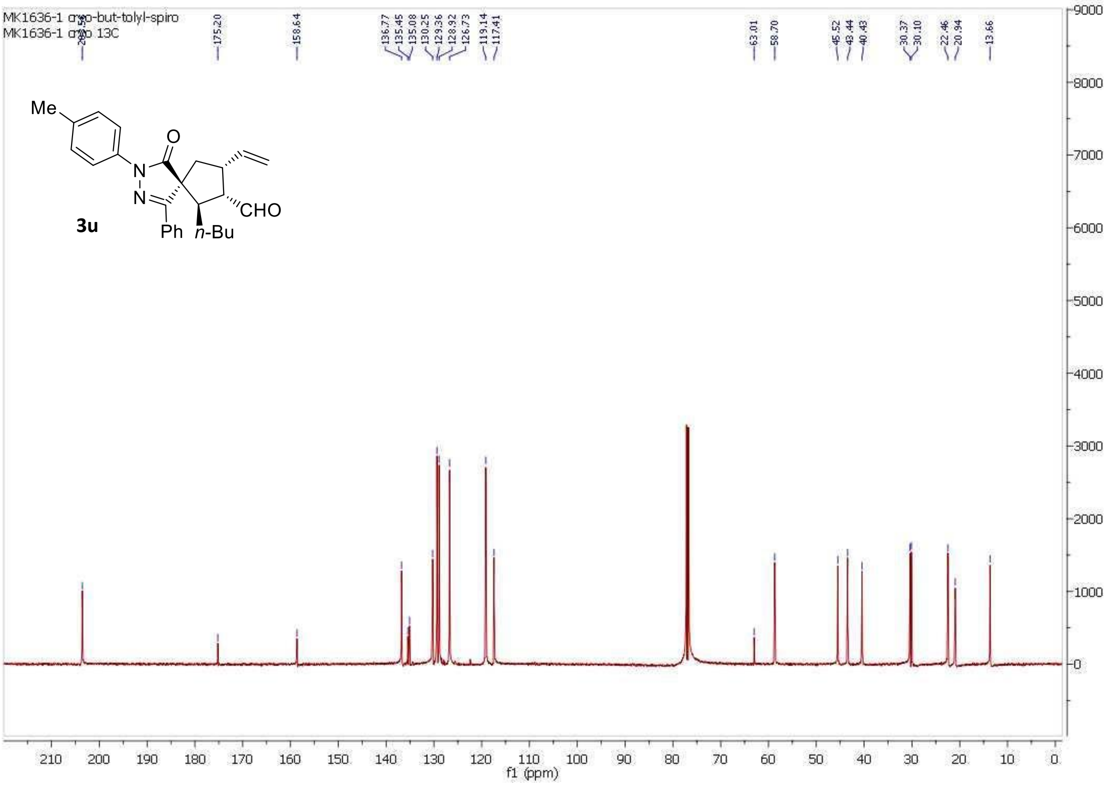
MK1636-1 cryo-but-tolyl-spiro
MK1636-1 cryo 13C
175.20
158.64
136.77
135.45
135.08
130.25
129.36
128.92
126.73
119.14
117.41
63.01
58.70
45.52
43.44
40.43
30.37
30.10
22.46
20.94
13.66
Me
O
N
N
CHO
3u
Ph
n-Bu
f1 (ppm)

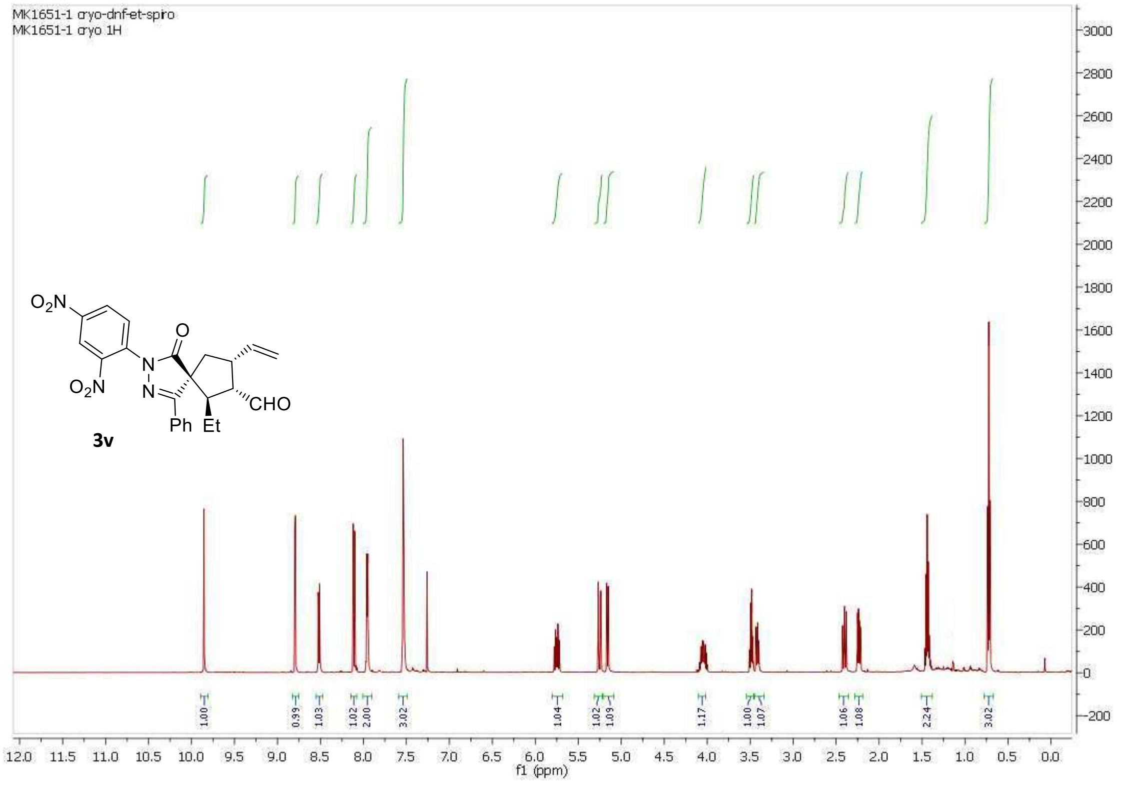
MK1651-1 cryo-dnf-et-spiro
MK1651-1 cryo 1H
3v
1.00
0.99
1.03
1.02
2.00
3.02
1.04
1.02
1.09
1.17
1.00
1.07
1.06
1.08
2.24
3.02
f1 (ppm)

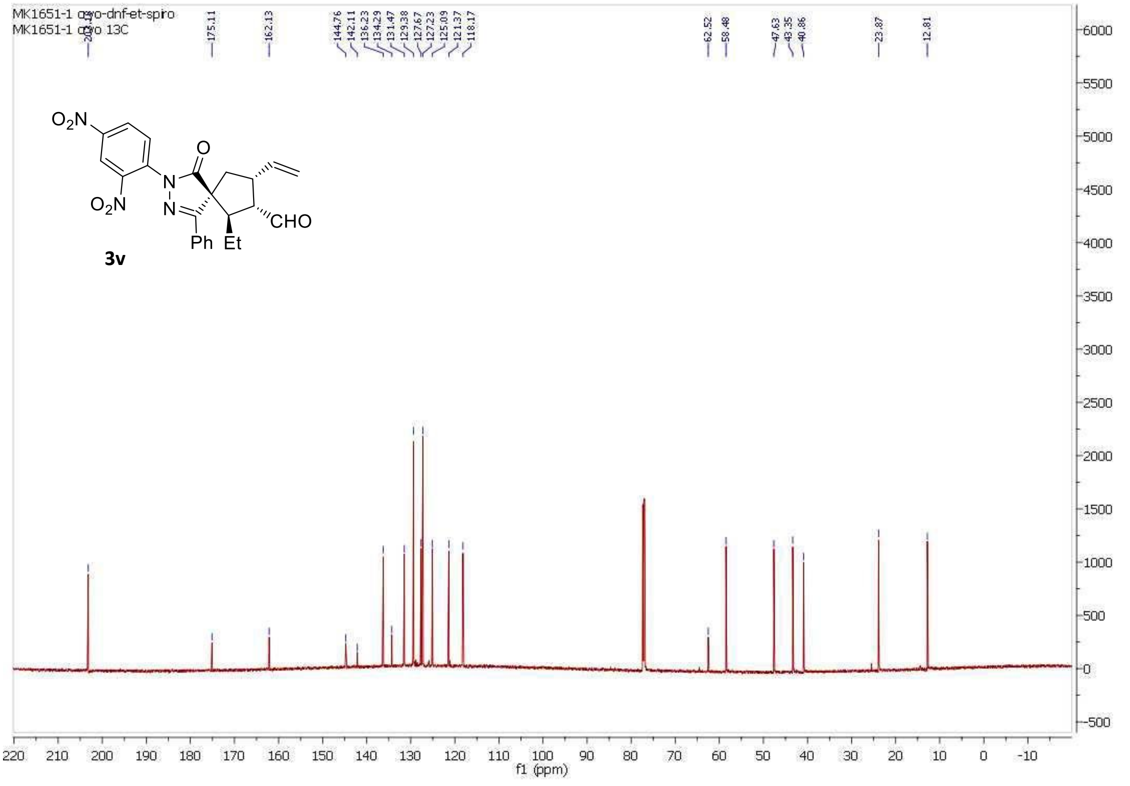
MK1651-1 cryo-dnf-et-spiro
MK1651-1 cryo 13C
175.11
162.13
144.76
142.11
136.23
134.29
131.47
129.38
127.67
127.23
125.09
121.37
118.17
62.52
58.48
47.63
43.35
40.86
23.87
12.81
3v
f1 (ppm)

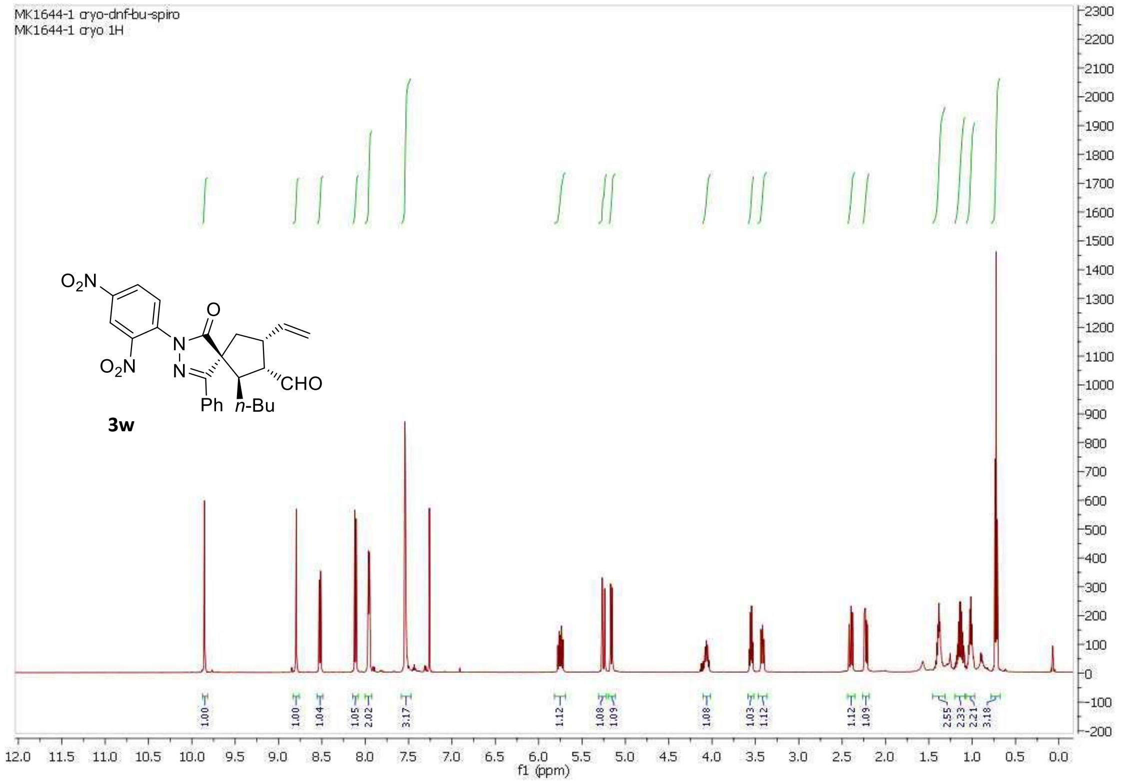
MK1644-1 cryo-dnf-bu-spiro
MK1644-1 cryo 1H
3w
1.00
1.00
1.04
1.05
2.02
3.17
1.12
1.08
1.09
1.08
1.03
1.12
1.12
1.09
2.55
2.33
2.21
3.18
f1 (ppm)

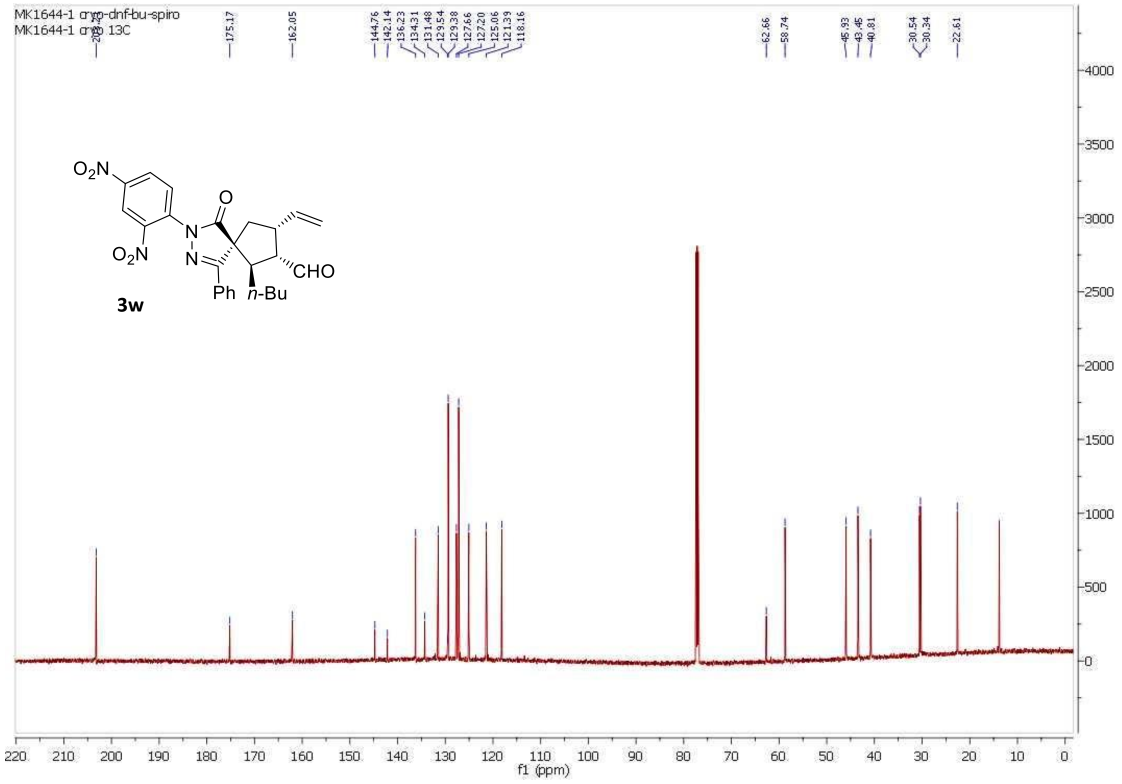
MK1644-1 cryo-dnf-bu-spiro
MK1644-1 cryo 13C
175.17
162.05
144.76
142.14
136.23
134.31
131.48
129.54
129.38
127.66
127.20
125.06
121.39
118.16
62.66
58.74
45.93
43.46
40.81
30.54
30.34
22.61
3w
f1 (ppm)

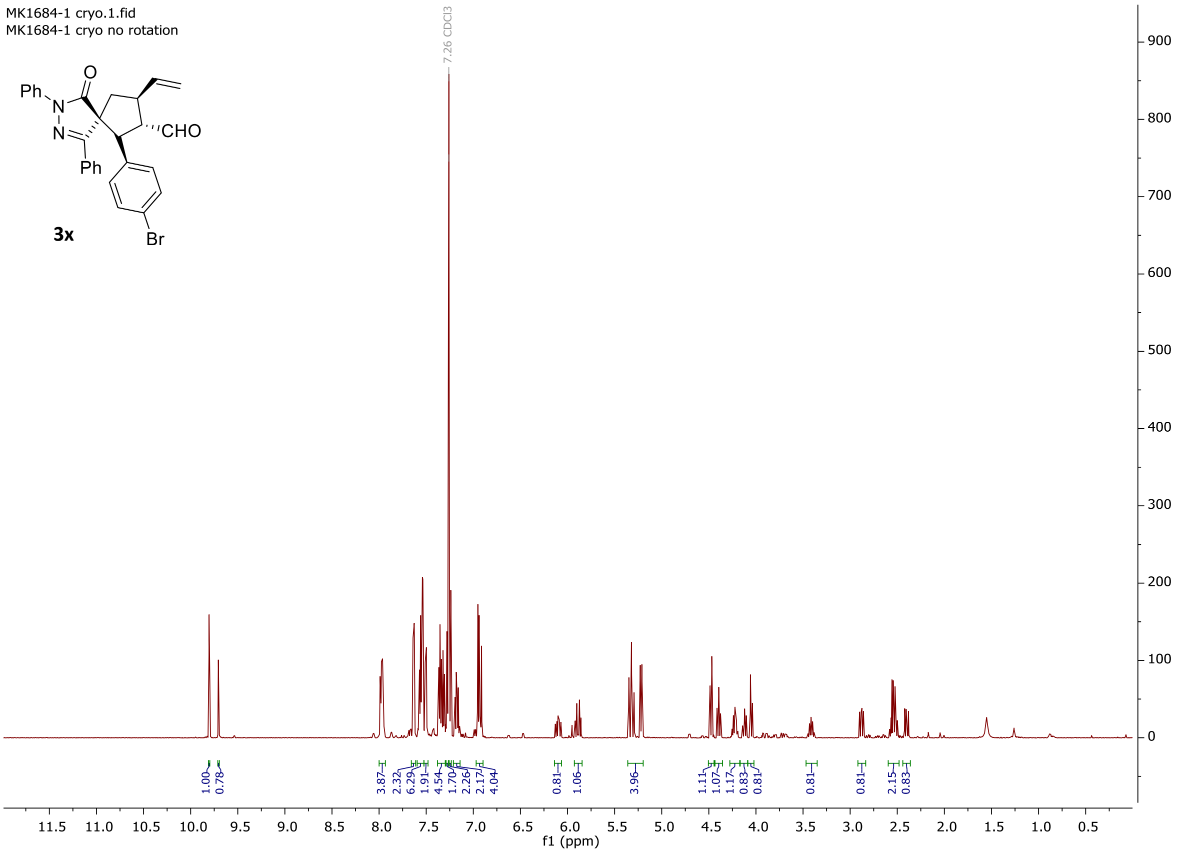
MK1684-1 cryo.1.fid
MK1684-1 cryo no rotation
Ph
O
N
N
CHO
Ph
3x
Br
7.26 CDCl3
1.00
0.78
3.87
2.32
6.29
1.91
4.54
1.70
2.26
2.17
4.04
0.81
1.06
3.96
1.11
1.07
1.17
0.83
0.81
0.81
0.81
2.15
0.83
f1 (ppm)

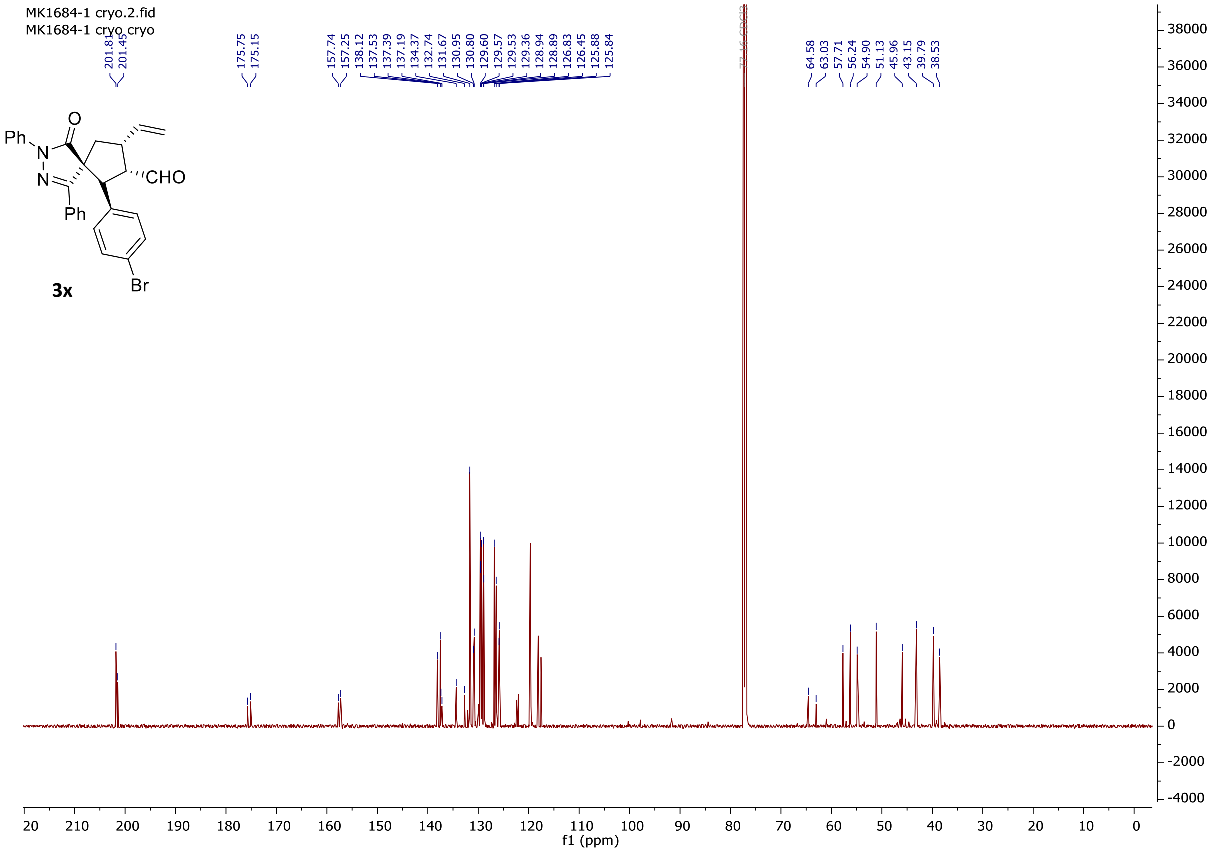
MK1684-1 cryo.2.fid
MK1684-1 cryo cryo
201.81
201.45
175.75
175.15
157.74
157.25
138.12
137.53
137.39
137.19
134.37
132.74
131.67
130.95
130.80
129.60
129.57
129.53
129.36
128.94
128.89
126.83
126.45
125.88
125.84
64.58
63.03
57.71
56.24
54.90
51.13
45.96
43.15
39.79
38.53
Ph
O
N
N
CHO
Ph
3x
Br
f1 (ppm)

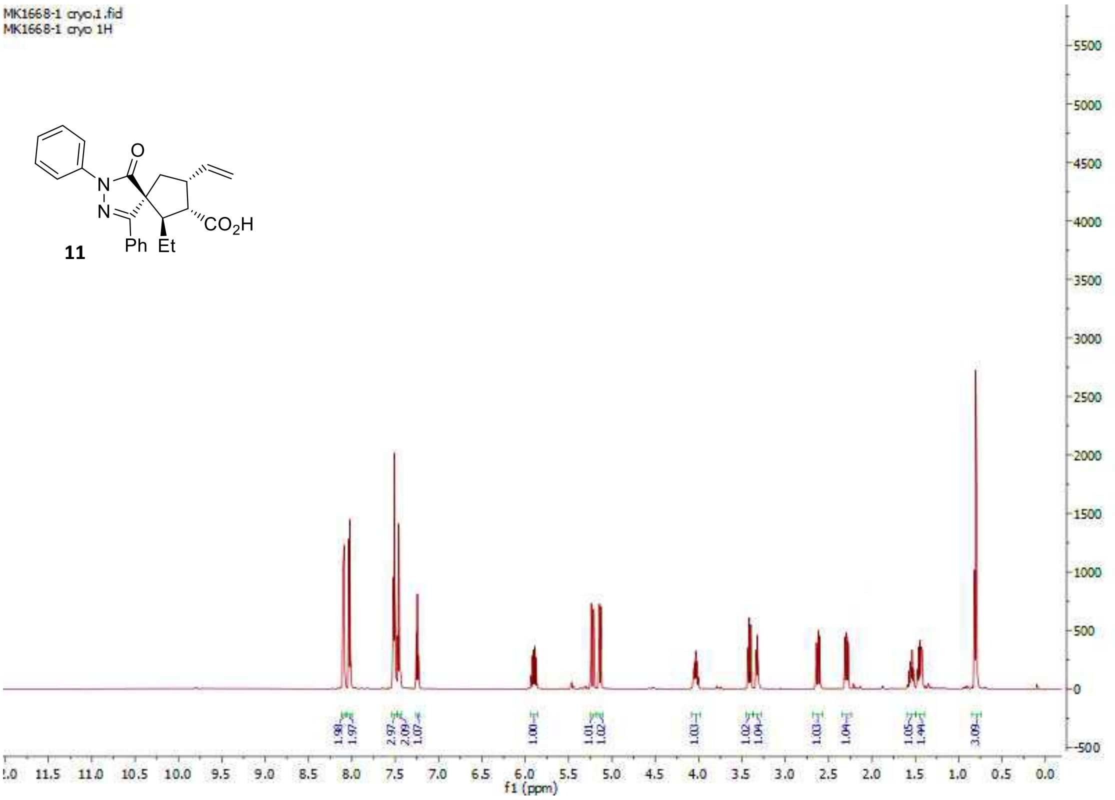
MK1668-1 cryo.1.fid
MK1668-1 cryo 1H
11
Ph Et
CO2H
f1 (ppm)

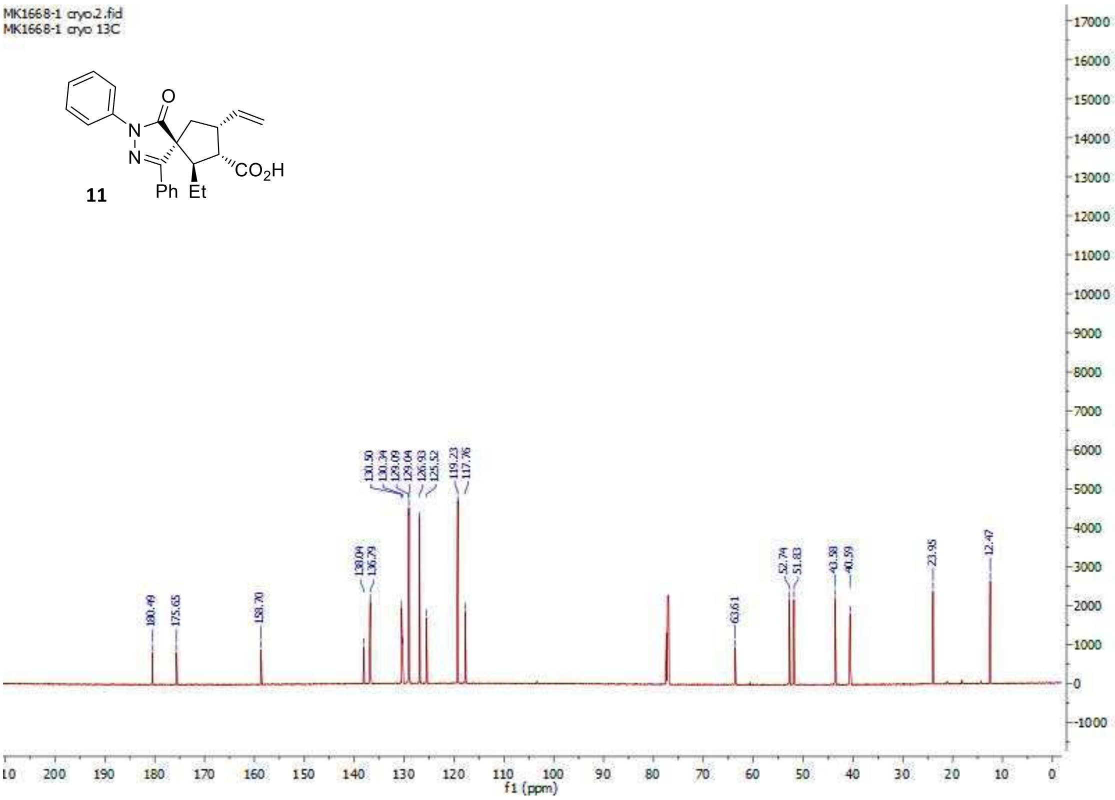
MK1668-1 cryo.2.fid
MK1668-1 cryo 13C
11
Ph Et
CO2H
180.49
175.65
158.70
138.09
136.79
130.50
130.34
129.09
129.04
126.93
125.52
119.23
117.76
63.61
52.74
51.83
43.58
40.59
23.95
12.47
f1 (ppm)

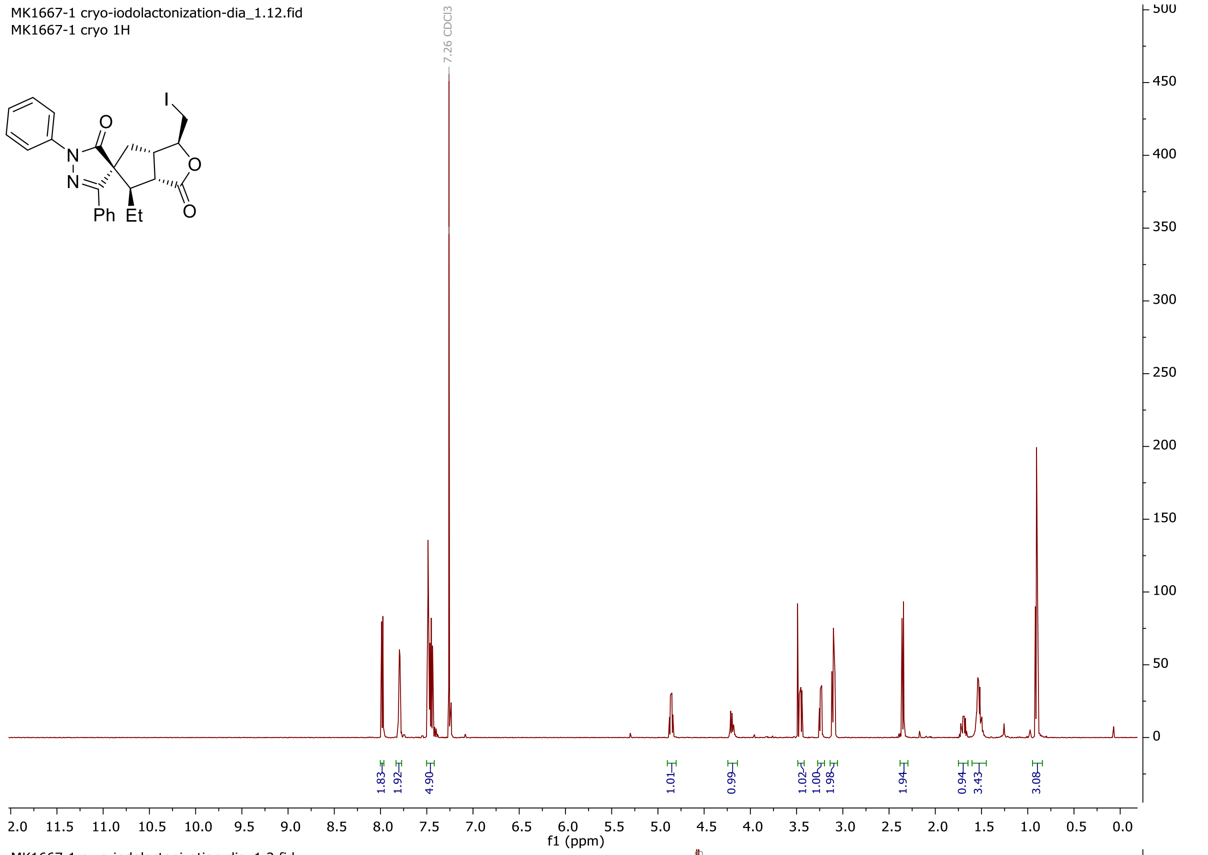
MK1667-1 cryo-iodolactonization-dia_1.12.fid
MK1667-1 cryo 1H
7.26 CDCl3
1.83
1.92
4.90
1.01
0.99
1.02
1.00
1.98
1.94
0.94
3.43
3.08
f1 (ppm)
O
N
N
Ph
Et
I
O
O

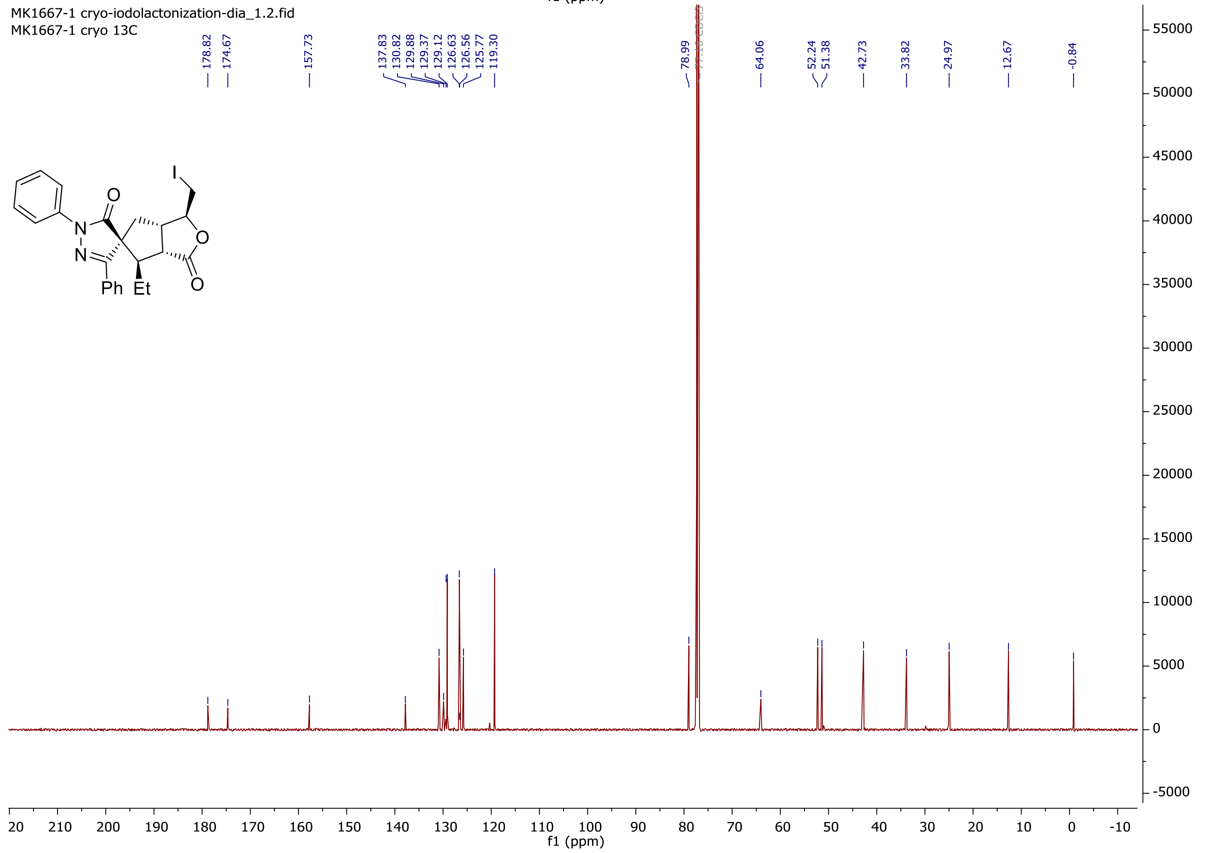
MK1667-1 cryo-iodolactonization-dia_1.2.fid
MK1667-1 cryo 13C
178.82
174.67
157.73
137.83
130.82
129.88
129.37
129.12
126.63
126.56
125.77
119.30
78.99
64.06
52.24
51.38
42.73
33.82
24.97
12.67
-0.84
f1 (ppm)
O
N
N
Ph
Et
I
O
O

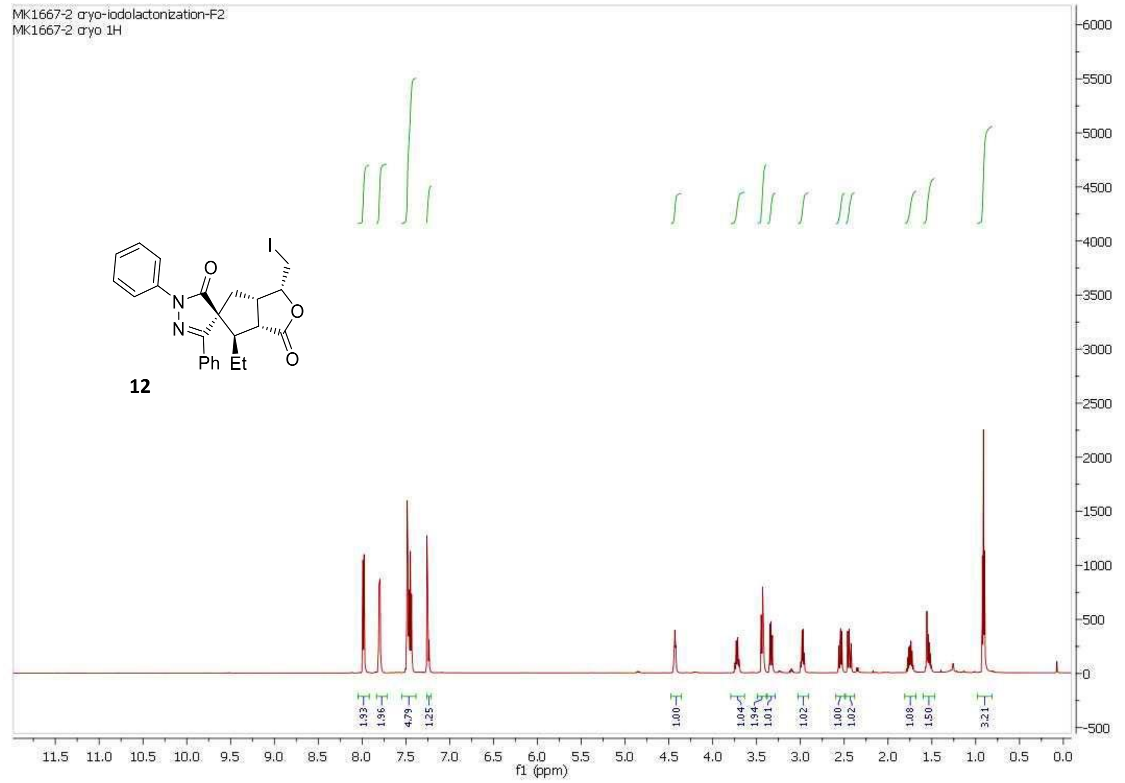
MK1667-2 cryo-iodolactonization-F2
MK1667-2 cryo 1H
12
1.93
1.96
4.79
1.25
1.00
1.04
1.94
1.01
1.02
1.00
1.02
1.08
1.50
3.21
f1 (ppm)

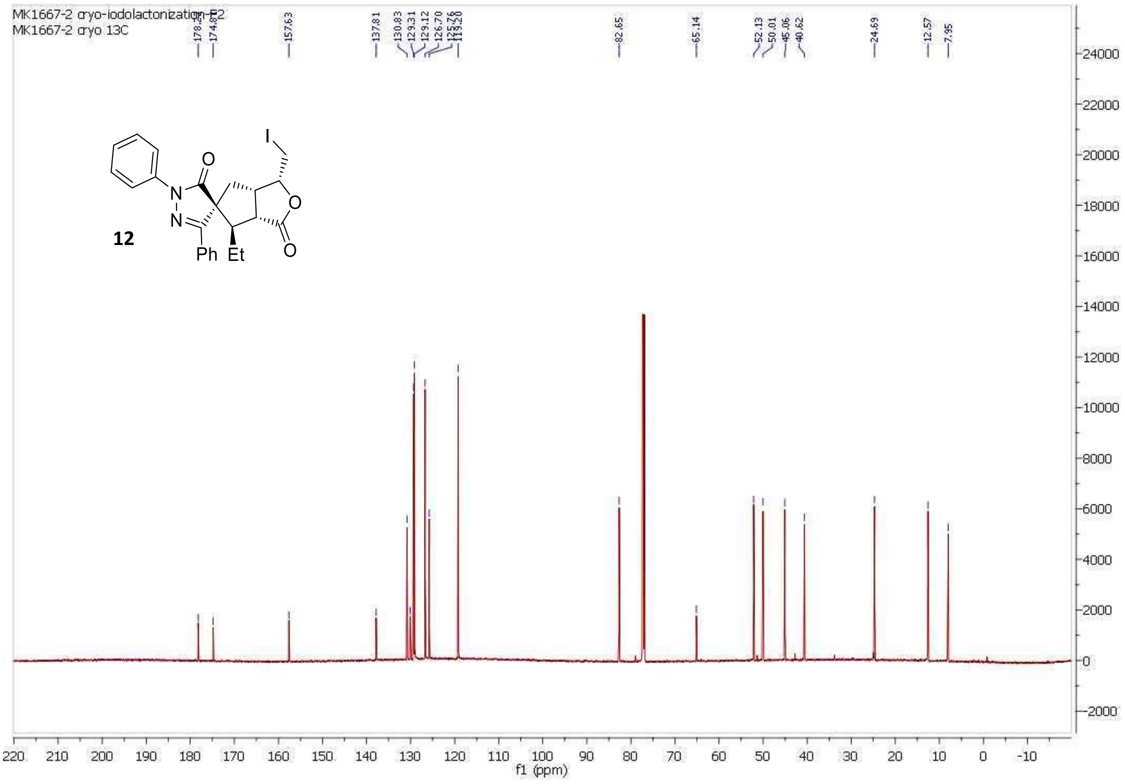
MK1667-2 cryo-iodolactonization-F2
MK1667-2 cryo 13C
157.63
137.81
130.83
129.31
129.12
126.70
119.20
82.65
65.14
52.13
50.01
45.06
40.62
24.69
12.57
7.95
12
f1 (ppm)

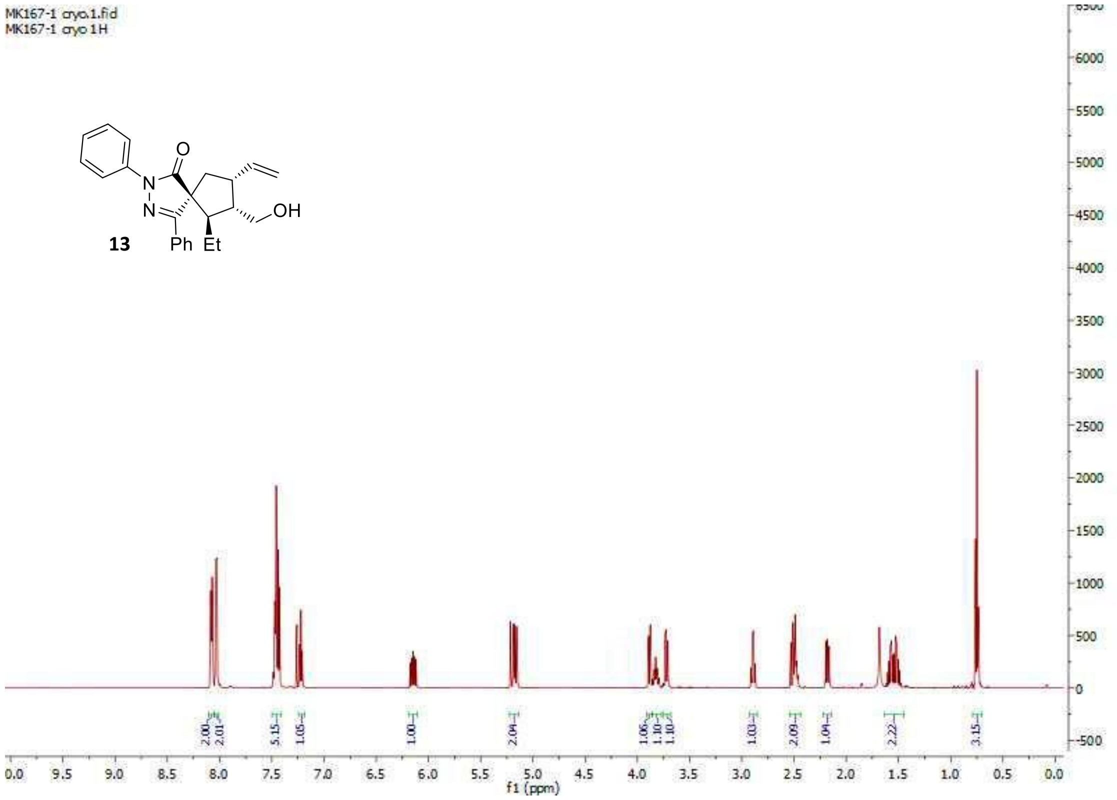
MK167-1 cryo.1.fid
MK167-1 cryo 1H
13
Ph
Et
OH
f1 (ppm)

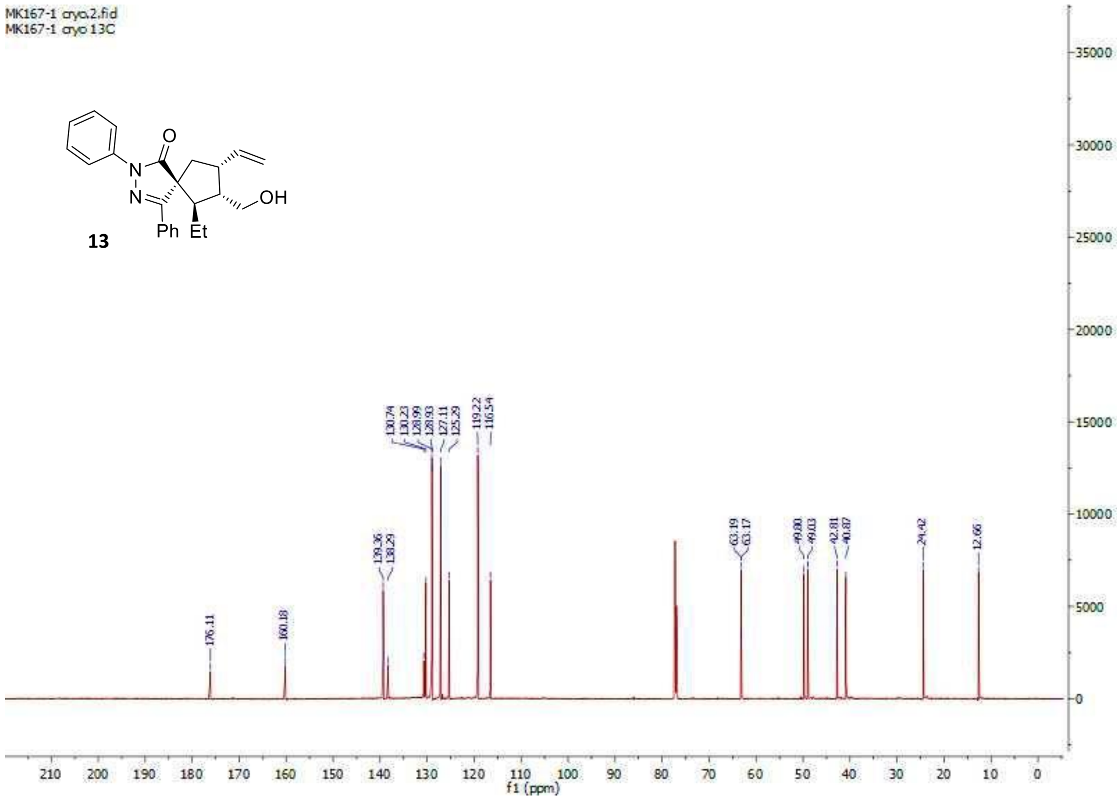
MK167-1 cryo.2.fid
MK167-1 cryo 13C
13
Ph
Et
OH
f1 (ppm)

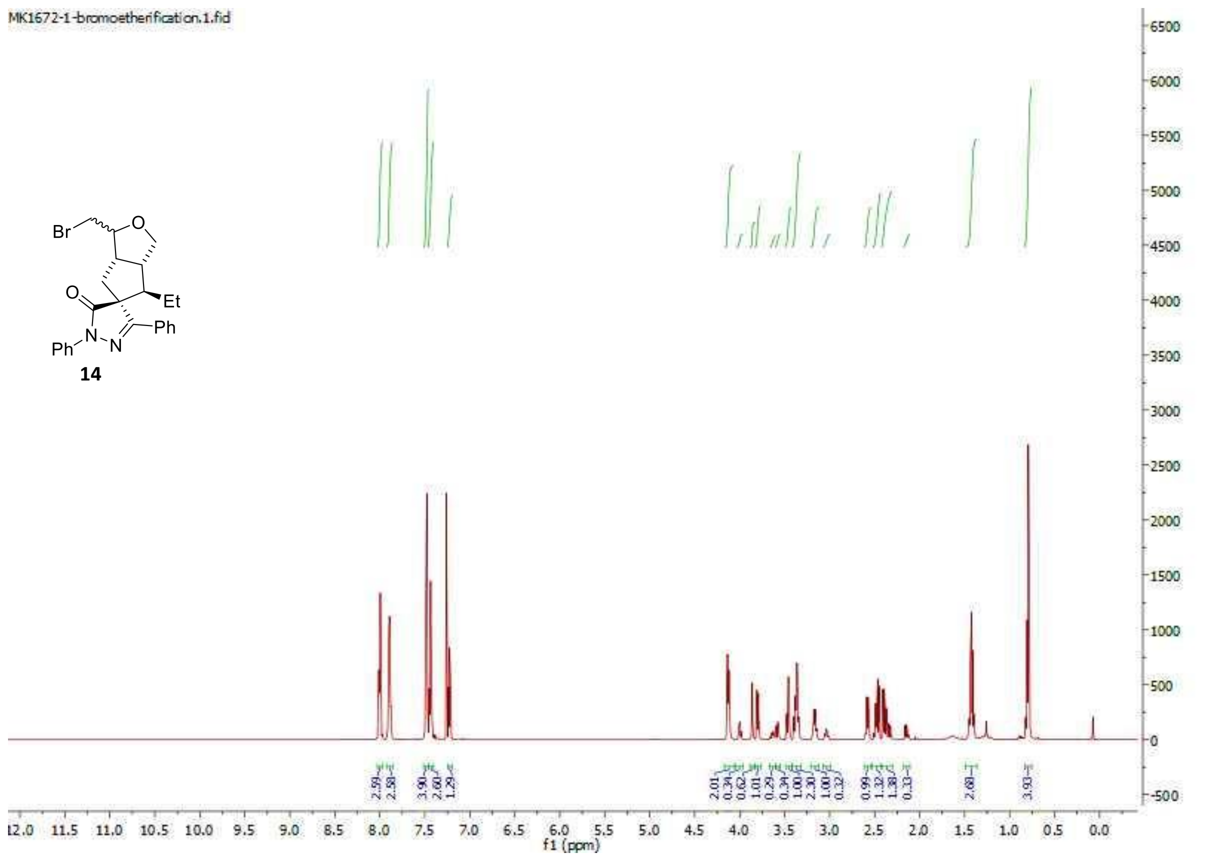

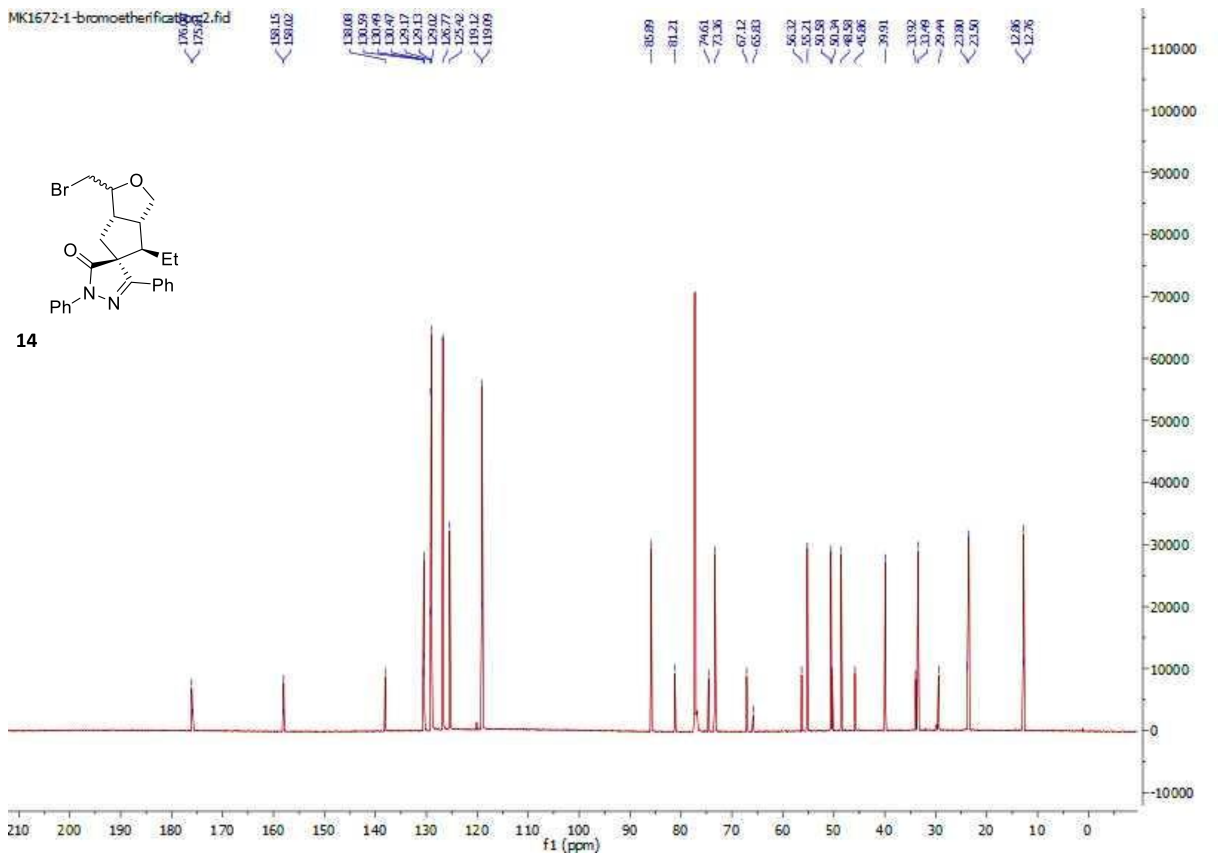

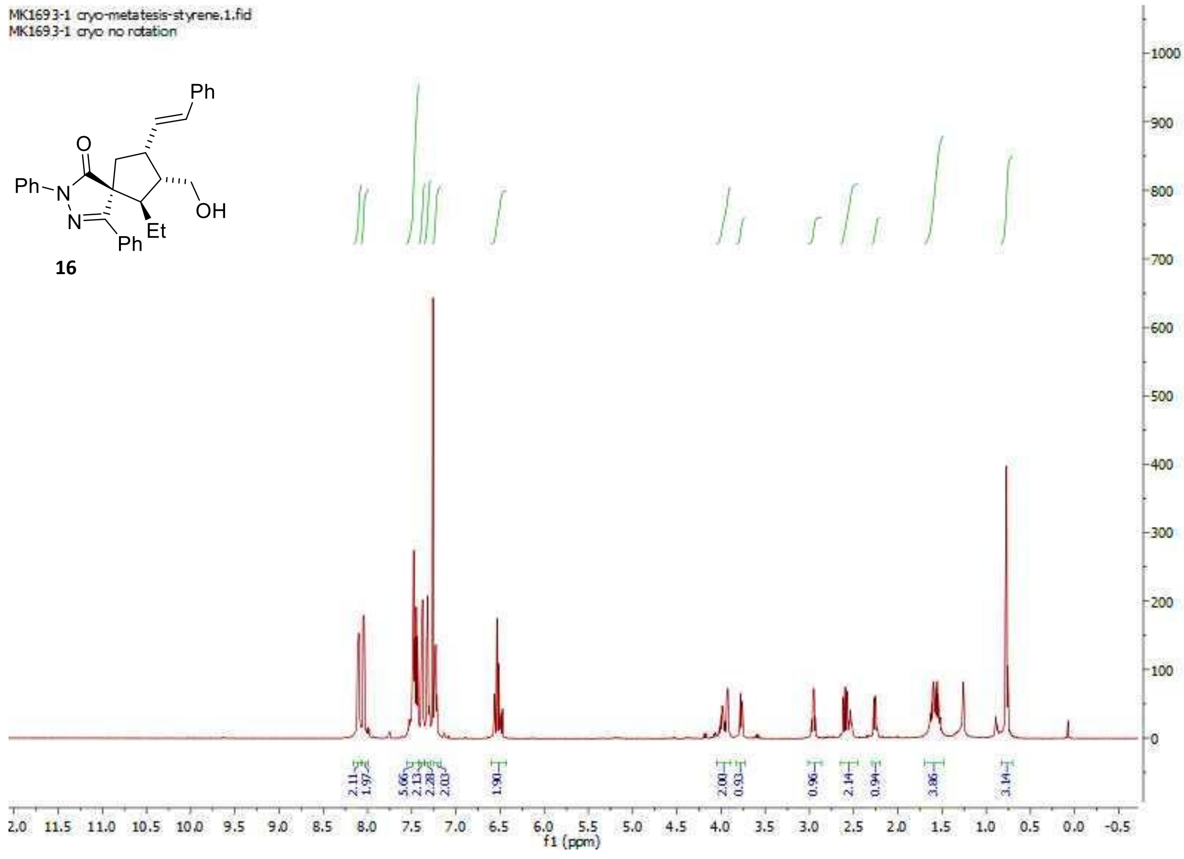
MK1693-1 cryo-metatesis-styrene.1.fid
MK1693-1 cryo no rotation
Ph
O
Ph
N
N
OH
Et
Ph
16
2.11
1.97
5.66
2.13
2.28
2.03
1.90
2.00
0.93
0.96
2.14
0.94
3.86
3.14
f1 (ppm)

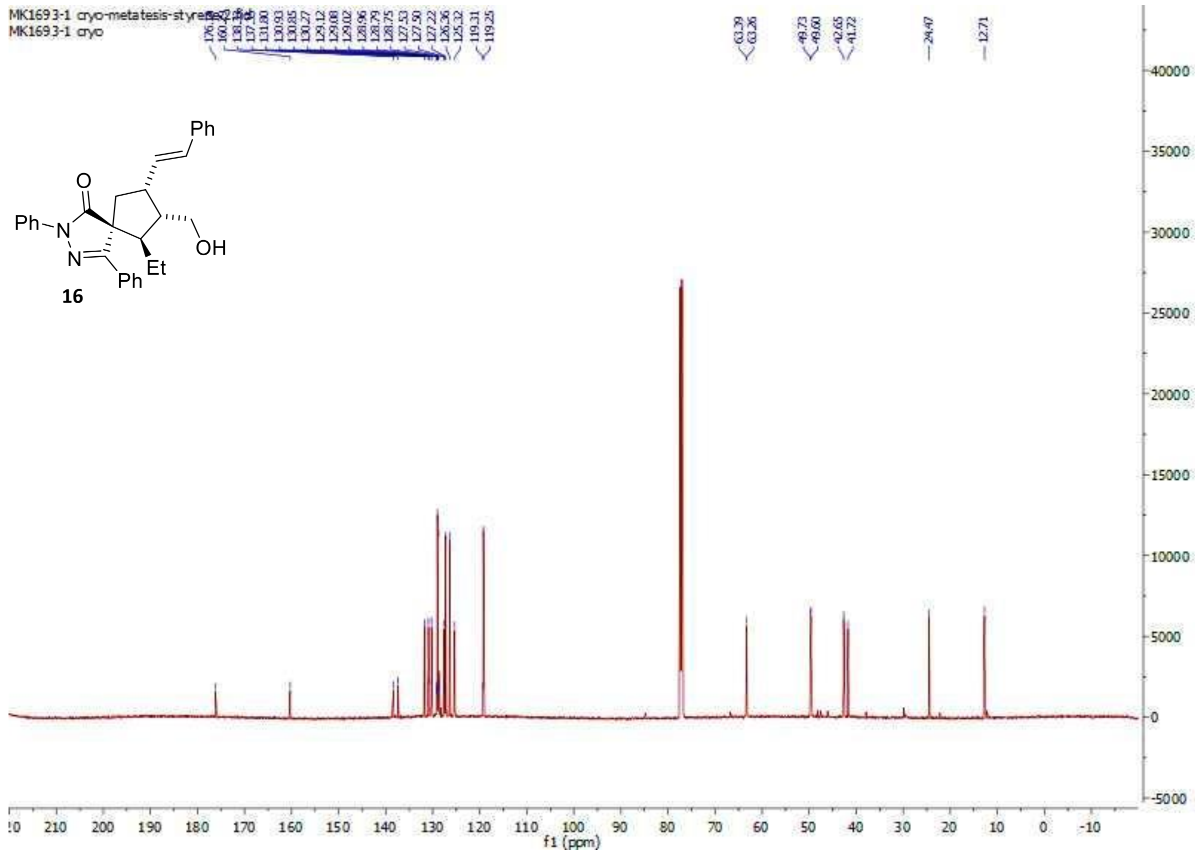
MK1693-1 cryo
131.80
130.93
130.85
130.27
129.12
129.08
129.02
128.96
128.79
128.75
127.53
127.50
127.22
126.36
125.32
119.31
119.25
63.39
63.26
49.73
49.60
42.65
41.72
24.47
12.71
Ph
O
Ph
N
N
OH
Et
Ph
16
f1 (ppm)

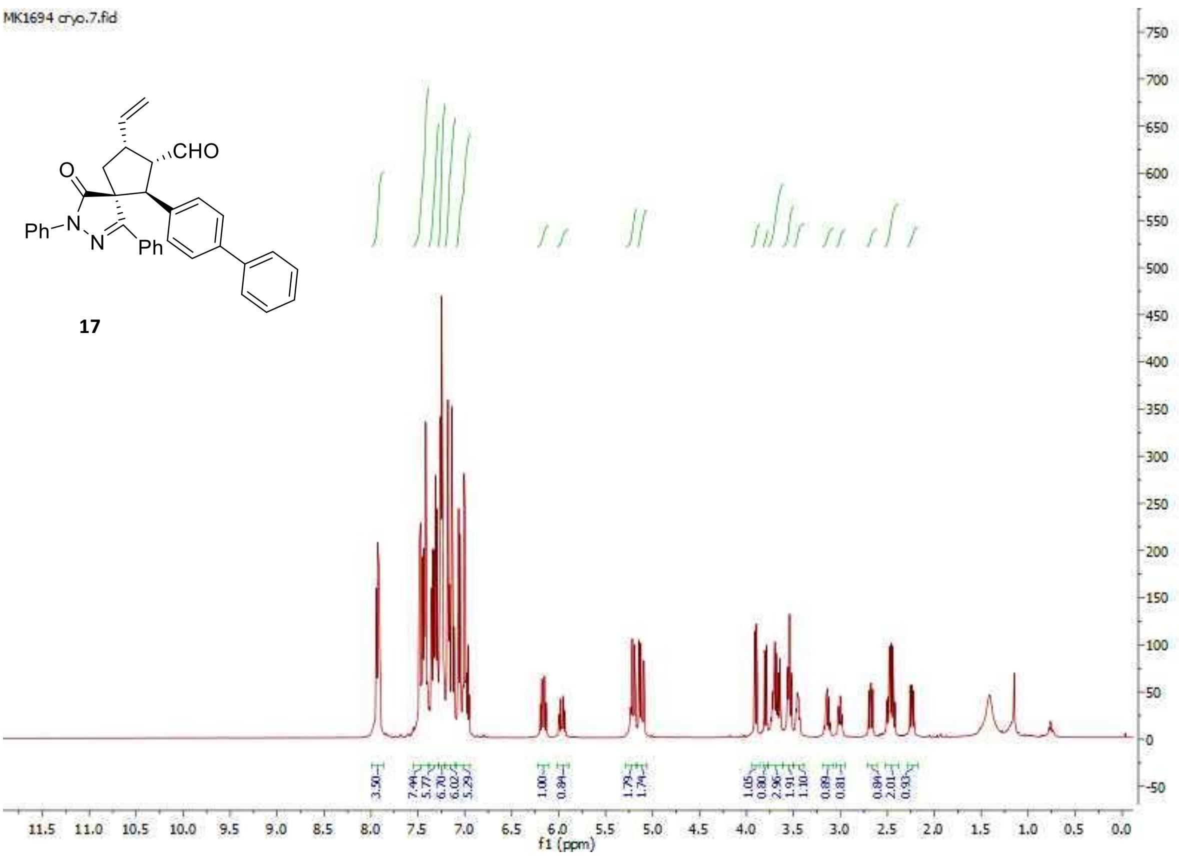
MK1694 cryo.7.fid
O
CHO
Ph
N
N
Ph
17
3.50
7.44
5.77
6.70
6.02
5.29
1.00
0.84
1.79
1.74
1.05
0.80
2.96
1.91
1.10
0.89
0.81
0.84
2.01
0.93
f1 (ppm)

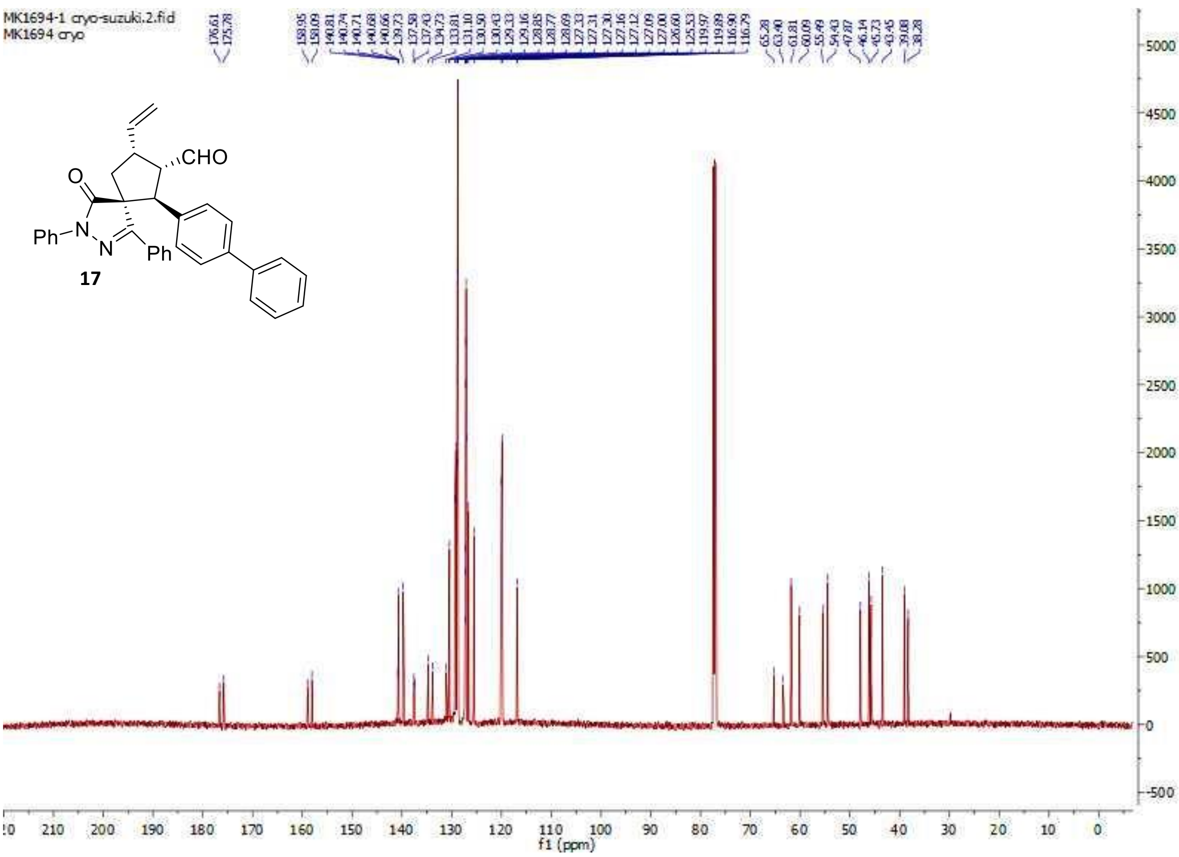
MK1694-1 cryo-suzuki.2.fid
MK1694 cryo
176.61
175.78
158.95
158.09
140.81
140.74
140.71
140.68
140.66
139.73
137.58
137.43
134.73
133.81
131.10
130.50
130.43
129.33
129.16
128.85
128.77
128.69
127.33
127.31
127.30
127.16
127.12
127.09
127.00
126.60
125.53
119.97
119.89
116.90
116.29
65.28
63.40
61.81
60.09
55.49
54.43
47.87
46.14
45.73
43.45
39.08
38.28
O
CHO
Ph
N
N
Ph
17
f1 (ppm)

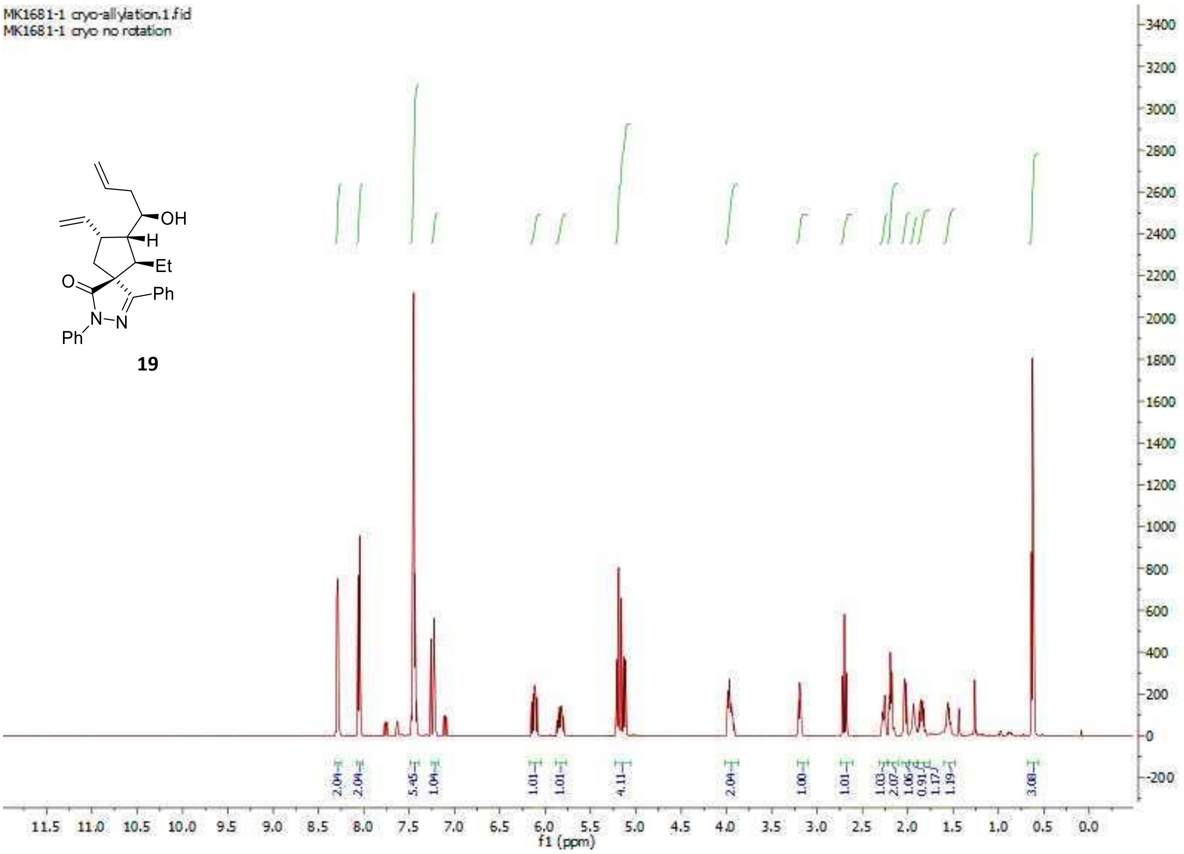
MK1681-1 cryo-allylation.1.fid
MK1681-1 cryo no rotation
OH
H
Et
Ph
O
N
N
Ph
19
2.04
2.04
5.45
1.04
1.01
1.01
4.11
2.04
1.00
1.01
1.03
2.07
1.06
0.91
1.17
1.19
3.08
f1 (ppm)

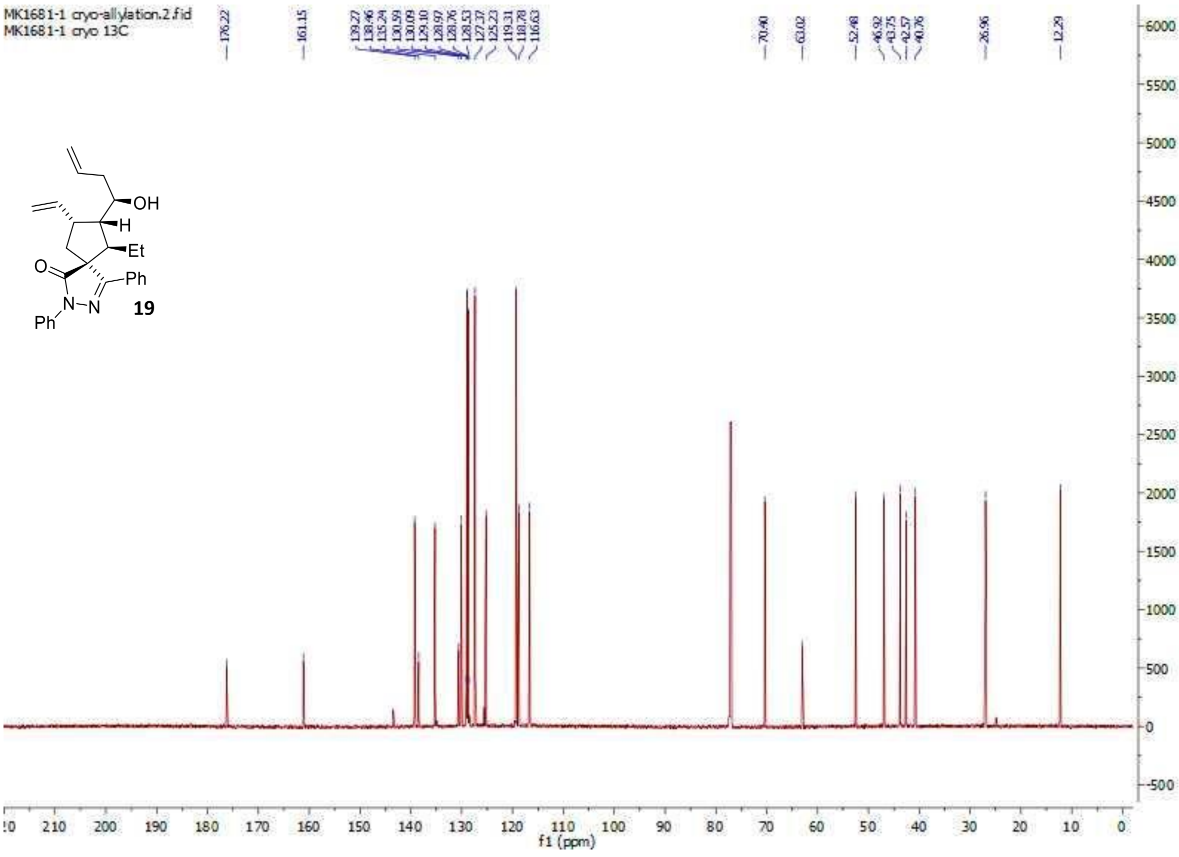
MK1681-1 cryo-allylation.2.fid
MK1681-1 cryo 13C
176.22
161.15
139.27
138.46
135.24
130.59
130.09
129.10
128.97
128.76
128.53
127.37
125.23
119.31
118.78
116.63
70.40
63.02
52.48
46.92
43.75
42.57
40.76
26.96
12.29
OH
H
Et
Ph
O
N
N
Ph
19
f1 (ppm)

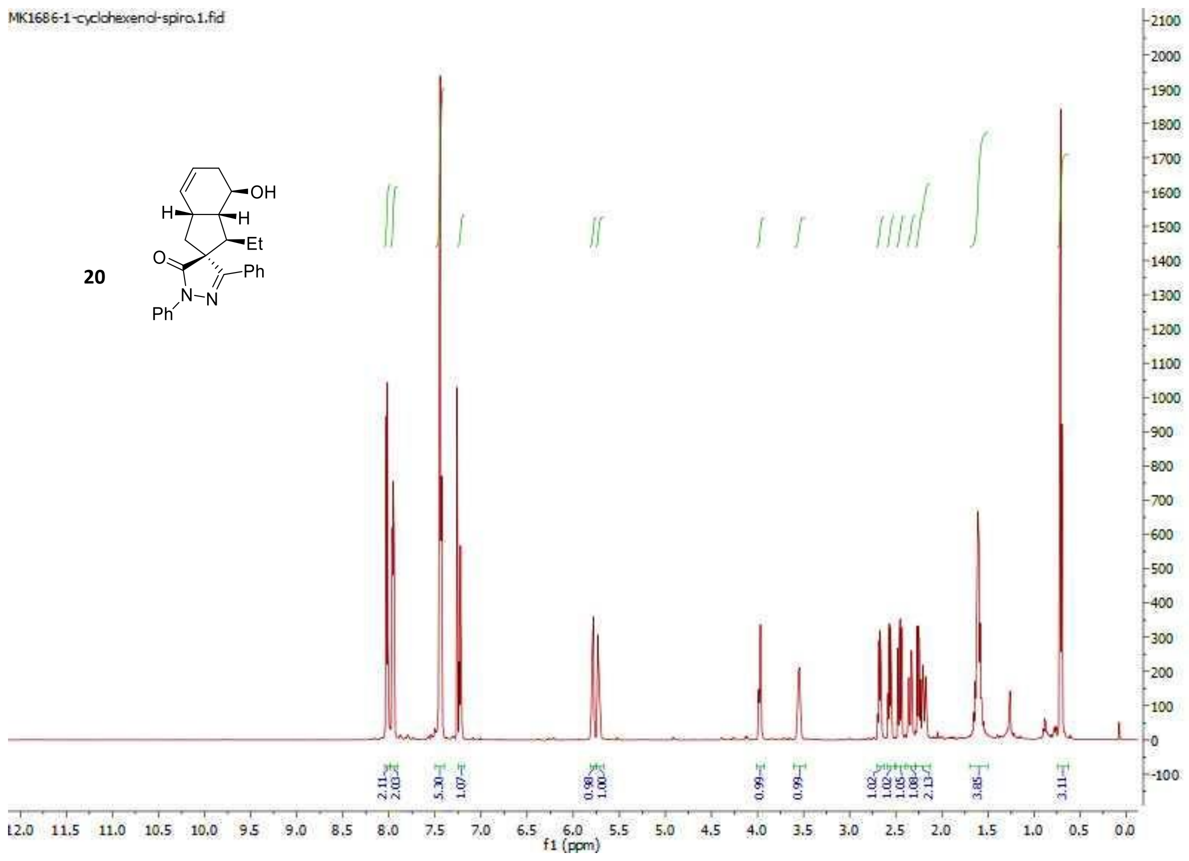
MK1686-1-cyclohexenol-spiro.1.fid
20
OH
H
Et
Ph
O
N
Ph
f1 (ppm)

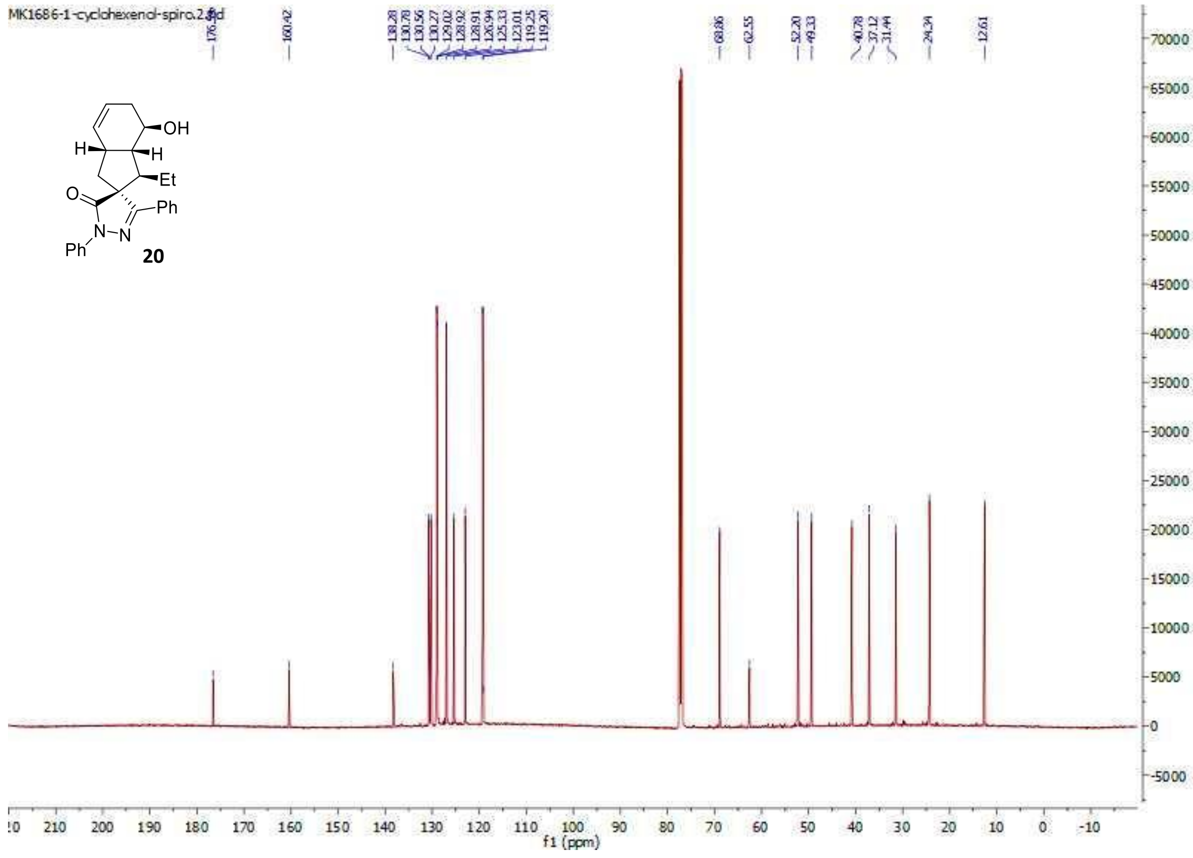
MK1686-1-cyclohexenol-spiro.2.fid
20
OH
H
Et
Ph
O
N
Ph
f1 (ppm)

9. HPLC traces

Racemic **3a**:

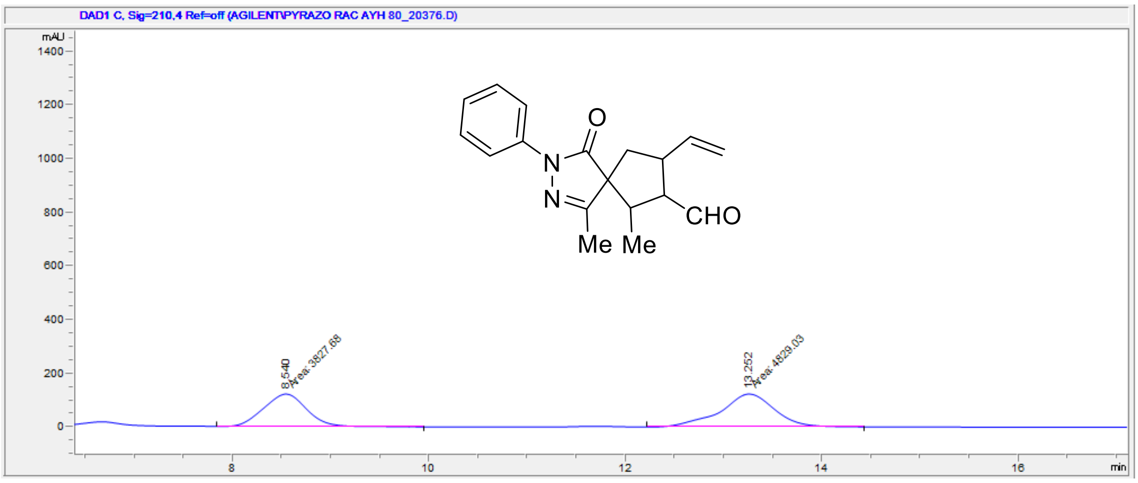

Peak #	RetTime [min]	Type	Width [min]	Area [mAU*s]	Height [mAU]	Area %
1	8.540	MM	0.5201	3827.67749	122.66637	44.2163
2	13.252	MM	0.6514	4829.02979	123.55486	55.7837

Chiral R-catalyst **3a**:

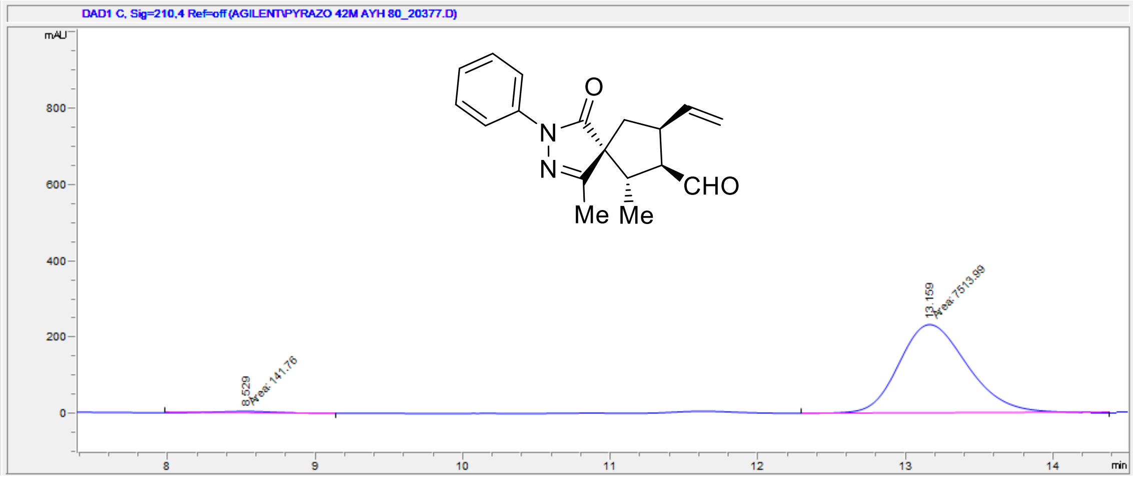

Peak #	RetTime [min]	Type	Width [min]	Area [mAU*s]	Height [mAU]	Area %
1	8.529	MM	0.5113	141.76050	4.62089	1.8517
2	13.159	MM	0.5415	7513.99268	231.25137	98.1483

Racemic **3b**:

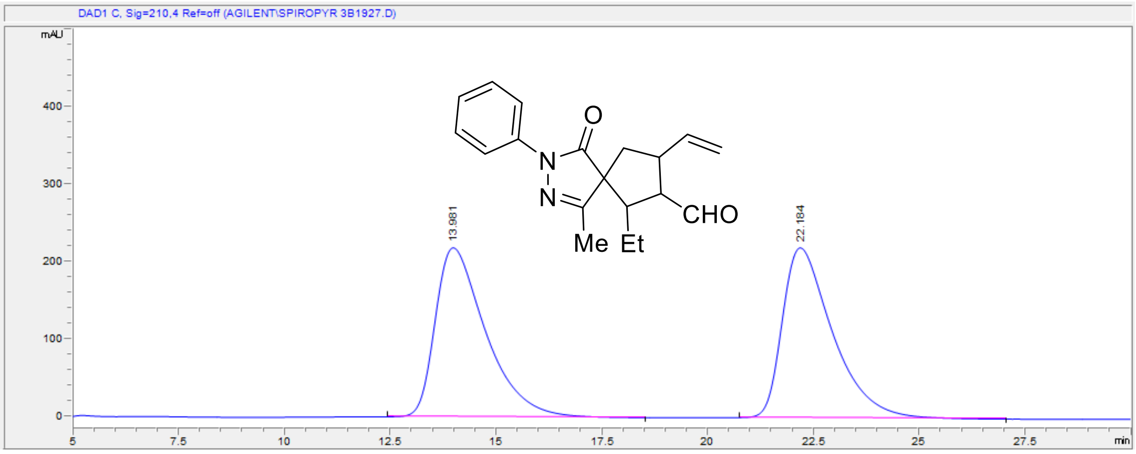

Peak #	RetTime [min]	Type	Width [min]	Area [mAU*s]	Height [mAU]	Area %
1	13.981	BB	1.2386	1.81793e4	218.51396	49.9983
2	22.184	BB	1.2338	1.81805e4	219.61974	50.0017

Chiral S-catalyst **3b**:

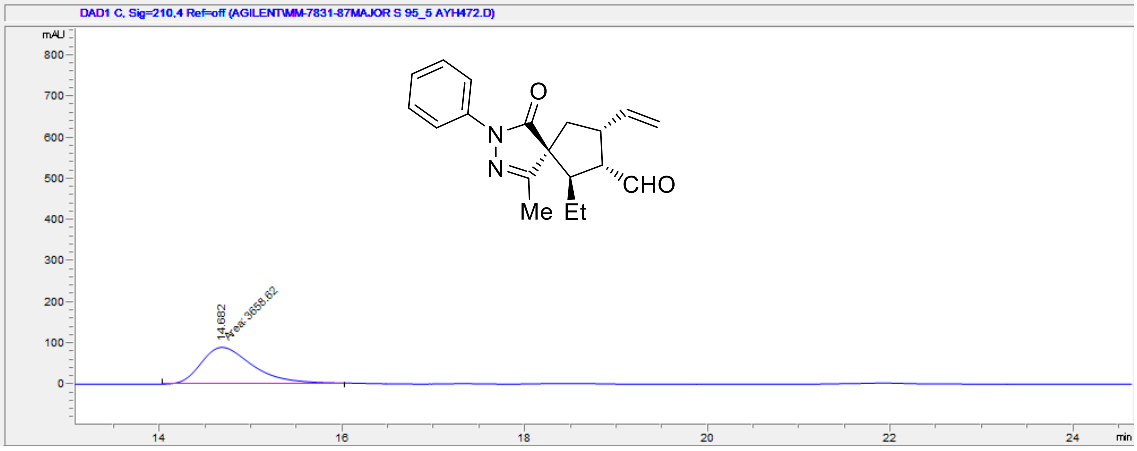

Peak #	RetTime [min]	Type	Width [min]	Area [mAU*s]	Height [mAU]	Area %
1	14.682	MM	0.6756	3658.61670	90.25788	100.0000

Racemic **3c**:

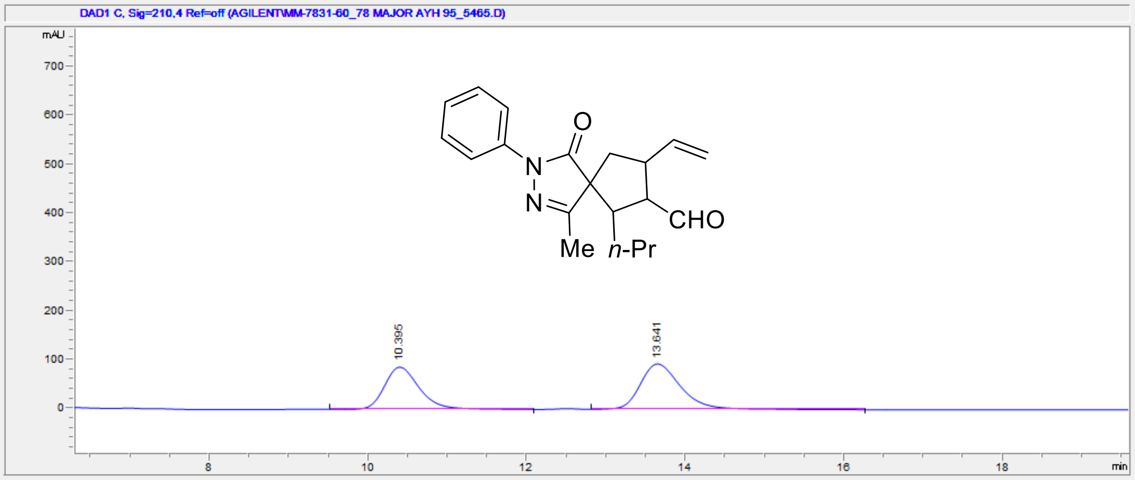

Peak #	RetTime [min]	Type	Width [min]	Area [mAU*s]	Height [mAU]	Area %
1	10.395	BB	0.4670	2652.92798	86.70617	44.3344
2	13.641	BB	0.5461	3330.96948	93.39201	55.6656

Chiral S-catalyst **3c**:

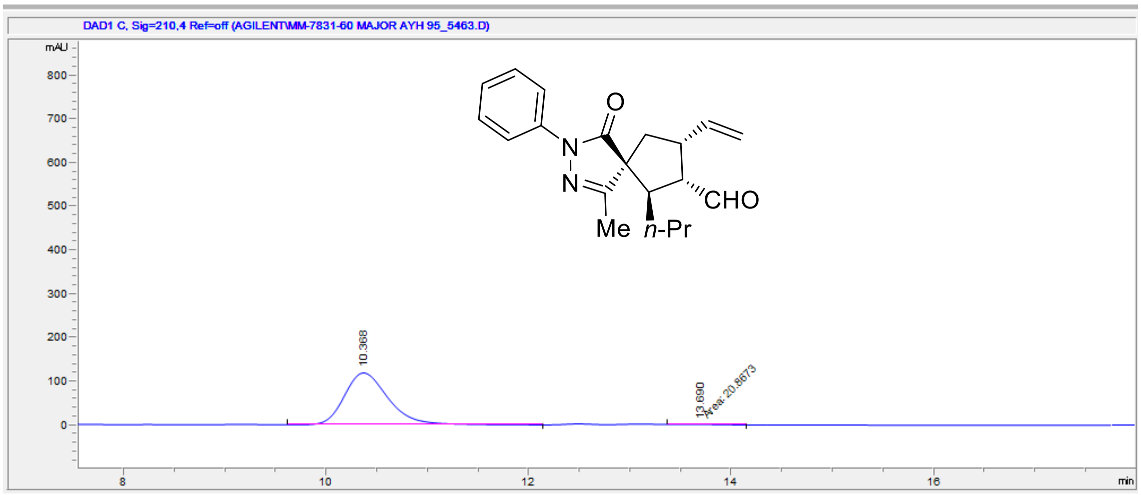

Peak #	RetTime [min]	Type	Width [min]	Area [mAU*s]	Height [mAU]	Area %
1	10.368	BB	0.4659	3604.73584	118.81724	99.4244
2	13.690	MM	0.4551	20.86733	7.64159e-1	0.5756

Racemic **3d**:

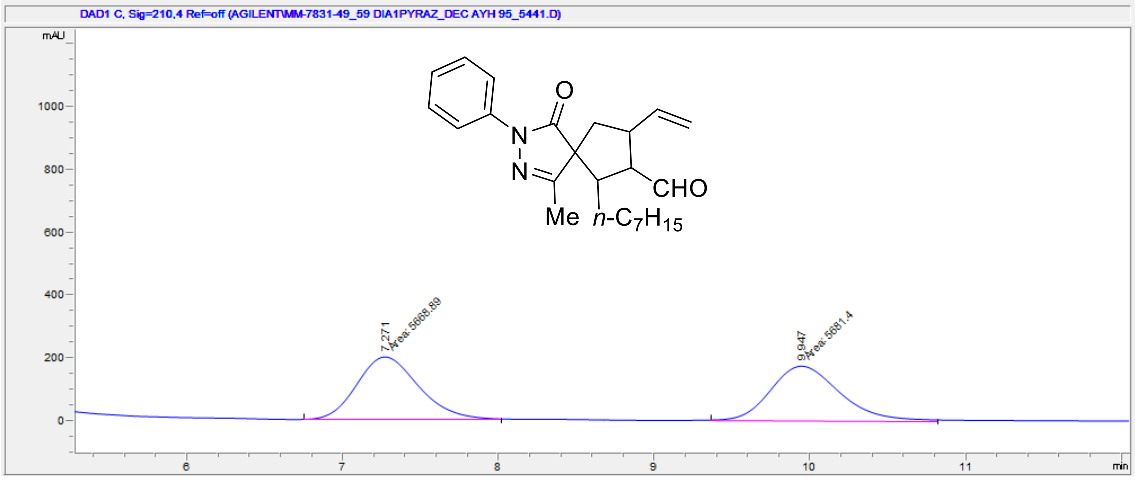

Peak #	RetTime [min]	Type	Width [min]	Area [mAU*s]	Height [mAU]	Area %
1	7.271	MM	0.4686	5668.89111	201.62810	49.9449
2	9.947	MM	0.5332	5681.39990	177.60349	50.0551

Chiral S-catalyst **3d**:

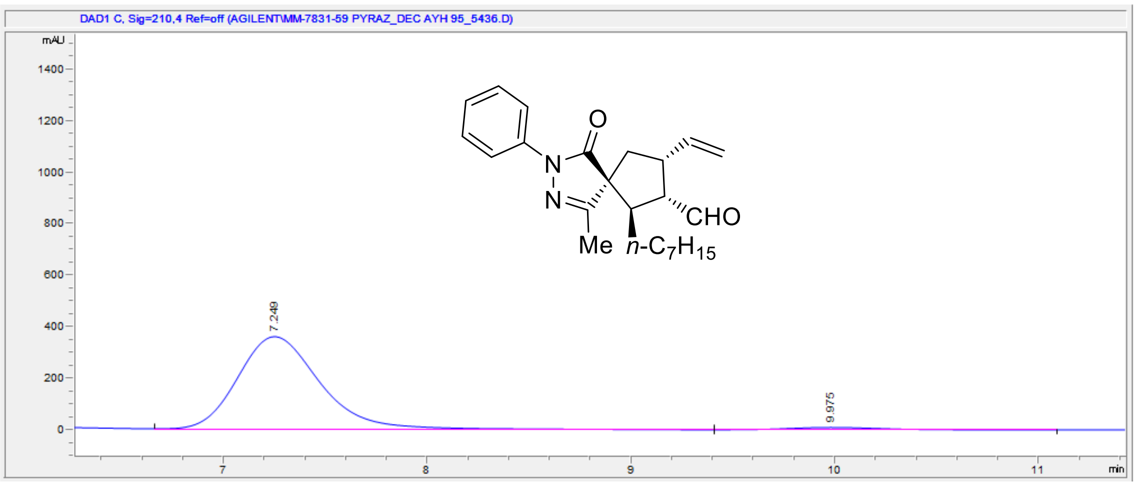

Peak #	RetTime [min]	Type	Width [min]	Area [mAU*s]	Height [mAU]	Area %
1	7.249	VB	0.4387	1.03637e4	361.21155	97.3841
2	9.975	BB	0.4572	278.38431	9.04076	2.6159

Racemic **3e**:

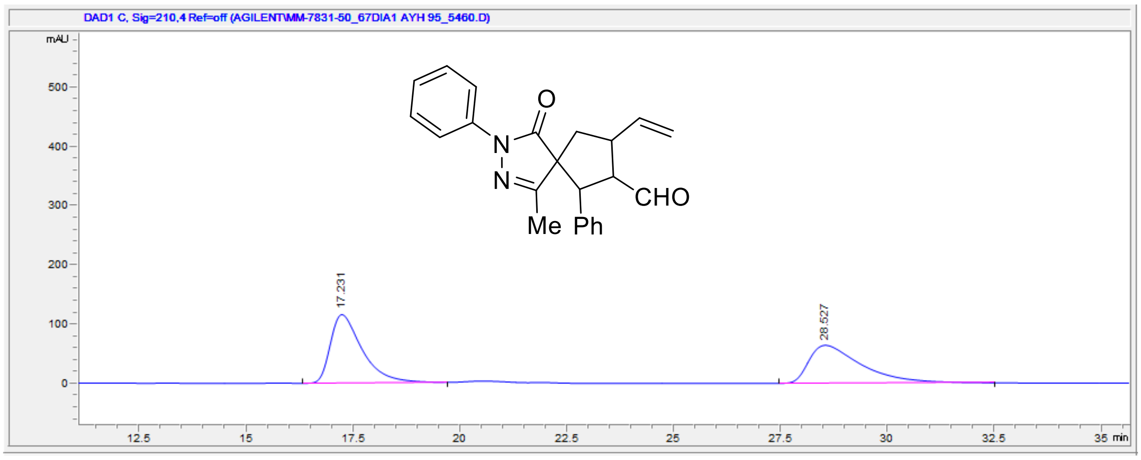

Peak #	RetTime [min]	Type	Width [min]	Area [mAU*s]	Height [mAU]	Area %
1	17.231	BB	0.7610	5973.08252	115.56862	52.0811
2	28.527	BB	1.2203	5495.73291	64.09579	47.9189

Chiral S-catalyst **3e**:

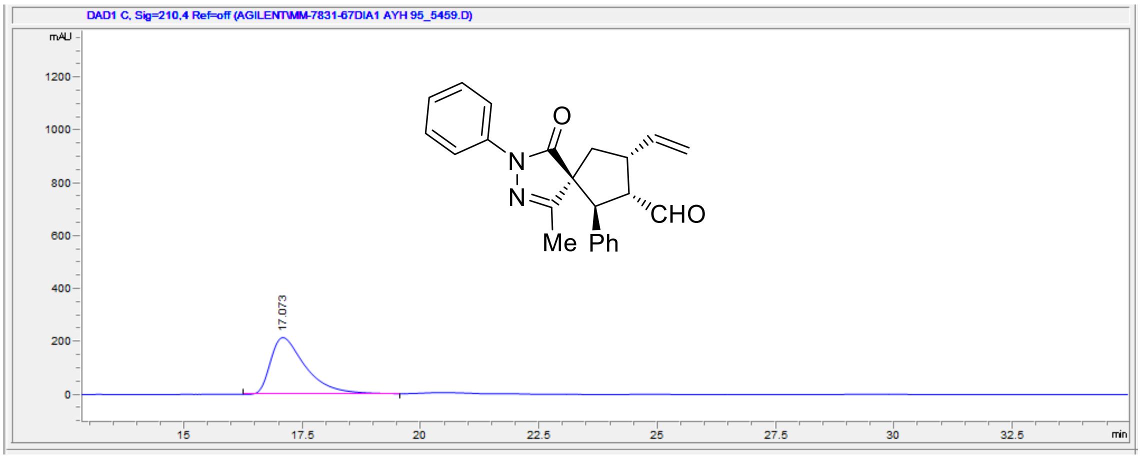

Peak #	RetTime [min]	Type	Width [min]	Area [mAU*s]	Height [mAU]	Area %
1	17.073	BB	0.7671	1.10469e4	214.39632	100.0000

Racemic **3f**:

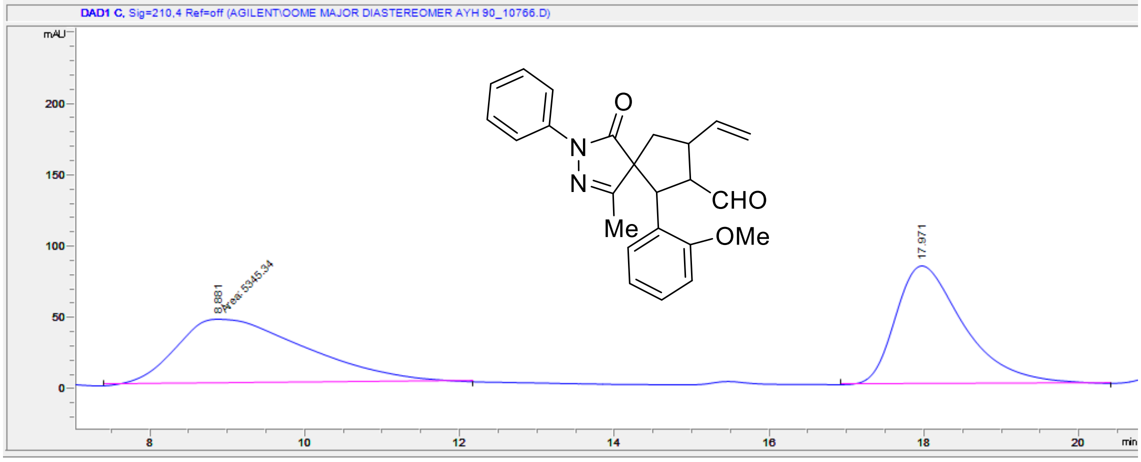

Peak #	RetTime [min]	Type	Width [min]	Area [mAU*s]	Height [mAU]	Area %
1	8.881	MM	1.9601	5345.34131	45.45207	51.0661
2	17.971	BB	0.9237	5122.14844	83.26894	48.9339

Chiral S-catalyst **3f**:

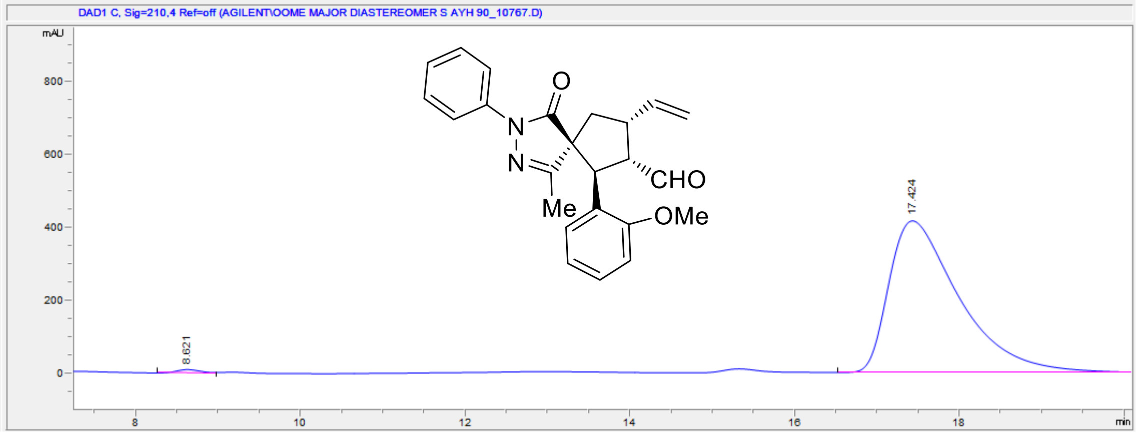

Peak #	RetTime [min]	Type	Width [min]	Area [mAU*s]	Height [mAU]	Area %
1	8.621	BV	0.3185	202.34747	9.73465	0.8183
2	17.424	BB	0.8849	2.45246e4	415.33023	99.1817

Racemic **3g**:

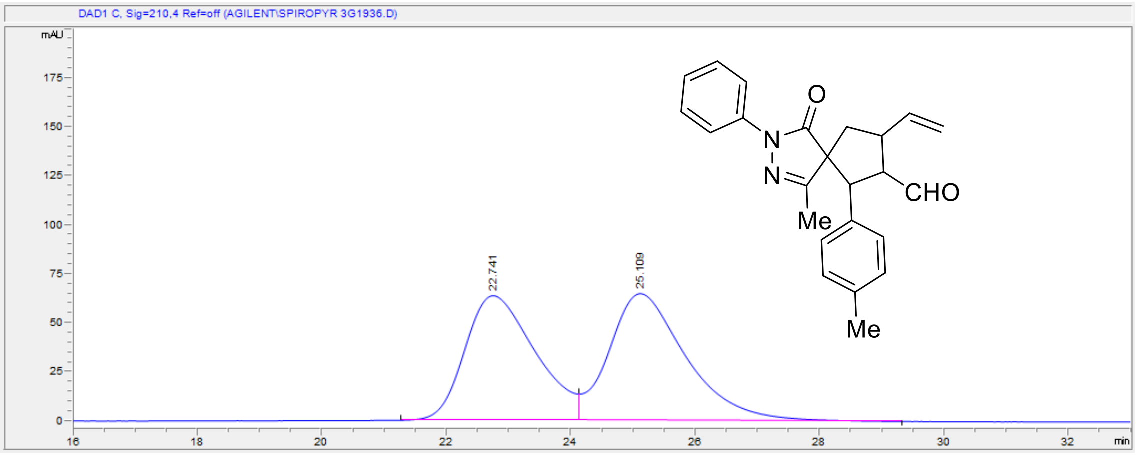

Peak #	RetTime [min]	Type	Width [min]	Area [mAU*s]	Height [mAU]	Area %
1	22.741	BV	1.1525	4999.61084	63.50286	47.1329
2	25.109	VB	1.2610	5607.86182	64.71004	52.8671

Chiral S-catalyst **3g**:

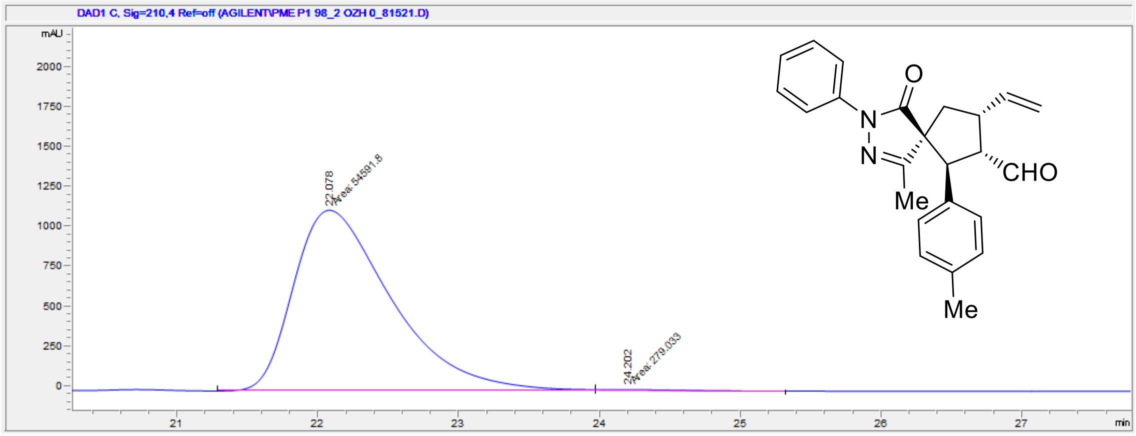

Peak #	RetTime [min]	Type	Width [min]	Area [mAU*s]	Height [mAU]	Area %
1	22.078	MM	0.8063	5.45918e4	1128.46704	99.4915
2	24.202	MM	0.6734	279.03348	6.90646	0.5085

Racemic **3h**:

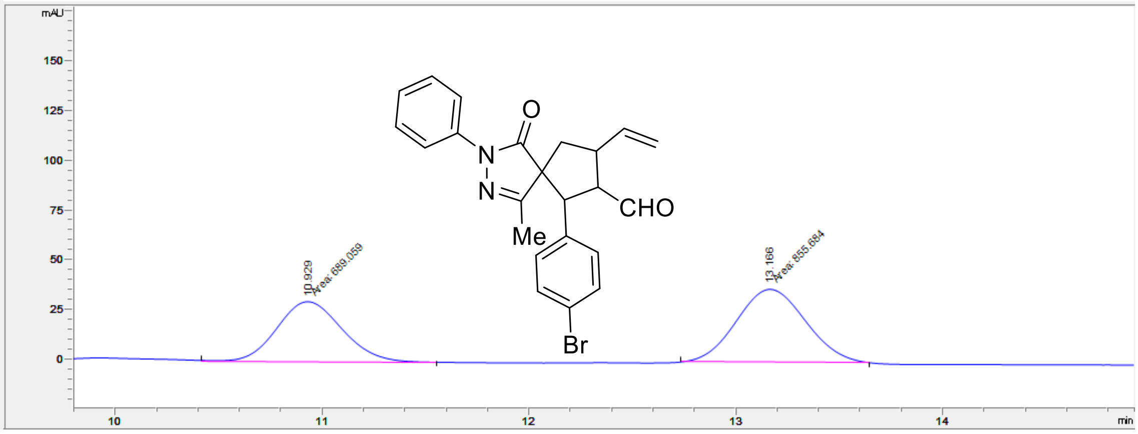

Peak #	RetTime [min]	Type	Width [min]	Area [mAU*s]	Height [mAU]	Area %
1	10.929	MM	0.3759	689.05890	30.54777	44.6067
2	13.166	MM	0.3885	855.68365	36.70696	55.3933

Chiral S-catalyst **3h**:

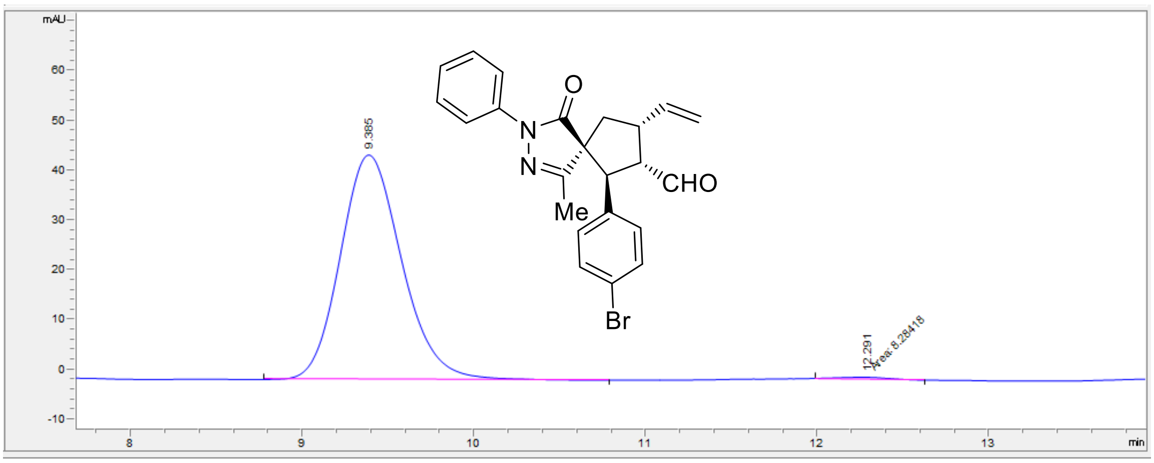

Peak #	RetTime [min]	Type	Width [min]	Area [mAU*s]	Height [mAU]	Area %
1	9.385	BB	0.3851	1126.19763	45.14216	99.2698
2	12.291	MM	0.3351	8.28418	4.12062e-1	0.7302

Racemic **3i major**:

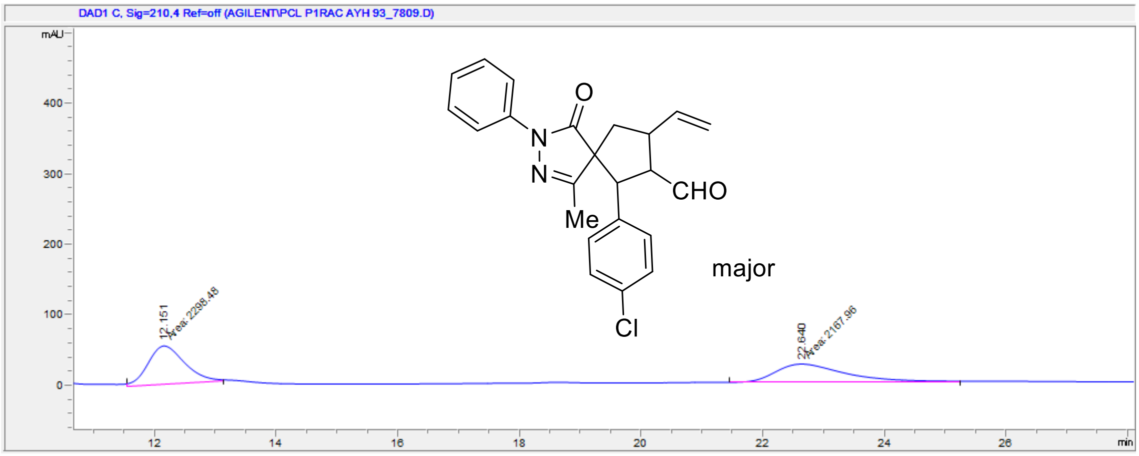

Peak #	RetTime [min]	Type	Width [min]	Area [mAU*s]	Height [mAU]	Area %
1	12.151	MM	0.7036	2298.48462	54.44628	51.4612
2	22.640	MM	1.3789	2167.95752	26.20407	48.5388

Chiral S-catalyst **3i major**:

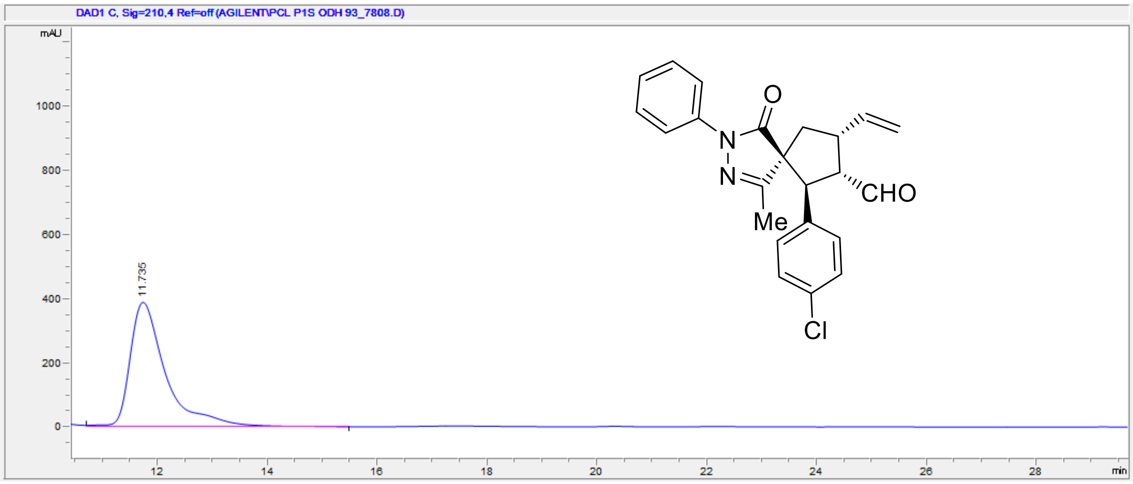

Peak #	RetTime [min]	Type	Width [min]	Area [mAU*s]	Height [mAU]	Area %
1	11.735	VB	0.6688	1.73813e4	386.37878	100.0000

Racemic **3j**:

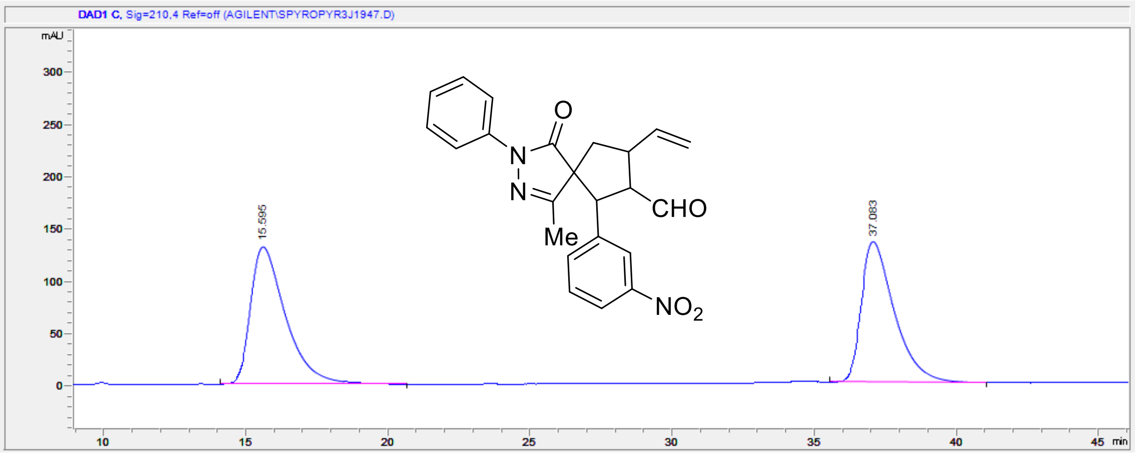

Peak #	RetTime [min]	Type	Width [min]	Area [mAU*s]	Height [mAU]	Area %
1	15.595	BB	1.2631	1.13974e4	131.76584	50.6251
2	37.083	BB	1.2197	1.11160e4	134.84590	49.3749

Chiral S-catalyst **3j**:

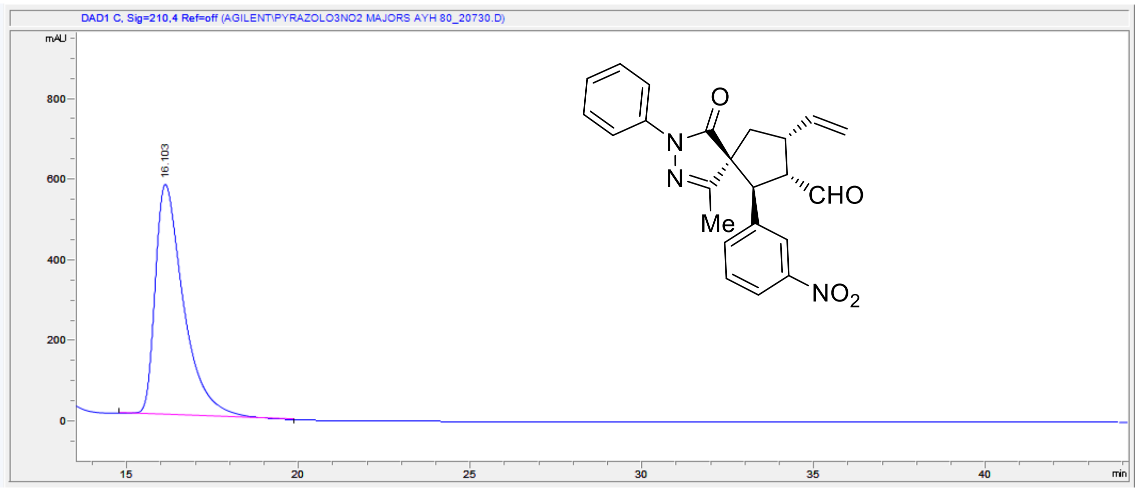

Peak #	RetTime [min]	Type	Width [min]	Area [mAU*s]	Height [mAU]	Area %
1	16.103	BB	0.8641	3.29665e4	572.22900	100.0000

Enantiomeric mixture **3k**:

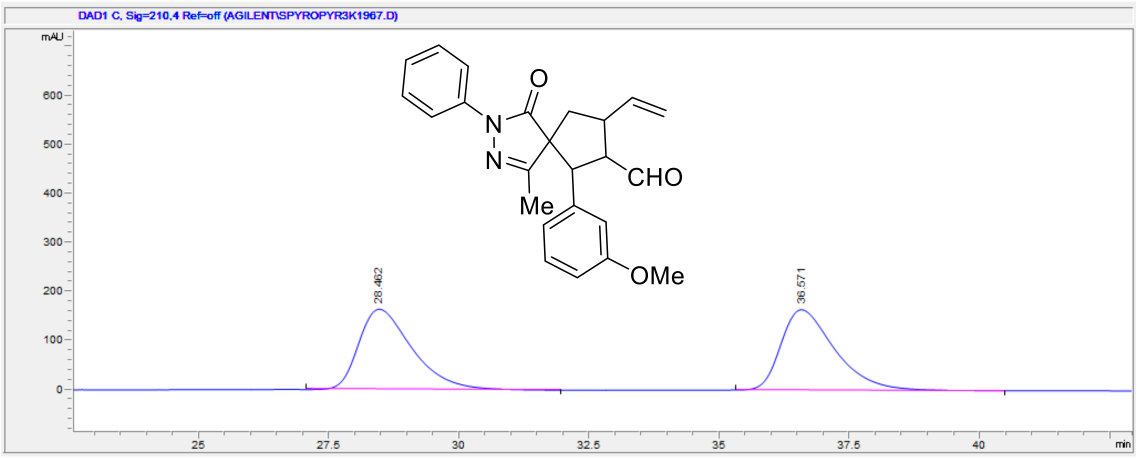

Peak #	RetTime [min]	Type	Width [min]	Area [mAU*s]	Height [mAU]	Area %
1	28.462	BB	1.0858	1.18367e4	164.83888	49.9541
2	36.571	BB	1.0719	1.18584e4	164.71542	50.0459

Chiral S-catalyst **3k**:

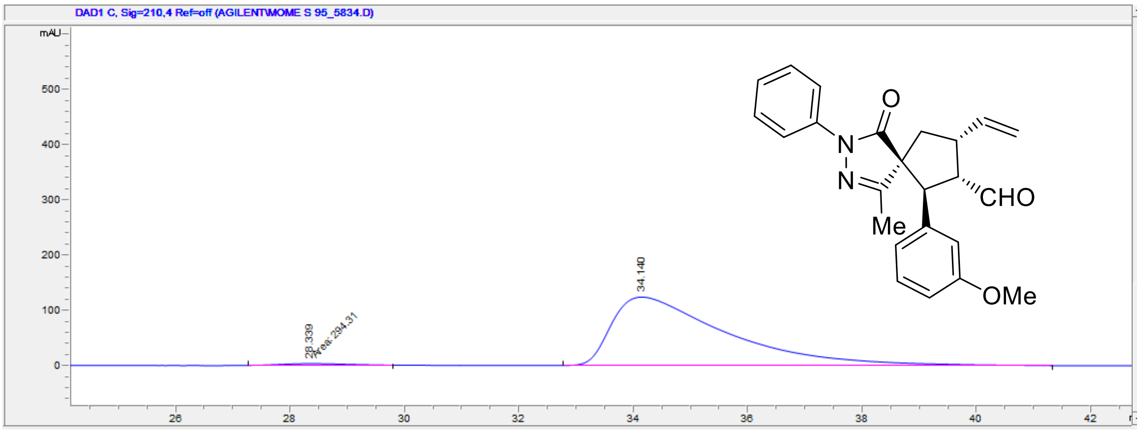

Peak #	RetTime [min]	Type	Width [min]	Area [mAU*s]	Height [mAU]	Area %
1	28.339	MM	1.4481	294.31003	3.38724	1.6816
2	34.140	BB	1.8371	1.72073e4	123.83883	98.3184

Racemic **3m**:

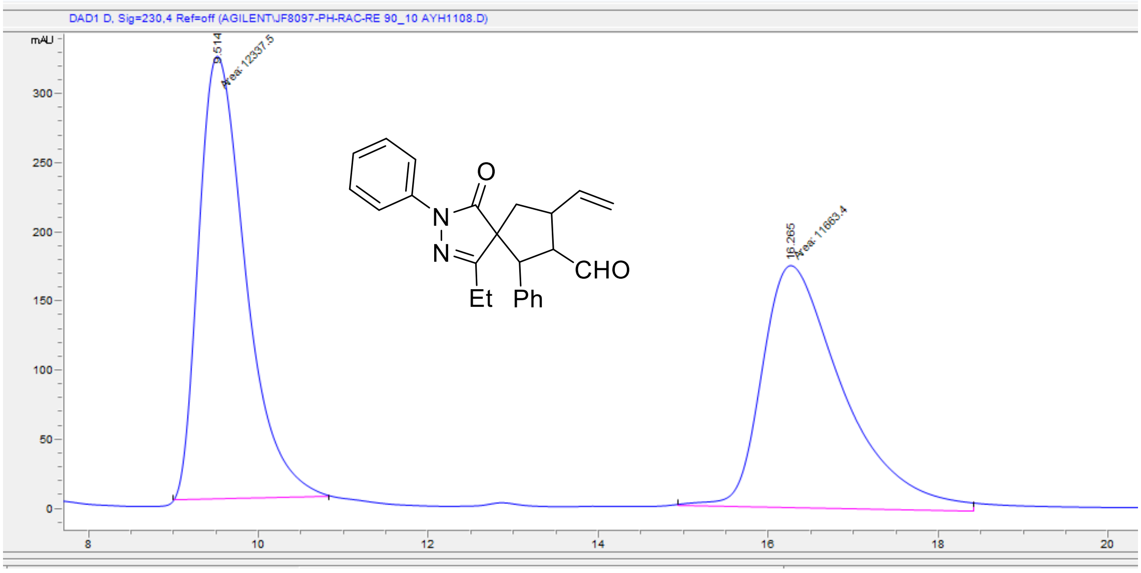

Peak #	RetTime [min]	Type	Width [min]	Area [mAU*s]	Height [mAU]	Area %
1	9.514	PM	0.6417	1.23375e4	320.44287	51.4042
2	16.265	MM	1.1116	1.16634e4	174.87427	48.5958

Chiral S-catalyst **3m**:

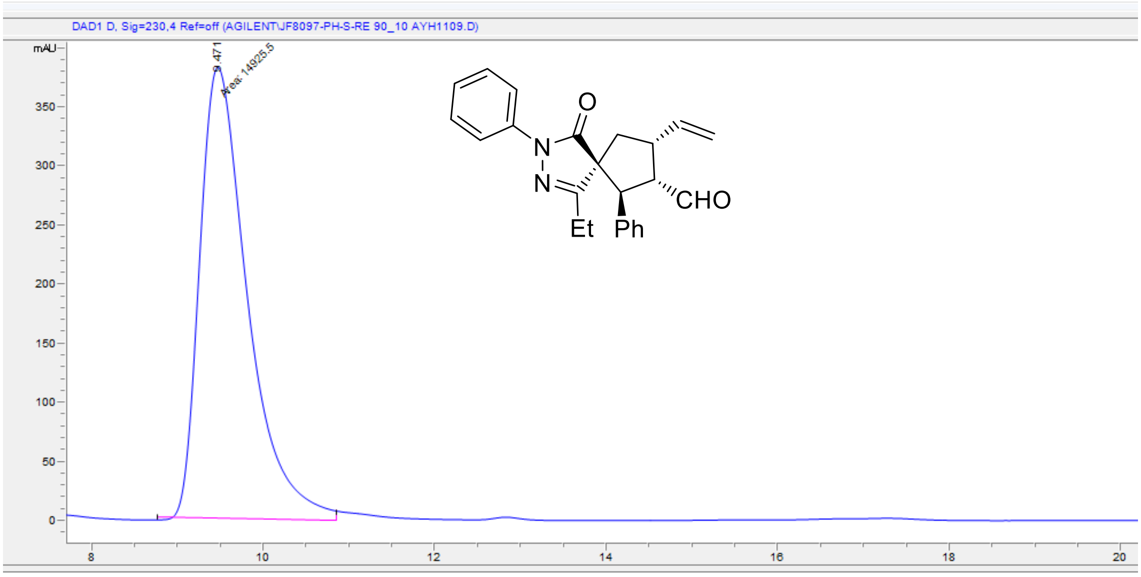

Peak #	RetTime [min]	Type	Width [min]	Area [mAU*s]	Height [mAU]	Area %
1	9.471	MM	0.6475	1.49255e4	384.20035	100.0000

Racemic **3n**:

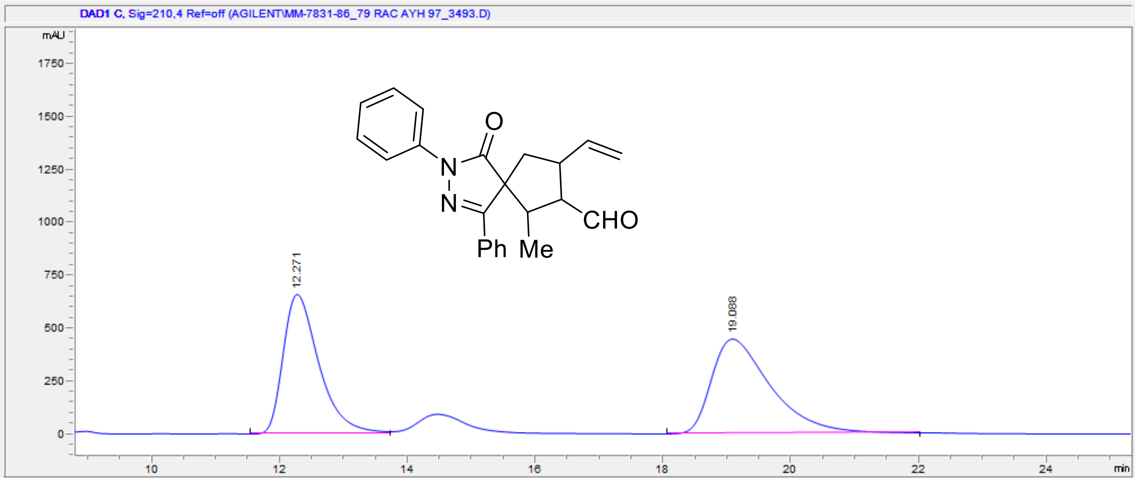

Peak #	RetTime [min]	Type	Width [min]	Area [mAU*s]	Height [mAU]	Area %
1	12.271	VV	0.5940	2.59211e4	660.24951	47.3973
2	19.088	BB	0.9708	2.87679e4	447.04059	52.6027

Chiral S-catalyst **3n**:

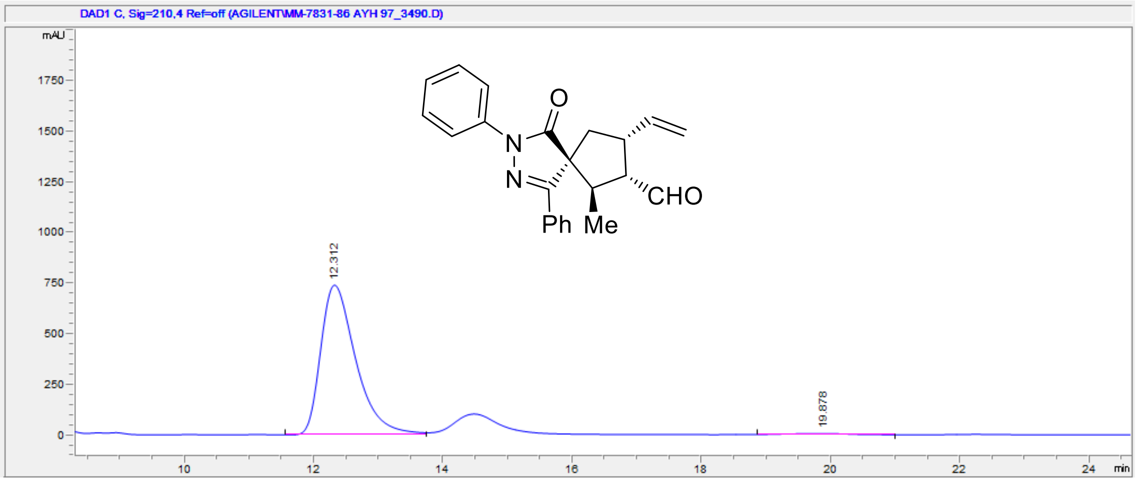

Peak #	RetTime [min]	Type	Width [min]	Area [mAU*s]	Height [mAU]	Area %
1	12.312	BV	0.5764	2.79338e4	740.02509	98.9898
2	19.878	BB	0.7103	285.07062	4.78994	1.0102

Racemic **3o**:

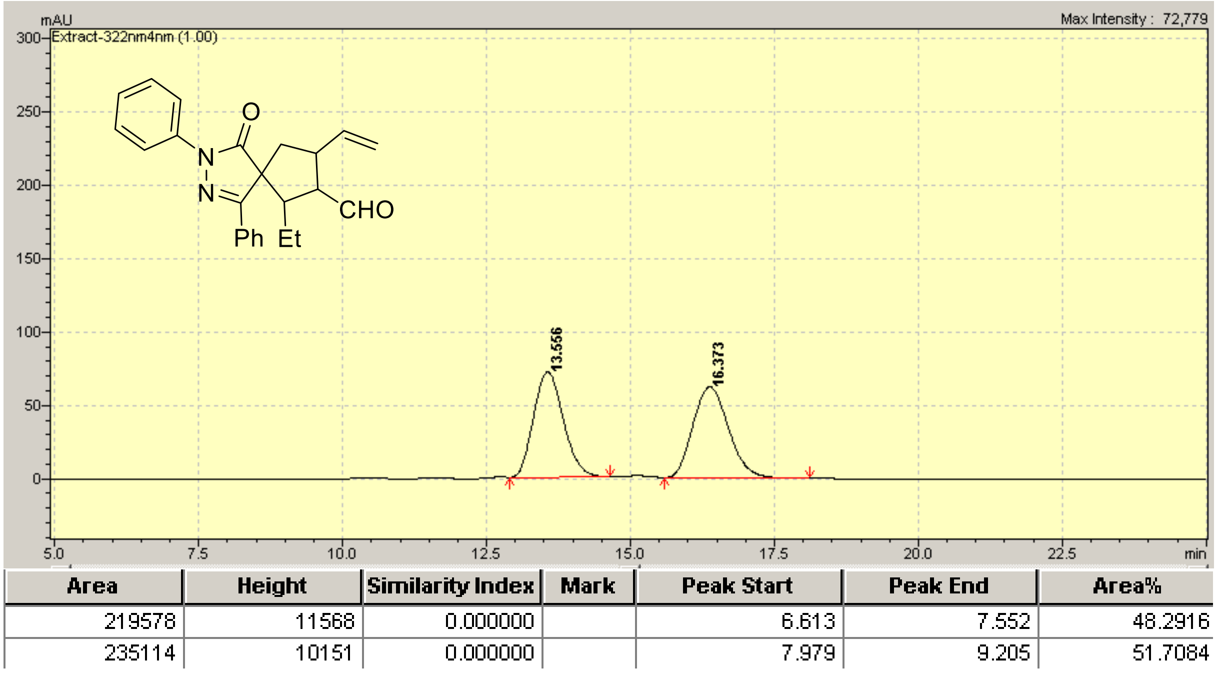

Area	Height	Similarity Index	Mark	Peak Start	Peak End	Area%
219578	11568	0.000000		6.613	7.552	48.2916
235114	10151	0.000000		7.979	9.205	51.7084

Chiral S-catalyst **3o**:

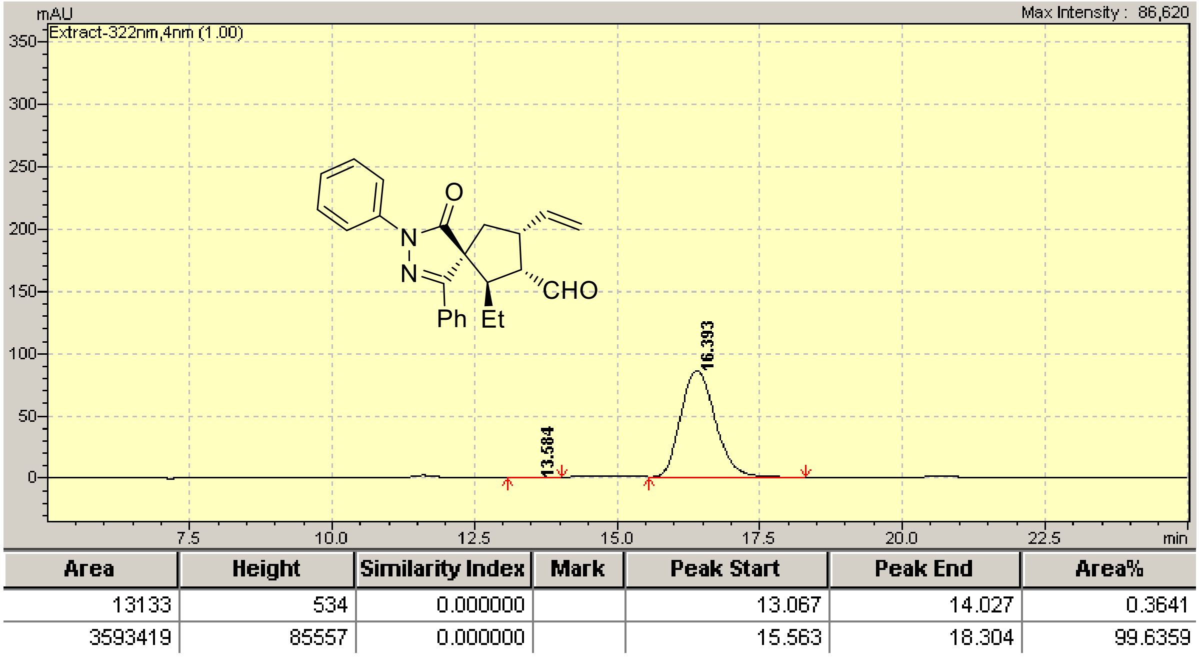

Area	Height	Similarity Index	Mark	Peak Start	Peak End	Area%
13133	534	0.000000		13.067	14.027	0.3641
3593419	85557	0.000000		15.563	18.304	99.6359

Racemic **3p**:

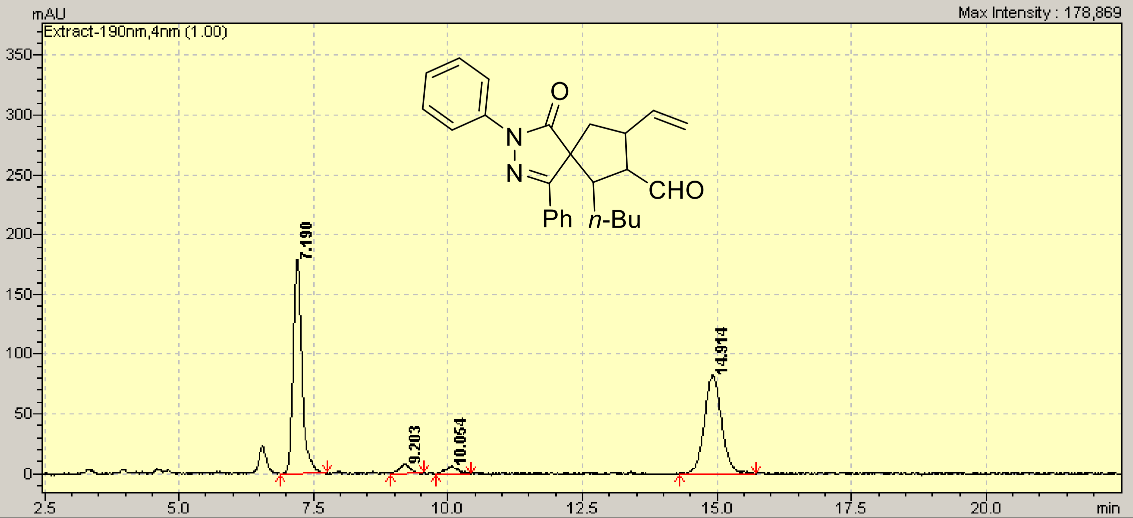

Area	Height	Similarity Index	Mark	Peak Start	Peak End	Area%
2002258	178960	0.000000		6.880	7.744	49.5184
100074	7975	0.000000		8.928	9.547	2.4750
114440	6998	0.000000		9.760	10.421	2.8302
1826690	82667	0.000000		14.315	15.712	45.1764

Chiral S-catalyst **3p**:

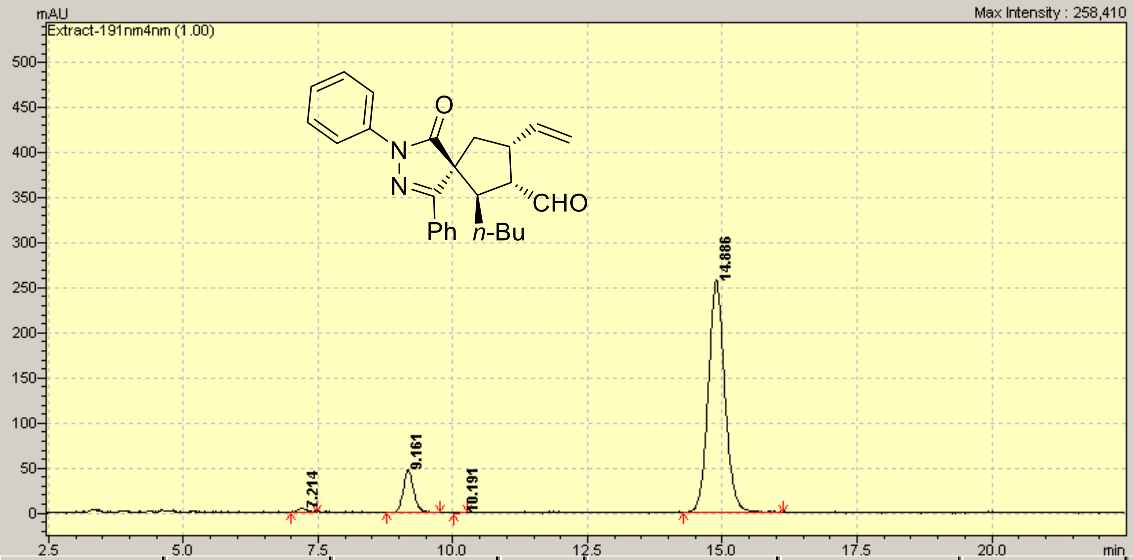

Area	Height	Similarity Index	Mark	Peak Start	Peak End	Area%
48056	4674	0.000000		6.987	7.488	0.7845
632741	46730	0.000000		8.779	9.771	10.3288
11176	1145	0.000000		10.016	10.272	0.1824
5434002	256936	0.000000		14.272	16.117	88.7043

Racemic **3q**:

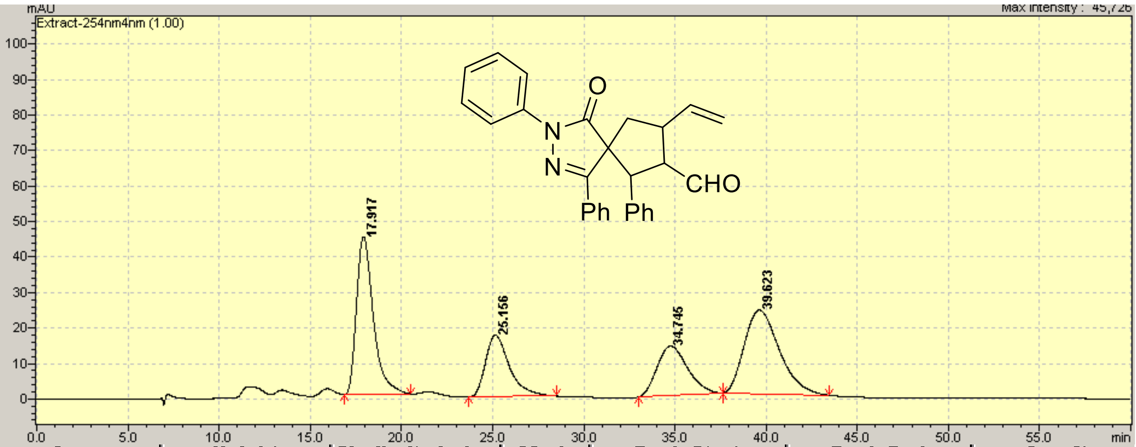

Area	Height	Similarity Index	Mark	Peak Start	Peak End	Area%
2859088	44426	0.000000		16.853	20.491	31.7259
1590662	17343	0.000000		23.680	28.512	17.6508
1610383	13914	0.000000		33.013	37.643	17.8696
2951712	23028	0.000000		37.771	42.539	32.7537

Chiral S-catalyst **3q**:

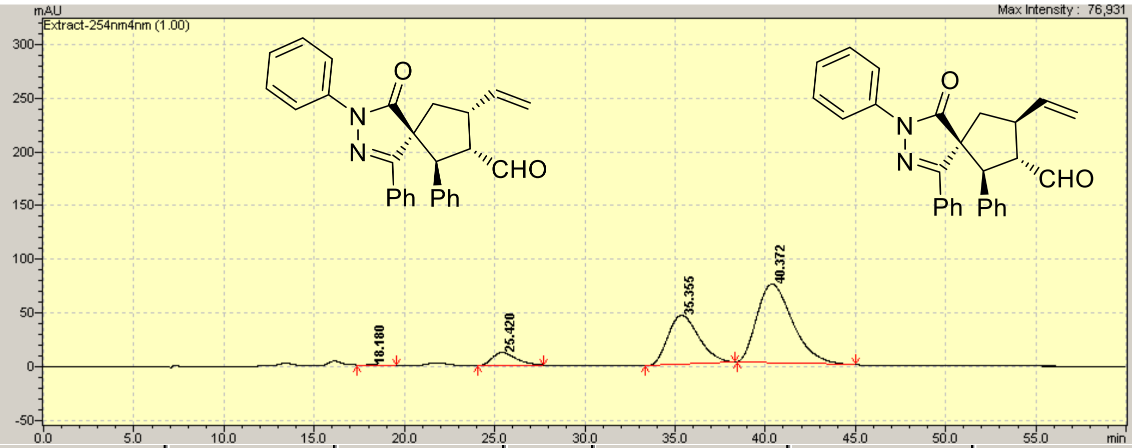

Area	Height	Similarity Index	Mark	Peak Start	Peak End	Area%
25818	571	0.000000		17.365	19.584	0.1549
1099706	12057	0.000000		24.096	27.744	6.5984
5455609	45704	0.000000		33.323	38.325	32.7343
10085198	73087	0.000000		38.453	45.013	60.5124

Racemic **3r**:

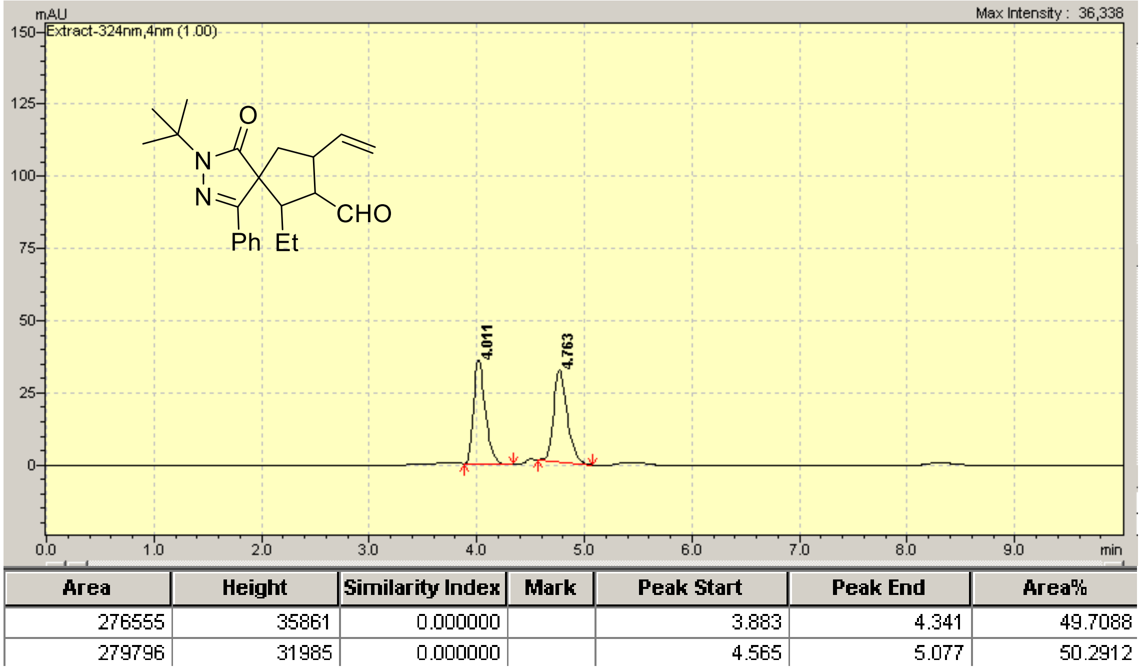

Area	Height	Similarity Index	Mark	Peak Start	Peak End	Area%
276555	35861	0.000000		3.883	4.341	49.7088
279796	31985	0.000000		4.565	5.077	50.2912

Chiral S-catalyst **3r**:

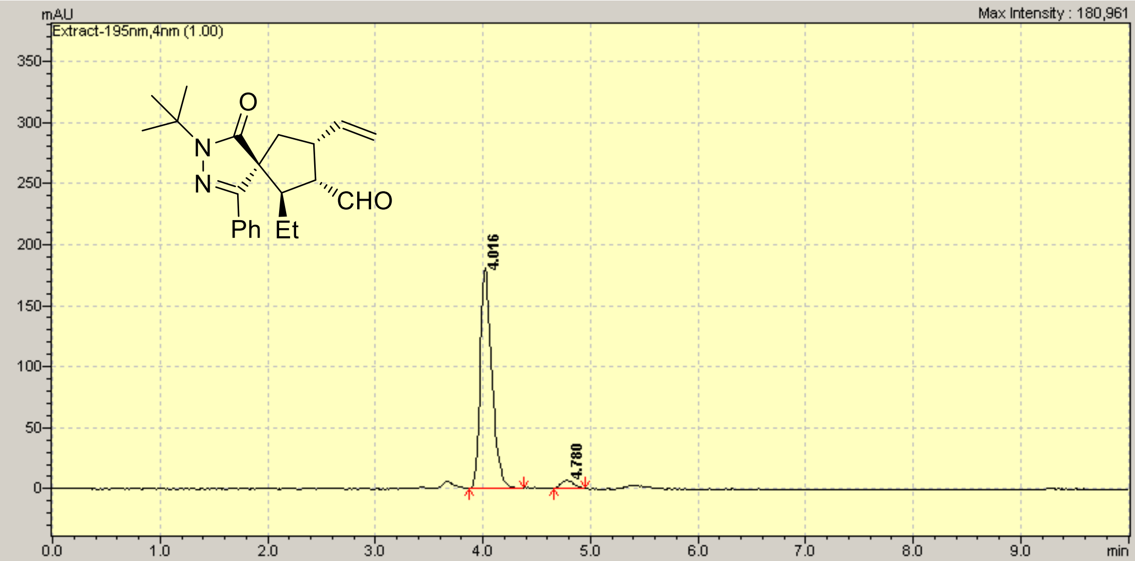

Area	Height	Similarity Index	Mark	Peak Start	Peak End	Area%
1345883	180202	0.000000		3.872	4.373	96.3107
51555	6363	0.000000		4.661	4.949	3.6893

Racemic **3s**:

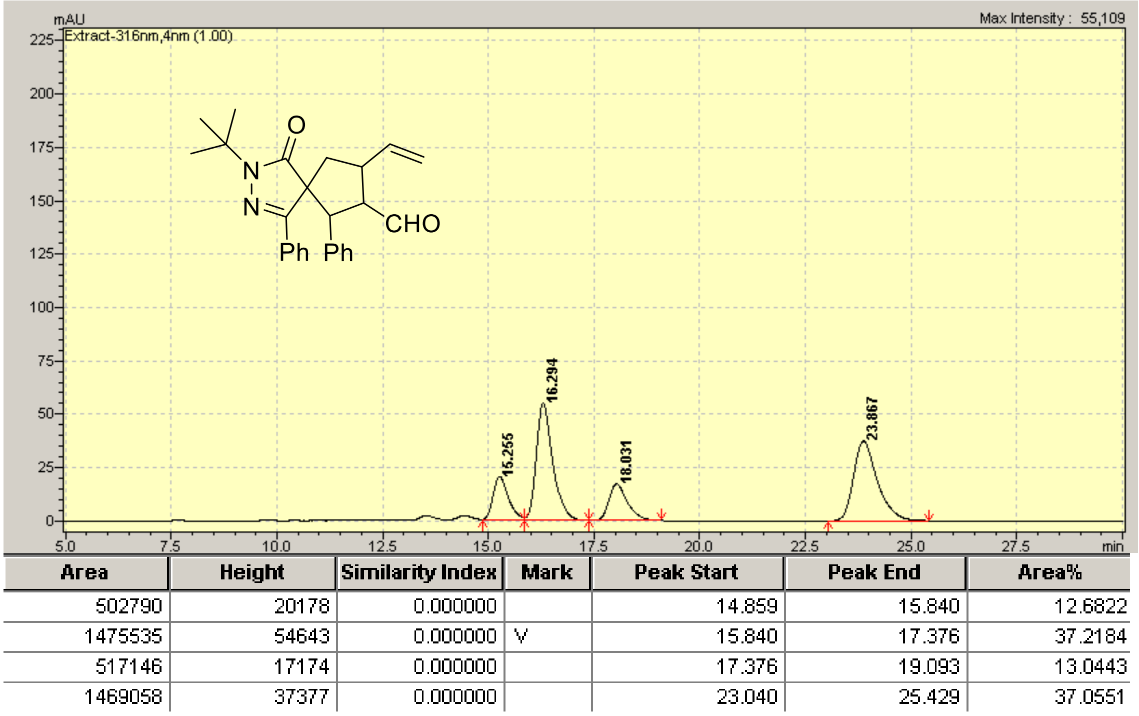

Area	Height	Similarity Index	Mark	Peak Start	Peak End	Area%
502790	20178	0.000000		14.859	15.840	12.6822
1475535	54643	0.000000	V	15.840	17.376	37.2184
517146	17174	0.000000		17.376	19.093	13.0443
1469058	37377	0.000000		23.040	25.429	37.0551

Chiral S-catalyst **3s**:

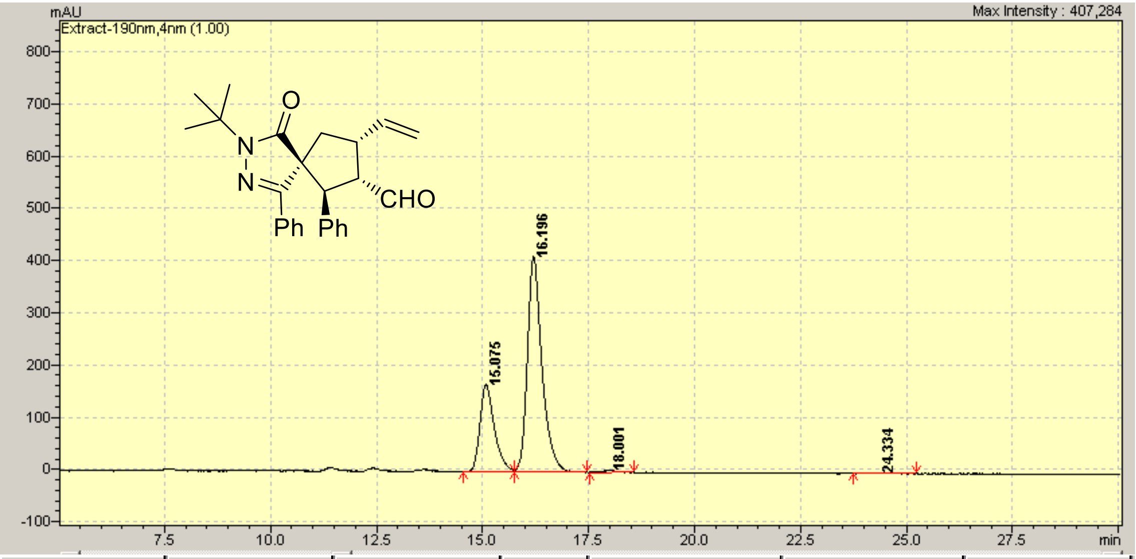

Area	Height	Similarity Index	Mark	Peak Start	Peak End	Area%
3998598	166913	0.000000		14.549	15.765	28.3883
9925266	412356	0.000000	V	15.765	17.461	70.4650
92379	3767	0.000000		17.515	18.571	0.6558
69147	2098	0.000000		23.733	25.248	0.4909

Racemic **3t**:

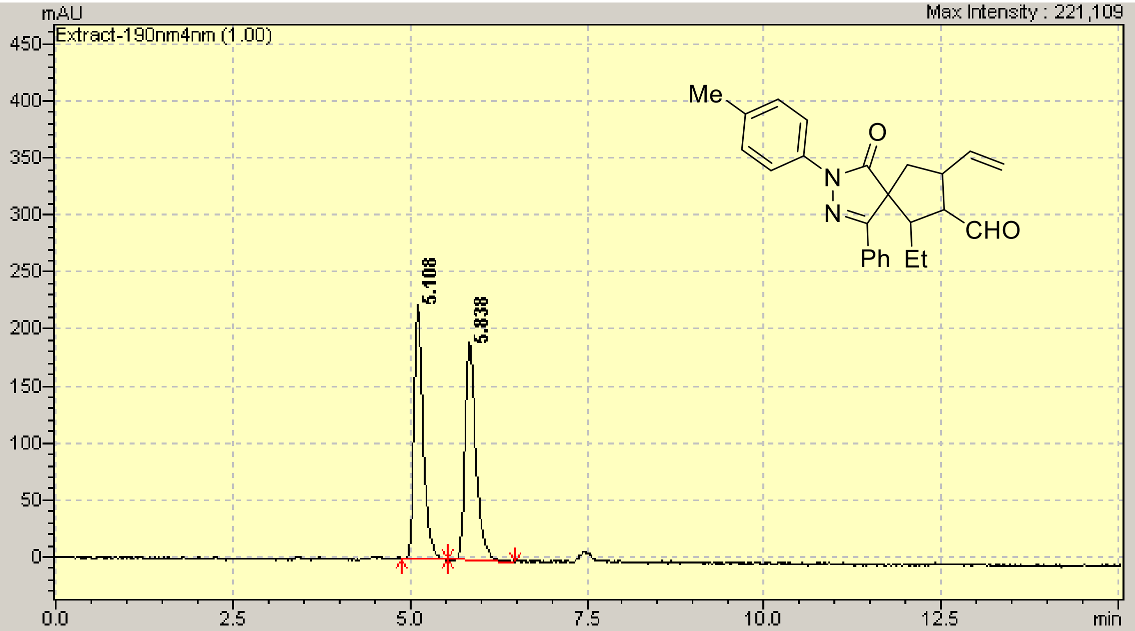

Area	Height	Similarity Index	Mark	Peak Start	Peak End	Area%
2026985	223312	0.000000		4.896	5.515	50.3935
1995327	191363	0.000000		5.621	6.272	49.6065

Chiral S-catalyst **3t**:

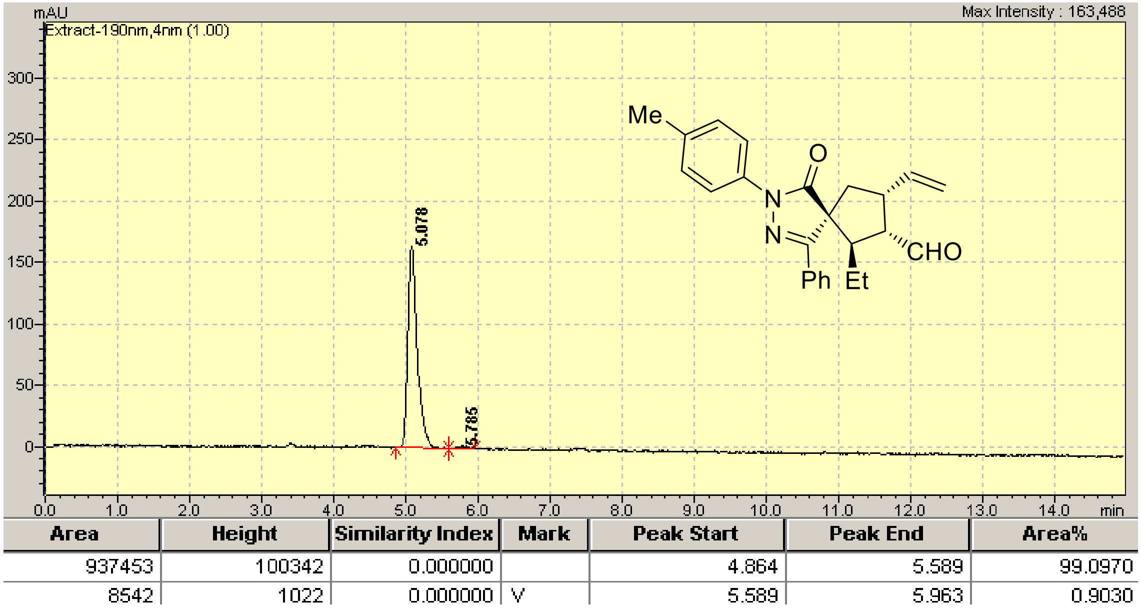

Area	Height	Similarity Index	Mark	Peak Start	Peak End	Area%
937453	100342	0.000000		4.864	5.589	99.0970
8542	1022	0.000000	V	5.589	5.963	0.9030

Racemic **3u**:

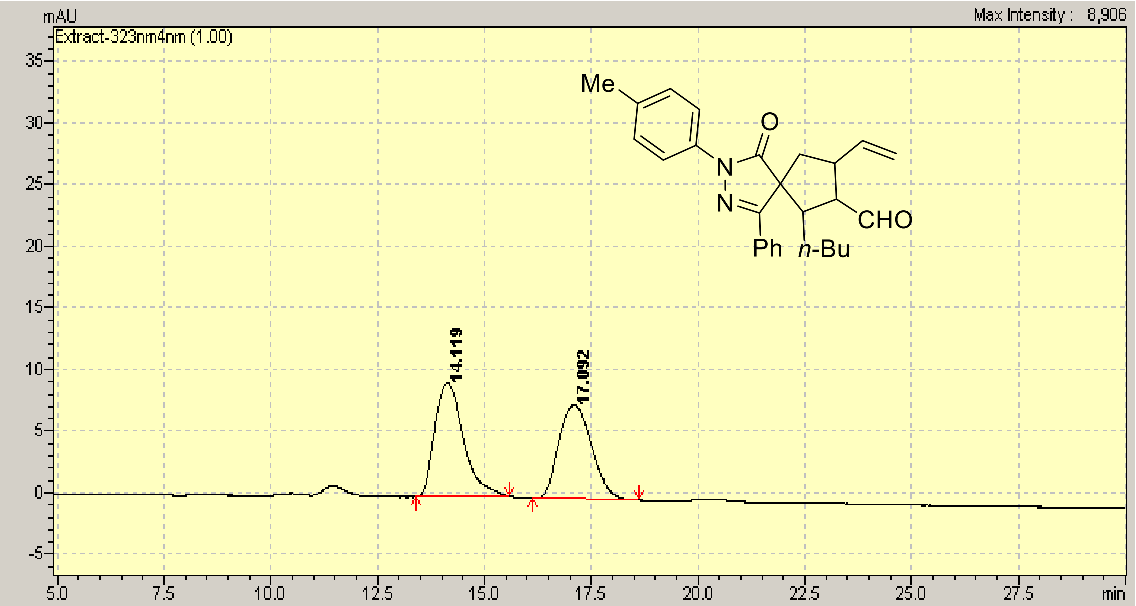

Area	Height	Similarity Index	Mark	Peak Start	Peak End	Area%
433059	9216	0.000000		13.387	15.595	51.3183
410810	7639	0.000000		16.128	18.613	48.6817

Chiral S-catalyst **3u**:

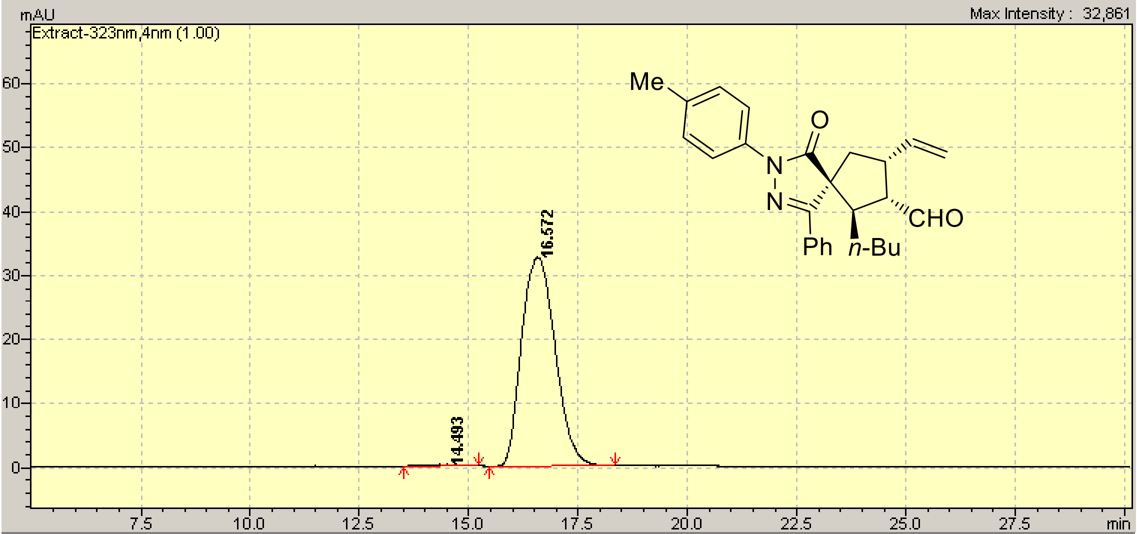

Area	Height	Similarity Index	Mark	Peak Start	Peak End	Area%
64698	1400	0.000000		13.515	15.243	0.9254
6926728	131804	0.000000		15.477	18.336	99.0746

Racemic **3v**:

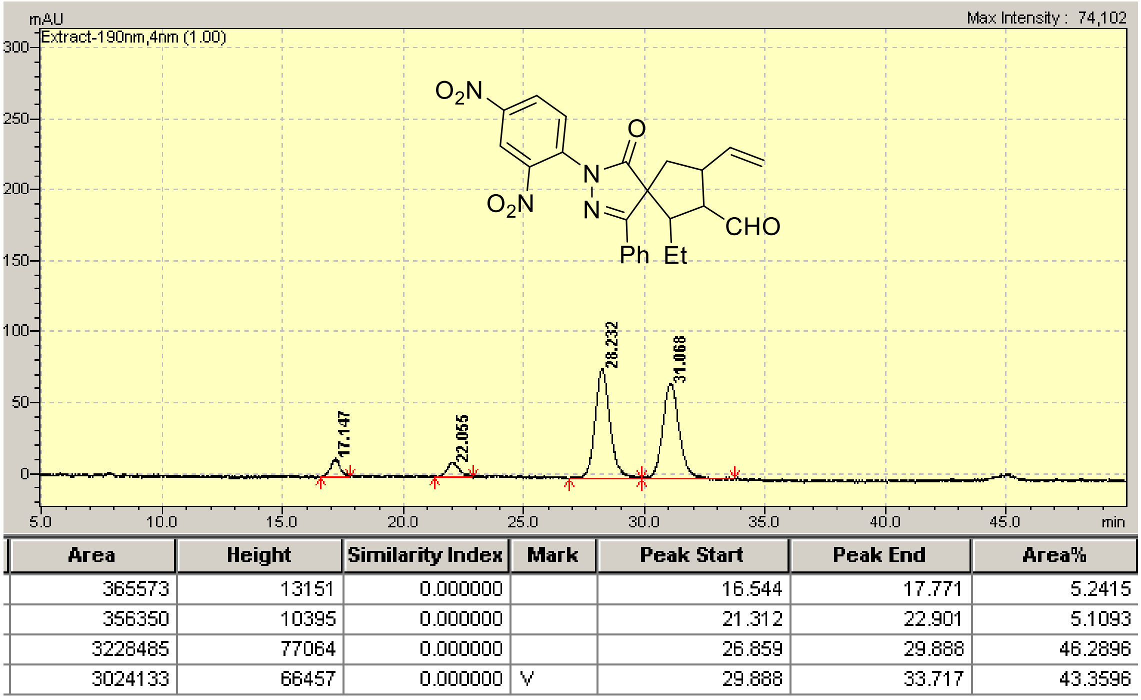

Area	Height	Similarity Index	Mark	Peak Start	Peak End	Area%
365573	13151	0.000000		16.544	17.771	5.2415
356350	10395	0.000000		21.312	22.901	5.1093
3228485	77064	0.000000		26.859	29.888	46.2896
3024133	66457	0.000000	V	29.888	33.717	43.3596

Chiral S-catalyst **3v**:

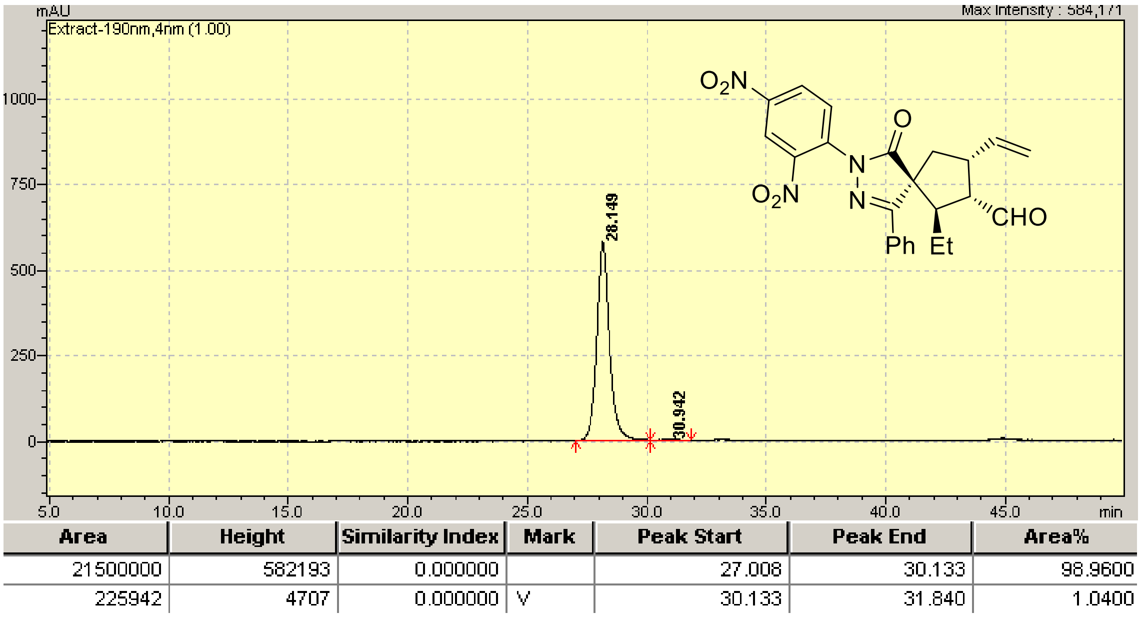

Area	Height	Similarity Index	Mark	Peak Start	Peak End	Area%
21500000	582193	0.000000		27.008	30.133	98.9600
225942	4707	0.000000	V	30.133	31.840	1.0400

Racemic **3w**:

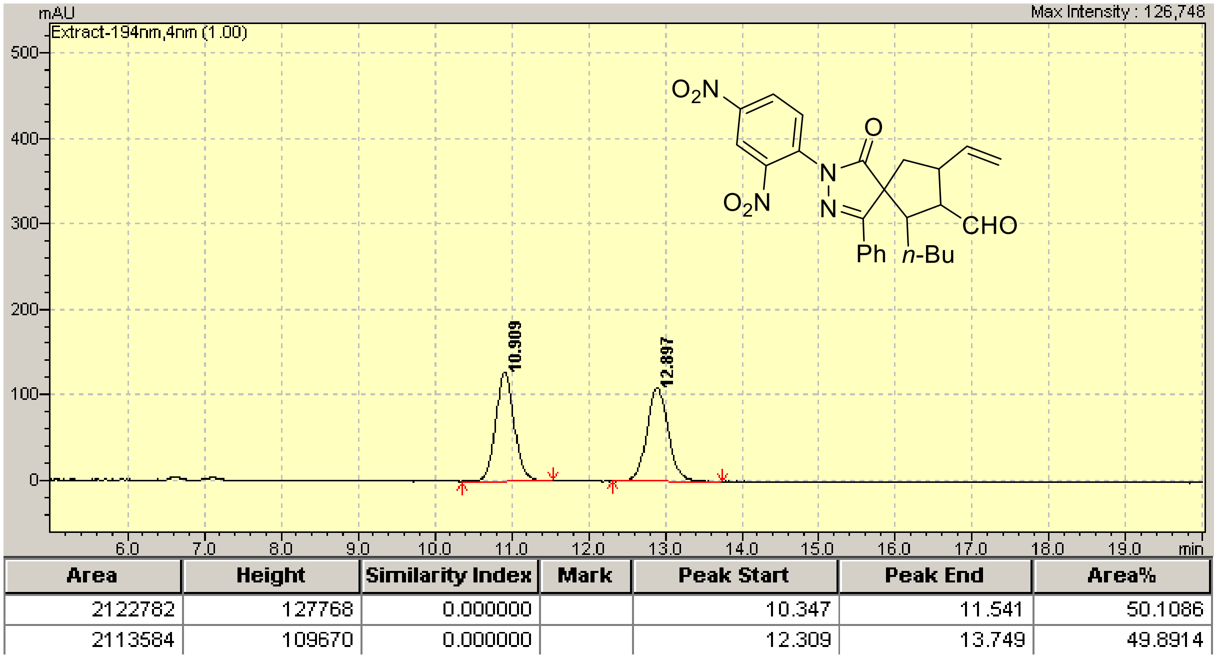

Area	Height	Similarity Index	Mark	Peak Start	Peak End	Area%
2122782	127768	0.000000		10.347	11.541	50.1086
2113584	109670	0.000000		12.309	13.749	49.8914

Chiral S-catalyst **3w**:

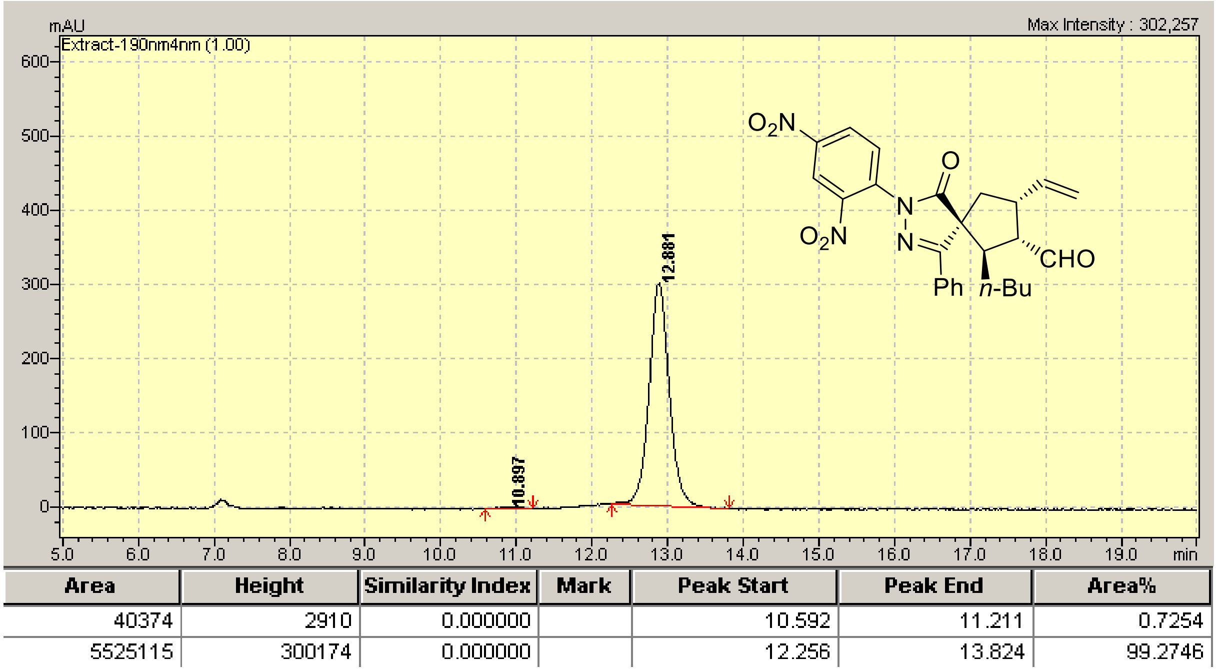

Area	Height	Similarity Index	Mark	Peak Start	Peak End	Area%
40374	2910	0.000000		10.592	11.211	0.7254
5525115	300174	0.000000		12.256	13.824	99.2746

Racemic **3x**:

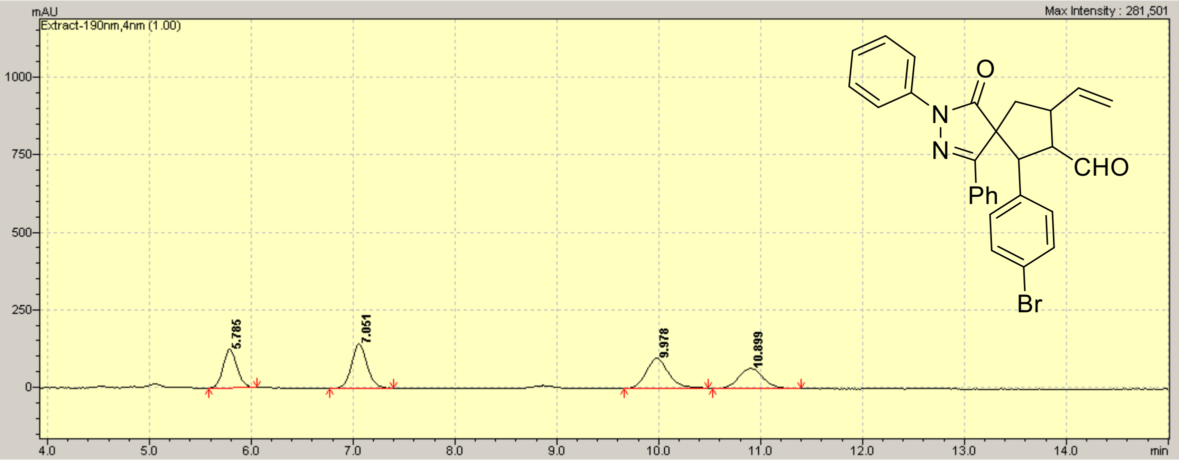

Area	Height	Similarity Index	Mark	Peak Start	Peak End	Area%
1126987	123830	0.000000		5.579	6.059	21.4379
1541008	144383	0.000000		6.773	7.392	29.3136
1491793	96904	0.000000		9.664	10.485	28.3774
1097186	65124	0.000000		10.528	11.403	20.8710

Chiral S-catalyst **3x**:

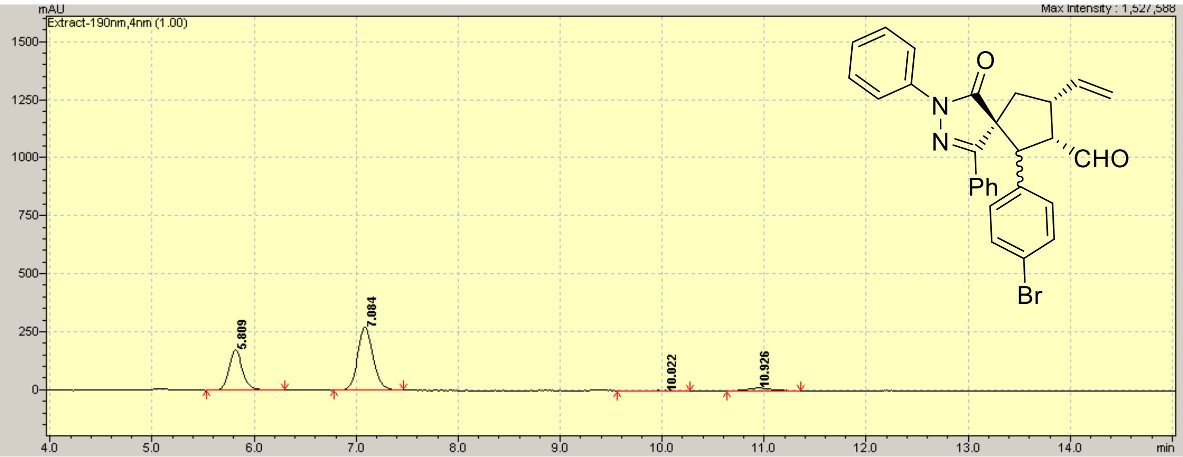

Area	Height	Similarity Index	Mark	Peak Start	Peak End	Area%
1643277	173732	0.000000		5.536	6.304	34.6727
2860663	271301	0.000000		6.784	7.467	60.3593
21263	1525	0.000000		9.557	10.272	0.4486
214191	12622	0.000000		10.635	11.360	4.5194

Racemic **11**:

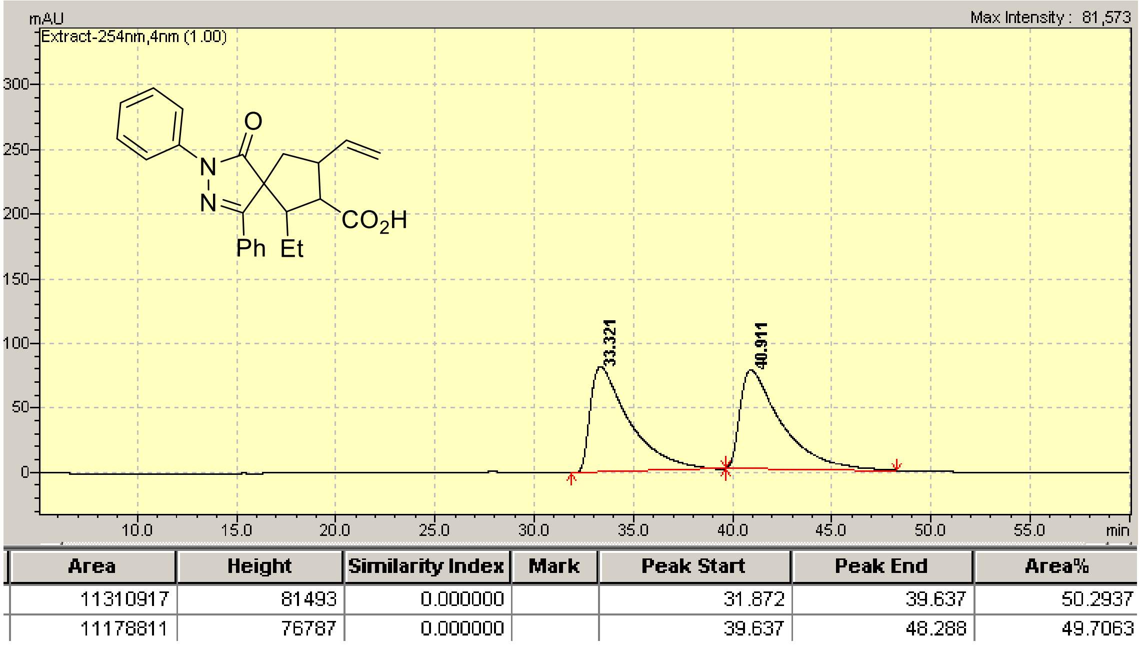

Area	Height	Similarity Index	Mark	Peak Start	Peak End	Area%
11310917	81493	0.000000		31.872	39.637	50.2937
11178811	76787	0.000000		39.637	48.288	49.7063

Chiral **11**:

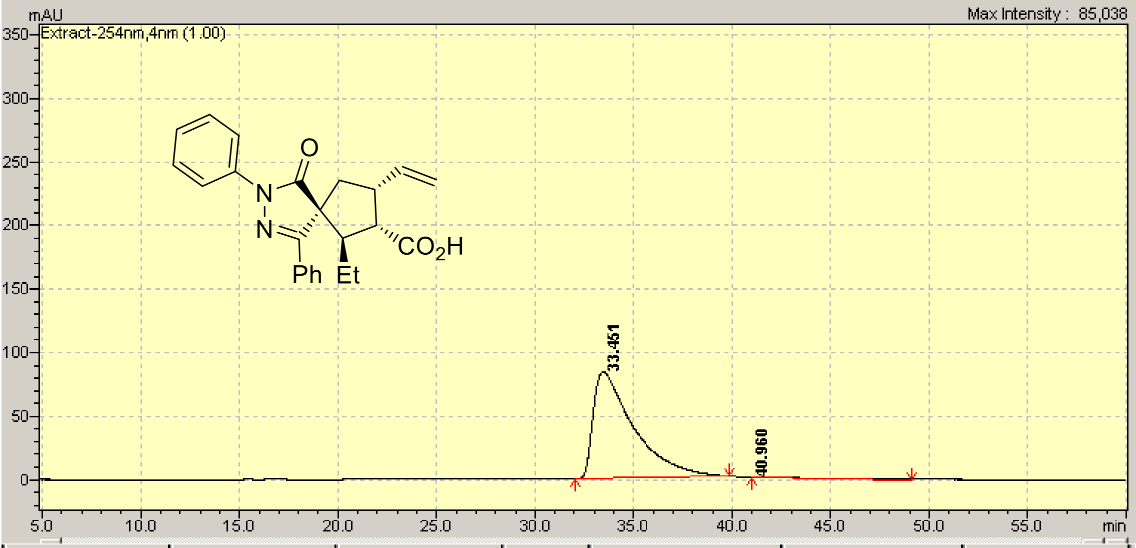

Area	Height	Similarity Index	Mark	Peak Start	Peak End	Area%
12983959	90367	0.000000		32.053	39.840	99.6697
43026	265	0.000000		40.960	49.120	0.3303

Racemic **12 dia 1**:

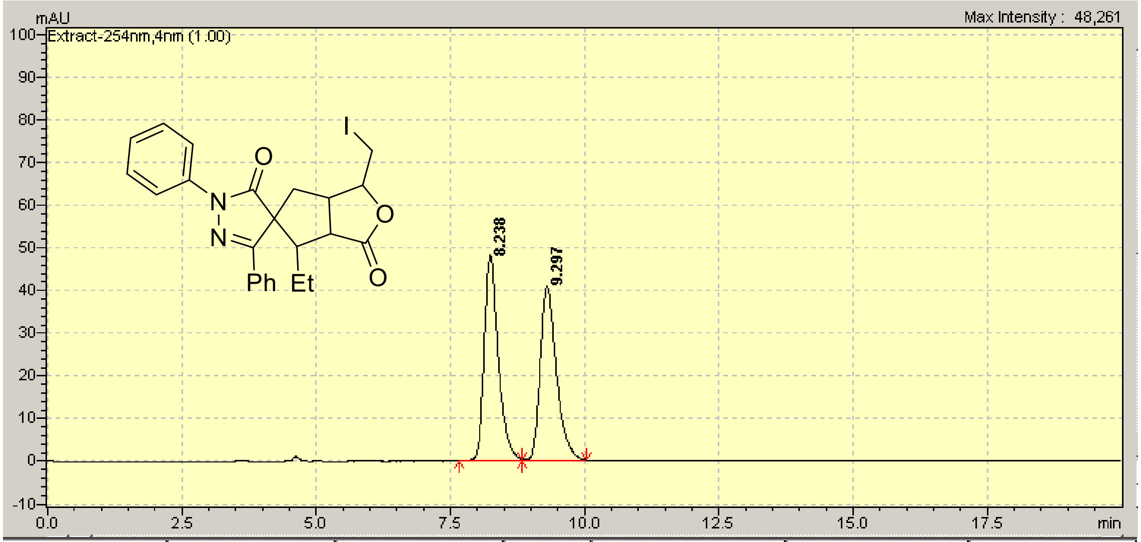

Area	Height	Similarity Index	Mark	Peak Start	Peak End	Area%
872585	48141	0.000000		7.659	8.832	50.5706
852892	40847	0.000000	V	8.832	10.037	49.4294

Chiral **12 dia 1**:

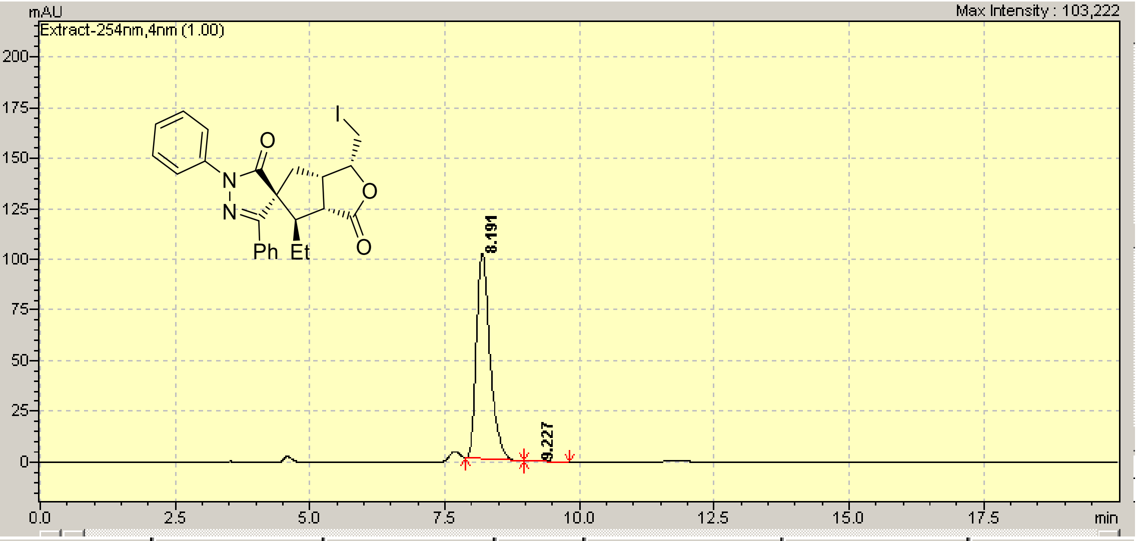

Area	Height	Similarity Index	Mark	Peak Start	Peak End	Area%
1777106	101839	0.000000		7.883	8.960	99.4034
10666	658	0.000000		8.981	9.824	0.5966

Racemic **12 dia 2**:

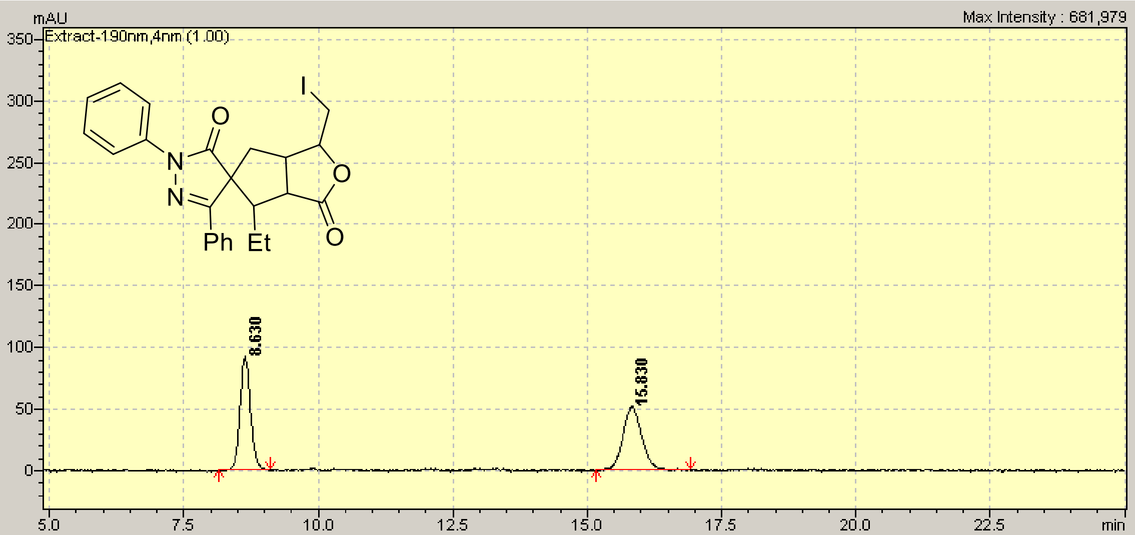

Area	Height	Similarity Index	Mark	Peak Start	Peak End	Area%
1228241	91874	0.000000		8.160	9.120	49.9880
1228829	51136	0.000000		15.157	16.917	50.0120

Racemic **12 dia 2**:

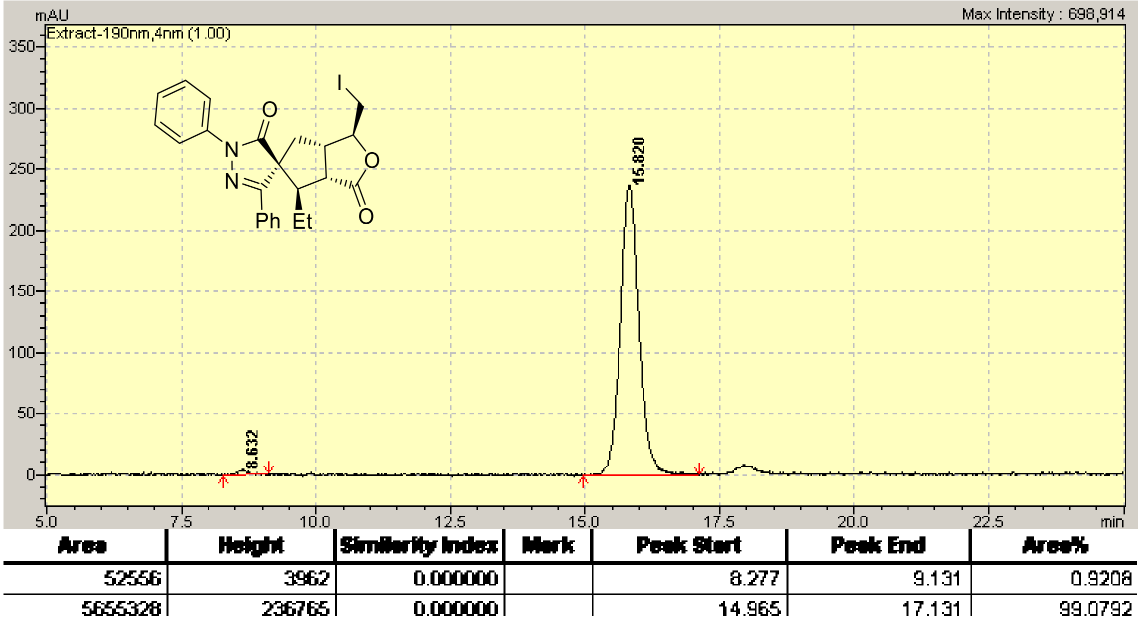

Area	Height	Similarity Index	Mark	Peak Start	Peak End	Area%
52556	3962	0.000000		8.277	9.131	0.9208
5655328	236765	0.000000		14.965	17.131	99.0792

Racemic **13**:

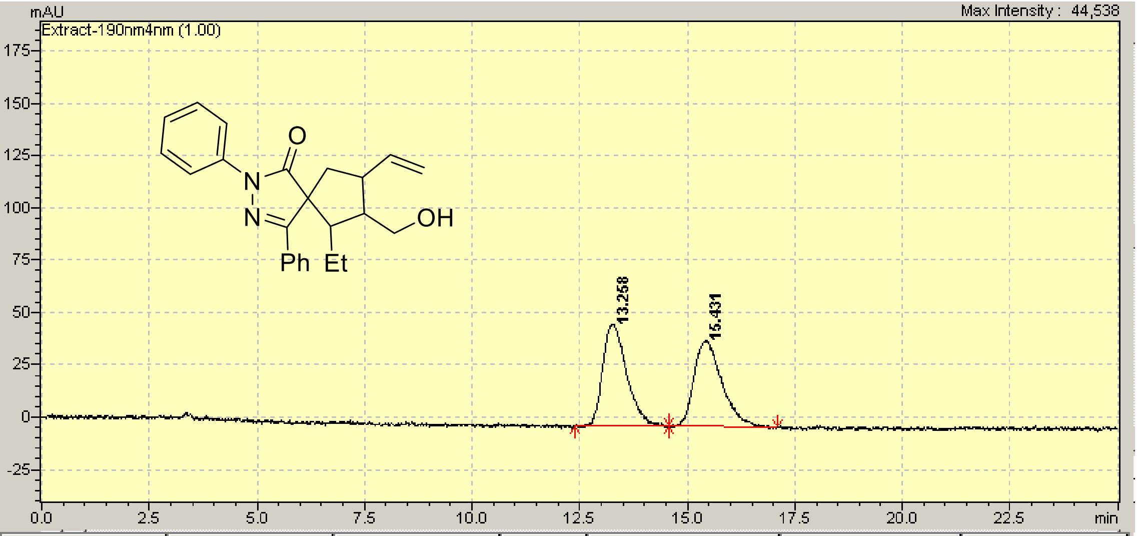

Area	Height	Similarity Index	Mark	Peak Start	Peak End	Area%
1872940	48822	0.000000		12.384	14.571	50.1940
1858466	41228	0.000000	V	14.571	17.099	49.8060

Chiral **13**:

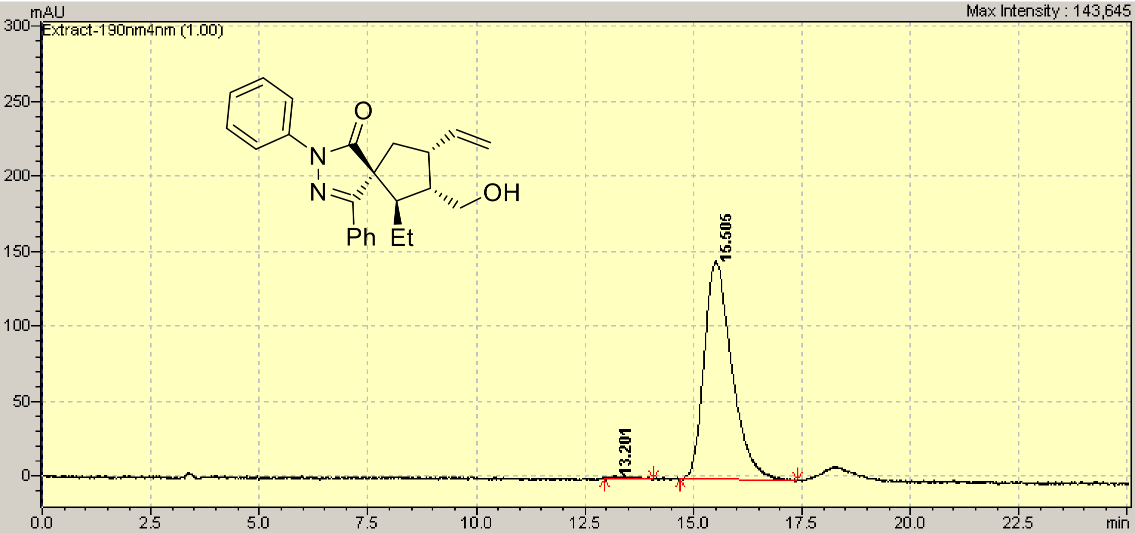

Area	Height	Similarity Index	Mark	Peak Start	Peak End	Area%
40640	1641	0.000000		12.949	14.069	0.6268
6443032	145642	0.000000		14.709	17.397	99.3732

Racemic **14**:

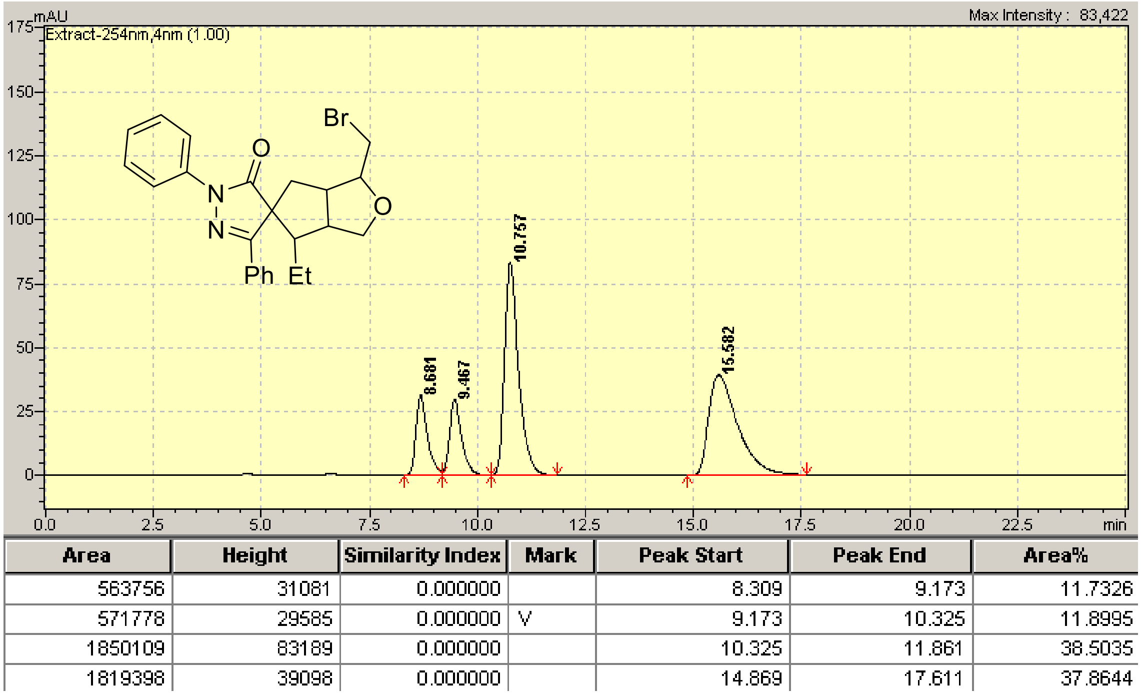

Area	Height	Similarity Index	Mark	Peak Start	Peak End	Area%
563756	31081	0.000000		8.309	9.173	11.7326
571778	29585	0.000000	V	9.173	10.325	11.8995
1850109	83189	0.000000		10.325	11.861	38.5035
1819398	39098	0.000000		14.869	17.611	37.8644

Chiral **14**:

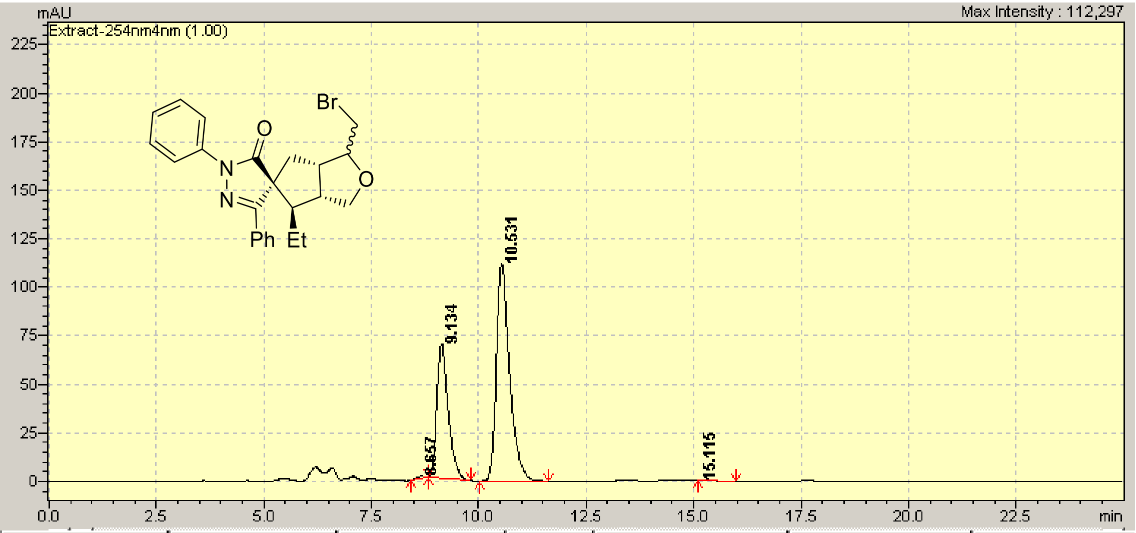

Area	Height	Similarity Index	Mark	Peak Start	Peak End	Area%
13401	976	0.000000		8.427	8.811	0.3659
1252245	69188	0.000000		8.821	9.824	34.1865
2387715	112082	0.000000		10.027	11.605	65.1851
9618	318	0.000000		15.104	15.968	0.2626

Racemic **16**:

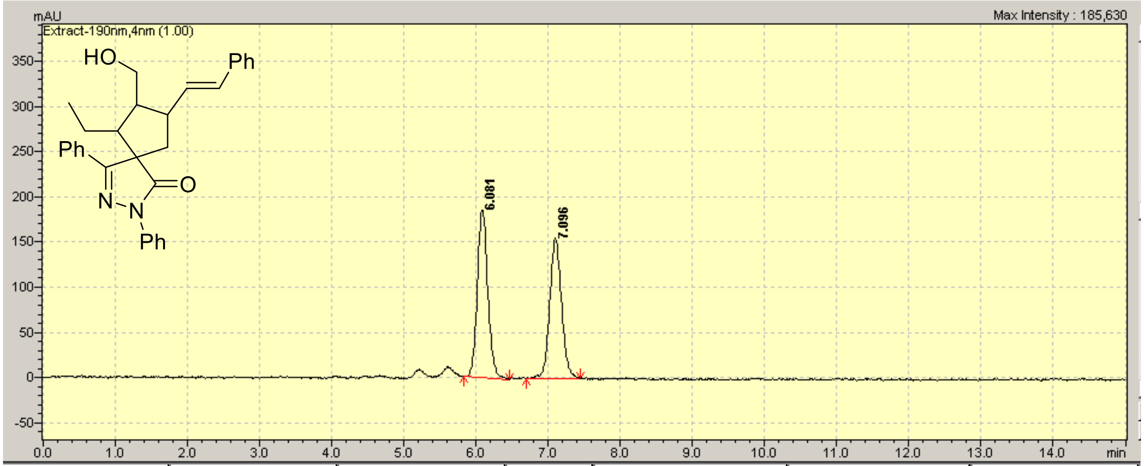

Area	Height	Similarity Index	Mark	Peak Start	Peak End	Area%
1890192	185834	0.000000		5.835	6.464	51.4902
1780784	155406	0.000000		6.699	7.445	48.5098

Chiral **16**:

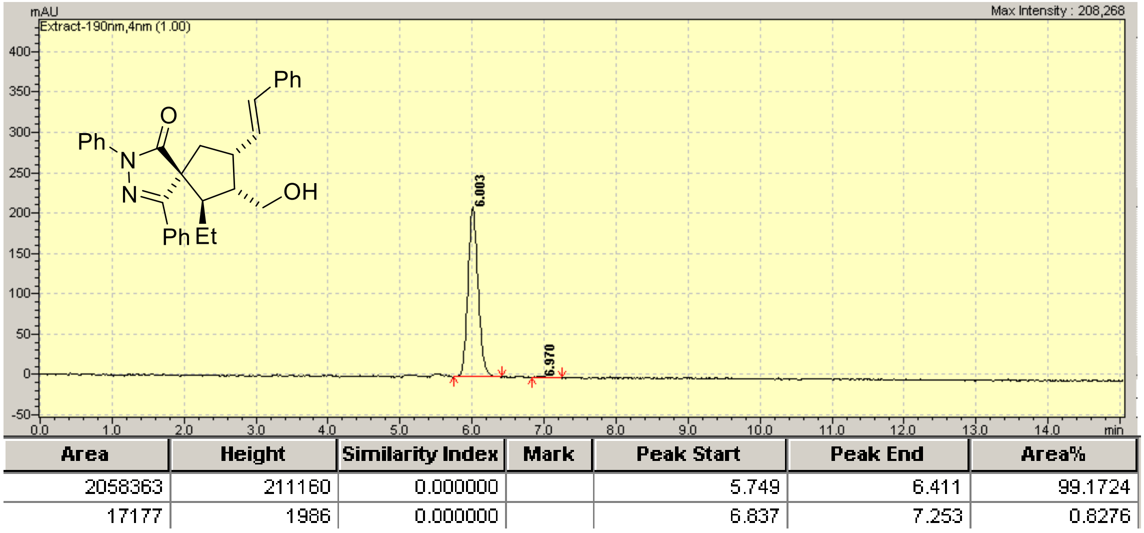

Area	Height	Similarity Index	Mark	Peak Start	Peak End	Area%
2058363	211160	0.000000		5.749	6.411	99.1724
17177	1986	0.000000		6.837	7.253	0.8276

Racemic **17**:

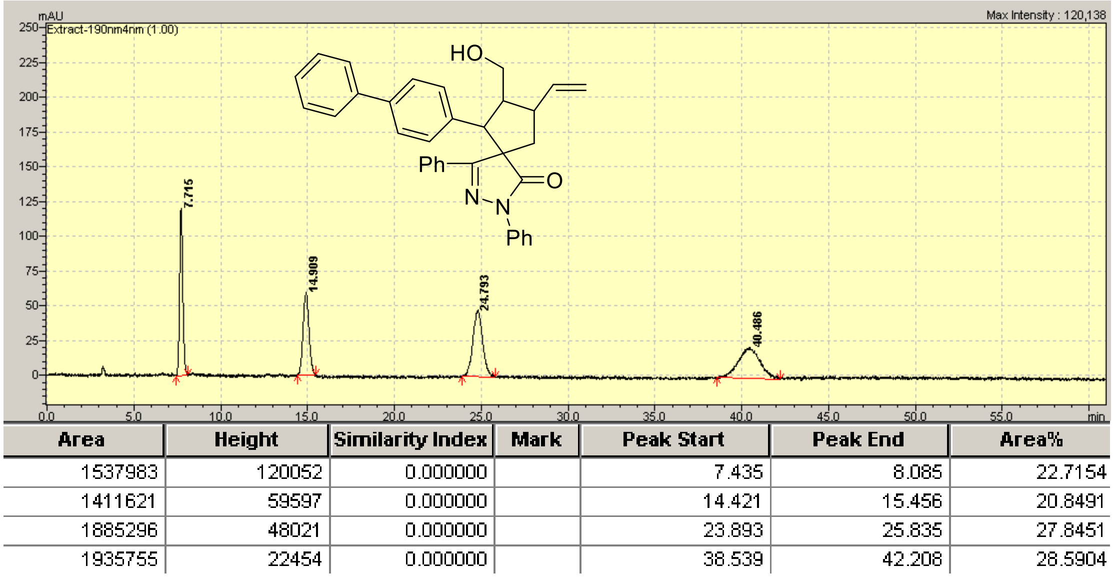

Area	Height	Similarity Index	Mark	Peak Start	Peak End	Area%
1537983	120052	0.000000		7.435	8.085	22.7154
1411621	59597	0.000000		14.421	15.456	20.8491
1885296	48021	0.000000		23.893	25.835	27.8451
1935755	22454	0.000000		38.539	42.208	28.5904

Chiral **17**:

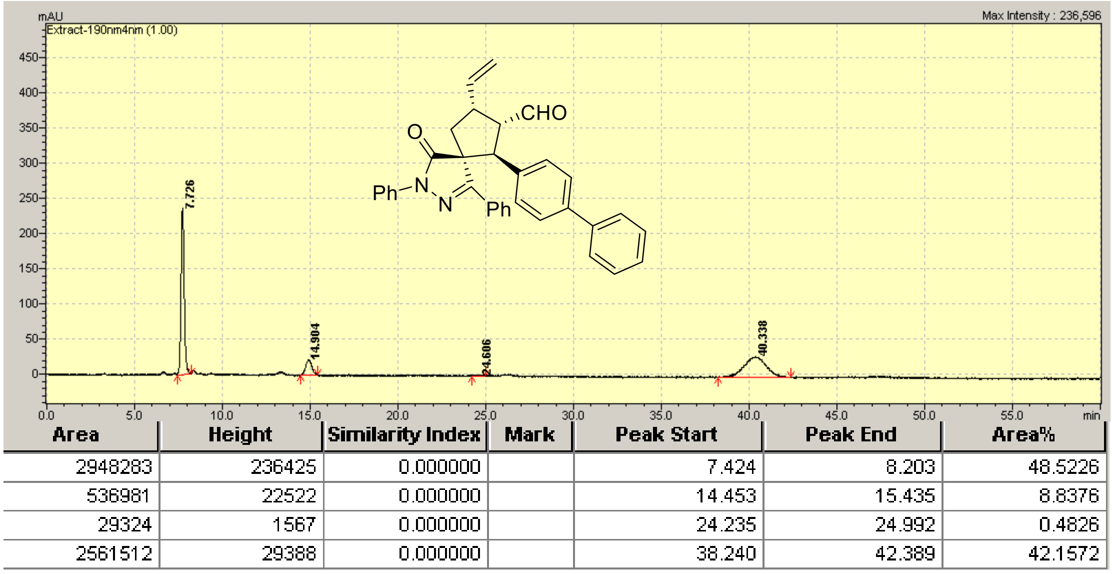

Area	Height	Similarity Index	Mark	Peak Start	Peak End	Area%
2948283	236425	0.000000		7.424	8.203	48.5226
536981	22522	0.000000		14.453	15.435	8.8376
29324	1567	0.000000		24.235	24.992	0.4826
2561512	29388	0.000000		38.240	42.389	42.1572

9. Mass spectrometry experiments

Preparation of reaction mixtures for mass spectrometry experiments

A) The solution of the catalyst was prepared by dissolving 2 mg of $Pd_2(dba)_3$ (5 mol% equiv) in 2 ml of dichloromethane. The solution of catalysts was filtered. The solution of reactant was prepared by dissolving 20 mg of pyrazolone **1a** (2 equiv, 0.09 mmol), 6 mg of cinnamaldehyde (1 equiv, 0.045 mmol), and 3 mg of Jorgensen (2-(diphenyl((trimethylsilyl)oxy)methyl)pyrrolidine) (20 mol% equiv, 0.009 mmol) in 2 ml of acetonitrile. The final solution was prepared by mixing of 0.5 ml of the solution of catalyst, 0.5 ml of the solution of reactant **1a**, Jorgensen-Hayashi catalyst, cinnamaldehyde, and 1.5 ml of acetonitrile.

B) The solution of the catalyst was prepared by dissolving 2 mg of $Pd_2(dba)_3$ (2.5 mol% equiv) in 2 ml of dichloromethane. The solution of catalysts was filtered. The solution of reactant was prepared by dissolving 20 mg of pyrazolone **1a** (1 equiv, 0.09 mmol) in 2 ml of acetonitrile. The final solution was prepared by mixing of 0.5 ml of the solution of catalyst, 0.5 ml of the solution of reactant, and 1.5 ml of acetonitrile.

Mass spectrometric experiments

The experiments were performed with a TSQ 7000 mass spectrometer with a quadrupole-octopole-quadrupole configuration. The ions were generated by electrospray ionization (ESI) from the reaction mixtures described above at soft ionization condition (low potentials on the entrance ion optics and the temperature of the capillary was 200 °C).

Results of mass spectrometry experiments

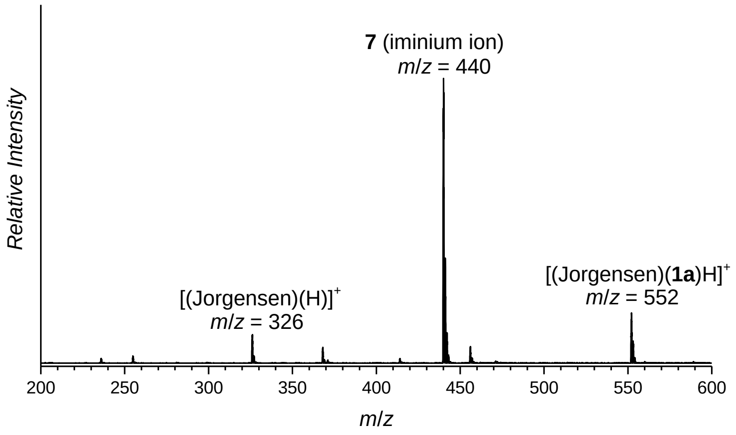

Figure S20. ESI-MS source spectrum of the solution of pyrazolone (**1a**), Jorgensen catalyst, cinnamaldehyde, and $Pd_2(dba)_3$ in acetonitrile.

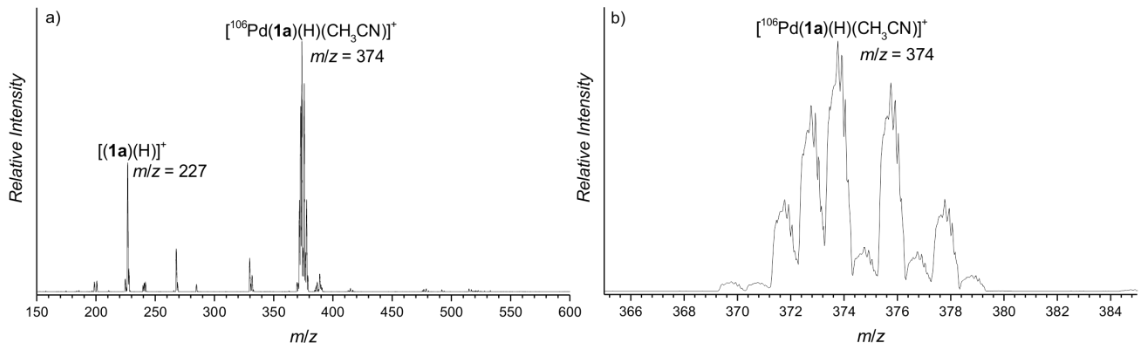

Figure S21. a) ESI-MS source spectrum of the final solution of pyrazolone (**1a**) with the catalyst $Pd_2(dba)_3$ in acetonitrile. b) Isotope pattern of the complex [Pd(**1a**)(H)(CH_3CN)]$^+$ (*m/z* = 374).

10. Infrared photodissociation (IRPD) spectroscopy and theoretical calculations

IRPD experiments

The experiments were performed with the ISORI instrument. ISORI is based on the combination of a low-temperature ion trap with a commercial TSQ 7000 instrument. In its original design TSQ 7000 has a quadrupole-octopole-quadrupole geometry. The original ion source vacuum chamber is connected to the main instrument via a customized flange. It preserves all of the ionization options provided by TSQ – here the electrospray ionization equipment is used. The new ultra-high vacuum chamber consists of three additional differentially pumped regions: 1) the region with the first quadrupole ($4P_1$), the quadrupole bender (QPB), and the octopole (8P), 2) the region with the ion trap (w4PT), and 3) the second quadrupole ($4P_2$) and the detector. The ion trap has a linear quadrupole geometry where the hyperbolic shape of each electrode is approximated by six wires. It is mounted into a copper box, which is screwed onto a cold-head. The copper box reaches temperature of 2.6 K. Cooling of the ions is achieved by collisions with a helium buffer gas. The temperature of the box, surrounding the trap, and of the heat shield is measured by silicon-diode sensors. The buffer gas is injected by a custom-made piezo valve, situated in vacuum, directly into the trap with a straight Teflon tube. The presence of the He buffer gas leads to elevation of the trap temperature via convective heat transfer by several tens of Kelvin.

The ions of interest were obtained by electrospray ionization of final solution, which is described above. The ions of interest were mass-selected by the $4P_1$, deflected by the QPB, and guided by the 8P to the w4PT. During the first 300 ms of the 1 Hz trapping cycle the ions were guided into the trap and simultaneously, He buffer gas has been injected. The maximum He number density was on the order of 10^{15} cm^{-3}. During the trapping time, the ions internally relaxed and formed helium-tagged complexes (typically, we have 1 – 3 % yield of He tagged complexes for closed shell ions produced by electrospray ionization). After 300 ms the ion filling of the trap was stopped (He valve was closed and the ions were deflected by QPB). The trapped ions were then irradiated. At 990 ms, the exit electrode of the trap was opened, the ions were mass-analyzed by the $4P_2$, and detected by a Daly type detector operated in ion-counting mode.

For IRPD spectroscopy, we used radiation of a pulsed (10 Hz repetition rate) OPO/OPA system (LaserVision, tuning range 700 – 4700 cm^{-1}, FWHM ~1.5 cm^{-1}, 10 ns pulse length). The OPO is pumped by Nd:YAG laser (Surelite EX from Continuum). The photon beam was focused into the trap by gold-coated parabolic mirrors and entered the vacuum chamber through the ZnSe-window mounted on the detector side. Energy of the photon pulses was controlled by attenuation of the pump energy delivered to the OPA stage and measured by laser energy meter Coherent Fieldmax II with J-25MB-LE sensor. The pulse energy of the light passing through the instrument and coming out from the CaF_2 window on QPB chamber was monitored routinely during acquiring of the spectra.

Computational Details

The calculations were performed using the density functional theory method B3LYP-D3 together with the SDD basis set for palladium and 6-311++G** for the remaining atoms as implemented in the Gaussian 09 suite. Computation of the Hessian matrix was performed for all optimized structures at the same level of theory in order to ensure that the structures correspond to genuine minima as well as to calculate the thermochemical data and IR spectra. We used two different scaling factors. The finger print region was scaled by a factor of 0.980. The region of C-H stretching vibrations was scaled by a factor of 0.963. Note that we used a different scaling factor (0.956) for the N-H vibration in the manuscript.

We have also tested different levels of theory: PBEPBE, M06L, mPW1PW91, and MN12SX (Figure S27).

Comparison of experimental IRPD with the calculated spectra

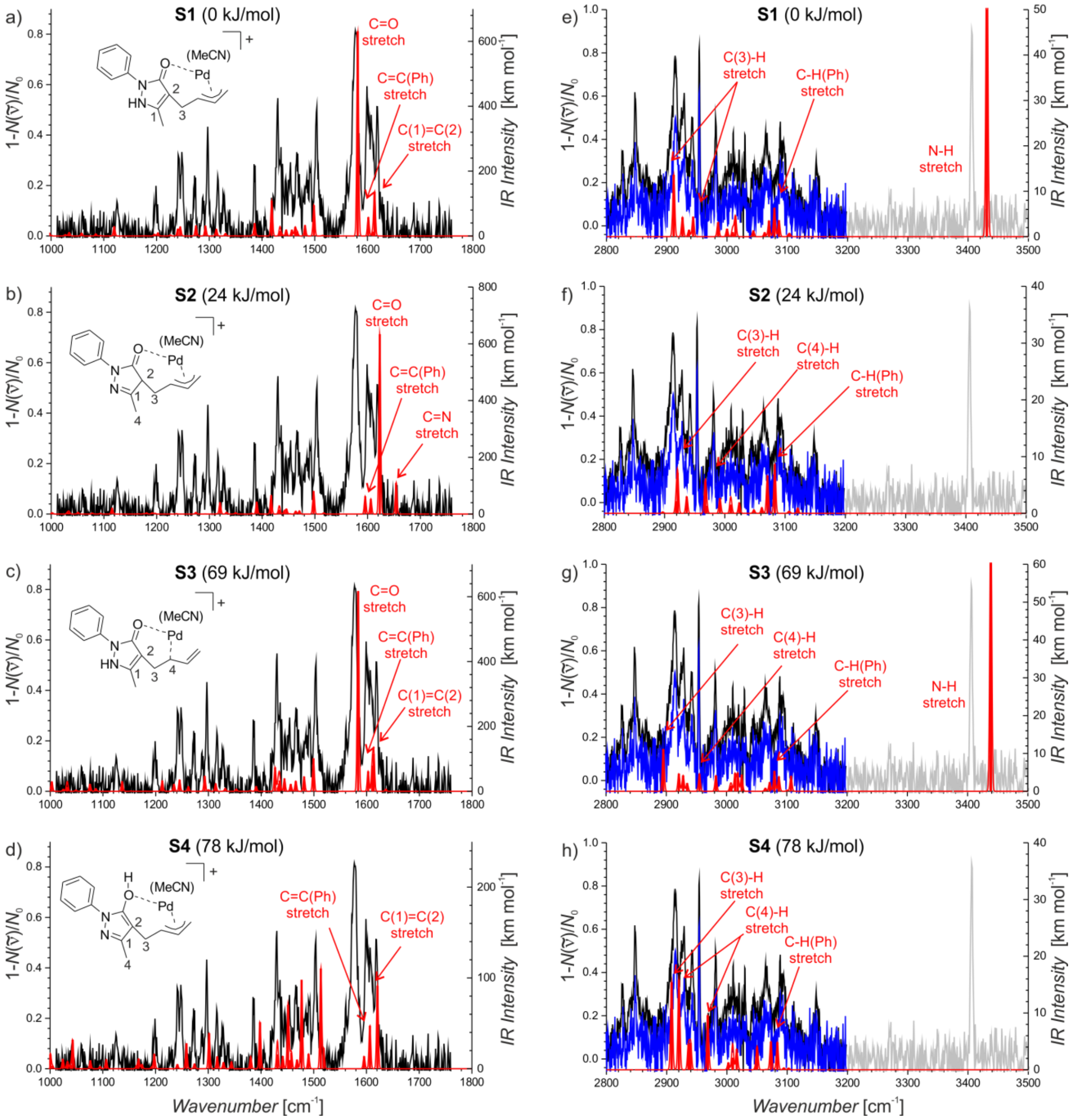

Figure S22. IRPD spectra of [^{105}Pd(**1a**)(H)(CH_3CN)]$^+$ (m/z = 373) are in black, blue, and grey. Theoretical IR spectra of **S1** (a,e), **S2** (b,f), **S3** (c,g), and **S4** (d,h) calculated at the B3LYP-D3/6-311++G**(SDD for Pd) level of theory are in red. The finger print region was scaled by a factor of 0.980. The region of C-H stretching vibrations was scaled by a factor of 0.963. Note that we used a different scaling factor (0.956) for the N-H vibration in the manuscript.

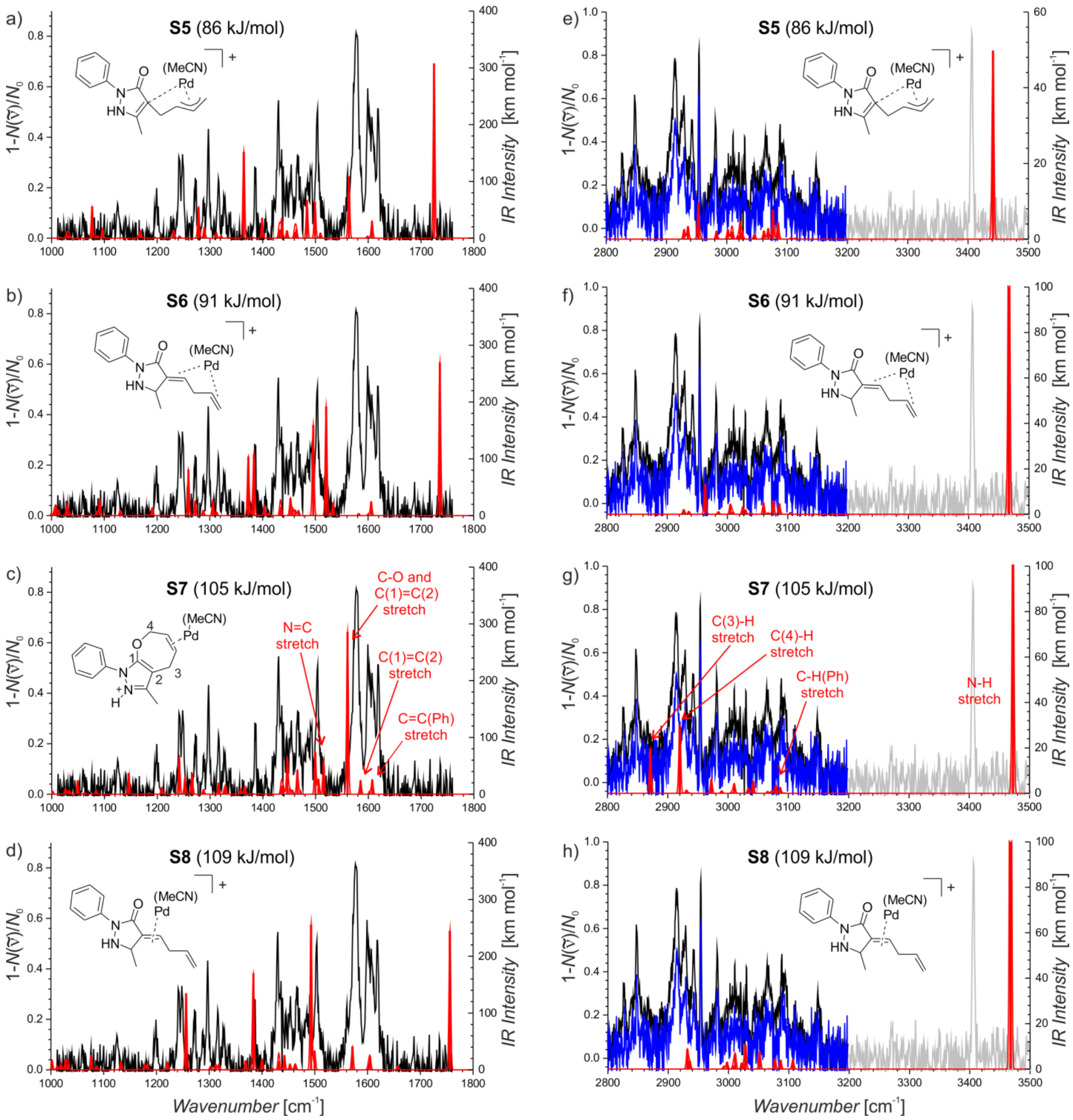

Figure S23. IRPD spectra of [^{105}Pd(**1**)(H)(CH$_3$CN)]$^+$ (m/z = 373) are in black, blue, and grey. Theoretical IR spectra of **S5** (a,e), **S6** (b,f), **S7** (c,g), and **S8** (d,h) calculated at the B3LYP-D3/6-311++G**(SDD for Pd) level of theory are in red. The finger print region was scaled by a factor of 0.980. The region of C-H stretching vibrations was scaled by a factor of 0.963. Note that we used a different scaling factor (0.956) for the N-H vibration in the manuscript.

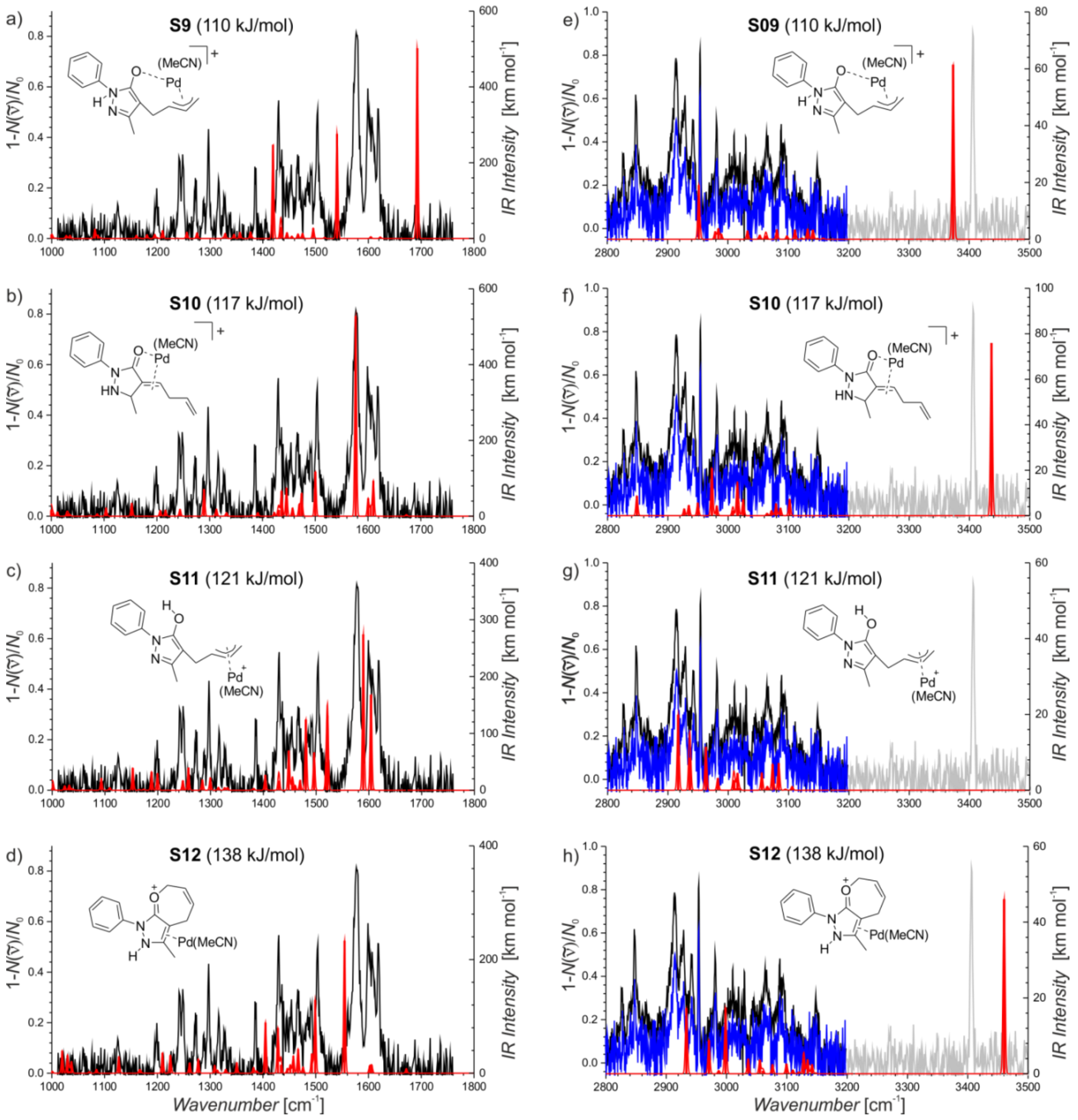

Figure S24. IRPD spectra of [^{105}Pd(**1a**)(H)(CH_3CN)]$^+$ (m/z = 373) are in black, blue, and grey. Theoretical IR spectra of **S9** (a,e), **S10** (b,f), **S11** (c,g), and **S12** (d,h) calculated at the B3LYP-D3/6-311++G**(SDD for Pd) level of theory are in red. The finger print region was scaled by a factor of 0.980. The region of C-H stretching vibrations was scaled by a factor of 0.963. Note that we used a different scaling factor (0.956) for the N-H vibration in the manuscript.

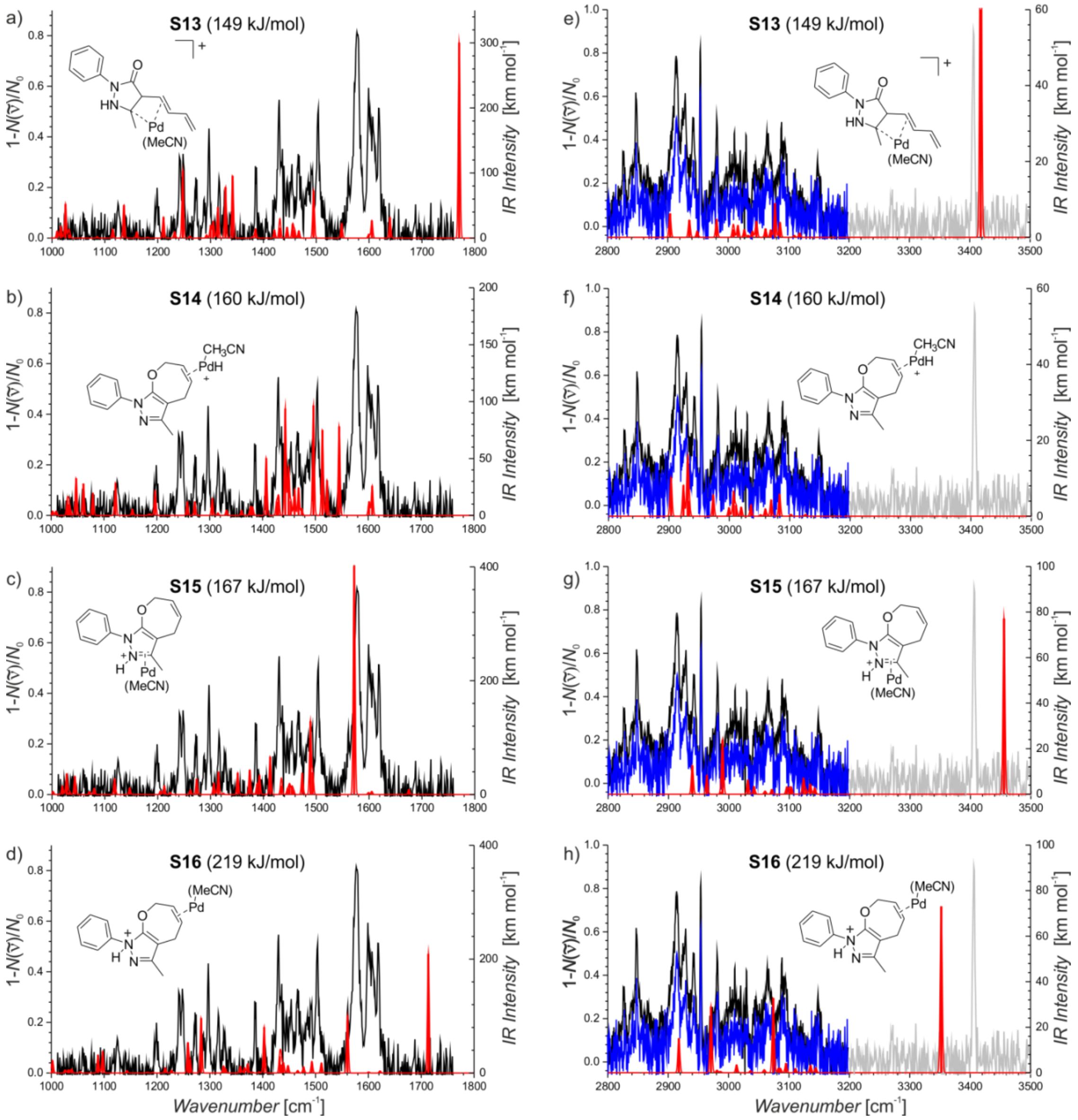

Figure S25. IRPD spectra of [^{105}Pd(**1a**)(H)(CH_3CN)]$^+$ (m/z = 373) are in black, blue, and grey. Theoretical IR spectra of **S13** (a,e), **S14** (b,f), **S15** (c,g), and **S16** (d,h) calculated at the B3LYP-D3/6-311++G**(SDD for Pd) level of theory are in red. The finger print region was scaled by a factor of 0.980. The region of C-H stretching vibrations was scaled by a factor of 0.963. Note that we used a different scaling factor (0.956) for the N-H vibration in the manuscript.

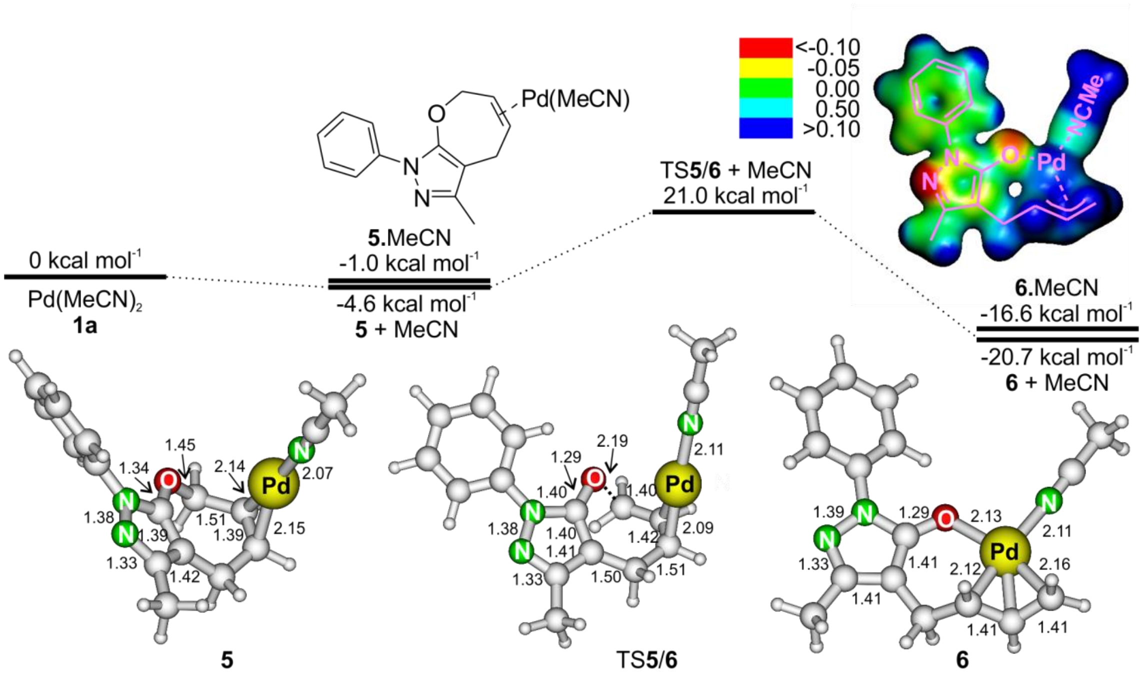

Figure S26. Potential energy surface for the insertion of palladium into the C-O bond (method: B3LYP-D3/6-311++G**(SDD for Pd) and implicit solvation in acetonitrile with the SMD model). The ball and stick models show structures of intermediates **5** and **6**; the distances are in Å. Electrostatic potential map for **6** is color-coded on the isodensity surface ρ = 0.02 e Å^{-3}.

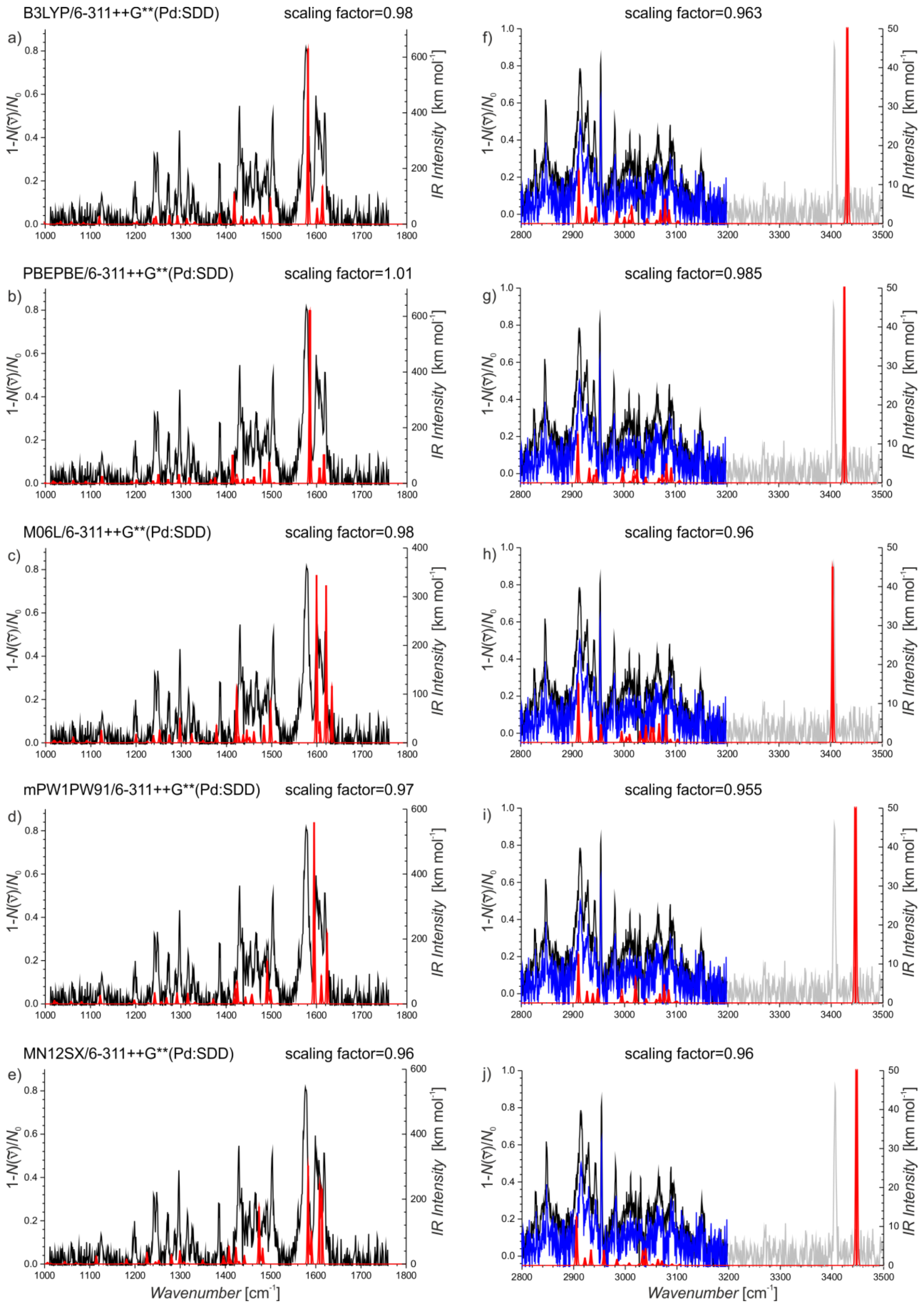

Figure S27. IRPD spectra of [^{105}Pd(**1a**)(H)(CH_3CN)]$^+$ (m/z = 373) are in black, blue, and grey. Theoretical IR spectra of **S1** calculated at different levels of theory ((a,f) B3LYP-D3, (b,g) PBEPBE-D3, (c,h) M06L-D3, (d,i) mPW1PW91, (e,j) MN12SX) with basis set 6-311++G**(SDD for Pd) are in red.

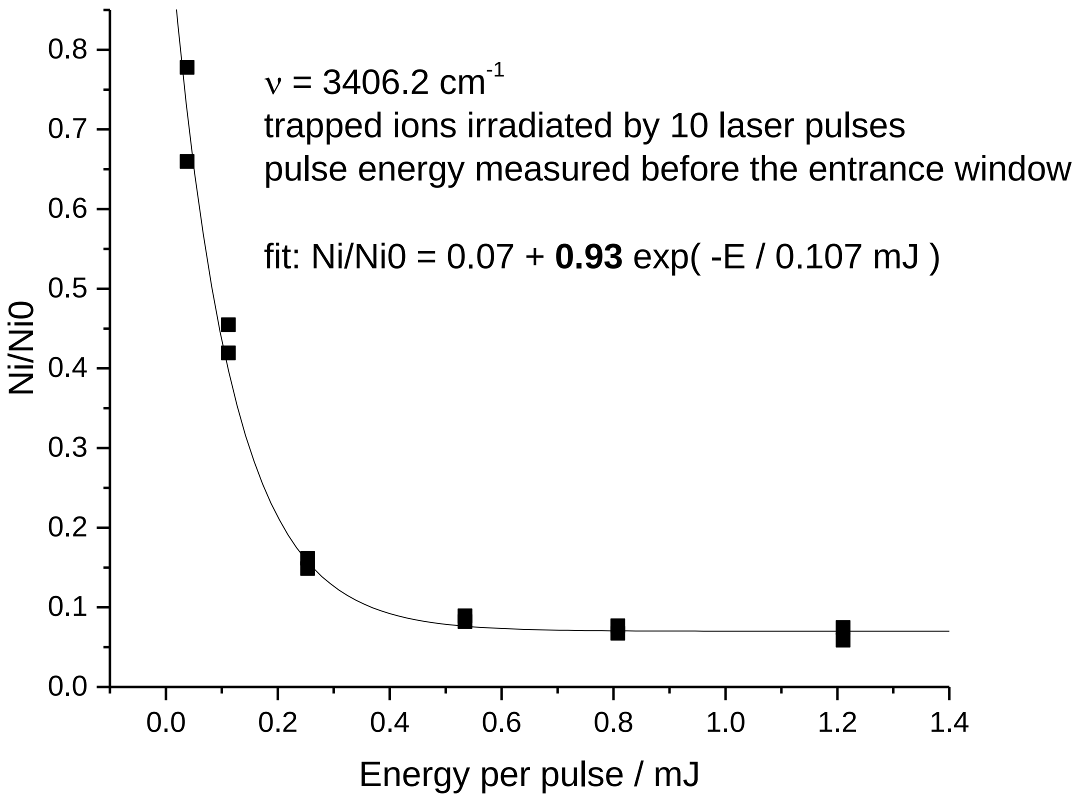

Figure S28. Dependence of the photofragmentation efficiency on the laser power measured at 3406.2 cm^{-1}.

Table S5. Geometries and energetics for calculated structures optimized at the B3LYP-D3/6-311++G**:SDD(Pd) level of theory.

**S1**	NH_I_4b.log<br><br>Low frequencies --- -14.1088 -10.1257 -9.8898 -0.0008 -0.0005 0.0003<br>Low frequencies --- 19.9905 20.4721 26.2084<br><br>Zero-point correction= 0.310976 (Hartree/Particle)<br>Thermal correction to Energy= 0.332707<br>Thermal correction to Enthalpy= 0.333651<br>Thermal correction to Gibbs Free Energy= 0.256217<br>Sum of electronic and zero-point Energies= -987.759647<br>Sum of electronic and thermal Energies= -987.737915<br>Sum of electronic and thermal Enthalpies= -987.736971<br>Sum of electronic and thermal Free Energies= -987.814405<br><br>N,0,0.000493331,0.0095527103,-0.002152097<br>N,0,-0.0018820893,0.012315993,1.3854927478<br>C,0,1.3039237981,0.0050703183,1.8121882693<br>C,0,1.2926774092,0.1628670681,-0.4463735403<br>C,0,2.1328026387,0.1438789397,0.7286784466<br>H,0,4.0609993174,-0.7435981333,0.440202168<br>C,0,3.6341432742,0.2279941104,0.7109953417<br>C,0,4.1475681089,1.2812089439,-0.2411506584<br>H,0,5.8005280954,0.1317711538,-1.0557845937<br>C,0,5.2765804999,1.0828914557,-1.0700559327<br>C,0,5.4519135178,1.9726726332,-2.1503757178<br>C,0,-1.2121681335,0.1837490493,-0.726323229<br>C,0,-2.2453362379,0.9433039762,-0.1765212804<br>C,0,-3.4353438604,1.0837838788,-0.8849585422<br>C,0,-3.5839691879,0.4867995778,-2.1346084862<br>C,0,-2.5389014359,-0.2603493482,-2.675907134<br>C,0,-1.3498072594,-0.4251114367,-1.9734665793<br>C,0,1.6096841745,-0.1636696831,3.2604331308<br>O,0,1.5640084466,0.320521568,-1.666239712<br>Pd,0,3.4850380868,1.0946679141,-2.2415606449<br>N,0,2.989627422,1.1448929043,-4.2825539388<br>C,0,2.6718359659,1.1771960021,-5.3855538274<br>H,0,1.671449461,-1.2239382584,3.5245884497<br>H,0,2.5671507939,0.2946571565,3.508236606<br>H,0,0.8391883211,0.2990270577,3.879840656<br>H,0,-0.5364273845,-1.008694937,-2.3805814234<br>H,0,-2.6542417166,-0.731547781,-3.644438096<br>H,0,-4.5104168224,0.6017332155,-2.6834767937<br>H,0,-4.2414106821,1.6704845997,-0.4618957522<br>H,0,-2.1137314541,1.4289320392,0.7820337765<br>H,0,-0.7069462619,-0.5737409041,1.8183849068<br>H,0,3.9898790895,0.453973986,1.7219724315<br>H,0,3.8411291858,2.3040187598,-0.0252303071<br>H,0,6.1691723195,1.7327036654,-2.925494608<br>H,0,5.2082316504,3.0251634698,-2.040685578<br>C,0,2.2705961691,1.21684926,-6.7786375648<br>H,0,2.9829378113,1.811199327,-7.3541478427<br>H,0,2.2411912687,0.2034077732,-7.1838117125<br>H,0,1.2788005286,1.665133884,-6.8646474285
**S2**	CH_1.log<br><br>Low frequencies --- -17.2177 -13.7374 -10.5445 -8.9099 -0.0006 -0.0004<br>Low frequencies --- 0.0006 17.1408 20.1232<br><br>Zero-point correction= 0.310400 (Hartree/Particle)<br>Thermal correction to Energy= 0.330946<br>Thermal correction to Enthalpy= 0.331890<br>Thermal correction to Gibbs Free Energy= 0.258226<br>Sum of electronic and zero-point Energies= -987.750547<br>Sum of electronic and thermal Energies= -987.730001<br>Sum of electronic and thermal Enthalpies= -987.729057<br>Sum of electronic and thermal Free Energies= -987.802721<br><br>C,0,0.0676816862,-0.5050810131,0.0900901208<br>C,0,0.0238233179,-0.0897983877,1.4232315701<br>C,0,1.1461865793,0.4720120308,2.0349555454<br>C,0,2.3204623906,0.6129711722,1.304074491<br>C,0,2.3801773938,0.2000579642,-0.025097889<br>C,0,1.2527521078,-0.3556373049,-0.6243616048<br>N,0,-1.1751850041,-0.2188798816,2.1878289223<br>N,0,-1.3249218394,0.589566987,3.3382420328<br>C,0,-2.4562380393,0.3174846734,3.8761779823

	C,0,-3.1902436134,-0.7739862167,3.141396228<br>C,0,-2.2378819804,-1.022747953,1.9815203918<br>C,0,-2.9367356091,1.0075201482,5.102864867<br>O,0,-2.3812700428,-1.81055547,1.0274659263<br>Pd,0,-4.3291439358,-2.6155144933,0.5105856277<br>N,0,-3.5707951158,-3.7546736176,-1.0811135388<br>C,0,-3.1092069228,-4.387244275,-1.9211950039<br>C,0,-2.5258929768,-5.185149075,-2.9819279522<br>C,0,-4.6483774523,-0.4819877892,2.7342785688<br>C,0,-5.3088052368,-1.7073266414,2.1626816038<br>C,0,-6.1847988881,-1.6998948451,1.0551160188<br>C,0,-6.4502445151,-2.9493594567,0.4493658093<br>H,0,-4.6806002091,0.3503675476,2.0271081166<br>H,0,-6.4024578698,-0.7743933186,0.5309183329<br>H,0,-3.8589107064,1.5614844596,4.9013938217<br>H,0,-3.1562076413,0.2844508727,5.8942271749<br>H,0,-2.1805351069,1.7061989961,5.4582582954<br>H,0,-0.8031968518,-0.9377794681,-0.3766497413<br>H,0,1.2895446496,-0.6722364237,-1.6597567604<br>H,0,3.2972168715,0.3136996616,-0.5898446509<br>H,0,3.190951321,1.0488103323,1.7786983201<br>H,0,1.0893755605,0.8004978491,3.0623615705<br>H,0,-5.1929497624,-0.1685230761,3.6315452535<br>H,0,-5.3614903846,-2.5617693219,2.8376321719<br>H,0,-6.9347951378,-2.9791177662,-0.5187289925<br>H,0,-6.5588674301,-3.8408695789,1.0597532307<br>H,0,-3.1752111546,-1.6785773871,3.765880544<br>H,0,-3.0411685807,-6.1453636337,-3.049506591<br>H,0,-2.6216220009,-4.6600700259,-3.9344918387<br>H,0,-1.4684418304,-5.3597304539,-2.7736038675
**S3**	NH_II_3.log<br><br>Low frequencies --- -17.5727 -10.8828 -9.6340 -0.0002 0.0006 0.0009<br>Low frequencies --- 12.1156 21.1026 23.5587<br><br>Zero-point correction= 0.310268 (Hartree/Particle)<br>Thermal correction to Energy= 0.332500<br>Thermal correction to Enthalpy= 0.333445<br>Thermal correction to Gibbs Free Energy= 0.253956<br>Sum of electronic and zero-point Energies= -987.733289<br>Sum of electronic and thermal Energies= -987.711057<br>Sum of electronic and thermal Enthalpies= -987.710113<br>Sum of electronic and thermal Free Energies= -987.789602<br><br>C,0,0.3926606672,-0.6834935465,0.3333009766<br>C,0,0.2367027415,0.049307055,1.509235611<br>C,0,1.3175700777,0.6962208204,2.108456922<br>C,0,2.5710680377,0.615112686,1.5086914122<br>C,0,2.7437295418,-0.1190085967,0.337583809<br>C,0,1.6560215941,-0.7702829589,-0.2418646756<br>N,0,-1.0563314391,0.1444044672,2.0988274781<br>N,0,-1.4348022429,1.2726276675,2.8124275692<br>C,0,-2.6740250588,1.0393990834,3.3597991976<br>C,0,-3.0261555953,-0.2651622139,3.119237229<br>C,0,-1.9846868013,-0.8336047656,2.3176592841<br>C,0,-3.3785883071,2.1177598004,4.1062263074<br>O,0,-1.8608085316,-2.013806464,1.8759488275<br>C,0,-4.2130553163,-1.0430828627,3.590002571<br>C,0,-4.6794851228,-2.1151182906,2.6063042772<br>C,0,-5.9359558366,-2.7745694949,2.9847485847<br>C,0,-6.9166501778,-3.0494679676,2.1187881694<br>H,0,-5.0561230436,-0.3546729111,3.7334151548<br>H,0,-6.0637377622,-3.0126046449,4.0380759832<br>H,0,-3.1994400631,3.0978364334,3.6582682716<br>H,0,-4.4531983169,1.9363285535,4.1144872559<br>H,0,-3.0336212739,2.1564704348,5.1435207343<br>H,0,-0.4574200178,-1.1799181604,-0.1128482103<br>H,0,1.7869636218,-1.3408227821,-1.1530345732<br>H,0,3.7225366337,-0.1851557875,-0.120883389<br>H,0,3.4147094046,1.1155371286,1.9676784332<br>H,0,1.1841493967,1.2370559213,3.0368587301<br>H,0,-1.1920759673,2.1575327405,2.3822238829<br>H,0,-4.0279035154,-1.4949585513,4.571329351<br>H,0,-7.8479955159,-3.4999120374,2.4405583875<br>H,0,-6.8191673003,-2.8223376715,1.0622432516<br>Pd,0,-3.147077917,-3.5021702932,2.4363700742<br>H,0,-4.7014351166,-1.751017433,1.5759781755<br>N,0,-4.171230129,-5.1710174439,2.9335465243<br>C,0,-4.6969352234,-6.1625778963,3.1801626853<br>C,0,-5.3763506752,-7.4053049345,3.4867655403<br>H,0,-5.079510667,-8.1755371269,2.7718586651

	H,0,-5.1164659686,-7.734307462,4.4949378542<br>H,0,-6.4568831362,-7.2588972028,3.4242256791
**S4**	OH_II_1b.log<br><br>Low frequencies --- -15.9934 -10.9540 -10.7181 -0.0003 -0.0001 0.0004<br>Low frequencies --- 8.6443 15.4251 27.0770<br><br>Zero-point correction= 0.310073 (Hartree/Particle)<br>Thermal correction to Energy= 0.332038<br>Thermal correction to Enthalpy= 0.332983<br>Thermal correction to Gibbs Free Energy= 0.254242<br>Sum of electronic and zero-point Energies= -987.729777<br>Sum of electronic and thermal Energies= -987.707812<br>Sum of electronic and thermal Enthalpies= -987.706868<br>Sum of electronic and thermal Free Energies= -987.785608<br><br>C,0,0.0003795304,-0.0011343324,-0.0009165337<br>N,0,-0.000273152,-0.002081015,1.3562183289<br>N,0,1.2809898746,-0.0008824474,1.8375370579<br>C,0,2.062264063,-0.0251893114,0.7662160557<br>C,0,1.3026705698,-0.0234028084,-0.443990961<br>C,0,-1.1275315953,0.127048635,2.2089122467<br>C,0,-2.2471273586,-0.6834241042,1.9990242178<br>C,0,-3.3740239576,-0.5153526417,2.8043433124<br>C,0,-3.3672141131,0.4295875766,3.8258452876<br>C,0,-2.2297452864,1.2079174605,4.0475505131<br>C,0,-1.1089479672,1.0689108132,3.2370310247<br>C,0,3.5483668513,-0.0634223585,0.9063124077<br>C,0,1.8030930586,-0.0653647191,-1.8610052553<br>C,0,1.1222374405,0.941148469,-2.7503309414<br>C,0,0.7175146839,0.6693993237,-4.0698407922<br>C,0,-0.1974385504,1.5712744011,-4.6643524004<br>Pd,0,-1.0386792724,0.8659972612,-2.8406234243<br>N,0,-3.1104001908,1.096906026,-3.1087656308<br>C,0,-4.2320980378,1.2497881165,-3.3071700199<br>C,0,-5.6460113371,1.442539311,-3.5631618392<br>O,0,-1.160277419,0.0678568394,-0.7341542366<br>H,0,1.7007938798,-1.0681727172,-2.2894543596<br>H,0,0.868458271,-0.3191445334,-4.4927134093<br>H,0,3.9635836186,-0.9854146185,0.4895558401<br>H,0,4.019590316,0.7779402672,0.3899371688<br>H,0,3.8168399329,-0.0129677432,1.961174156<br>H,0,-2.2117931314,-1.4840639744,1.2680821504<br>H,0,-4.2394348328,-1.1490031328,2.6529882356<br>H,0,-4.2367430365,0.5483272671,4.4603531281<br>H,0,-2.2181565236,1.9316894749,4.8533189328<br>H,0,-0.2195953577,1.6648112229,3.3928216801<br>H,0,2.8757896639,0.1600836765,-1.8533533813<br>H,0,1.214158062,1.9820481808,-2.4422576577<br>H,0,-0.6963387849,1.2943038156,-5.5852762894<br>H,0,-0.1131159127,2.6377868536,-4.4760162441<br>H,0,-6.1037308819,1.9811629477,-2.7309116797<br>H,0,-5.7789595815,2.0213107058,-4.4795528494<br>H,0,-6.1367714728,0.4737962643,-3.6773392171<br>H,0,-1.9250344971,0.1837756253,-0.1452017438
**S5**	NH_I_1.log<br><br>Low frequencies --- -13.3958 -11.5181 -8.2834 -0.0005 0.0002 0.0007<br>Low frequencies --- 10.5576 25.3881 29.7789<br><br>Zero-point correction= 0.309726 (Hartree/Particle)<br>Thermal correction to Energy= 0.332040<br>Thermal correction to Enthalpy= 0.332984<br>Thermal correction to Gibbs Free Energy= 0.253919<br>Sum of electronic and zero-point Energies= -987.726711<br>Sum of electronic and thermal Energies= -987.704397<br>Sum of electronic and thermal Enthalpies= -987.703453<br>Sum of electronic and thermal Free Energies= -987.782518<br><br>C,0,0.004024121,0.0567864155,-0.0122823566<br>C,0,0.0137114864,0.006192799,1.3833981005<br>C,0,1.2185360931,-0.0199499791,2.0900679311<br>C,0,2.4158916923,0.0061467663,1.3832555247<br>C,0,2.4202017208,0.0380105915,-0.0105040466<br>C,0,1.212882563,0.0620171535,-0.7033621398<br>N,0,-1.2061559282,-0.0371658148,2.1063182825<br>C,0,-1.5334576248,0.6141590884,3.3085448224<br>C,0,-2.9923311925,0.4368230443,3.4369040271<br>C,0,-3.438563341,-0.2308094438,2.3070511349<br>N,0,-2.3808025297,-0.4289469416,1.4706866375<br>O,0,-0.7617348871,1.195024293,4.047960942

	C,0,-4.8125317666,-0.6252613954,1.8863252233<br>Pd,0,-2.949668469,-1.5934116203,4.838230805<br>N,0,-2.1636496766,-3.3640025887,4.0446725747<br>C,0,-1.6176106541,-4.3107416724,3.6874782194<br>C,0,-0.9262083449,-5.5071537204,3.2483882182<br>C,0,-3.7721393449,1.1179599575,4.5458869137<br>C,0,-3.436389977,0.2573455325,5.7417958312<br>C,0,-4.2872217944,-0.7321271927,6.2880272811<br>C,0,-3.6723080032,-1.8261461662,6.9259542221<br>H,0,-4.258249508,-2.6966016902,7.1937736248<br>H,0,-5.3326850319,-0.7827465206,5.9998184628<br>H,0,-2.4978817176,0.4859719794,6.242184447<br>H,0,-3.4319025072,2.1469077708,4.6731379481<br>H,0,-4.8420193576,1.1222697891,4.3324463699<br>H,0,-2.7342807834,-1.696759675,7.4562358684<br>H,0,-4.8154404164,-1.6043922274,1.4017194163<br>H,0,-5.4820758117,-0.6681659094,2.7440079366<br>H,0,-5.2125395876,0.1022448268,1.1742312902<br>H,0,1.2098425696,-0.0338001042,3.1701784059<br>H,0,3.3518541915,-0.0009340241,1.9284625641<br>H,0,3.3579285174,0.0535907625,-0.5517353135<br>H,0,1.2061327624,0.1042812787,-1.7856660483<br>H,0,-0.9308258665,0.1243999229,-0.5544508277<br>H,0,-1.6067406833,-6.1417359828,2.6770396335<br>H,0,-0.0755297766,-5.2346799731,2.6202680109<br>H,0,-0.5653232907,-6.062621027,4.1166264398<br>H,0,-2.3090643459,-1.2441408571,0.8742176155
**S6**	NH_V_1b.log<br><br>Low frequencies --- -16.7140 -12.9380 -10.9001 -0.0003 0.0004 0.0008<br>Low frequencies --- 9.4622 12.9075 17.2238<br><br>Zero-point correction= 0.309489 (Hartree/Particle)<br>Thermal correction to Energy= 0.332103<br>Thermal correction to Enthalpy= 0.333048<br>Thermal correction to Gibbs Free Energy= 0.251728<br>Sum of electronic and zero-point Energies= -987.725063<br>Sum of electronic and thermal Energies= -987.702448<br>Sum of electronic and thermal Enthalpies= -987.701504<br>Sum of electronic and thermal Free Energies= -987.782824<br><br>C,0,-1.3811204065,0.2322540313,3.3593745914<br>C,0,-1.1811056813,-0.3331416522,2.0996093716<br>C,0,-2.1147000278,-1.2126666306,1.5484713274<br>C,0,-3.271959962,-1.5108680987,2.2626500311<br>C,0,-3.4817181485,-0.9564853731,3.5227166168<br>C,0,-2.5315152236,-0.095159969,4.0688845347<br>N,0,-0.004376855,0.0032703374,1.3769384821<br>N,0,0.0027103868,-0.0339793775,-0.0104314055<br>C,0,1.2389386507,0.1520107002,-0.496798967<br>C,0,2.1333489589,0.1942201811,0.6013657752<br>C,0,1.3153914363,0.079045257,1.84128911<br>O,0,1.6567196104,0.0430869345,3.0048452728<br>C,0,3.542788862,0.3460396082,0.5236280485<br>C,0,4.494852733,0.346296362,1.6994563977<br>C,0,5.0252155685,1.764896064,1.5757192865<br>C,0,4.4730206574,2.8254231196,2.2180069248<br>Pd,0,2.8391912469,2.334135468,0.4397796652<br>N,0,1.8772236205,4.2003664177,0.288693808<br>C,0,1.4185030821,5.2532460353,0.2258530466<br>C,0,0.842420447,6.5826492429,0.1497394562<br>C,0,1.495274313,0.1355774349,-1.9647504222<br>H,0,4.0059768977,0.1635586243,-0.4438944058<br>H,0,5.8390116853,1.9320473805,0.8751040768<br>H,0,0.7366048448,0.7068405048,-2.5037416299<br>H,0,2.4685818261,0.564486167,-2.1945610068<br>H,0,1.4732623745,-0.8931399554,-2.3372884311<br>H,0,-0.6389752348,0.8974334746,3.7765275646<br>H,0,-2.6877328142,0.3321540308,5.0517177789<br>H,0,-4.3779164224,-1.1998299043,4.0795888025<br>H,0,-3.9989255428,-2.192614337,1.8388532677<br>H,0,-1.9314729034,-1.6849516809,0.5907623614<br>H,0,-0.8607771393,0.1834594432,-0.49016171<br>H,0,4.8802545044,3.8212917473,2.0898049838<br>H,0,3.7380711791,2.6824744508,3.0040920541<br>H,0,3.9682499667,0.197725404,2.6399328819<br>H,0,5.280904718,-0.4025705552,1.5957794678<br>H,0,0.5790207787,6.8153350552,-0.8840099995<br>H,0,-0.0567191182,6.6337889256,0.767136893<br>H,0,1.5628381309,7.320833132,0.5080390983
**S7**	NH_R_3.log

	Low frequencies --- -14.4102 -10.4852 -5.9714 0.0001 0.0005 0.0007<br>Low frequencies --- 9.8769 19.4113 20.6161<br><br>Zero-point correction= 0.312520 (Hartree/Particle)<br>Thermal correction to Energy= 0.334340<br>Thermal correction to Enthalpy= 0.335284<br>Thermal correction to Gibbs Free Energy= 0.255499<br>Sum of electronic and zero-point Energies= -987.719532<br>Sum of electronic and thermal Energies= -987.697712<br>Sum of electronic and thermal Enthalpies= -987.696768<br>Sum of electronic and thermal Free Energies= -987.776553<br><br>C,0,-0.029091034,-0.3391544733,0.1269482144<br>C,0,0.0232386849,-0.3563960911,1.6409177051<br>C,0,1.2096653562,-0.1573175254,2.3521655312<br>Pd,0,-0.0098323892,1.5643793009,2.5679502811<br>N,0,-1.0350294446,3.3147340422,3.052755724<br>C,0,-1.5786194798,4.2223227215,3.5064184742<br>C,0,-2.2710854899,5.3578311773,4.0862930922<br>C,0,2.5536629628,-0.0144756909,1.689942043<br>O,0,2.7414942435,1.1859063912,0.8603545207<br>C,0,1.8689220236,1.4547855133,-0.0748726508<br>N,0,2.0879896184,2.5511501121,-0.840112951<br>N,0,1.0086854721,2.6982579283,-1.6782403767<br>C,0,0.1758022264,1.6351010143,-1.5163451748<br>C,0,0.6614465234,0.8440254582,-0.4882897513<br>C,0,3.0805501537,3.5739331579,-0.7180392451<br>C,0,4.4267944588,3.2216482693,-0.7581538851<br>C,0,5.3844523798,4.2240747197,-0.6424552805<br>C,0,4.995521273,5.5561842374,-0.5101663135<br>C,0,3.6434750118,5.8926555307,-0.4800670918<br>C,0,2.6735899568,4.8989137664,-0.5729945513<br>C,0,-1.0576445452,1.486957918,-2.3362573878<br>H,0,3.344247392,0.0861993964,2.4277338899<br>H,0,1.2768614809,-0.5564918054,3.3610632809<br>H,0,-0.78287293,-0.9037838243,2.1198353155<br>H,0,-1.0715021155,-0.3315239427,-0.1957939111<br>H,0,0.3996564677,-1.2747542236,-0.261422192<br>H,0,2.7818790824,-0.8683723481,1.0429476632<br>H,0,-1.1173336033,2.2536399018,-3.1099124857<br>H,0,-1.0873355497,0.507342703,-2.8167674568<br>H,0,-1.9440154718,1.5762660346,-1.7023666444<br>H,0,4.7149963094,2.1855635853,-0.8748831265<br>H,0,6.4352832118,3.9642675588,-0.6680255306<br>H,0,5.7466539266,6.3321528587,-0.4298528442<br>H,0,3.3418603243,6.9268666374,-0.3704783644<br>H,0,1.6195443208,5.1417328571,-0.5190758778<br>H,0,-2.885939168,5.8490819012,3.3294934732<br>H,0,-2.9128597176,5.0228631729,4.9037837202<br>H,0,-1.5459617124,6.0751463954,4.4760827965<br>H,0,1.1487523561,3.2265965338,-2.5294115787
**S8**	NH_III_1.log<br><br>Low frequencies --- -14.2351 -11.1780 -9.6161 -0.0002 0.0003 0.0006<br>Low frequencies --- 10.0637 16.2868 21.8605<br><br>Zero-point correction= 0.308843 (Hartree/Particle)<br>Thermal correction to Energy= 0.332021<br>Thermal correction to Enthalpy= 0.332965<br>Thermal correction to Gibbs Free Energy= 0.249898<br>Sum of electronic and zero-point Energies= -987.718000<br>Sum of electronic and thermal Energies= -987.694822<br>Sum of electronic and thermal Enthalpies= -987.693878<br>Sum of electronic and thermal Free Energies= -987.776945<br><br>C,0,-0.7435246157,0.1511412752,0.1844773808<br>N,0,-0.4714518096,-0.1031801351,1.5449564309<br>N,0,0.8616521969,0.1652181382,1.7924503001<br>C,0,1.5097900412,0.5624326494,0.7100679033<br>C,0,0.5604026929,0.637227977,-0.3570722816<br>C,0,-1.379293076,-0.3301190036,2.6159948562<br>C,0,-2.4151987348,-1.2456260548,2.4363705516<br>C,0,-3.3060162768,-1.4646120623,3.4820436292<br>C,0,-3.151924604,-0.7976907162,4.6962980286<br>C,0,-2.1106847741,0.1121933443,4.8634980317<br>C,0,-1.2263369342,0.3629431336,3.8182157567<br>C,0,2.9882090393,0.7265506271,0.6943955402<br>C,0,0.8550290884,0.9903865318,-1.7080841749<br>C,0,-0.0642470129,0.645009493,-2.8537441648<br>O,0,-1.8191438707,-0.0035139829,-0.3430708298

	C,0,0.2192468005,-0.7736274823,-3.3010142115<br>C,0,-0.61625603,-1.7884180832,-3.097484812<br>H,0,1.9107096861,1.0362148583,-1.971551846<br>H,0,1.1696546558,-0.9412371786,-3.8024424158<br>H,0,3.3766321917,0.9210423332,1.6953248827<br>H,0,3.4543263282,-0.1883435225,0.3150631459<br>H,0,3.2767422872,1.5504233793,0.043531291<br>H,0,-2.5215640268,-1.7628972879,1.4935331416<br>H,0,-4.1172090192,-2.169543908,3.3489719905<br>H,0,-3.8437211495,-0.9830854593,5.5084251439<br>H,0,-1.9949686807,0.6430120521,5.8003063919<br>H,0,-0.4479696057,1.1089399849,3.9335394363<br>H,0,1.2472497195,-0.0620904914,2.6999982065<br>H,0,-0.3773634679,-2.7890123273,-3.4388398381<br>H,0,-1.5680291926,-1.6456692378,-2.5972513501<br>H,0,-1.1055106487,0.7397023542,-2.5522610936<br>H,0,0.1307334662,1.3416527693,-3.6745333521<br>Pd,0,0.437976657,2.7647279843,-0.705006557<br>N,0,0.3726872844,4.5859172835,-1.6864818591<br>C,0,0.2817016961,5.5894325825,-2.2409577842<br>C,0,0.1652008791,6.8517131327,-2.944076325<br>H,0,-0.3464105627,7.5806322062,-2.3121115042<br>H,0,1.1577602199,7.2303928006,-3.1962300873<br>H,0,-0.4092011672,6.7108110484,-3.8620895495
**S09**	NH_I_4b2a.log<br><br>Low frequencies --- -13.9594 -11.1037 -9.9765 -0.0007 -0.0003 0.0006<br>Low frequencies --- 13.8903 19.3672 24.1823<br><br>Zero-point correction= 0.310630 (Hartree/Particle)<br>Thermal correction to Energy= 0.332190<br>Thermal correction to Enthalpy= 0.333134<br>Thermal correction to Gibbs Free Energy= 0.255627<br>Sum of electronic and zero-point Energies= -987.717916<br>Sum of electronic and thermal Energies= -987.696357<br>Sum of electronic and thermal Enthalpies= -987.695413<br>Sum of electronic and thermal Free Energies= -987.772920<br><br>C,0,-0.0112439935,-0.0254778016,-0.0077190121<br>C,0,-0.007945627,-0.0180110502,1.3778266793<br>C,0,1.1649080477,-0.0141443642,2.1188679974<br>C,0,2.3770837826,-0.0159113311,1.4372964355<br>C,0,2.4006131394,-0.0210676858,0.0421973923<br>C,0,1.2101839418,-0.0248146451,-0.6790618249<br>N,0,-1.2975996064,-0.0600864626,2.0862321491<br>C,0,-1.6454763116,-1.4611232253,2.6314791391<br>C,0,-1.8538791301,-1.2606941053,3.9721724516<br>C,0,-1.6978869151,0.1474081723,4.2323233185<br>N,0,-1.3794981376,0.8910287181,3.2055183402<br>O,0,-1.666502152,-2.3962505348,1.8033561862<br>Pd,0,-1.9441120151,-4.3812030163,2.6465322657<br>N,0,-2.0364176547,-5.3286102833,0.7766049303<br>C,0,-2.0732881559,-5.8194655759,-0.2609910666<br>C,0,-2.2758126582,-2.3145312255,4.9637166895<br>C,0,-1.7144299719,-3.6794408251,4.6463815412<br>C,0,-2.4988413632,-4.8536430944,4.6675260337<br>C,0,-1.9692274964,-6.0066445754,4.0494936038<br>C,0,-1.8759071796,0.7964120449,5.5622311776<br>H,0,-3.3690990483,-2.3729708733,5.0179583114<br>H,0,-3.5619483979,-4.7889895869,4.8794577866<br>H,0,-2.8877420791,0.6212959701,5.9378034829<br>H,0,-1.1779047665,0.370802254,6.2886328884<br>H,0,-1.7027158327,1.8687580976,5.4870644169<br>H,0,-0.9440535515,-0.0308293337,-0.5602707077<br>H,0,1.2264317774,-0.0201982074,-1.7616245166<br>H,0,3.3487194264,-0.0142237594,-0.4811039388<br>H,0,3.3043047867,-0.0025714027,1.996347854<br>H,0,1.1302035911,0.0127702007,3.1996652494<br>H,0,-1.9323263221,-2.0234543779,5.9628267252<br>H,0,-0.6336681924,-3.7794155332,4.7379373167<br>H,0,-2.6181057167,-6.8522495146,3.8573373466<br>H,0,-0.9093531484,-6.2319315771,4.1239044841<br>H,0,-2.0335008618,0.20750226,1.4273432815<br>C,0,-2.1220916504,-6.4403246473,-1.5706841442<br>H,0,-1.7760258814,-5.7346506467,-2.3283694766<br>H,0,-1.4818004645,-7.3245264847,-1.5860723676<br>H,0,-3.1471696327,-6.7377238516,-1.8008129471
**S10**	NH_IV_3b.log<br><br>Low frequencies --- -16.8193 -10.1261 -8.9400 0.0004 0.0005 0.0006<br>Low frequencies --- 8.4783 9.8894 22.8303

	Zero-point correction= 0.309735 (Hartree/Particle)<br>Thermal correction to Energy= 0.332089<br>Thermal correction to Enthalpy= 0.333033<br>Thermal correction to Gibbs Free Energy= 0.251915<br>Sum of electronic and zero-point Energies= -987.715189<br>Sum of electronic and thermal Energies= -987.692835<br>Sum of electronic and thermal Enthalpies= -987.691890<br>Sum of electronic and thermal Free Energies= -987.773009<br><br>N,0,-2.0580800882,1.0107805386,2.1636887768<br>N,0,-0.9783747457,0.14726344,2.3283707783<br>C,0,-1.3353509146,-0.7825044836,3.2572059919<br>C,0,-2.6220245094,-0.4448043735,3.751088318<br>C,0,-3.0277083126,0.687407041,3.0902276492<br>C,0,0.1287488117,0.1690630785,1.4336911029<br>C,0,-0.0494866727,0.6028110851,0.120053184<br>C,0,1.0486555169,0.636427262,-0.7344993745<br>C,0,2.3009893605,0.2223843613,-0.2869117907<br>C,0,2.4587837312,-0.2221172792,1.0246923083<br>C,0,1.3771276823,-0.243421604,1.8987521348<br>O,0,-0.6677821564,-1.8093779075,3.6034417325<br>Pd,0,-1.8019983262,-2.8000697645,5.0332010465<br>N,0,-2.8327638281,-3.7770304809,6.4509698741<br>C,0,-3.3503552165,-4.385041201,7.2775448788<br>C,0,-4.0007266177,-5.154582372,8.3191153904<br>C,0,-3.2530558304,-1.3673012386,4.7573991593<br>C,0,-4.5598839975,-2.0124892025,4.3138265697<br>C,0,-4.4462635624,-2.8378729176,3.0627340869<br>C,0,-4.8434099663,-4.1021272179,2.9630317571<br>C,0,-4.2648594096,1.5082919713,3.2001911994<br>H,0,-3.3379409076,-0.9166277055,5.7492773381<br>H,0,-4.0084724616,-2.3435121497,2.19896555<br>H,0,-4.0486514867,2.5742766561,3.0964818676<br>H,0,-4.9791706698,1.2356035035,2.4179690301<br>H,0,-4.7394538213,1.3498138057,4.1681083077<br>H,0,1.4920833782,-0.5812355312,2.9191643361<br>H,0,3.4309828236,-0.5453512332,1.375564889<br>H,0,3.1502816885,0.2432008096,-0.9583218668<br>H,0,0.9186258721,0.9730758979,-1.7555486223<br>H,0,-1.0313639432,0.8888290607,-0.2353174926<br>H,0,-1.8126417999,1.9717371593,1.9544306299<br>H,0,-4.7455182961,-4.6569927497,2.0379218998<br>H,0,-5.2877617491,-4.6265226327,3.8034992307<br>H,0,-4.9780617865,-2.6083776569,5.1269140622<br>H,0,-5.2707600748,-1.1875425101,4.1399175035<br>H,0,-4.0309498924,-6.2086655773,8.0352570181<br>H,0,-3.4483019624,-5.0508448709,9.2553980069<br>H,0,-5.0212138594,-4.7938260104,8.4641755382
**S11**	OH_R_5.log<br><br>Low frequencies --- -18.0107 -7.8069 -7.1069 -0.0009 -0.0005 0.0005<br>Low frequencies --- 13.5985 14.9721 17.3349<br><br>Zero-point correction= 0.309367 (Hartree/Particle)<br>Thermal correction to Energy= 0.331953<br>Thermal correction to Enthalpy= 0.332898<br>Thermal correction to Gibbs Free Energy= 0.251340<br>Sum of electronic and zero-point Energies= -987.713432<br>Sum of electronic and thermal Energies= -987.690846<br>Sum of electronic and thermal Enthalpies= -987.689902<br>Sum of electronic and thermal Free Energies= -987.771459<br>C,0,-0.0642591739,0.1064756948,-0.3081049607<br>C,0,0.1032893441,0.0600195534,1.1837506563<br>Pd,0,2.1024979062,0.4161555283,2.1099538544<br>N,0,3.8167143424,1.1204933206,3.080702803<br>C,0,4.7631461351,1.5121067209,3.6030859434<br>C,0,5.9558504721,2.003621325,4.2623350352<br>C,0,0.5708759126,-1.0767153922,1.8609975444<br>C,0,0.8904435337,-0.9305522196,3.231534079<br>C,0,-1.4957815809,-0.2593361782,-0.5697696672<br>C,0,-2.0402335701,-1.5293723981,-0.4723845495<br>N,0,-3.3609952463,-1.4362474446,-0.7708223871<br>N,0,-3.7061712051,-0.1416264213,-1.0826660155<br>C,0,-2.5975611093,0.5670553426,-0.9417582156<br>C,0,-4.2914218842,-2.5014326336,-0.9062289491<br>C,0,-5.1100982502,-2.5558942424,-2.0336488078<br>C,0,-6.0055752049,-3.6103001491,-2.1703798621<br>C,0,-6.073753594,-4.6120433108,-1.2014104314<br>C,0,-5.2583766527,-4.5456814011,-0.075270525<br>C,0,-4.3717420329,-3.4818829613,0.0854070798

	O,0,-1.3933231745,-2.671620111,-0.1709693973<br>C,0,-2.5948292625,2.0408255854,-1.1827206989<br>H,0,0.3169390247,-0.2573632647,3.8618537476<br>H,0,0.8806105712,-1.9534637051,1.3029723833<br>H,0,-0.4425268929,0.8123944359,1.7499187487<br>H,0,0.1383544343,1.1104644683,-0.6864714879<br>H,0,0.6234081397,-0.5871284466,-0.7980851953<br>H,0,1.4379672428,-1.7117069293,3.7456859629<br>H,0,-3.5843425044,2.3594925487,-1.5092903349<br>H,0,-2.3393078131,2.5963275359,-0.2751582565<br>H,0,-1.8714775929,2.3149446077,-1.9562725733<br>H,0,-3.7906358156,-3.3875157567,0.9955868117<br>H,0,-5.3270829811,-5.3058955065,0.693203788<br>H,0,-6.7690541935,-5.4338976465,-1.3193755988<br>H,0,-6.6482295337,-3.6544816456,-3.0411707706<br>H,0,-5.0415254398,-1.7720738274,-2.7759414569<br>H,0,-1.9596661758,-3.4366613718,-0.3526190055<br>H,0,6.4878389304,2.6886923295,3.5987773629<br>H,0,5.6815144876,2.5310685161,5.1782514022<br>H,0,6.6106039469,1.1658311595,4.511442302
**S12**	NH_R7_2.log<br><br>Low frequencies --- -18.1590 -12.0391 -11.7886 -0.0009 -0.0005 0.0004<br>Low frequencies --- 11.6945 16.8524 19.7557<br><br>Zero-point correction= 0.312528 (Hartree/Particle)<br>Thermal correction to Energy= 0.334181<br>Thermal correction to Enthalpy= 0.335125<br>Thermal correction to Gibbs Free Energy= 0.256973<br>Sum of electronic and zero-point Energies= -987.707015<br>Sum of electronic and thermal Energies= -987.685362<br>Sum of electronic and thermal Enthalpies= -987.684418<br>Sum of electronic and thermal Free Energies= -987.762570<br><br>C,0,1.0213619634,1.2631845754,1.1980009322<br>C,0,0.2233748126,0.173875423,1.5419432827<br>C,0,0.6863229995,-1.1367218322,1.4287391876<br>C,0,1.9767043129,-1.3546523733,0.9569907691<br>C,0,2.7921737386,-0.2754768166,0.6199273991<br>C,0,2.3154442027,1.0283400611,0.7445569133<br>N,0,-1.1092178154,0.3944684748,1.998182297<br>C,0,-1.5835287566,1.2975623768,2.8671174379<br>C,0,-3.0219942089,1.2106714614,2.9188067487<br>C,0,-3.3648509164,0.2275359339,1.9421429268<br>N,0,-2.1664328993,-0.3607032035,1.4880643109<br>O,0,-0.7318507744,2.0251512452,3.5482386934<br>C,0,-1.1725090361,2.6341322166,4.8222873534<br>C,0,-2.3236281699,3.5822218664,4.6788236688<br>C,0,-4.6181776048,-0.5824922596,1.8192000438<br>C,0,-3.8568387131,1.7119488598,4.0770271974<br>C,0,-3.5505723809,3.1622816126,4.3707357053<br>H,0,-0.2707498428,3.1296452978,5.1686429912<br>H,0,-2.1272027302,4.6351253813,4.8424828301<br>H,0,-4.3605463871,3.8777478376,4.2843700049<br>H,0,-4.9112998021,1.5858759324,3.8375557105<br>H,0,-3.6659186193,1.0968026429,4.9673819622<br>H,0,-1.3993253503,1.8055271496,5.4977938247<br>H,0,-4.7604484931,-0.9203906568,0.7907539722<br>H,0,-4.5650426868,-1.4646655556,2.4655015704<br>H,0,-5.4875054739,0.0092439028,2.0998743702<br>H,0,0.0504489899,-1.9644938816,1.7165354539<br>H,0,2.348230906,-2.367628609,0.8654969248<br>H,0,3.7986014539,-0.4506588347,0.2605256723<br>H,0,2.9475481816,1.8662522987,0.4781420535<br>H,0,0.6358016393,2.2700038516,1.2846061879<br>H,0,-2.0675731599,-0.5368015765,0.4922395653<br>Pd,0,-3.5546033657,2.2311730247,1.111835156<br>N,0,-4.1577902952,3.2714092819,-0.5743753938<br>C,0,-4.5157206191,3.8920922447,-1.4746726876<br>C,0,-4.9687251094,4.670937509,-2.6113863223<br>H,0,-4.8662658432,5.73624883,-2.3946569539<br>H,0,-4.3707137035,4.427849902,-3.491987871<br>H,0,-6.0175659715,4.4497538333,-2.8194651443
**S13**	NH_III_3.log<br><br>Low frequencies --- -17.0903 -14.2031 -12.4505 -0.0008 -0.0006 -0.0004<br>Low frequencies --- 11.0519 16.9022 17.0593<br><br>Zero-point correction= 0.308576 (Hartree/Particle)<br>Thermal correction to Energy= 0.331177<br>Thermal correction to Enthalpy= 0.332121

	Thermal correction to Gibbs Free Energy= 0.251494<br>Sum of electronic and zero-point Energies= -987.703075<br>Sum of electronic and thermal Energies= -987.680474<br>Sum of electronic and thermal Enthalpies= -987.679530<br>Sum of electronic and thermal Free Energies= -987.760157<br><br>C,0,0.4081051825,-0.7458273559,0.1830586802<br>N,0,-0.288480926,-0.2249867286,1.2646717974<br>N,0,0.2910163946,0.974436575,1.6984459573<br>C,0,1.3816444104,1.3179756282,0.9360098084<br>C,0,1.580050269,0.2070323922,-0.0847003149<br>C,0,-1.6306824385,-0.5200724297,1.6399155362<br>C,0,-2.0625974529,-1.847209679,1.6382420044<br>C,0,-3.3776754243,-2.1270105146,1.9955636<br>C,0,-4.2471581586,-1.1034737175,2.367569891<br>C,0,-3.8000965394,0.2155634427,2.373885981<br>C,0,-2.4922036635,0.5164734843,2.0047883268<br>C,0,2.5392541293,1.9601745929,1.6503950007<br>C,0,1.4914060805,0.8812865838,-1.4527445761<br>C,0,0.2942782125,1.0468077408,-2.1171148118<br>O,0,0.1006752598,-1.7172448554,-0.4597802204<br>C,0,0.1835036368,1.6138755015,-3.4505516422<br>C,0,-0.9783488933,1.6730525232,-4.1139312575<br>H,0,2.4256850729,1.0968210473,-1.9609578804<br>H,0,1.0952989119,1.9826786595,-3.9117370644<br>H,0,2.2070312741,2.7846074269,2.2838078241<br>H,0,3.0346299716,1.2120125014,2.2816922115<br>H,0,3.2728274966,2.3437764681,0.9417398179<br>H,0,-1.385520462,-2.6385518217,1.3520309745<br>H,0,-3.7190479594,-3.1547922444,1.9911051173<br>H,0,-5.2662213121,-1.3329864138,2.6528062966<br>H,0,-4.4714781268,1.0165044826,2.658608121<br>H,0,-2.1492300007,1.5431115664,1.9852912079<br>H,0,0.2916780563,1.1130218112,2.7027001649<br>H,0,-1.0376787146,2.0826990906,-5.1143285838<br>H,0,-1.8998655403,1.3046140109,-3.6756554946<br>H,0,-0.6121243192,0.6140919699,-1.7032329845<br>H,0,2.5324452829,-0.3149877104,0.0423393814<br>Pd,0,0.7669049511,2.7368134542,-0.5620617059<br>N,0,0.4023843778,4.4989969421,0.5152057552<br>C,0,0.1966295299,5.5249088112,0.9928264867<br>C,0,-0.0647272473,6.8207282612,1.5883529832<br>H,0,-0.3184015303,7.5393896572,0.8061573658<br>H,0,-0.8982293874,6.7473468704,2.2898494794<br>H,0,0.8224962639,7.1712168479,2.1197582541
**S14**	MH_R_5.log<br><br>Low frequencies --- -20.4646 -16.0252 -11.7041 -0.0005 0.0003 0.0008<br>Low frequencies --- 5.1916 23.0888 37.2593<br><br>Zero-point correction= 0.309541 (Hartree/Particle)<br>Thermal correction to Energy= 0.330475<br>Thermal correction to Enthalpy= 0.331419<br>Thermal correction to Gibbs Free Energy= 0.256550<br>Sum of electronic and zero-point Energies= -987.698548<br>Sum of electronic and thermal Energies= -987.677614<br>Sum of electronic and thermal Enthalpies= -987.676670<br>Sum of electronic and thermal Free Energies= -987.751539<br><br>C,0,0.0761754098,-0.7082572671,-0.1015436929<br>C,0,0.1781789059,-1.0166319797,1.3778966745<br>C,0,1.3496394326,-0.7366015509,2.0638185346<br>Pd,0,0.0618626272,1.0401799895,2.1185950314<br>N,0,-0.3766494585,3.0326312724,2.3406414076<br>C,0,-0.4221613926,4.1797913349,2.3247651781<br>C,0,-0.466867796,5.6256349548,2.3049646295<br>C,0,2.655533548,-0.3944069973,1.3551535689<br>O,0,2.7422850881,0.9102131192,0.7331656455<br>C,0,1.6781524979,1.3650962799,0.0619324001<br>N,0,1.6350308016,2.6457415196,-0.393046513<br>N,0,0.4444500855,2.8879797567,-1.0412354802<br>C,0,-0.2504264865,1.7721960306,-0.9942220275<br>C,0,0.4592102582,0.7482866907,-0.2834877738<br>C,0,2.5184500952,3.7403041626,-0.1404331069<br>C,0,3.8442543366,3.5361442384,0.244842227<br>C,0,4.6573371713,4.6391903119,0.4950556696<br>C,0,4.1678440836,5.9337829318,0.3498879341<br>C,0,2.8492324369,6.1253963999,-0.0594399719<br>C,0,2.0196412355,5.0361758501,-0.3023726434<br>C,0,-1.5948975471,1.6641090046,-1.6305744707<br>H,0,3.47751113,-0.3943517453,2.0654674446

	H,0,1.4620795195,-1.1083750782,3.0763487049<br>H,0,-0.6069023823,-1.5833522668,1.8664687482<br>H,0,-0.9473977451,-0.8524956841,-0.4456656038<br>H,0,0.7058677373,-1.3855570444,-0.6910532391<br>H,0,2.8559431693,-1.1498585538,0.58872764<br>H,0,-1.8863545453,2.6306809521,-2.0399758935<br>H,0,-1.5871220528,0.9351240847,-2.4456469264<br>H,0,-2.3515856894,1.3466631746,-0.907819021<br>H,0,4.2441707933,2.5394362381,0.3409677119<br>H,0,5.6862924998,4.4779101755,0.7923367695<br>H,0,4.8111483847,6.7845579468,0.536706501<br>H,0,2.4658038713,7.1285847457,-0.2046834713<br>H,0,1.0047000135,5.1713239263,-0.6470058978<br>H,0,-1.1791678489,5.9598261541,1.5477290338<br>H,0,-0.773296445,6.0022456274,3.2828781489<br>H,0,0.5262617606,6.0069154917,2.0564828618<br>H,0,-0.2174531162,0.772767664,3.5943361273
**S15**	NH_R7_4.log<br><br>Low frequencies --- -14.2014 -11.4664 -9.6103 -0.0004 0.0005 0.0007<br>Low frequencies --- 18.7273 24.6718 25.4302<br><br>Zero-point correction= 0.312809 (Hartree/Particle)<br>Thermal correction to Energy= 0.334302<br>Thermal correction to Enthalpy= 0.335247<br>Thermal correction to Gibbs Free Energy= 0.257913<br>Sum of electronic and zero-point Energies= -987.696220<br>Sum of electronic and thermal Energies= -987.674727<br>Sum of electronic and thermal Enthalpies= -987.673782<br>Sum of electronic and thermal Free Energies= -987.751117<br><br>C,0,-0.0979853166,1.1520107089,0.4251975169<br>C,0,-0.1346215378,0.1352486008,1.3779181828<br>C,0,0.9676781704,-0.6746919927,1.6224736393<br>C,0,2.1352415285,-0.4645057138,0.891425761<br>C,0,2.1838469956,0.5436989635,-0.0684885135<br>C,0,1.0698913222,1.3513161863,-0.3021436112<br>N,0,-1.3386853761,-0.0897103585,2.1364328019<br>C,0,-1.8394033871,0.7914009294,3.0739286933<br>C,0,-3.2182601279,0.7071958363,3.1533138054<br>C,0,-3.625109845,-0.2272097542,2.1563544433<br>N,0,-2.4592406311,-0.621077039,1.4606447761<br>O,0,-0.9502571917,1.5396168724,3.6908472337<br>C,0,-1.4161964848,2.3162449474,4.8492813155<br>C,0,-2.4791940429,3.3140741289,4.5004701769<br>C,0,-4.8129367418,-1.1408857796,2.244958353<br>C,0,-4.1448953675,1.5031141453,4.0336014081<br>C,0,-3.7125280928,2.9484624993,4.1506824376<br>H,0,-0.5081890476,2.7911549348,5.2086351745<br>H,0,-2.2130145403,4.3638238434,4.5430821351<br>H,0,-4.448162914,3.7099973932,3.9150434404<br>H,0,-5.1486817968,1.4552810237,3.6099645884<br>H,0,-4.2198144231,1.0543729479,5.0330734846<br>H,0,-1.7565223601,1.5942391389,5.5971303169<br>H,0,-4.9363557545,-1.7334737964,1.3384212747<br>H,0,-4.7046814718,-1.8138113555,3.1030788919<br>H,0,-5.7212157805,-0.5553197383,2.3875137121<br>H,0,0.9103944418,-1.4469694594,2.3794555251<br>H,0,3.0031068117,-1.0859721844,1.0731470494<br>H,0,3.0928338548,0.704046747,-0.635391302<br>H,0,1.1140840445,2.136430025,-1.0467529817<br>H,0,-0.9748010665,1.7699449252,0.2633157104<br>H,0,-2.3274661231,-1.5922369804,1.1908940498<br>Pd,0,-3.5913502446,0.6287149644,0.1526478029<br>N,0,-4.8855246823,1.7741115468,-0.944252701<br>C,0,-5.5824354454,2.3939604592,-1.6180819746<br>C,0,-6.4632141295,3.1716647127,-2.4684565186<br>H,0,-7.3569441597,2.5915470584,-2.7072631834<br>H,0,-6.7590707941,4.0907214282,-1.9585883323<br>H,0,-5.9486078307,3.4295121497,-3.3964445173
**S16**	NH_R_3a.log<br><br>Low frequencies --- -13.9489 -13.4401 -12.9252 0.0005 0.0006 0.0008<br>Low frequencies --- 10.6701 24.3136 25.1720<br><br>Zero-point correction= 0.312525 (Hartree/Particle)<br>Thermal correction to Energy= 0.333848<br>Thermal correction to Enthalpy= 0.334792<br>Thermal correction to Gibbs Free Energy= 0.257562<br>Sum of electronic and zero-point Energies= -987.676174<br>Sum of electronic and thermal Energies= -987.654852

	Sum of electronic and thermal Enthalpies= -987.653907<br>Sum of electronic and thermal Free Energies= -987.731138<br><br>O,0,0.0069035945,0.154338928,0.0184561349<br>C,0,0.0547620806,0.0124724015,1.3146583718<br>C,0,0.9789498753,0.0214551182,2.2933971013<br>C,0,0.2447852514,-0.25339848,3.5285554725<br>C,0,0.8609062115,-0.3479542779,4.8787694325<br>N,0,-1.0374560064,-0.4054509278,3.3904040553<br>N,0,-1.2710012696,-0.2216964176,1.9346233036<br>C,0,-2.2825016827,0.8310081137,1.674434449<br>C,0,-1.8837757653,2.158670118,1.6773501755<br>C,0,-2.8558691682,3.1301522502,1.4646326503<br>C,0,-4.1867935556,2.765841413,1.2625291436<br>C,0,-4.5574035626,1.4229616339,1.2677090535<br>C,0,-3.5959874579,0.4369215219,1.4763039312<br>C,0,2.4444091033,0.3251799242,2.1998103288<br>C,0,2.8353948995,1.2536612905,1.0719341219<br>Pd,0,1.5816424412,2.9587810035,0.8069094959<br>N,0,0.9031361138,4.7545571679,1.6237160406<br>C,0,0.6455338916,5.8204244731,1.97457123<br>C,0,0.3373707863,7.1669121747,2.4183428432<br>C,0,1.2860915927,0.221676672,-0.7107973785<br>C,0,2.2791277368,1.2346221501,-0.2116770977<br>H,0,0.9704365144,0.4510727799,-1.7243762249<br>H,0,2.8801573185,1.6419951436,-1.0217630751<br>H,0,3.8290391056,1.6781712606,1.1824422018<br>H,0,2.758649686,0.7738543165,3.1449074285<br>H,0,3.0163841003,-0.611389155,2.1149907109<br>H,0,1.6925564478,-0.7953486468,-0.6857062592<br>H,0,0.1101280502,-0.6067632804,5.6233170195<br>H,0,1.6538166089,-1.1002758897,4.8819043604<br>H,0,1.3160159407,0.6101054483,5.1461144035<br>H,0,-3.8749898671,-0.6109539814,1.4916237978<br>H,0,-5.5898302809,1.138955745,1.1093303722<br>H,0,-4.9365222301,3.5292783914,1.0954298054<br>H,0,-2.5669254517,4.1734295571,1.4479087082<br>H,0,-0.8414651106,2.4324513403,1.8088736046<br>H,0,-0.2953170261,7.135872355,3.3076355885<br>H,0,1.2618595254,7.6968718978,2.65718404<br>H,0,-0.1849652558,7.7079637867,1.626599775<br>H,0,-1.6492069809,-1.1147204585,1.6017932318
MeCN	ACN_SMD.log<br><br>Low frequencies --- -26.4452 -21.9287 -0.0014 -0.0011 -0.0008 32.3575<br>Low frequencies --- 401.5251 402.5779 932.9815<br><br>Zero-point correction= 0.045047 (Hartree/Particle)<br>Thermal correction to Energy= 0.048621<br>Thermal correction to Enthalpy= 0.049566<br>Thermal correction to Gibbs Free Energy= 0.021065<br>Sum of electronic and zero-point Energies= -132.766892<br>Sum of electronic and thermal Energies= -132.763317<br>Sum of electronic and thermal Enthalpies= -132.762373<br>Sum of electronic and thermal Free Energies= -132.790874<br><br>C,0,0.0001283283,-0.0000059461,-0.0021245724<br>C,0,0.0000277465,-0.0007162794,1.4482185438<br>N,0,-0.0000454568,0.0004906328,2.6020942087<br>H,0,-0.9576746742,0.3716369199,-0.3721309148<br>H,0,0.8007370039,0.6437579869,-0.3723645219<br>H,0,0.1569345309,-1.0151898995,-0.3724060583
**1a**	R7b_SMD.log<br><br>Low frequencies --- -15.2305 -13.1281 -4.6286 -0.0004 -0.0004 -0.0002<br>Low frequencies --- 27.4166 43.4270 58.7770<br><br>Zero-point correction= 0.251908 (Hartree/Particle)<br>Thermal correction to Energy= 0.266318<br>Thermal correction to Enthalpy= 0.267262<br>Thermal correction to Gibbs Free Energy= 0.209131<br>Sum of electronic and zero-point Energies= -726.619483<br>Sum of electronic and thermal Energies= -726.605073<br>Sum of electronic and thermal Enthalpies= -726.604129<br>Sum of electronic and thermal Free Energies= -726.662260<br><br>C,0,-0.0042284582,-0.1175886283,0.0072404023<br>C,0,0.0056201955,-0.0078064272,1.4001649427<br>C,0,1.2157077349,0.1082652175,2.0899402358<br>C,0,2.4138893888,0.1147693164,1.3814184607

	C,0,2.4128624297,0.0205234237,-0.010555839<br>C,0,1.2009853593,-0.092705333,-0.690978795<br>N,0,-1.206089333,-0.0325568645,2.1336383002<br>C,0,-2.4186212027,0.5231121507,1.8153277242<br>C,0,-3.2835480418,0.2896610273,2.864117018<br>C,0,-2.5012629451,-0.4363241654,3.803841206<br>N,0,-1.2603312367,-0.632135644,3.3661419597<br>O,0,-2.59022245,1.1655667802,0.6491450879<br>C,0,-3.236980051,2.462226258,0.7432558263<br>C,0,-4.6839289658,2.4389631915,1.1476989282<br>C,0,-5.2913597562,1.687273891,2.0692597815<br>C,0,-4.7368891016,0.6292093517,2.9792521254<br>C,0,-2.9481145578,-0.9737847736,5.1229698137<br>H,0,-3.142055202,2.8777195475,-0.2589968014<br>H,0,-5.2913683914,3.1667135261,0.6154308048<br>H,0,-6.3583337925,1.8493425796,2.2037360904<br>H,0,-4.9559627988,0.9356552774,4.0103927309<br>H,0,-5.3368948468,-0.281073034,2.8370963623<br>H,0,-2.6446739763,3.0838147226,1.4238595444<br>H,0,-2.1199614163,-1.4733818513,5.62880105<br>H,0,-3.3179264445,-0.1725411618,5.7702089433<br>H,0,-3.7639317545,-1.6930063869,5.0004722689<br>H,0,1.2092175013,0.1932627098,3.168471569<br>H,0,3.3501512264,0.2036562611,1.9205355218<br>H,0,3.3475497238,0.032012617,-0.5589894312<br>H,0,1.1890996041,-0.1769742351,-1.7716513594<br>H,0,-0.9403010673,-0.2268423761,-0.5219323406
**5**	R7_1b_SMD.log<br><br>Low frequencies --- -13.6718 -11.5925 -5.2754 0.0001 0.0001 0.0004<br>Low frequencies --- 13.4844 21.2684 35.4833<br><br>Zero-point correction= 0.299414 (Hartree/Particle)<br>Thermal correction to Energy= 0.320458<br>Thermal correction to Enthalpy= 0.321402<br>Thermal correction to Gibbs Free Energy= 0.245561<br>Sum of electronic and zero-point Energies= -987.363406<br>Sum of electronic and thermal Energies= -987.342363<br>Sum of electronic and thermal Enthalpies= -987.341418<br>Sum of electronic and thermal Free Energies= -987.417260<br><br>C,0,-0.0370657227,0.470668387,-0.0278043346<br>C,0,0.0170309457,0.1733251746,1.3365443327<br>C,0,1.2450682495,-0.0746970162,1.9551962932<br>C,0,2.4172479909,-0.0247838884,1.2056133253<br>C,0,2.3709439925,0.2571249113,-0.1597415191<br>C,0,1.1409904521,0.501619096,-0.7703542251<br>N,0,-1.1658909028,0.140843789,2.1165627561<br>N,0,-1.1573925605,0.6063850956,3.410832103<br>C,0,-2.3833367596,0.3785071586,3.8720264953<br>C,0,-3.2118198058,-0.2530622947,2.9035933739<br>C,0,-2.3874867364,-0.3894996345,1.7979182092<br>O,0,-2.5435005002,-0.9554473299,0.5951403536<br>C,0,-3.905393001,-1.2077490121,0.1620002065<br>C,0,-4.7020748565,-2.1726573522,1.0060590327<br>Pd,0,-3.6091510233,-3.5217335442,2.2549247592<br>N,0,-2.4603411793,-4.906133621,3.2820148185<br>C,0,-1.7919693493,-5.6742192452,3.8221501431<br>C,0,-0.957437981,-6.6345337252,4.5097415171<br>C,0,-4.6263783757,-0.7126443054,3.1168229279<br>C,0,-5.0571377604,-1.9300372382,2.3321304836<br>C,0,-2.7621476328,0.7635390658,5.2640638813<br>H,0,-3.7848458392,-1.6001412892,-0.8455387875<br>H,0,-5.3488034272,-2.8192768001,0.4164135768<br>H,0,-5.9546764514,-2.4119800766,2.712825514<br>H,0,-4.7602240393,-0.9172716472,4.1822077156<br>H,0,-5.3342692355,0.0999773923,2.8872037053<br>H,0,-4.4136950422,-0.2379744362,0.0976588968<br>H,0,-1.9031305026,1.1924300367,5.7833369963<br>H,0,-3.5722474337,1.4992597472,5.2668970972<br>H,0,-3.110996198,-0.1054473848,5.8305146275<br>H,0,-0.9876453641,0.6776579113,-0.4989664945<br>H,0,1.0954070778,0.7313904238,-1.8287894663<br>H,0,3.2846645531,0.2885607601,-0.7417887482<br>H,0,3.3677157795,-0.2171042302,1.6901824763<br>H,0,1.2718484176,-0.3054142659,3.011891257<br>H,0,-1.2490226092,-6.6834738628,5.5614012036<br>H,0,-1.0795000381,-7.6208883196,4.0569234251<br>H,0,0.0896360594,-6.3316465745,4.4401365973
**6**	I_3_SMD_2.log

	Low frequencies --- -16.0859 -10.3085 -7.7929 -0.0003 0.0002 0.0003<br>Low frequencies --- 19.9211 25.1465 30.8417<br><br>Zero-point correction= 0.297094 (Hartree/Particle)<br>Thermal correction to Energy= 0.318342<br>Thermal correction to Enthalpy= 0.319286<br>Thermal correction to Gibbs Free Energy= 0.244058<br>Sum of electronic and zero-point Energies= -987.389903<br>Sum of electronic and thermal Energies= -987.368655<br>Sum of electronic and thermal Enthalpies= -987.367711<br>Sum of electronic and thermal Free Energies= -987.442940<br><br>C,0,0.0214937325,0.1027389246,0.0282462903<br>C,0,0.017674579,0.036176925,1.4296677492<br>C,0,1.2373855785,-0.0580412443,2.1160993507<br>C,0,2.4364712522,-0.0832259579,1.4100298412<br>C,0,2.4447409628,-0.0097525139,0.016769267<br>C,0,1.2307329618,0.0842415807,-0.663094571<br>N,0,-1.1862670258,0.0394650725,2.1591564656<br>N,0,-1.2036722814,-0.4556010338,3.4548058118<br>C,0,-2.4540550857,-0.2774395047,3.873123733<br>C,0,-3.2711075526,0.3268494214,2.8906233694<br>C,0,-2.4328501634,0.5383478751,1.7827930288<br>O,0,-2.6297185555,1.0783942218,0.6253464359<br>Pd,0,-4.6285431104,1.5554126174,0.0556707231<br>N,0,-4.0003983988,2.3831115892,-1.7764028586<br>C,0,-3.6244400692,2.8010315313,-2.7795934973<br>C,0,-3.1542763503,3.3273903765,-4.0416653891<br>C,0,-4.7066217293,0.7591080322,3.0347676859<br>C,0,-5.4940946782,0.6320180076,1.7571486319<br>C,0,-6.3732184003,1.6411144422,1.2992071202<br>C,0,-6.7859996479,1.5954193103,-0.0480261958<br>C,0,-2.8588428647,-0.707661077,5.2464689056<br>H,0,-4.7747441357,1.7951122535,3.3895061581<br>H,0,-6.4760675918,2.5601538326,1.8688849305<br>H,0,-3.1961580538,0.1438289854,5.8461745448<br>H,0,-3.6851683922,-1.4246351235,5.2117187473<br>H,0,-2.0169029375,-1.1768387896,5.7598383281<br>H,0,-0.9153119622,0.176200277,-0.5025914955<br>H,0,1.218338348,0.1360511525,-1.7463828386<br>H,0,3.3809964127,-0.0260610105,-0.5289996704<br>H,0,3.3704913764,-0.155588511,1.9563825665<br>H,0,1.2339683221,-0.1108769677,3.1957272934<br>H,0,-5.1836648335,0.1373777928,3.8021925337<br>H,0,-5.619297244,-0.3783130879,1.3692348001<br>H,0,-7.2823572989,2.4577681077,-0.4785600025<br>H,0,-6.9800961892,0.6415623608,-0.5310291058<br>H,0,-3.6151942935,2.7745736496,-4.8630707597<br>H,0,-3.4230049575,4.3829546928,-4.1212775301<br>H,0,-2.0686008585,3.2251862091,-4.1003923236
TS**5/6**	TS_ACN_3a_SMD.log<br><br>Low frequencies --- -321.0366 -14.0210 -7.8342 -4.4341 0.0003 0.0006<br>Low frequencies --- 0.0007 13.4078 19.1988<br><br>Zero-point correction= 0.294744 (Hartree/Particle)<br>Thermal correction to Energy= 0.316065<br>Thermal correction to Enthalpy= 0.317009<br>Thermal correction to Gibbs Free Energy= 0.240405<br>Sum of electronic and zero-point Energies= -987.322176<br>Sum of electronic and thermal Energies= -987.300856<br>Sum of electronic and thermal Enthalpies= -987.299912<br>Sum of electronic and thermal Free Energies= -987.376515<br><br>N,0,0.0124195618,-0.1224071327,-0.0663804425<br>N,0,-0.04885552,-0.1170785665,1.3094723524<br>C,0,1.2316344058,-0.1003282764,1.8812919689<br>C,0,2.1230603803,-0.078694949,0.7960180826<br>C,0,1.3109988627,-0.0954005341,-0.3614027157<br>C,0,-1.2987376837,-0.096389538,1.9554825026<br>C,0,-2.4296134586,0.3531003424,1.2586781964<br>C,0,-3.6737756516,0.3652710724,1.8824369435<br>C,0,-3.8118297881,-0.0575618616,3.2049107529<br>C,0,-2.6849298913,-0.5035210847,3.8950877562<br>C,0,-1.4348120577,-0.5328593977,3.2813928056<br>O,0,1.458870858,-0.0402434432,3.1451027721<br>C,0,3.6244041935,-0.079508508,0.7993871013<br>C,0,4.4050033091,0.1305441669,2.075566873<br>C,0,4.0841017883,0.994470208,3.1535441268<br>C,0,2.858367042,1.6401191381,3.3246161966

	C,0,1.7679924348,-0.1108447259,-1.7836106372<br>Pd,0,3.8827231828,-1.2177543238,3.5834825445<br>N,0,3.4312918471,-2.6387658693,5.0810866567<br>C,0,3.1659160783,-3.4783780556,5.8241153418<br>C,0,2.8306237027,-4.5270225581,6.7626318907<br>H,0,2.633695003,2.1009071948,4.280115905<br>H,0,4.840069856,1.1460519564,3.9231904852<br>H,0,5.4666228233,-0.0801103236,1.9425692205<br>H,0,3.9820485602,-1.0274845957,0.371664806<br>H,0,3.9821933646,0.6810335559,0.0868311415<br>H,0,2.2657113577,1.9686459848,2.4847228908<br>H,0,2.3730108874,0.7718827197,-2.015305581<br>H,0,2.3893821333,-0.9881256461,-1.9914462389<br>H,0,0.9101623781,-0.1278789927,-2.4589490278<br>H,0,-0.5659463416,-0.8827144919,3.8178845348<br>H,0,-2.7756688808,-0.8428734068,4.9211371409<br>H,0,-4.7817024724,-0.0413828938,3.6883509603<br>H,0,-4.5388089424,0.715546256,1.3301549187<br>H,0,-2.323488222,0.6890647833,0.2366088579<br>H,0,2.9815898825,-5.5020896547,6.294186166<br>H,0,3.4689050806,-4.4488789751,7.6455215449<br>H,0,1.7850867369,-4.4304854898,7.0630581566
**5.**MeCN	R7_2_SMD.log<br><br>Low frequencies --- -17.7758 -14.3619 -8.7177 -0.0007 0.0002 0.0005<br>Low frequencies --- 13.9039 20.7002 34.3023<br><br>Zero-point correction= 0.346002 (Hartree/Particle)<br>Thermal correction to Energy= 0.371739<br>Thermal correction to Enthalpy= 0.372683<br>Thermal correction to Gibbs Free Energy= 0.286760<br>Sum of electronic and zero-point Energies= -1120.143102<br>Sum of electronic and thermal Energies= -1120.117366<br>Sum of electronic and thermal Enthalpies= -1120.116421<br>Sum of electronic and thermal Free Energies= -1120.202344<br><br>C,0,-0.0849104516,-0.0927424471,0.0326926392<br>N,0,-0.1388759111,-0.2234879943,1.3971579077<br>N,0,1.1311541008,-0.2554294839,1.9330661791<br>C,0,1.9533667782,-0.1531646159,0.8925526346<br>C,0,1.2521412717,-0.0514591195,-0.3381681484<br>C,0,-1.2625411362,-0.1532121733,2.254496964<br>C,0,-2.5030912991,-0.6686179566,1.8656944505<br>C,0,-3.5876790603,-0.5817520173,2.7358436156<br>C,0,-3.4456186002,-0.001940173,3.9956261146<br>C,0,-2.2029218656,0.5016955549,4.3802435291<br>C,0,-1.1149970417,0.4364259638,3.5149045477<br>C,0,3.4330149365,-0.1208226103,1.0920316619<br>C,0,1.8330293012,0.281281486,-1.6875123838<br>C,0,1.1383438657,1.4626527434,-2.3500858077<br>Pd,0,0.3131249565,2.8215395889,-0.9576081446<br>N,0,-1.3736366026,3.5147403299,0.2511531838<br>C,0,-2.2481231717,3.4070691621,0.9955762019<br>C,0,-3.3430892317,3.2340204908,1.9260882048<br>O,0,-1.2267012642,0.0151098899,-0.652725816<br>C,0,-1.1178443505,0.2861745177,-2.0857684295<br>C,0,-0.2773356203,1.4778025258,-2.4779918617<br>N,0,1.8498022821,3.6060229837,0.355409292<br>C,0,2.6744774787,3.6659128017,1.1591326111<br>C,0,3.7108619118,3.7131423615,2.1690303894<br>H,0,-2.1534292841,0.4295879658,-2.3873288045<br>H,0,-0.7076144362,2.0306090116,-3.3110561574<br>H,0,1.7280500208,1.9756917336,-3.1075570993<br>H,0,2.8918588668,0.5127730669,-1.5519680212<br>H,0,1.8006553269,-0.5890517797,-2.3602691657<br>H,0,-0.7468343827,-0.6357839871,-2.5501211553<br>H,0,3.6719822184,-0.1923899999,2.1546433956<br>H,0,3.927282033,-0.9450889318,0.5695560728<br>H,0,3.8579678705,0.8102861877,0.7044138717<br>H,0,-2.6171659809,-1.1336033003,0.89752828<br>H,0,-4.5463572675,-0.9822533076,2.4262393216<br>H,0,-4.2926246532,0.0579584852,4.6690338073<br>H,0,-2.0790100437,0.960344133,5.3547595588<br>H,0,-0.1537582433,0.8388377579,3.8033323168<br>H,0,4.6931636161,3.6503455438,1.6960043411<br>H,0,3.642042122,4.646674094,2.731527356<br>H,0,3.588752164,2.8715566265,2.8545114682<br>H,0,-3.7430979639,4.2066875746,2.2202739041<br>H,0,-4.1348707193,2.6467563786,1.4564288585<br>H,0,-2.9872349264,2.7073240983,2.8139098464
**6.**MeCN	I_32_SMDd.log

	Low frequencies --- -22.7923 -13.6563 -9.8416 -0.0003 -0.0001 0.0005<br>Low frequencies --- 8.9611 20.5470 27.3565<br><br>Zero-point correction= 0.343478 (Hartree/Particle)<br>Thermal correction to Energy= 0.369956<br>Thermal correction to Enthalpy= 0.370900<br>Thermal correction to Gibbs Free Energy= 0.281741<br>Sum of electronic and zero-point Energies= -1120.165442<br>Sum of electronic and thermal Energies= -1120.138964<br>Sum of electronic and thermal Enthalpies= -1120.138020<br>Sum of electronic and thermal Free Energies= -1120.227179<br><br>C,0,-0.1167476494,0.8073116196,-0.3352940223<br>C,0,-0.0066413197,0.3690290031,0.9912636836<br>C,0,1.2589484126,0.0871379997,1.5229526528<br>C,0,2.3949497702,0.2343583416,0.7318645696<br>C,0,2.2883636771,0.6549357132,-0.5942242364<br>C,0,1.0272268003,0.9384218348,-1.1180993847<br>N,0,-1.149690933,0.2124834055,1.7995639892<br>N,0,-1.03503185,0.2798359298,3.1790975553<br>C,0,-2.2482515836,-0.018818868,3.6387497462<br>C,0,-3.1636376048,-0.2996572585,2.5983247155<br>C,0,-2.4360456648,-0.1456590081,1.4076641573<br>O,0,-2.7529586639,-0.2953071315,0.1607651075<br>Pd,0,-4.584639204,-1.2966196783,-0.2950063374<br>N,0,-4.2407027952,-1.1442741798,-2.3695553417<br>C,0,-4.0231756251,-1.0400058764,-3.4938661244<br>C,0,-3.7530158628,-0.9062544437,-4.9077564192<br>C,0,-4.625720974,-0.6383705913,2.718110239<br>C,0,-5.0592950852,-1.6941626775,1.7341795518<br>C,0,-6.2644763563,-1.6138057591,0.9987917692<br>C,0,-6.4017025933,-2.4502338219,-0.1281988778<br>C,0,-2.5213454581,-0.0150769703,5.1088682504<br>N,0,-0.5188138504,-1.2279274333,-3.825115755<br>C,0,-0.716834697,-1.7538885198,-2.8169611835<br>C,0,-0.9765963256,-2.4027188828,-1.5480845035<br>H,0,-5.2552255877,0.2499141351,2.5847169442<br>H,0,-6.9095407381,-0.7480650356,1.1177236004<br>H,0,-3.2671891346,0.7411690456,5.3739208506<br>H,0,-2.9100582711,-0.9824451669,5.442059269<br>H,0,-1.6053412538,0.1977615451,5.6639423186<br>H,0,-1.0910065701,1.0255774842,-0.745754693<br>H,0,0.9272922109,1.2693951311,-2.1456759956<br>H,0,3.1748016215,0.7611550523,-1.2088948514<br>H,0,3.3679082733,0.010023786,1.155123991<br>H,0,1.3400464006,-0.2489027428,2.5475019032<br>H,0,-4.8173803736,-1.0056548563,3.7333388439<br>H,0,-4.5843010713,-2.6689468543,1.8409017375<br>H,0,-7.2098496719,-2.2671852876,-0.8274005844<br>H,0,-6.0020248155,-3.4606567167,-0.1141886885<br>H,0,-4.6833265338,-0.6943594395,-5.4393109233<br>H,0,-3.0465363822,-0.0900570365,-5.0686184607<br>H,0,-3.321576662,-1.8344962259,-5.2872312107<br>H,0,-0.0312018467,-2.6541415711,-1.0625475653<br>H,0,-1.5457527259,-1.7239095533,-0.9061139754<br>H,0,-1.5503730053,-3.3174897693,-1.711978609
$Pd(MeCN)_2$	PdACN2_SMD.log<br><br>Low frequencies --- -14.0837 -10.7448 -0.0006 -0.0005 -0.0004 5.0889<br>Low frequencies --- 27.4990 33.9371 44.9628<br><br>Zero-point correction= 0.093026 (Hartree/Particle)<br>Thermal correction to Energy= 0.103362<br>Thermal correction to Enthalpy= 0.104306<br>Thermal correction to Gibbs Free Energy= 0.054746<br>Sum of electronic and zero-point Energies= -393.500234<br>Sum of electronic and thermal Energies= -393.489899<br>Sum of electronic and thermal Enthalpies= -393.488955<br>Sum of electronic and thermal Free Energies= -393.538515<br><br>C,0,0.001151779,0.0182632039,-0.0069529928<br>C,0,0.0012981197,-0.003610919,1.4399371599<br>N,0,0.0008491426,-0.0205103637,2.5935579704<br>Pd,0,-0.0016958075,-0.0473241719,4.5858941101<br>N,0,-0.004223696,-0.0736435122,6.5782315834<br>C,0,-0.005760306,-0.0884644627,7.731880601<br>H,0,-1.0266868297,0.0235421891,-0.3767608635<br>H,0,0.5148268075,0.914054019,-0.3632957835<br>H,0,0.5154185452,-0.8658854585,-0.3904350468<br>C,0,-0.006796568,-0.1077396137,9.178783048

	H,0,1.0175163668,-0.1937016771,9.5485262832<br>H,0,-0.4492776346,0.8150481794,9.5607990852<br>H,0,-0.5895824103,-0.9593677922,9.5370354205
